# Complex NADASE Infusions Improve Clinical Outcome in Substance Use Disorder: Descriptive Annotation in Fifty Cases

**Published:** 2024-08-23

**Authors:** Kenneth Blum, Thomas Mclaughlin, Kai Uwe Lewandrowski, Alireza Sharafshah, Catherine Dennen, Panayotis K. Thanos, David Baron, Edward J. Modestino, Keerthy Sunder, Kevin T. Murphy, Milan Makle, Elizabeth Giley, Eric R. Braverman, John Giordano, Rajendra D. Badgaiyan

**Affiliations:** 1 Division of Addiction Research and Education, Center for Sports, Exercise and Mental Health, Western University Health Sciences, Pomona, USA; 2 Division of Reward Deficiency Clinical Research, SpliceGen Therapeutics, Inc., Austin, USA; 3 Division of Personalized and Translational Medicine, The Kenneth Blum Neurogenetic and Behavioral Institute, LLC., Austin, USA; 4 Department of Psychiatry, Wright University Boonshoft School of Medicine, Dayton, USA; 5 Division of Personalized Pain Therapy Research, Center for Advanced Spine Care of Southern Arizona, Tucson, USA; 6 Brain Lab, Department of Psychology, Curry College, Milton, USA; 7 Division of Personalized Neuromodulation Research, Sunder Foundation, Palm Springs, USA; 8 Department of Orthopaedics, Fundación Universitaria Sanitas Bogotá, D.C. Colombia; 9 Department of Orthopedics, Hospital Universitario Gaffree Guinle, Universidade Federal do Estado do Rio de Janeiro, Rio de Janeiro, Brazil; 10 Cellular and Molecular Research Center, School of Medicine, Guilan University of Medical Sciences, Rasht, Iran; 11 Department of Family Medicine, Jefferson Health Northeast, Philadelphia, USA; 12 Department of Psychology, State University of New York at Buffalo, Buffalo, New York, USA; 13 Department of Psychiatry, Stanford University School of Medicine, Palo Alto, USA; 14 Department of Psychiatry, UC Riverside School of Medicine, Riverside, USA; 15 Department of Radiation Oncology, University of California, San Diego, La Jolla, USA; 16 Division of Personalized Transmodulation, Peak Logic, LLC, Del Mar, California, USA; 17 Executive Counseling Program, JC’s Counseling and Recovery Center, Hollywood, Florida, USA; 18 Department of Psychiatry, Mt. Sinai University, Ichan School of Medicine, New York, USA

**Keywords:** Nicotinamide adenine dinucleotide infusions, Cravings, Anxiety depression, Dopamine homeostasis, Reward deficiency syndrome, Medication assistant treatment, NADASE

## Abstract

**Background:**

The present study relates to a method to treat and detoxify patients with substance use disorder (SUD) utilizing a series of nicotinamide adenine dinucleotide (NAD+) and enkephalinase infusions (NADASE) in subjects attending chemical dependency programs.

**Objective:**

The primary objective of the current investigation is to provide some additional clinical evidence to show that NAD+ other amino acids including d-phenylalanine, glycine and ananylglutamine dipeptide and Myer’s cocktail (B complex) infusions significantly attenuates substance craving behavior and concomitant psychiatric burden sequalae in poly-drug abusers attending both in-patient and out-patient level of care in a number of chemical dependency programs in orange country.

**Methods:**

At symmetry approximately 1,000 now performed approximately 1,000 infusions on 900 patients without any serious side effects pointing to the safety of this procedure. The study cohort (n = 50) as a subgroup consisted of highly addicted poly-drug mixed gender and varied ethnic individuals previously resistant to standard treatment with a range of failed treatment attempts from one to ten. Each patient included in this study received a minimum of 7 infusions for an average duration of four weeks. The data includes craving scores, anxiety, depression, and sleep. We utilized likert scales (1 – 10) self-reported responses, accomplished via a counselor to patient structured interview.

**Results:**

In summary considering all fifty subjects using wilcoxon signed rank tests and sign tests, we found the following significance comparing the baseline scores to post outcome scores after NAD infusions; craving scores (P = 1.063E-9); anxiety (P = 5.487E-7); and depression (P = 1.763E-4). There was an improvement trend in the number of sleep hours post infusions, it was non-significant (Pre 6.28, and post 7.34). Importantly, urine analysis of a standard panel of illicit drugs of abuse during the course of NAD infusions resulted in a subset of 40 patients tested at midway during infusions 100% of these patients tested negative.

**Conclusion:**

These annotated notes serve an important function showing patient to patient similarities and differences and contribute to the emerging literature concerning NAD efficacy in SUD.

## Introduction

In a PubMed search (2/27/21) using the search term NAD+ we found 64,344 results and for NADH we found 86,586 results. This is a plethora of literature related to various aspects of NAD/NADH on structure and function. Moreover, a PubMed search using the term NAD and addiction resulted in fifty-one articles. However, there is a paucity of clinical studies in humans with SUD and the incorporation of NAD infusions in this cohort. The earliest known published clinical on NAD+ for addiction was authored by O’ Hollaren in 1961 [1].

Potential risks in raising NAD+ levels in various clinical disorders using NAD+ precursors include the accumulation of putative toxic metabolites, tumorigenesis and promotion of cellular senescence. The take home message is thus NAD+ metabolism represents a promising target, and further studies are needed to recapitulate the preclinical benefits in human clinical trials Braidy et al. [2].

In the face of the current opioid crisis in America killing close to 800,000 people since 2004, and an estimated death rate daily of 127, there is a pressing need to find a safe side effect free solution. In 2020 the overdose death rate was 82,000 people. We are cognizant that for example, naltrexone implants have heuristic value, but may interfere with dopamine homeostasis and induce anti-reward benefits. This pilot demonstration study with a small N requires future stringent larger randomized double blinded placebo-controlled studies enabling a clearer interpretation.

This article of original research is proposing a unique combination coupling the compound NAD+ with known enkephalinase inhibitors (EI) including DL-Phenylalanine2 to help detoxify and treat individuals diagnosed with reward deficiency as manifested by SUD. In this article we describe intravenous therapy of a specialized cocktail basically consisting of complex vitamin B, NAD and DLPA, and other amino acids (NADASE).

**Figure 1:**
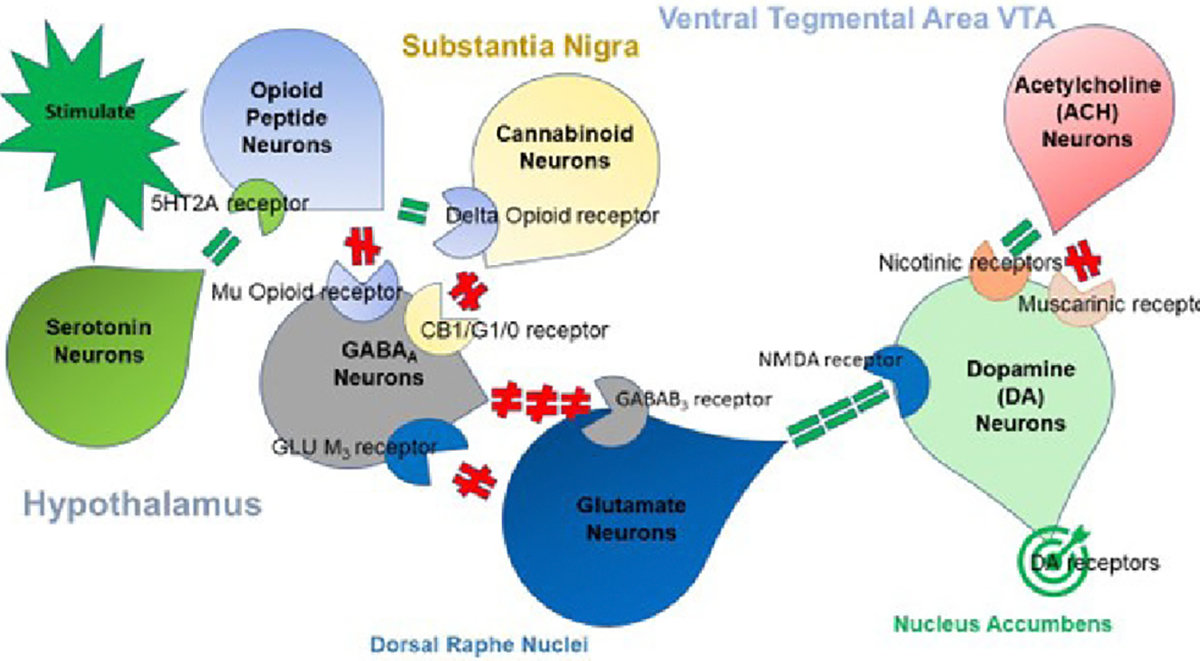
The brain reward cascade illustrates the interaction of at least seven major neurotransmitter-pathways involved in the BRC. In the hypothalamus, environmental stimulation causes the release of serotonin, which in turn via, for example, 5HT-2a receptors activate (green equal sign) the subsequent release of opioid peptides from opioid peptide neurons, also in the hypothalamus. Then, in turn, the opioid peptides have two distinct effects, possibly via two different opioid receptors. One that inhibits (red hash sign) through the mu-opioid receptor (possibly via enkephalin) and projecting to the substania nigra to GABAA neurons. Another stimulates (green equal sign) cannabinoid neurons (e.g., anandamide and 2-archydonoglcerol) through beta-endorphin linked delta receptors, which in turn inhibits GABAA neurons at the substania nigra. Cannabinoids primarily 2-archydonoglcerol, when activated, can also indirectly disinhibit (red hash sign) GABAA neurons in the substania nigra through activation of G1/0 coupled to CB1 receptors. Glutamate neurons located in the dorsal raphe nuclei (DRN) can indirectly disinhibit GABAA neurons in the substania nigra through activation of GLU M3 receptors (red hash sign). GABAA neurons, when stimulated, will, in turn, powerfully (red hash signs) inhibit VTA glutaminergic drive via GABAB 3 neurons. It is also possible that stimulation of ACH neurons that at the NAc ACH can stimulate both muscarinic (red hash) or nicotinic (green hash). Finally, glutamate neurons in the VTA will project to dopamine neurons through NMDA receptors (green equal sign) to preferentially release dopamine at the NAc shown as a bullseye indicates a euphoria, or “wanting” response. The result is that when dopamine release is low (unhappiness: endorphin deficiency). At the same time, general (usual) happiness depends on the dopamine homeostatic tonic set point [3].

Future studies must await both functional magnetic resonance imaging and positron emission tomography scanning to determine the acute and chronic effects of oral and or intravenous NADASE on numbers of D2 receptors and direct interaction at the nucleus accumbens (NAc). Confirmation of these results in large, population-based, case-controlled experiments is necessary. These studies would provide important information that could ultimately lead to significant improvement in recovery for those with RDS and dopamine deficiency as a result of a multiple neurotransmitter signal transduction breakdown in the brain reward cascade [3] (Figure 1).

While there is little evidence directly published on NAD/NAH and RDS and associated drug and non-drug addictions, there is some rationale to utilize this substance based on metabolic effects. However, until these studies are performed the undocumented use of this compound is not consistent with evidence based medical practice, in spite of many clinics using it for SUD as an off -label modality [4–36].

The following is a detailed original annotated executed pilot demonstration to assist in future larger required experiments in humans and animal self-administration models.

## Methods and Materials

The primary objective of the current investigation is to provide some additional clinical evidence to show that NADASE infusions significantly attenuates substance craving behavior and concomitant psychiatric burden sequalae in poly-drug abusers attending an out-patient chemical dependency program. NADASE infusions are displayed in table 1.

The use of intravenous administration of amino acids for the potential treatment of RDS was approved by the Path Foundation NY IRB along with approved consent forms signed by each participant. Our group at symmetry provided NAD infusions to two treatment facilities whereby for this investigation involving the 50 subsets of patients were derived from two programs located in the orange county area. Symmetry and their associated treatment facilities have now performed approximately 1,000 infusions on 900 patients without any serious side effects pointing to the safety of this procedure.

The study cohort (n = 50) as a subgroup consisted of highly addicted poly-drug mixed gender and ethnicity individuals previously resistant to standard treatment with a failed range of treatment attempts. The incorporation of the NAD infusions involved detoxification, in-patient residence and out-patients. Table 2 is a summary of the status of the demographics.

Scrutiny of this data reveal that the various dugs of choice in these 50 patients include opiates (heroin), methamphetamine, cocaine, ethanol, benzodiazepines, marijuana and spray cans of air duster. The study consisted of twenty-one (n = 21) females and twenty-nine (n = 29) males, age range from 21 to 61 years of age whereby females 34.7 ± 8.9 years and males 34.3 ± 6.4. The study participants consisted of 84% Caucasians, 4% African Americans, 2% Hispanic, 2% Arabic and 8% Native Americans. The years of use ranged from 5 to 33 with an average of 16.80 ± 6.83. The number of treatment facilities attended by these 50 patients ranged from first time to as high as 10 with an average of 2.1 ± 2.1. In these 50 patients the range of last use of any drug of abuse self-reported is from one day to 80 days with an average of 21.76 ± 15.28. The longest sobriety of these patients ranged from 4 days to 7 years with an average sobriety rate at 1.18 ± 1.41 years. In this cohort the years of use varied from 5 to 33 years with an average of 16.80 ± 6.83. Each patient included in this study received a minimum of 7 infusions for a duration of four weeks at one to two infusions per week. The data on a number of outcomes included: craving scores; anxiety, depression, and sleep. To measure the various behavioral related levels, while there are existing validated scales, we made the decision to utilize likert scales (1 – 10) self-reported responses, accomplished via a counselor to patient structured interview. It is to be noted that each of the two treatment centers used self-reported likert scales to obtain the data on 50 patients. Over the duration of these 7 infusions of NAD each of the subjects included in this study agreed as per treatment policy to be urine screened for the presence or absence of non-prescribed psychoactive drugs. The first and last urine samples on these subjects were analyzed and presented herein.

## Case Series Presentations

### Patient 1 (Female/42/meth/benzos)

#### Consultation:

42-year-old female patient presents today for complaints of DOC is Xanax, meth via IV, smoking, snorting with last use 4 years ago, and alcohol 1 pints daily. Last drink was 8/3, longest sobriety is 1 year, and this is client’s 1^st^ treatment facility. States had no OD and no seizures in the past. Currently “I have no energy daily, ++anxiety 8/10 and sleeping 3 – 4 h a night without sleeping medication, difficulty concentrating, and I feel dehydrated and not drinking water.”

#### Baseline:

Patient is in great spirits. Blood drawn from LAC via IV catheter per practitioner’s order i.e, 4 SST, 1 LAV, 1 UA, and HCG negative. Patient self-rates her anxiety and depression at a level 2 out of 10 (situational), and pain at a level 2 out of 10. The patient states she gets headaches when reading. Patient states having a good appetite by eating 3 meals a day but hardly drinking any water. Patient states having no BM issues and having a good active activity level. Patients tolerated treatment well with no pain or discomfort.

#### Patient reports:

Cravings 6/10; anxiety 2/10; depression 2/10; and hours of uninterrupted sleep 7.

#### Infusion 1:

Patient self-rates anxiety 2/10, and depression 0/10. Patient denies myalgia and patient reports drinking 48 oz of water and sleeping 7 – 8 h. Patient c/o constipation. Encouraged patients to increase water intake and fiber to help constipation. Patient was receptive and patient reports her sleep after her first infusion has improved and she wakes up with enough energy for the day. Patient was observed relaxing and watching TV during infusion and patient tolerated treatment well with no complaint of discomfort.

#### Patient reports:

Cravings 4/10; anxiety 2/10; depression 0/10; and hours of uninterrupted sleep 7.

#### Infusion 2:

Patient self-rates anxiety, depression, and cravings 0/10. Patient denies myalgia and patient reports drinking 8 cups of water, good appetite, no GI problems, slept 7 h. Patient reports her sleep has been great and she wakes up rested and energized. Patient was observed relaxing and reading during infusion and patient tolerated treatment well with no complaint of discomfort.

#### Patient reports:

Cravings 0/10; anxiety 0/10; depression 0/10; and hours of uninterrupted sleep 7.

#### Infusion 3:

Patient comes into the office in a good mood. The patient denies pain and has no fever. Patient states eating 4 meals a day and drinking approx. 80 oz of water daily. Patient states she has constipation and has a moderate activity level. Patient states she has felt like she has better focus and a clearer mind. Patients tolerated treatment well with no pain or discomfort.

#### Patient reports:

Cravings 0/10; anxiety 0/10; depression 0/10; and hours of uninterrupted sleep 6.

#### Practitioner FU:

Patient presents to clinic for follow up infusion services. Currently infusion 5, states “this is the best I’ve felt in a long time, I feel like I am getting cleaned from the inside out, my outlook is positive, and my mind is not foggy anymore.”

#### Infusion 4:

Patient states she is doing well. The patient is on her 5^th^ infusion and reports tolerating infusion well during the past visits. Patient denies cravings, anxiety, depression or pain. She admits she does not drink as much water as she knows she should. Water intake is reported at 24 – 32 oz a day, advised patient to increase water intake, patient expressed understanding. IV site dry and intact, patient tolerated infusion well.

#### Patient reports:

Cravings 0/10; anxiety 0/10; depression 0/10; and hours of uninterrupted sleep 8.

#### Infusion 5:

Patient reports feeling “amazing” she says she has been liking the infusions a lot and feels like she’s doing good for the body. Patient denies cravings, anxiety, depression and pain. Water intake is reported at 32 oz a day, advised patients to increase water intake and educated patients on importance of water intake. IV site dry and intact, patient tolerated infusion well.

#### Patient reports:

Cravings 0/10; anxiety 0/10; depression 0/10; and hours of uninterrupted sleep 8.

#### Infusion 6:

Patient reports “I’m feeling good, sleep is improving” patient denies cravings, anxiety, depression, and pain. Sleep is about 8 h an evening, she states she wakes up feeling rested and has improved energy. Water intake is poor at 32 oz a day, advised patients to increase water intake and educated patients on importance of water intake. IV site dry and intact, patient tolerated infusion well.

#### Patient reports:

Cravings 0/10; anxiety 0/10; depression 0/10; and hours of uninterrupted sleep 8.

#### Infusion 7:

**Figure 2:**
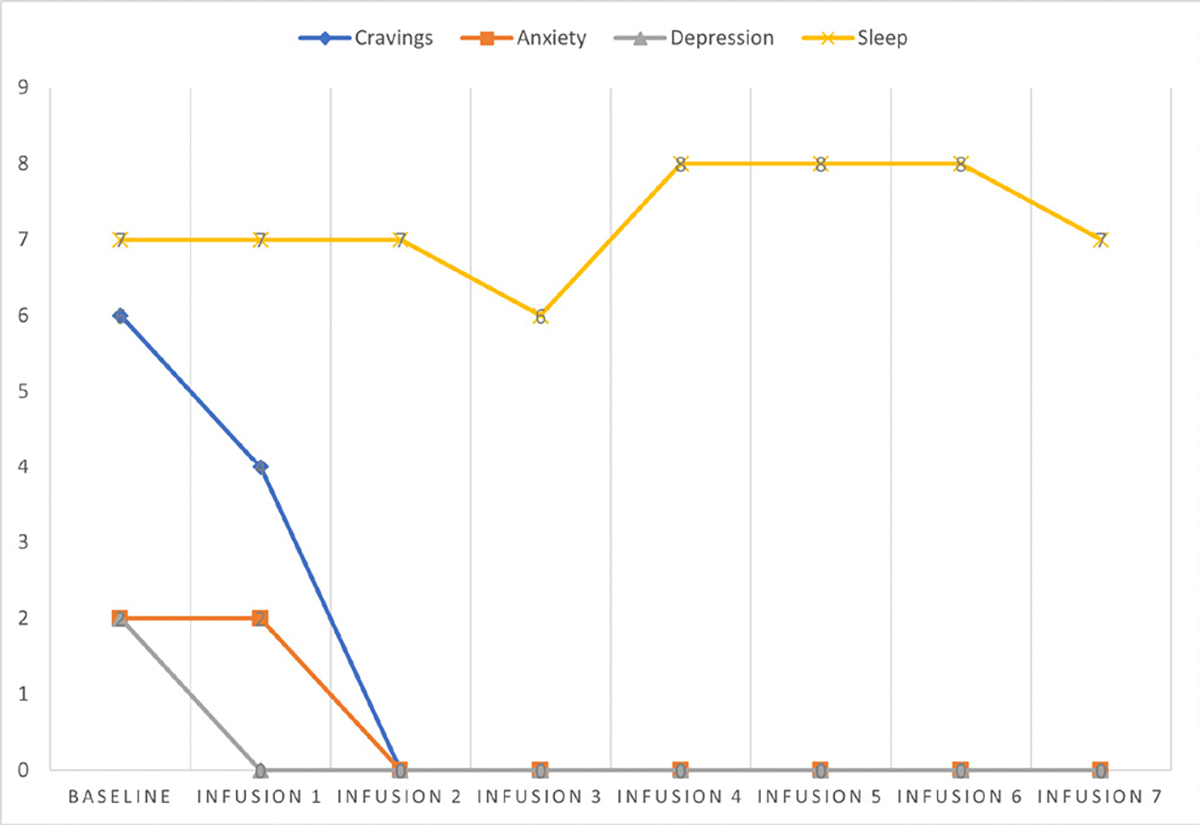
Patient 1 (Female/42/meth/benzos).

She says she has been liking the infusions because her mental clarity has improved, and she is able to focus during classes and meetings. Patient denies cravings, anxiety, depression, and pain. Water intake is reported at 32 oz a day, advised patients to increase water intake and educated patients on importance of water intake. IV site dry and intact, patient tolerated infusion well (Table 3 and figure 2).

#### Patient reports:

Cravings 0/10; anxiety 0/10; depression 0/10; and hours of uninterrupted sleep 7.

### Patient 2 (Female/35/meth/ETOH/duster)

#### Consultation:

35-year-old female patient presents today for complaints of DOC is meth via IV, and alcohol occasional last use was 15 days ago. In the past used to do dust inhalants last use in 2007. The longest sobriety is 9 months when she was pregnant. Has been to psych hospitals and 2 rehabs for alcoholism. Denies any OD in the past and many convulsions from dust inhalants. Currently 1. I have been anxious the last 2 – 3 days from issues at home and don’t know how to approach 8/10. 2. Also with trouble sleeping and on meds to help me sleep. 3. I have a really, really bad time with concentration and want to be tested for attention deficit hyperactivity disorder (ADHD). 4. I don’t drink a lot of water and am dehydrated.

#### Baseline:

Blood drawn from left dorsal hand from IV catheter per practitioner’s order, 4SST 1LAV 1UA obtained and sent to Integritox. Patient self-rates cravings 7/10, anxiety 8/10, and depression 5/10. Patient denies myalgia. Patient reports drinking 16 oz of water and sleeping 4 – 6 h with frequent awakening throughout the night. Encouraged patients to increase fluid intake throughout her day and educated her about the benefits of proper hydration. Patient was receptive. Patient reports being anxious and depressed due to being away from her kids and problems back home. The patient was observed restless frequently getting up and talking on her phone. Patient tolerated treatment well with no complaint of discomfort.

#### Patient reports:

Cravings 7/10; anxiety 8/10; depression 5/10; and hours of uninterrupted sleep 5.

#### Infusion 1:

Patient self-rates anxiety 8/10, cravings 6/10, and depression 7/10. Patient states the problems back home stress her out and raise her anxiety. Encouraged patients to practice meditation or breathing techniques to help with anxiety. Patient was receptive. Patient reports drinking 4 – 6 cups of water and sleeping 7 – 10 h. Patient c/o constipation l as BM a few days ago. Encouraged patients to increase water and fiber intake to help aid with digestion. Patient was receptive. Patient was observed relaxing and talking with peers during infusion. Patient tolerated treatment well with no complaint of discomfort.

#### Patient reports:

Cravings 6/10; anxiety 8/10; depression 7/10; and hours of uninterrupted sleep 6.

#### Infusion 2:

Patient self-rates cravings 3/10, depression 7/10 situational, and anxiety 6/10. Patient denies myalgia. Patient reports having increased her water intake to 8 cups and slept 6 h. Patient reports she feels great, and she notices a difference in her energy and her mood. Patient was excited to share her food journal progress and share the changes she has made to her diet. The patient has decreased her sugary drink intake and has been exercising. Patient has lost 4 lbs since she’s started changing her habits. Patient has started taking melatonin to help with her sleep. Encouraged patient to continue with healthy choices and to increase her fluid intake. Patient was receptive. The patient was observed relaxing watching TV during infusion. Patient tolerated treatment well with no complaint of discomfort.

#### Patient reports:

Cravings 3/10; anxiety 6/10; depression 7/10; and hours of uninterrupted sleep 5.

#### Infusion 3:

Patient states cravings at a level 3 out of 10, anxiety at a level 6 out of 10, and depression at a level 2 out of 10 and no pain. Patient states having a good appetite by eating 2 – 3 meals a day and drinking approx. 24 oz of water daily. Patient states no BM issues. Patient reports having slept for 4 h last night. Patients tolerated treatment well with no pain or discomfort.

#### Patient reports:

Cravings 3/10; anxiety 6/10; depression 2/10; and hours of uninterrupted sleep 4.

#### Infusion 4:

Patient self-rates anxiety7/10 situational, cravings 2/10, and depression 3/10. Patient denies myalgia. Patient reports staying active drinking 8 oz of water and sleeping 3 – 4 h. Encouraged patient to increase water intake and to minimize intake of caffeinated drinks. Patient was receptive. Patient continues to follow her diet but struggles with drinking water and avoiding energy drinks. Patient expressed understanding. Patient was observed to be restless and talking on the phone during infusion. Patient tolerated treatment well with no complaint of discomfort.

#### Patient reports:

Cravings 2/10; anxiety 7/10; depression 3/10; and hours of uninterrupted sleep 4.

#### Practitioner FU:

Client presents to office for NAD follow up. Currently on infusion 6. States “I feel fantastic, I have lost 4 lbs. and able to keep my diet going well because I am motivated. I am sleeping well and no anxiety at this time.”

#### Infusion 5:

Patient comes into the office for her 6^th^ visit. Patient self-rates no cravings, anxiety at a level 4 out of 10 (situational), no depression or pain. Recommended natural ways to help with anxiety such as yoga or meditation. Patient states having a good appetite by eating 2 – 3 meals a day and drinking approx. 36 oz of water daily. Patient states have no BM issues and have a high activity level. The patient stated she has been taking calcium and iron daily PO. Patients tolerated treatment well with no pain or discomfort.

#### Patient reports:

Cravings 0/10; anxiety 4/10; depression 0/10; and hours of uninterrupted sleep 5.

#### Infusion 6:

Patient self-rates her cravings at a level 0 out of 10, anxiety at a level 6 out of 10, depression at a level 3 out of 10, and no pain. Recommended natural ways to help with anxiety and depression such as yoga or meditation. The patient states eating 2 – 3 meals a day and drinking 16 – 24 oz of water daily. Patient states have no BM issues. Patient states she started taking TOPAMAX last week. Patient states having a low activity level. Patients tolerated treatment well with no pain or discomfort.

#### Patient reports:

Cravings 0/10; anxiety 6/10; depression 3/10; and hours of uninterrupted sleep 5.

#### Infusion 7:

Patient comes into the office for her 8th treatment. The patient self-rates her anxiety at a level 4 out of 10 and depression at a level 3 out of 10 (situational) and patient denies pain. Patient states having a good appetite by eating 2 – 3 meals a day and drinking approx. 36 oz of water daily. Patient reports no BM issues and having a high activity level. Patients tolerated treatment well with no pain or discomfort.

#### Patient reports:

**Figure 3:**
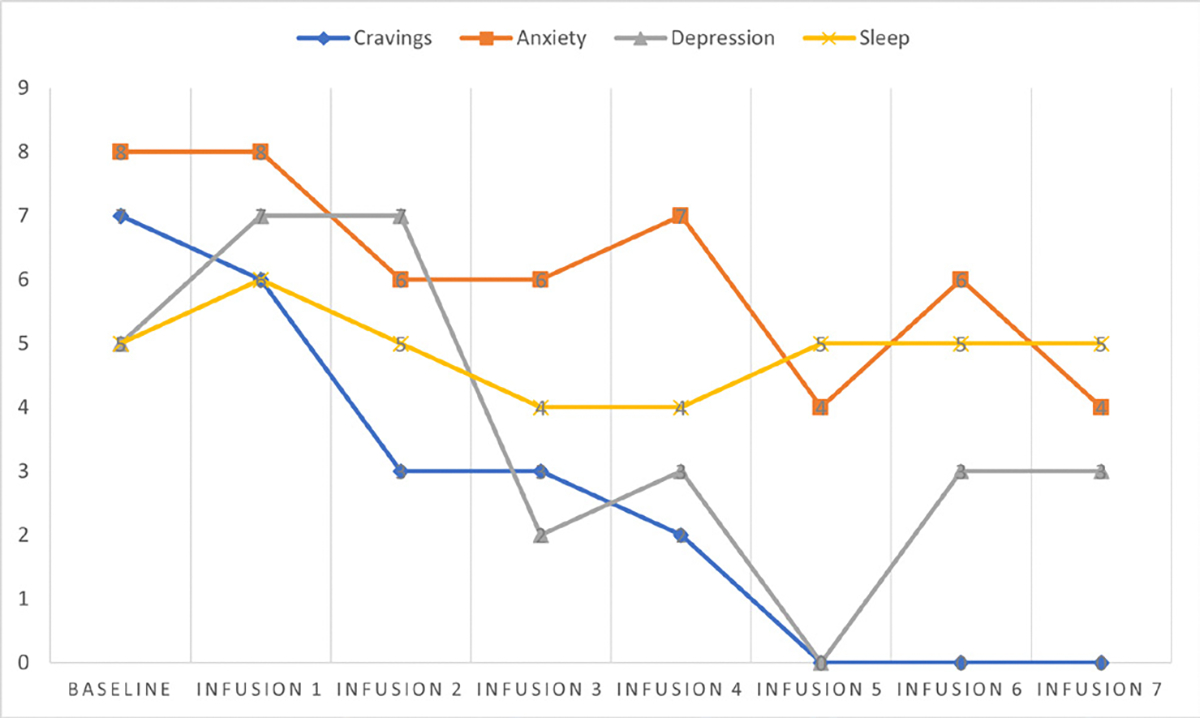
Patient 2 (Female/35/meth/ETOH/duster).

Cravings 0/10; anxiety 4/10; depression 3/10; and hours of uninterrupted sleep 5 (Table 4 and figure 3).

### Patient 3 (Female/27/meth)

#### Consultation:

27-year-old female patient presents today for complaints of “DOC is meth, via IV, smoking, snorting, last use being 6/17, this is clients 1^st^ treatment facility. The longest sobriety is 7 months. States had no OD or seizures in the past. States “I get very dehydrated every day and can’t drink enough water and makes me nauseous every day and makes me feel weak. I also suffer from daily anxiety and have a hard time concentrating.”

#### Baseline:

Patient reports to first infusion. Blood drawn from right AC from IV catheter per practitioner’s order. 4SST 1LAV 1UA obtained and sent to Integritox. Patient self-rates myalgia 5/10 to her ankle from an injury 5 years ago. Patient reports low activity and sleeping 8 – 9 h. Patient drinks about 2 – 3 cups of water daily. Encouraged patients to increase fluid intake and educated them on the importance of proper hydration. Patient was receptive. Patient reports no GI problems. The patient was observed relaxing watching TV during infusion. Patient tolerated treatment well with no complaint of discomfort.

#### Patient reports:

Cravings 5/10; anxiety 6/10; depression 0/10; and hours of uninterrupted sleep 8.

#### Infusion 1:

Patient self-rates anxiety, cravings at a 2/10 and her depression 0/10. Patient denies myalgia. Patient reports drinking half a gallon of water today and sleeping 9 h. Patient reports she woke up feeling well rested and not as foggy. The patient was observed relaxing during infusion. Patient tolerated treatment well with no complaint of discomfort.

#### Patient reports:

Cravings 2/10; anxiety 2/10; depression 0/10; and hours of uninterrupted sleep 9.

#### Infusion 2:

Patient self-rates anxiety, cravings and depression 0/10. Patient denies myalgia. Patient reports sleeping 8 h and drinking 16 oz of water. Encouraged patients to increase fluid intake and educated them on the importance of proper hydration. Patient was receptive. The patient reports feeling great overall, and she can concentrate better. Patient was observed relaxing and conversing with peers during infusion. Patient tolerated treatment well with no complaint of discomfort.

#### Patient reports:

Cravings 0/10; anxiety 0/10; depression 0/10; and hours of uninterrupted sleep 8.

#### Infusion 3:

Patient self-rates anxiety, depression and cravings 0/10. Patient denies myalgia. Patient reports sleeping 8 h and drinking 10 oz of water. Encouraged patients to increase fluid intake and reminded her of the importance of proper hydration. Patient was receptive. Patient reports her overall wellbeing improved with infusions. The patient was observed relaxing during infusion. Patient tolerated treatment well with no complaint of discomfort.

#### Patient reports:

Cravings 0/10; anxiety 0/10; depression 0/10; and hours of uninterrupted sleep 8.

#### Practitioner FU:

Client presents to clinic for NAD follow up, currently on infusion 4 as well as lab follow up. 1. Increased LDL, denies hx of high cholesterol and denies any diet restrictions. 2. Decreased vit D levels. 3. Decreased b12 and decreased energy levels. States NAD has been really good for her “I have noticed I have little to no anxiety, my nausea has subsided and I’m able to concentrate better.

#### Infusion 4:

Patient self-rates anxiety, depression, cravings 0/10. Patient denies myalgia. Patient reports drinking 80 oz of water and sleeping 8 – 9 h. Good appetite and no GI complaints. Patient reports she feels more alert and is able to concentrate better. Patient reports feeling great. The patient was observed relaxing watching TV during infusion. Patient tolerated treatment well with no complaint of discomfort.

#### Patient reports:

Cravings 0/10; anxiety 0/10; depression 0/10; and hours of uninterrupted sleep 9.

#### Infusion 5:

Client states she feels fine, client reports transitioning to IOP program and feels good about it. Client has a visible injury in her left leg, client admits she had broken it and now her leg is in a brace, client reports no pain felt and she has a physician who monitors it. Client reports drinking about 60 oz of water a day and sleeping 8 h. Sleep is good but sometimes clients wake up in the middle of the night and find difficulty falling back to sleep. Advised client to avoid liquids close to bedtime and to practice meditation and breathing exercises to help fall back to sleep client expressed understanding. IV site dry and intact, client tolerated infusion well.

#### Patient reports:

Cravings 0/10; anxiety 0/10; depression 1/10; and hours of uninterrupted sleep 8.

#### Infusion 6:

Client reports feelings good. Client appears to be in a good mood, good mood is evidenced by client actively participating in conversation and joking around with staff and client. Client self-rates her anxiety and depression at a 1 out of 10. Client states it’s not intolerable and does not affect how to carries herself throughout her day. Advised client to practice meditation and breathing techniques when necessary. IV site dry and intact, client tolerated infusion well.

#### Patient reports:

Cravings 0/10; anxiety 1/10; depression 1/10; and hours of uninterrupted sleep 8.

#### Infusion 7:

Client states, “I feel fine” client appears to be in a good mood. Good mood is evidenced by client’s talkative nature and client joking around. Client reports sleep is at 8 h an evening, client says sleep is restful. Client drinks about 40 oz of water a day, advised client to increase water intake. IV site dry and intact, client tolerated infusion well.

#### Patient reports:

**Figure 4:**
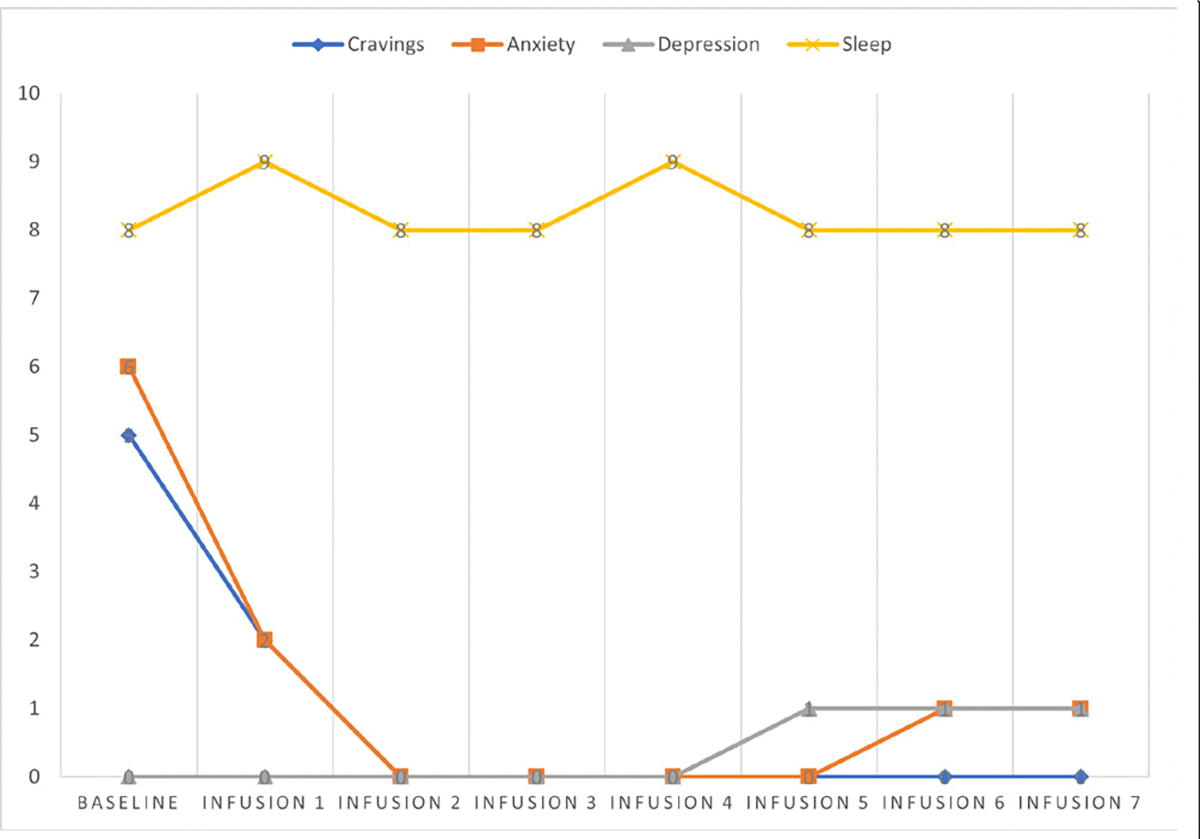
Patient 3 (Female/27/meth).

Cravings 0/10; anxiety 1/10; depression 1/10; and hours of uninterrupted sleep 8 (Table 5 and figure 4).

### Patient 4 (Female/41/ETOH)

#### Consultation:

41-year-old female patient presents today for complaints of “anxiety and insomnia”. The patient has a 20+ year history of alcohol abuse. Has remained sober since 5/15/2020. Patient has a history of alcohol addiction for 20+ years. She has remained sober since 5/15/2020. Her longest sobriety has been around 6 months.

#### Baseline:

Patient reports cravings at level 5 of 10, anxiety and depression at level 6 of 10 due to emotional/situational, advised of different ways to help lower anxiety and depression such as exercise and meditation. No pain, no fever. Slept for 8 h last night. Eat 3 meals and drink 60 oz of water daily. Low energy level. Tolerated treatment well with no pain or discomfort.

#### Patient Reports:

Cravings 5/10; anxiety 6/10; depression 6/10; and hours of uninterrupted sleep 8.

#### Infusion 1:

No cravings, no anxiety, depression at level 2 of 10 due to (situational/emotional) advised of different ways to help lower anxiety and depression such as exercise and meditation. No pain, no fever. Slept for 7 h last night. Eat 3 meals and drink 60 oz of water. Smokes 5 to 10 cigarettes per day. Tolerated treatment well with no pain or discomfort.

#### Patient reports:

Cravings 0/10; anxiety 0/10; depression 2/10; and hours of uninterrupted sleep 7.

#### Infusion 2:

No cravings, anxiety at level 3 of 10, no depression, anxiety due to being away from home. Advised different ways to help lower anxiety and depression such as exercise and meditation. Slept for 5 h last night no fever, increased energy and reports being able to sleep better good appetite eats 3 meals and drinks 70 oz of water. Smoke 10 cigarettes per day. Tolerated treatment well with no pain or discomfort.

#### Patient reports:

Cravings 0/10; anxiety 3/10; depression 0/10; and hours of uninterrupted sleep 5.

#### Infusion 3:

Cravings at level 1 of 10, anxiety at level 2 of 10, no depression, anxiety due to situational. Advised different ways to help lower anxiety such as exercise and meditation. Slept for 7 h last night no pain, no fever, good appetite eats 3 meals and drinks 60 oz of water. Smoke 10 cigarettes per day. Increased mental clarity and energy level. Tolerated treatment well with no pain or discomfort.

#### Patient reports:

Cravings 1/10; anxiety 2/10; depression 0/10; and hours of uninterrupted sleep 7.

#### Infusion 4:

No cravings, anxiety and depression at level 3 of 10 due to situational/emotional, advised of different ways to help lower anxiety and depression such as exercise and meditation. Slept for 6 h last night. Good appetite eats 3 meals and drinks 70 oz of water daily. Smoke 10 cigarettes per day. Increased energy level. Tolerated treatment well with no pain or discomfort.

#### Patient reports:

Cravings 0/10; anxiety 3/10; depression 3/10; and hours of uninterrupted sleep 7.

#### Infusion 5:

No cravings, no depression, anxiety at level 2 of 10 due to situational/emotional, advised of different ways to help lower anxiety and depression such as exercise and meditation. Slept for 8 h last night. Good appetite eats 3 meals and drinks 50 oz of water. Smoke 10 cigarettes per day. Increased energy level and mental clarity. Tolerated treatment well with no pain or discomfort.

#### Patient reports:

Cravings 0/10; anxiety 2/10; depression 0/10; and hours of uninterrupted sleep 8.

#### Infusion 6:

No cravings no anxiety, depression at level 2 of 10 due to situational/emotional, advised of different ways to help lower anxiety and depression such as exercise and meditation. No pain, no fever. Slept for 6 h last night. Patient reports better sleep. Good appetite, eats 3 meals and drinks 70 oz water, advised to drink more water. Smokes tolerated treatment well with no pain or discomfort.

#### Patient reports:

Cravings 0/10; anxiety 0/10; depression 2/10; and hours of uninterrupted sleep 6.

#### Infusion 7:

No cravings no anxiety, depression at level 2 of 10 due to situational/emotional, advised of different ways to help lower anxiety and depression such as exercise and meditation. No pain, no fever. Slept for 8 h last night. Good appetite eats 3 meals and drinks 80 oz water. smokes. Tolerated treatment well with no pain or discomfort.

#### Patient reports:

**Figure 5:**
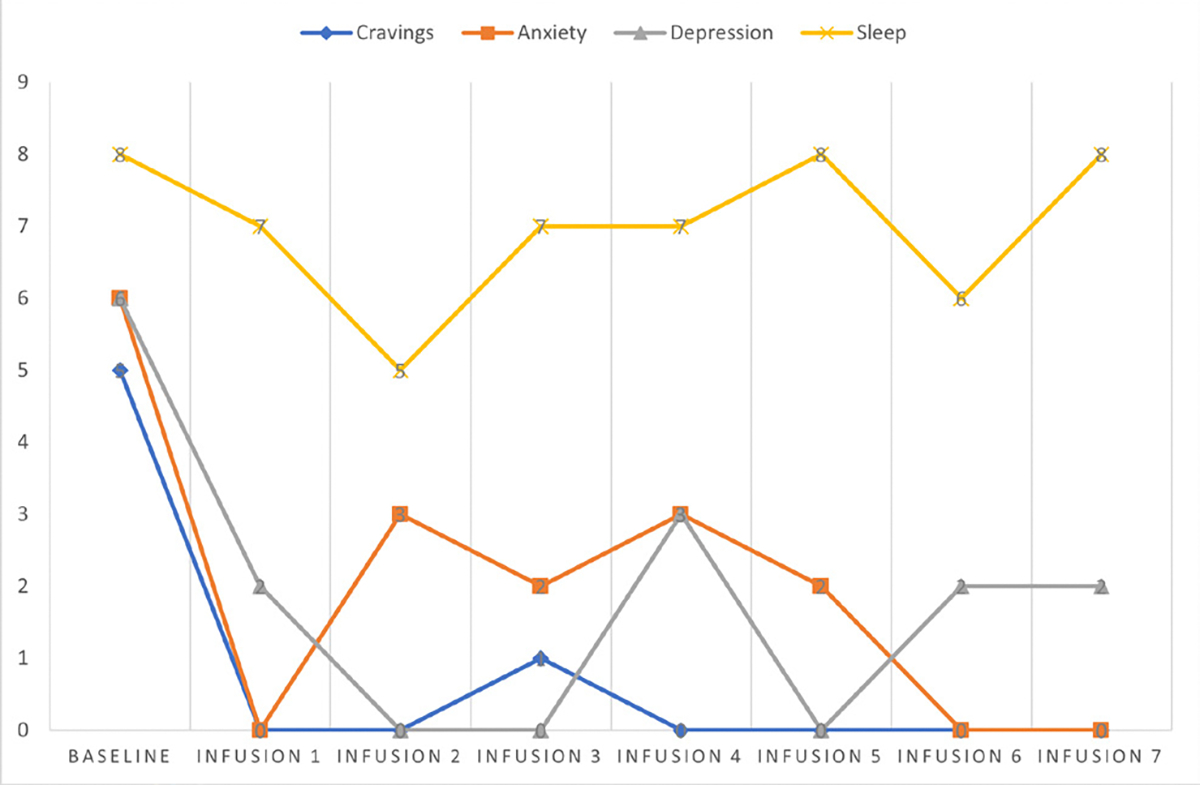
Patient 4 (Female/41/ETOH).

Cravings 0/10; anxiety 0/10; depression 2/10; and hours of uninterrupted sleep 8 (Table 6 and figure 5).

### Patient 5 (Female/45/meth)

#### Consultation:

45-year-old female presents for first time infusion treatments. Patients have a history of methamphetamine abuse. Started using meth around 22 years old. Last use was on August 11, 2020. This was her longest sobriety. She does not have a history of alcohol abuse. She smokes cigarettes, around 1 pack a day. She also stated she uses marijuana every day.

#### Baseline:

Patient comes into the office in a good mood. Blood will get drawn on next visit since today is Friday. Negative HCG. Patient self-rates her anxiety, depression and pain at 0/10. Patient states having a good appetite by eating 3 meals a day and drinking approx. 3 – 4 cups of water daily. Explaining the importance of drinking more water, the patient was receptive. Patient states having no BM issues and having a moderate activity level. Patients tolerated treatment well with no pain or discomfort.

#### Patient reports:

Cravings 8/10; anxiety 0/10; depression 0/10; and hours of uninterrupted sleep 7.

#### Infusion 1:

Blood drawn from L AC via IV catheter per practitioners’ orders. 4 SST, 2 LAV. BW sent to integritox. Patient is in a good mood. She self-rates her anxiety, depression and pain at 2/10. Patient states she hit her R hand on a door right above pinky finger and has been throbbing. Was told might be a cyst. Recommended to put ice to bring swelling down. Patient was receptive. Patient states having a good appetite by eating 3 meals a day and drinking approx. 6 cups of water daily. Told patient the importance of drinking more water. Patient was receptive. Patient states having no BM issues and having a moderate activity level. Patients tolerated treatment well with no pain or discomfort.

#### Patient reports:

Cravings 2/10; anxiety 2/10; depression 2/10; and hours of uninterrupted sleep 5.

#### Infusion 2:

Patient self-rates anxiety, depression and cravings 0/10. Patient denies myalgia. Patient reports good appetite 3 meals and drinks 3 – 4 cups of water. Encouraged patients to increase their water intake. Patient was receptive. No GI complaints, slept for 5 h. Patient reports moderate activity throughout the day. Patient reports she had a draining day with classes but has noticed she can pay attention for longer. Patient reports her memory and mental clarity have improved. The patient was observed relaxing watching TV during infusion. Patient tolerated treatment well with no complaint of discomfort.

#### Patient reports:

Cravings 0/10; anxiety 0/10; depression 0/10; and hours of uninterrupted sleep 5.

#### Infusion 3:

Patient reports that she enjoys receiving the infusions and believes her energy and mental clarity have improved. Sleep is okay at 5 h an evening, however patient reports sleeping through the night and waking up with energy. Advised her to incorporate nightly meditation or yoga and help with sleep, longer sleep, patient expressed understanding. IV site dry and intact, she tolerated infusion well.

#### Patient reports:

Cravings 0/10; anxiety 2/10; depression 0/10; and hours of uninterrupted sleep 5.

#### Infusion 4:

Patient reports feeling good overall. Patient reports feeling energy although also reporting sleep is recorded at 5 h last evening. She denies cravings, anxiety, depression and pain. Water intake is reported at 60 oz a day, advised patient that it is still important to increase water intake to at least half her body weight in oz. IV site dry and intact, she tolerated infusion well.

#### Patient reports:

Cravings 0/10; anxiety 0/10; depression 0/10; and hours of uninterrupted sleep 5.

#### Infusion 5:

Patient reports she feels “really good” sleep is recorded at 5 h of uninterrupted sleep, but patient reveals she has been sleeping through the night. There are issues with housemates, but she says she tries to stay out of it. Mental clarity has improved in a way where she is able to retain information whereas before she was always forgetful. Patient denies cravings, denies anxiety and depression and pain. IV site dry and intact, she tolerated infusion well.

#### Patient reports:

Cravings 0/10: anxiety 0/10: depression 0/10; and hours of uninterrupted sleep 5.

#### Infusion 6:

Patient appears to be in a mood and has good energy level. Good mood and energy are evidenced by patient’s willingness to engage in conversation and patient smiling. Patient denies cravings, anxiety, depression and pain. Patient states she feels improvements overall. Mental clarity has improved, and she is retaining information better. She reports 5 h of uninterrupted sleep and wakes up sometimes to use the restroom, advised patient to avoid liquids close to bedtimes to avoid late night bathroom use, she states she understands. IV site dry and intact, she tolerated infusion well.

#### Patient reports:

Cravings 0/10; anxiety 0/10; depression 0/10; and hours of uninterrupted sleep 5.

#### Infusion 7:

Patient appeared to be happy and confident about her recovery. Mood is evidenced by patients engaging in conversation and talking about how she is feeling better than she has ever felt before. She denies depression and pain. Patient reports her cravings levels is at a 1 out of 10 but she does not have the desire to use, patient states she does not have the desire to use but as she progresses in her recovery she thinks back to her past and starts remembering what she had gone through. Patient is observed relaxed in recliner, watching tv. IV site dry and intact, she tolerated infusion well.

#### Patient reports:

**Figure 6:**
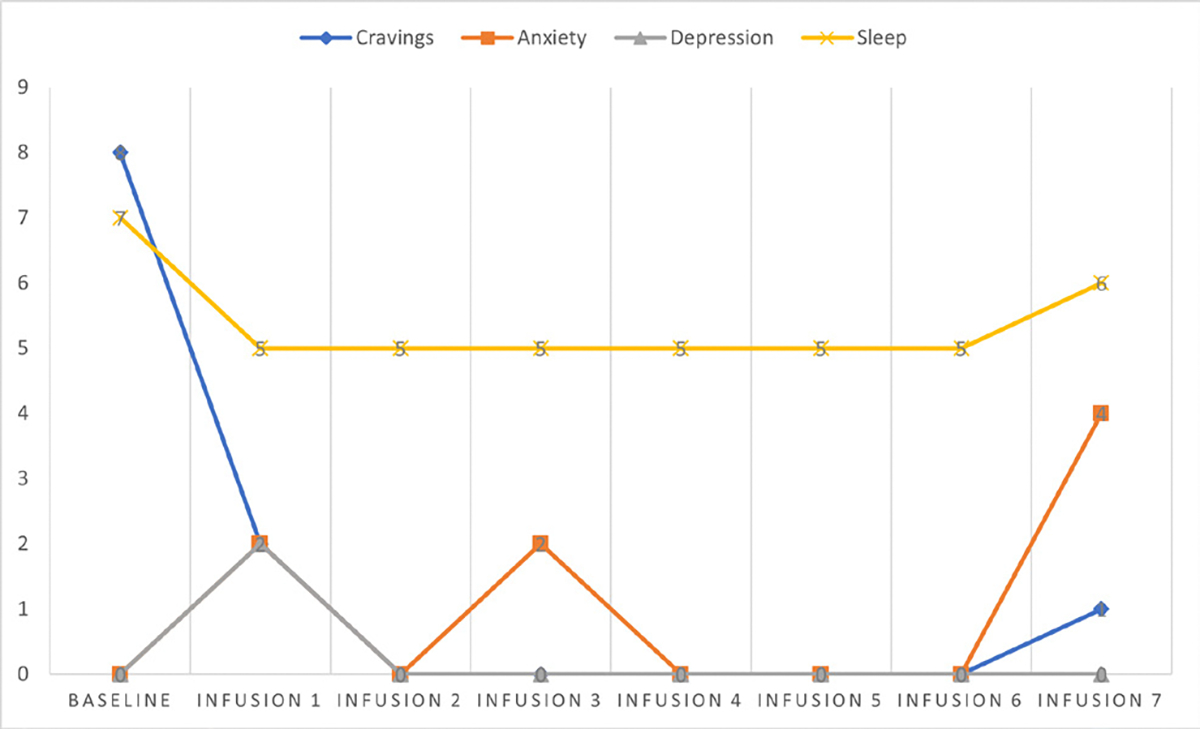
Patient 5 (Female/45/meth).

Cravings 1/10; anxiety 4/10; depression 0/10; and hours of uninterrupted sleep 6 (Table 7 and figure 6).

### Patient 6 (Female/45/meth/marijuana/duster/ETOH)

#### Consultation:

45-year-old female patient presents today for complaints of “DOC is duster, meth, marijuana, and vodka. Was drinking 1/2 pint daily as well as 2 cans a day, last use being 5/7/2020. The longest sobriety is 1.5 years in 2013. Has been to 4 treatment facilities. Denies any OD or seizures in the past. Currently I have a lot of fatigue, also with irritability. I have a hard time concentrating and with anxiety 4/10. I drink 24 oz of water daily, but I feel dehydrated because I drink sugary drinks.

#### Baseline:

Patient reports to first infusion with c/o of fatigue. Patient reports getting 3 h of sleep and feeling tired when waking up. Encouraged patients to increase water intake to help with fatigue and hydration. Patient was receptive. Patient reports high anxiety and cravings and knows she is doing the right thing but is nervous and wants to use it. Encouraged deep breathing and meditation. Patient is trying to remain positive. The patient was observed relaxing and conversing during infusion. Patient tolerated treatment well with no complaint of discomfort. The patient completed treatment and was left in stable condition.

#### Patient reports:

Cravings 10/10; anxiety 6/10; depression 4/10; and hours of uninterrupted sleep 3.

#### Infusion 1:

Patient reports having great sleep after her first infusion. Patients report immediate decrease in anxiety, and cravings. Feels less desire to use. Trying to be active and make healthy choices. IV insertion attempted x3 with no success. Encouraged patient to hydrate prior to coming for infusion treatment. Patient was receptive and expressed understanding. Patient left in stable condition.

#### Patient reports:

Cravings 2/10; anxiety 3/10; depression 0/10; and hours of uninterrupted sleep 8.

#### Infusion 2:

Patient reports she has been happy and applying herself to her work to stay sober. Patient states she did feel a difference from her first infusion waking up with more energy and more alert. Also reports a large decrease in anxiety and cravings. Patients are happy to do infusions. Patient was observed relaxing on recliner and watching TV during infusion. Patient tolerated treatment with no complaint of discomfort.

#### Patient reports:

Cravings 2/10; anxiety 2/10; depression 0/10; and hours of uninterrupted sleep 8.

#### Infusion 3:

Patient reports sleeping 8 h of uninterrupted sleep and waking up energized. Patient reports drinking 42 oz of water today and has been cutting down on high sugary drinks. Encouraged patient to continue with her healthier choices and to maintain a positive outlook on her new healthy habits without focusing on restricting or skipping meals. Patient was receptive. The patient was observed cheerful and conversing with her peers during infusion. Patient tolerated treatment well with no complaint of discomfort. The patient completed treatment and was left in stable condition.

#### Patient reports:

Cravings 0/10; anxiety 0/10; depression 0/10; and hours of uninterrupted sleep 8.

#### Infusion 4:

Patient reports feeling more energy in the mornings since she started infusions. Patient states she is really happy to be able to come to infusions. The patient was observed to be cheerful and conversing with peers during infusion. Patient tolerated treatment well with no complaint of discomfort.

#### Patient reports:

Cravings 0/10; anxiety 0/10; depression 0/10; and hours of uninterrupted sleep 8.

#### Infusion 5:

Patient reports an increase in her energy levels throughout the day since she started her infusions. Patient also reports her short-term memory has improved. The patient was observed relaxing on recliner during infusion. Patients IV site was infiltrated after infusing for 26 min. IV was attempted x3 times with no success. Encouraged patients to hydrate before coming to infusions. Patient was receptive and expressed understanding Patient assessed V/S WNL. Patient left in stable condition.

#### Patient reports:

Cravings 0/10; anxiety 0/10; depression 0/10; and hours of uninterrupted sleep 8.

#### Infusion 6:

Patient reports “improved overall well-being” patient feels happy and energetic. Patient expressed that she is most happy knowing that we were able to start her infusions because she has “difficult veins” patient likes knowing she is getting vitamins for her body. Patient reports she is trying her best to drink water. IV site dry and intact, patient tolerated infusion well.

#### Patient reports:

Cravings 0/10; anxiety 0/10; depression 0/10; and hours of uninterrupted sleep 8.

#### Infusion 7:

Patient reports “I feel full of energy” patient feels very blessed and happy to be in the position she is in currently. Patient reports she is happy that she is sober and living in a healthy household. Patient says she likes her housemates, and she likes that they keep each other accountable. Patient reports sleep has improved at 8 h and evening, patient admits she sometimes wakes up in the middle of the night and has a tough time falling back to sleep. Advised patient to try meditation and breathing techniques, client expressed understanding. IV site dry and intact, patient tolerated infusion well.

#### Patient reports:

**Figure 7:**
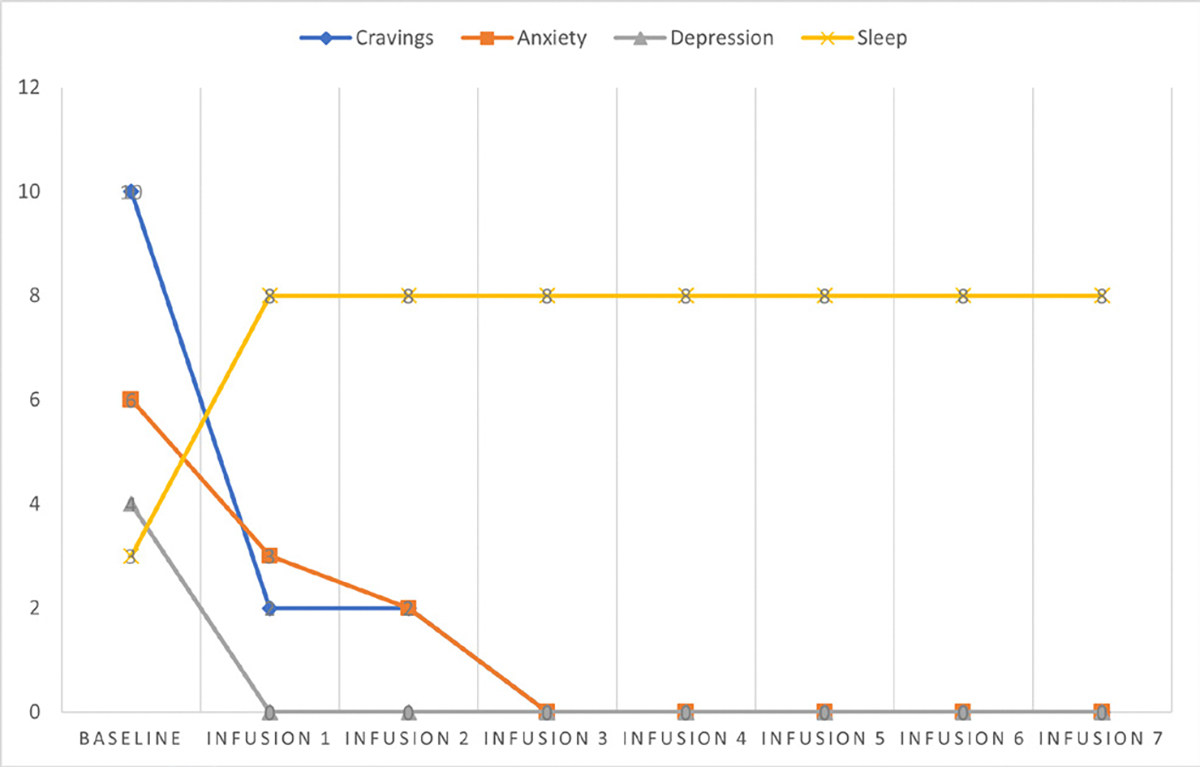
Patient 6 (Female/45/meth/marijuana/duster/ETOH).

Cravings 0/10; anxiety 0/10; depression 0/10; and hours of uninterrupted sleep 8 (Table 8 and figure 7).

### Patient 7 (Female/29/meth/ppiates/benzos)

#### Consultation:

29-year-old female patient presents today for complaints of drug use: meth, percocet’s, xanax. She takes 8 balls of meth per day. She takes 10+ percocet’s per day and she takes an occasional xanax. She last used percocet 6/8. She has never been in treatment. She has been using meth for 4 years. She has been taking percocet’s for 9 years. She last did meth 6/8 and did not sleep for 5 days after that point. Longest sobriety is 5 years 2013 – 2015. Currently I am so drained; I wake up with energy and am super tired after 20 min. I have gained a lot of weight, can’t sleep and take seroquel which helps. I have super high anxiety because treatment is new for me and makes me anxious at each new step and new house I go to.

#### Baseline:

Blood drawn from right AC from IV catheter per practitioner’s order. 3SST 2LAV 1UA obtained and sent to integritox. Client reports she has been in treatment for 1 week, client is just starting. She reports experiencing brain fog and her anxiety is high at a 5 out of 10 due to being away from her children. Client reports sleeping around 8 – 10 h currently because she is detoxing, client hopes in treatment her brain fog and fatigue improves. IV site dry and intact, client tolerated infusion well.

#### Patient reports:

Cravings 5/10; anxiety 5/10; depression 1/10; and hours of uninterrupted sleep 9.

#### Infusion 1:

Client reports feeling good, she says she did not feel sick or different after her first infusion. Client reports drinking about 32 oz of water a day, so she is thankful she is receiving vitamin infusions. Advised client to increase water intake, client expressed understanding. IV site dry and intact, client tolerated infusion well.

#### Patient reports:

Cravings 3/10; anxiety 4/10; depression 0/10; and hours of uninterrupted sleep 6.

#### Infusion 2:

Patient reports low activity level and poor appetite. Encouraged patients to try small meals during the day and to increase fluid intake. Educated on the importance of nutrition. Patient was receptive. The patient was observed relaxing on recliner during infusion. patient tolerated treatment well with no complaint of discomfort.

#### Patient reports:

Cravings 0/10; anxiety 3/10; depression 0/10; and hours of uninterrupted sleep 5.

#### Infusion 3:

“Things aren’t foggy, improved cognitive function” client states she is having a bad day, nothing specific happened but she isn’t feeling the best. Client did say she feels better after infusions. Client reports sleep has improved, and she is having an easier time remembering information, this is helping her during groups and treatment, client feels optimistic about her sobriety/recovery. IV site dry an intact, client tolerated infusion well.

#### Patient reports:

Cravings 0/10; anxiety 0/10; depression 0/10; and hours of uninterrupted sleep 6.

#### Practitioner FU:

Client presents to clinic for follow up. This is client’s 4^th^ infusion states she feels a minor increase in energy, and less anxiety. States she has not noticed any difference in sleep at this time. “My moods are like a roller coaster at this time.”

#### Infusion 4:

Client reports “improved overall mood” client states she is feeling more positive overall, and it has been amazing with her sobriety. The client expressed that she harbors a lot of anger toward her mother, and she tries to get over it. Advised client to discuss with case manager and therapist, advised client that the anger she feels could hinder her journey toward sobriety, client expressed understanding. IV site dry and intact, client tolerated infusion well.

#### Patient reports:

Cravings 2/10; anxiety 0/10; depression 0/10; and hours of uninterrupted sleep 6.

#### Infusion 5:

Client reports feeling very good because she is focusing on herself and her sobriety, client is now “single” she just got out of a toxic relationship because client reports she sees the importance of putting her sobriety first and not stress about “guy problems” commended client for wanting to focus on herself and advised client to discuss any issues or problems she might have with her therapist and case manager, client expressed understanding. IV site dry and intact, client tolerated infusion well.

#### Patient reports:

Cravings 0/10; Anxiety 0/10; Depression 0/10; and hours of uninterrupted sleep 8.

#### Infusion 6:

Client reports she feels “meh” today, client is a little sad her roommate will be moving to a different house today. Advised client to maintain focus on herself and her sobriety. Client states she is feeling more positive overall, and it has been amazing with her sobriety. The client expressed that she harbors a lot of anger toward her mother, and she tries to get over it. Advised client to discuss with case manager and therapist, advised client that the anger she feels could hinder her journey toward sobriety, client expressed understanding. IV site dry and intact, client tolerated infusion well.

#### Patient reports:

Cravings 0/10; anxiety 3/10; depression 0/10; and hours of uninterrupted sleep 8.

#### Infusion 7:

Client states she is feeling more positive overall, and it has been amazing with her sobriety. Client expressed that she still is harboring a lot of anger toward her mother, and she tries to get over it. Advised client to discuss with case manager and therapist, advised client that the anger she feels could hinder her journey toward sobriety, client expressed understanding. IV site dry and intact, client tolerated infusion well.

#### Patient reports:

**Figure 8:**
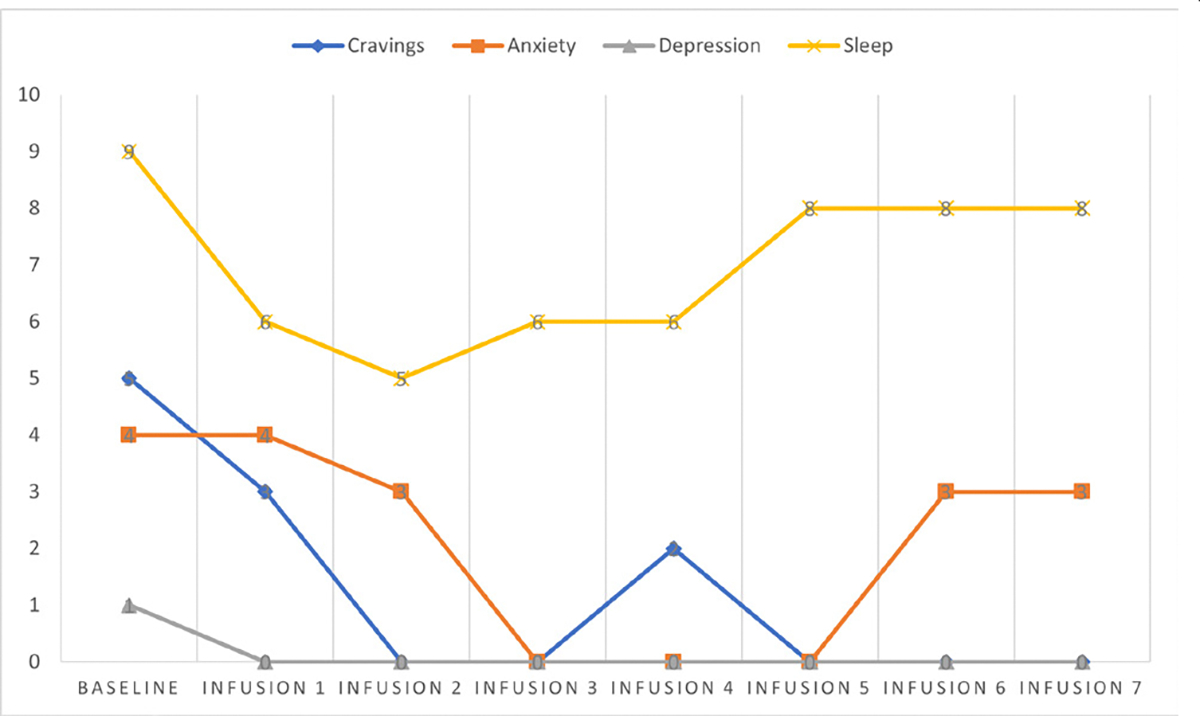
Patient 7 (Female/29/meth/opiates/benzos).

Cravings 0/10; anxiety 3/10; depression 0/10; and hours of uninterrupted sleep 8 (Table 9 and figure 8).

### Patient 8 (Female/28/suboxone)

#### Consultation:

28-year-old female patient presents today for complaints of “DOC is suboxone 8 – 24 mg daily for 5 years, and red bulls 5 – 6 daily. Last use was 12 days ago. The longest sobriety is 32 days. This is the client’s first treatment facility. “I have severe anxiety and irritability around people. I am nauseated and can’t drink water. I’ve been trying to concentrate but hard to. I have not been eating healthy and enough food. I am also restless. I’ve also been sneezing nonstop.

#### Baseline:

Patient reports to first infusions with complaints of RLS that affects her sleep. Patient reports only getting 3 h of sleep and waking up feeling fatigued. Patient reports having poor appetites only snacking 1 – 3 times a day. Encouraged patients to try small frequent meals and educated them on the importance of nutrition and hydration. Patient was receptive and expressed understanding. Patient was observed to be relaxing during infusion. Patient tolerated treatment well with no complaint of discomfort.

#### Patient reports:

Cravings 8/10; anxiety 2/10; depression 0/10; and hours of uninterrupted sleep 3.

#### Infusion 1:

Blood drawn from left AC from IV catheter per practitioner’s order. 4SST 1LAV 1UA obtained and sent to Integritox. Patient reports feeling great and noticing a huge difference with her first infusion. Patient reports her RLS stopped and her sleep as improved. Patient reports getting 8 h of sleep the first night after her infusion and thereafter. Patient reports feeling more energy and is able to concentrate better. Patient is excited to continue with her treatment. Patient reports struggling with her appetite and water intake. Educated patients on the importance of good nutrition and hydration. Patient was receptive and expressed understanding. Patient was observed to be cheerful and conversing with staff during infusion. Patient tolerated treatment well with no complaint of discomfort.

#### Patient reports:

Cravings 5/10; anxiety 2/10; depression 0/10; and hours of uninterrupted sleep 8.

#### Infusion 2:

Patient self-rates anxiety, depression, and cravings 0/10. Patient denies any pain. Patient reports she is happy and excited to continue infusions. Patient reports not being able to sleep and feel rested since she got sober and has been sleeping a full 8 h since she started the infusions. Patient reports increasing her water intake to 64 oz and her appetite has been getting better. The patient was observed relaxing with her eyes closed during infusion. Patient tolerated treatment well with no complaint of discomfort.

#### Patient reports:

Cravings 0/10; anxiety 0/10; depression 0/10; and hours of uninterrupted sleep 8.

#### Infusion 3:

Patient self-rates anxiety, cravings, depression 0/10. Patient denies pain. Patient states, “I feel great overall, my sleep is great, and my appetite has gotten a lot better” patient reports increasing her water intake but sometimes forgets to drink water. Encouraged patient to maintain her positive attitude and continue with healthy habits. Patient was receptive. The patient was observed cheerful and conversing with peers during infusion. Patient tolerated treatment well with no complaint of discomfort.

#### Patient reports:

Cravings 0/10; anxiety 0/10; depression 0/10; and hours of uninterrupted sleep 8.

#### Practitioner FU:

Client presents to clinic for NAD follow up. Currently I am now sleeping 8 h uninterrupted without medication, where before infusions I was sleeping 2 h at a time interrupted and 4 h nightly. I have more energy and most importantly my restless leg has subsided. Stop bentyl for my stomach cramps because I feel better. I love this stuff and has changed my life.

#### Infusion 4:

Patient self-rates cravings 0/10, depression and anxiety 0/10. Patient reports she has pain 4/10 due to her getting pleurisy. Patient recently wanted to quit smoking and pick up vaping. Patient states she has been hurting for a few days now. Encouraged patient to rest and to follow any instructions that were given to her by diagnosing doctor, patient was receptive. Patient reports her appetite has improved but still struggles with drinking water. Patient reports she her sleep has been great, getting about 7 – 8 h a night. The patient was observed cheerful and conversing with peers during infusion. Patient tolerated treatment well with no complaint of discomfort.

#### Patient reports:

Cravings 0/10; anxiety 0/10; depression 0/10; and hours of uninterrupted sleep 7.

#### Infusion 5:

Client reports she feels good, and sleep has improved overall. Improved sleep has in turn increased the client’s energy level. Client denies cravings and pain. The client admitted she is experiencing anxiety and depression, client self-rated her anxiety and pain at a 6 out of 10, advised client to practice meditation and breathing exercises and to discuss with treatment team. IV site dry and intact, client tolerated infusion well.

#### Patient reports:

Cravings 0/10; anxiety 6/10; depression 6/10; and hours of uninterrupted sleep 8.

#### Infusion 6:

Patient reports feeling happy today. Patient admits she feels some anxiety, she talks to her peers and treatment team and tries to work through it but her anxiety, which is self-rated at a 2 out of 10 is due to “future tripping” where client worries about events that have not happened yet and does not know what she would do without her support team, advise patient to discuss with her healthcare provider and her therapist, patient expressed agreement. IV site dry and intact, patient tolerated infusion well.

#### Patient reports:

Cravings 0/10; anxiety 2/10; depression 3/10; and hours of uninterrupted sleep 8.

#### Infusion 7:

Patient states she is feeling an increase in energy levels. Sleep is reported at 7 h of sleep. Patient said she was put on blood thinners by her physician the beginning of July, she stopped recently because she started her menstrual cycle. Patient denies anxiety and pain. Patient self-rated her cravings at a 0 out of 10 and states she gets through it by talking to her peers and therapist. The patient is currently working on the 3^rd^ step of her step program, and she is eager to complete it. IV site dry and intact, patient tolerated infusion well.

#### Patient reports:

**Figure 9:**
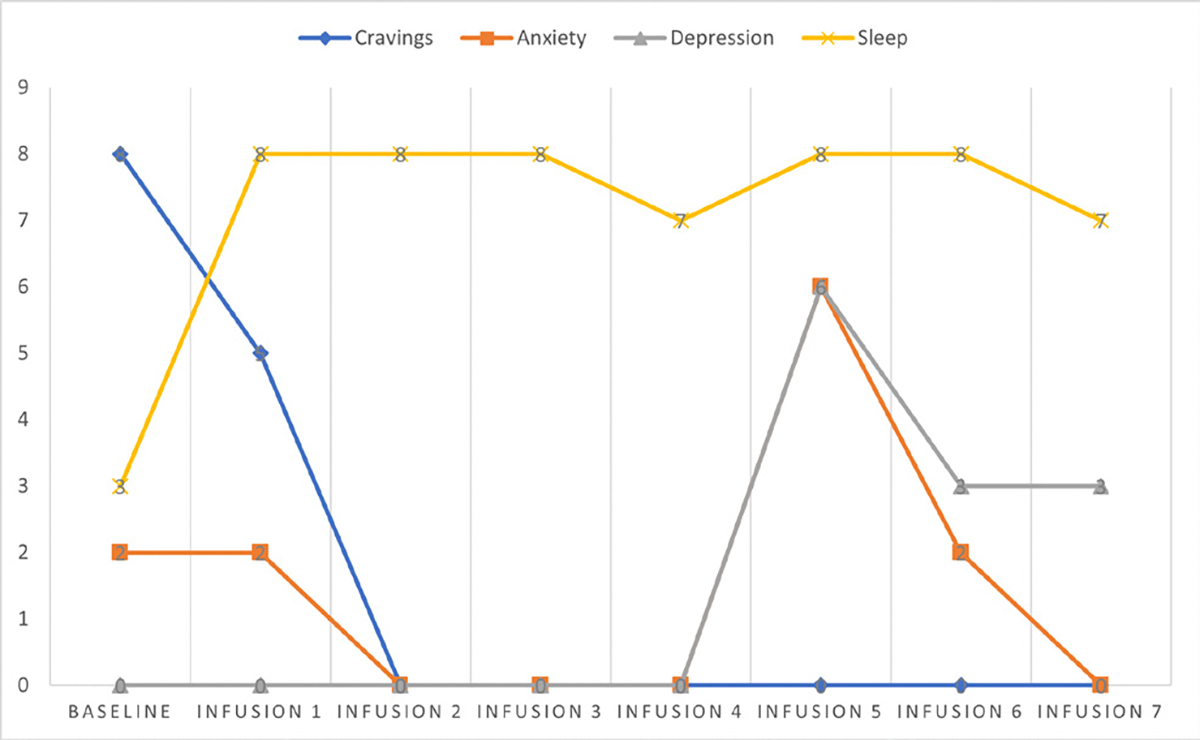
Patient 8 (Female/28/suboxone).

Cravings 0/10; anxiety 0/10; depression 3/10; and hours of uninterrupted sleep 7 (Table 10 and figure 9).

### Patient 9 (Female/38/meth)

#### Consultation:

38-year-old female patient presents today for complaints of DOC is meth via smoking and IV, last use was 6/5. Has been using it for 5 years. This is my client’s 1st treatment facility. 2 OD on pills and no seizures. Currently denies any chest pain, no SOB, no abdominal pain, no n/v/d. Takes Gabapentin 800 mg TID and Buspar 10 mg TID. Currently I am always living in a state of fatigue. I have anxiety right now and I’m irritable. I didn’t drink any water and feel dehydrated. I want to get my mind right.

#### Baseline:

Client is here for her first infusion; client reports her craving level for meth and cannabis is about 6 out of 10. Advised client to discuss with case manager and refocus her energy and thoughts to treatment, her sobriety and meditation. Client expressed understanding. Client states her current anxiety level is about a 7 out of 10, client is nervous she will relapse due to her high craving levels, advised client to focus on her program and reach out whenever she needs help, let client know she has many outlets she can reach out like her case manager, medical staff as well as sponsor. Client states she wants to start her life, she doesn’t want to need drugs anymore. IV site dry and intact, client tolerated infusion well.

#### Patient reports:

Cravings 6/10; anxiety 7/10; depression 0/10; and hours of uninterrupted sleep 10.

#### Infusion 1:

Client states she feels okay, she thinks the infusion helped her with sleep last time. Client reports sleeping through the night and feeling very tired the day after her infusion. Client reports waking up feeling rested. Client states she has increased her water intake, currently client is at around 180 oz a day of water, client drink caffeine sometimes. IV site dry and intact, client tolerated infusion well.

#### Patient reports:

Cravings 0/10; anxiety 2/10; depression 1/10; and hours of uninterrupted sleep 8.

#### Infusion 2:

Client reports feeling “pretty good” after her last infusion. The client states the evening after her infusion she slept “very well”, and she woke up feeling rested and energized. Client claims she normally has broken sleep, but it seems to be improving. Client drinks about 24 oz of water a day, advised client to increase water intake, client expressed understanding. IV site dry and intact, client tolerated infusion well.

#### Patient reports:

Cravings 0/10; anxiety 2/10; depression 2/10; and hours of uninterrupted sleep 7.

#### Infusion 3:

Patient reports feeling the difference in her energy since she started the infusion. Patient reports better mood and minimal anxiety. Patient was observed to be cheerful and conversing with her peers during infusion. Patient tolerated treatment well with no complaint of discomfort.

#### Patient reports:

Cravings 0/10; anxiety 3/10; depression 0/10; and hours of uninterrupted sleep 8.

#### Practitioner FU:

Client presents to clinic for follow up. This is infusion 5 for a client. “I feel like I have less anxiety 3/10 where I used to be 8/10, my mind is clearer and able to concentrate during my sobriety and I sleep good. I still have a really high appetite. I notice when I miss my infusion times, I feel blah.”

#### Infusion 4:

“I feel good after the treatments” client reports feeling is evidenced by client sleeping well and feeling energetic and rested after sleep. Client reports her mental clarity has improved and her thought processes are better. Client reports she believes her memory has gotten better because she seems to be retaining information better. Today a client went to the dentist this morning to check a cracked tooth she had; she still feels a little numb. IV site dry and intact, client tolerated infusion well.

#### Patient reports:

Cravings 0/10; anxiety 0/10; depression 0/10; and hours of uninterrupted sleep 8.

#### Infusion 5:

Client reports “mind is clearing up, anxiety is lowered, and I feel good overall” client states she feels happy because her improvements are noticeable. Client states she is able to remember and recall information a lot quicker than previously. Sleep is good at 8 h and evening, client sometimes wakes up in the middle of the night and has difficulty falling back to sleep, advised client to practice grounding and breathing techniques and meditation to calm herself back to sleep, client expressed understanding. IV site dry and intact, client tolerated infusion well.

#### Patient reports:

Cravings 0/10; anxiety 0/10; depression 0/10; and hours of uninterrupted sleep 8.

#### Infusion 6:

Client reports feeling good overall, client tolerates infusions well and enjoys coming in for infusions. Client states, “sleeps is getting better, lowered anxiety levels” client admits that her anxiety was very bad initially and since treatment and infusion therapy, client reports feeling less anxiety and she believes she is handling different situations better. IV site dry and intact, client tolerated infusion well.

#### Patient reports:

Cravings 0/10; anxiety 0/10; depression 0/10; and hours of uninterrupted sleep 8.

#### Infusion 7:

Client reports feeling good overall, client tolerates infusions well. Client states, “sleep is getting better, lowered anxiety levels” since treatment and infusion therapy, client reports feeling less anxiety. IV site dry and intact, client tolerated infusion well.

#### Patient reports:

**Figure 10:**
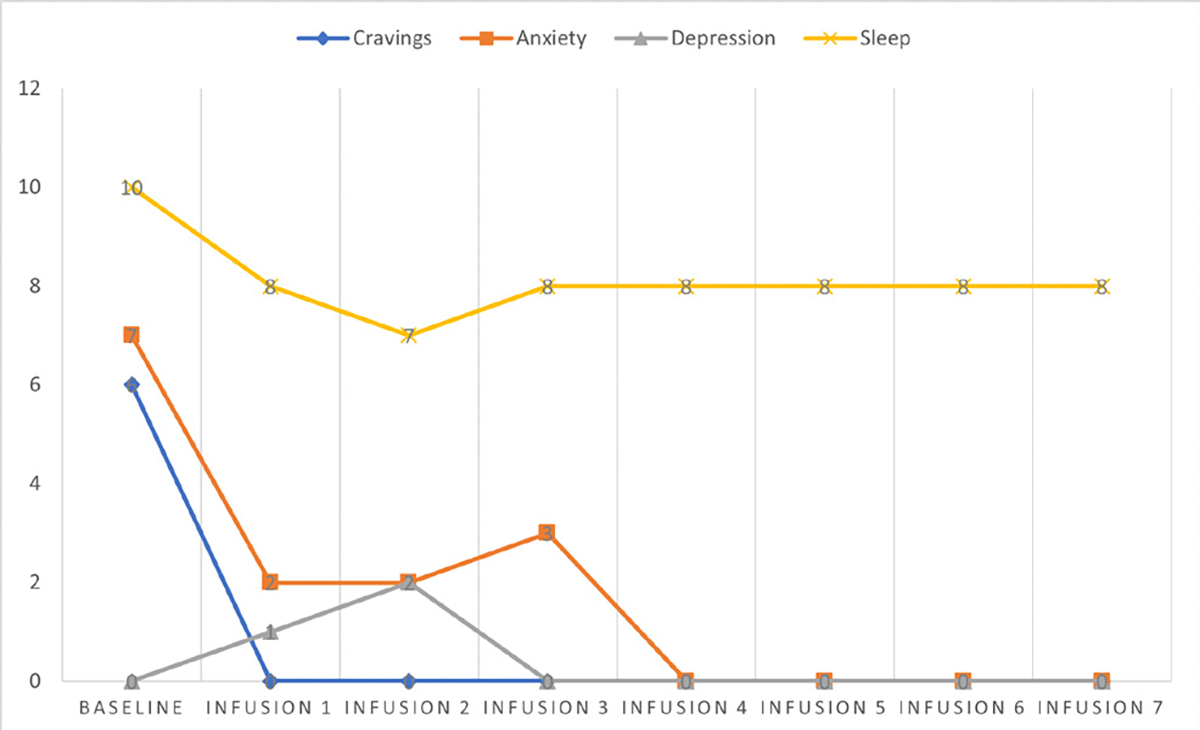
Patient 9 (Female/38/meth).

Cravings 0/10; anxiety 0/10; depression 0/10; and hours of uninterrupted sleep 8 (Table 11 and figure 10).

### Patient 10 (Female/32/meth/opiates/benzos)

#### Consultation:

32-year-old female with a history of polysubstance abuse including methamphetamines, opiates and benzodiazepines. Presents today for initial consultation for the start of infusion therapy. Current complaints consist of anxiety, decreased energy, insomnia. 19-year history of substance abuse including methamphetamines, opiates and benzodiazepines. Last stated use 10/06/20. Reports 8 – 9 months sobriety with pregnancies x 3.1 treatment center.

#### Baseline:

Patient had blood drawn from left ac via IV catheter. 4SST 1LAV 1UA obtained and sent to Integritox. HCG urine test Negative. Patient self-rates cravings 4/10, anxiety 6/10 (situational), and depression 1/10. Patient denies myalgia. Patient reports drinking no water, no GI complaints, moderate activity level, good appetite. Encouraged patients to increase water intake and educated on the importance of proper hydration. Patient was receptive. Patient reports feeling fatigued throughout the day and admitted to being anemic and having had a recent blood transfusion due to the severity. The patient has been sobering for almost a month and is happy to be taking care of herself for her children. Patient was observed relaxing and conversing with peers during infusion. Patient tolerated treatment well with no complaint of discomfort.

#### Patient reports:

Cravings 4/10; anxiety 6/10; depression 1/10; and hours of uninterrupted sleep 8.

#### Infusion 1:

Patient self-rates cravings1/10, anxiety 2/10, depression 1/10. Patient denies myalgia. Patient reports good appetite, water intake 8 oz, no GI complaints, sleep 8 h, moderate activity level. Patient reports to have slept really well last night after infusion and waking up less tired. Encouraged patients to increase their water intake. Patient states “ I forget to drink water, and it doesn’t taste good”. Encouraged patient to try water flavoring to help with taste patient was receptive. The patient was observed relaxing and watching TV during infusion. Patient tolerated treatment well with no complaint of discomfort.

#### Patient reports:

Cravings 1/10; anxiety 2/10; depression 1/10; and hours of uninterrupted sleep 8.

#### Infusion 2:

Patient self-rates her cravings, anxiety and depression at 1/10, pain at 4/10 patient states she slept wrong and has had pain for two days now. Patient has no fever, patient states she eats 3 meals a day and drinks approx. 10 oz of water, educated on importance of staying hydrated patient was receptive. Patient denies BM issues and states having a moderate activity level. Patient tolerated treatment well with no pain or discomfort.

#### Patient reports:

Cravings 1/10; anxiety 1/10; depression 1/10; and hours of uninterrupted sleep 4.

#### Infusion 3:

Patient self-rates cravings, anxiety and depression 0/10. Patient denies myalgia. Patient reports 6 h of sleep, no GI complaints, moderate activity level, good appetite, water intake 8 oz. Encouraged patients to increase water intake and reiterated the importance of proper hydration. Encouraged patients to try alternative flavors for water to help increase intake. Patient was receptive. The patient was observed relaxing and talking on phone during infusion. Patient tolerated treatment well with no complaint of discomfort.

#### Patient reports:

Cravings 0/10; anxiety 0/10; depression 0/10; and hours of uninterrupted sleep 6.

#### Infusion 4:

Patient self-rates cravings and depression 0/10, and anxiety 3/10 situational. Patient denies myalgia. Patient reports 6 h of sleep, no GI complaints, good appetite, 3 meals + snacks, moderate activity level, water intake 8 oz. Encouraged patients to increase their water intake. Patient stated she will try to drink more water with the advice given to her last visit. Patient reports feeling a difference with her anxiety since she started infusions. The patient was observed relaxing during infusion. Patient tolerated treatment well with no complaint of discomfort.

#### Patient reports:

Cravings 0/10; anxiety 3/10; depression 0/10; and hours of uninterrupted sleep 6.

#### Infusion 5:

Patient report sleep is poor at 4 – 5 h an evening. Advised her to practice meditation and breathing techniques to promote better sleep. Water intake is poor at 4 – 5 cups a day, advised her to increase water intake and educated her on importance of water intake. Patient expressed understanding. IV site dry and intact, she tolerated infusion well.

#### Patient reports:

Cravings 4/10; anxiety 3/10; depression 3/10; and hours of uninterrupted sleep 5.

#### Infusion 6:

Patient reports sleep is okay, wakes up feeling tired sometimes because she wakes up to use the restroom. Advised patients to avoid food and liquids close to bedtime. Patient claims she does not drink any water, “0 oz a day” strongly advised patient to increase water intake and educated her on the importance of hydration. She expressed understanding. IV site dry and intact, she tolerated infusion well.

#### Patient reports:

Cravings 0/10; anxiety 0/10; depression 0/10; and hours of uninterrupted sleep 6.

#### Infusion 7:

Patient denies cravings, anxiety, depression and pain. She reports last bowel movement was 1/13/2021. Water intake is poor at 8 oz. Educated the patient on the importance of hydration and advised her to increase water intake. Patient expressed understanding. Patient is observed relaxed in recliner watching TV. IV site dry and intact, she tolerated infusion well.

#### Patient reports:

**Figure 11:**
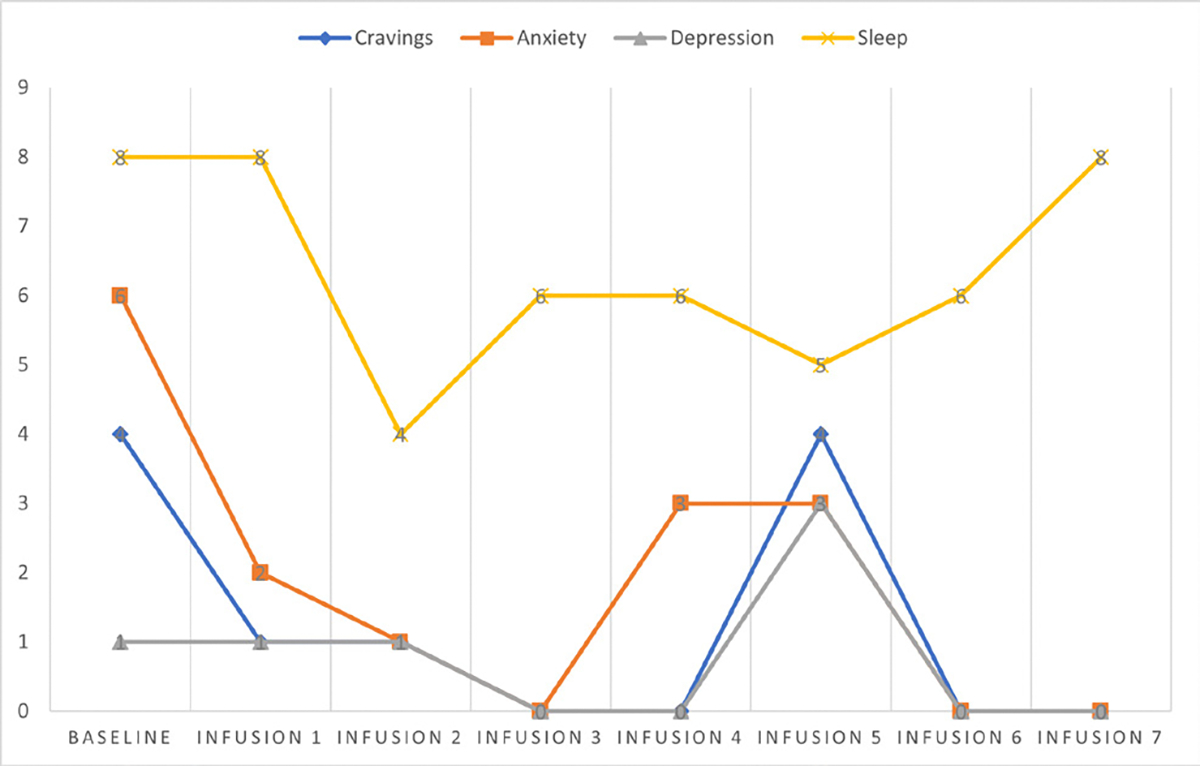
Patient 10 (Female/32/meth/opiates/benzos).

Cravings 0/10; anxiety 0/10; depression 0/10; and hours of uninterrupted sleep 8 (Table 12 and figure 11).

### Patient 11 (Female/34/meth/heroin/ETOH)

#### Consultation:

34-year-old female patient presents today for complaints of DOC is alcohol and meth and heroin via eating, smoking and snorting 1 – 2 g daily. Was drinking 1/5 of whiskey daily. Last use was 8/28, longest sobriety is 4 years. This is the client’s 1st treatment facility. 1 OD and 1 seizure in the past. Currently “I have a hard time sleeping and medications are helping. I have trouble focusing on one thing. My anxiety is usually 10/10. I also get nausea every day.”

#### Baseline:

Blood drawn from R wrist via IV catheter per practitioners’ orders. 4 SST, 2 LAV, 1 UA, NEGATIVE HCG, sent to INTEGRITOX. Patient self-rates her cravings at 5/10, anxiety at 4/10 and pain at 4/10 on her back and states it is chronic pain. Patient states she snacks throughout the day and drinks approx. 1 gallon of water daily. Patient denies BM issues and states having a moderate activity level. Patients tolerated treatment well with no pain or discomfort.

#### Patient reports:

Cravings 5/10; anxiety 4/10; depression 0/10; and hours of uninterrupted sleep 4.

#### Infusion 1:

Patient self-rates her cravings today at 2/10, anxiety at 3/10 (situational), and pain at 2/10 on her back. Patient states she has dislocated discs and pain is chronic, she did mention that her back does not hurt as much as yesterday. Patient states she snacks throughout the day and drinks approx. 120 oz of water daily. Patient denies BM issues and states having a moderate to high activity level. Patients tolerated treatment well with no pain or discomfort.

#### Patient reports:

Cravings 2/10; anxiety 3/10; depression 0/10; and hours of uninterrupted sleep 8.

#### Infusion 2:

Patient self-rates anxiety 7/10 (situational/emotional), cravings 4 – 5/10, depression 2/10. Patient c/o of lower back pain due to fused disk. Patient reports sleeping 8 h, moderate activity level, no GI problems, drinks about 64 oz of water daily. The patient was observed relaxing watching TV during infusion. Patient tolerated treatment well with no complaint of discomfort.

#### Patient reports:

Cravings 4/10; anxiety 7/10; depression 2/10; and hours of uninterrupted sleep 8.

#### Infusion 3:

Patient self-rates anxiety and depression 3/10, no cravings. Patient c/o myalgia r/t her fused disks in her back. Patient reports the anti-inflammatory helped with the pain. Patient reports poor appetite encouraged patient to try eating some of her favorite foods in small portions or try ensuring. Patient was receptive. Patient reports drinking one gallon of water today. The patient was observed keeping to herself and on her phone during infusion. Patient tolerated treatment well with no complaint of discomfort.

#### Patient reports:

Cravings 0/10; anxiety 3/10; depression 3/10; and hours of uninterrupted sleep 8.

#### Infusion 4:

Patient self-rates cravings 3/10, anxiety 5/10, and depression 2/10. Patient denies myalgia. Patient reports have been feeling irritable throughout the day. The patient has had recent changes to her medication regimen. Recently, started naltrexone and decreased her depakote which could be related to her mood. Advised patient to communicate with her prescribing doctor regarding any other symptoms that may arise. Patient was receptive. Encouraged patients to practice meditation or breathing exercises to help ease anxiety. Patient was observed relaxing and conversing with peers during infusion. Patient tolerated treatment well with no complaint of discomfort.

#### Patient reports:

Cravings 3/10; anxiety 5/10; depression 2/10; and hours of uninterrupted sleep 9.

#### Infusion 5:

Patient denies cravings, self-rates anxiety 3/10, and depression 0/10. Patient c/o of chronic back and neck pain. Patient states the ani-inflammatory support has been helping her pain. Patient reports drinking 1 gallon of water, sleeping 3 h, no GI complaints, moderate activity level and poor appetite. Encouraged patient to try snack and shakes like ensure when she doesn’t feel hungry. Patient was receptive. Patient c/o of restless sleep and feeling fatigued throughout the day. Encouraged patients to practice meditation or light exercise like walking in the evening to help with her anxiety and sleep. Patient was receptive. Patient was observed relaxing on recliner watching TV during infusion. Patient tolerated treatment well with no complaint of discomfort.

#### Patient reports:

Cravings 0/10; anxiety 3/10; depression 0/10; and hours of uninterrupted sleep 3.

#### Infusion 6:

Patient self-rates cravings and anxiety 4/10, depression 1/10. Patient c/o back pain due to untreated herniated disks 4/10. Patient reports drinking 60 oz of water daily, no GI complaints, moderate appetite, sleep 10 h. Patient disclosed to that she was advised to cut food and only drink protein shakes and cheese. Patients were encouraged to practice a well-balanced diet without restricting and focus on portion control and quality of meals. Patient was receptive. The patient was observed relaxing and conversing with staff during infusion. Patient tolerated treatment well with no complaint of discomfort.

#### Patient reports:

Cravings 4/10; anxiety 4/10; depression 1/10; and hours of uninterrupted sleep 10.

#### Infusion 7:

Patient presents to the office for her infusion and anti-inflammatory support. Patient self-rates her cravings today at 5/10, anxiety at 4/10 (situational and emotional), depression at 3/10 (situational and emotional), and pain at 3/10, herniated discs that still has not been treated. Patient states having a poor appetite, she only eats 1 meal a day and drinks approx. 1 gallon of water daily. Patient denies BM issues at the moment and states having a moderate activity level. Patients tolerated treatment well with no pain or discomfort.

#### Patient reports:

**Figure 12:**
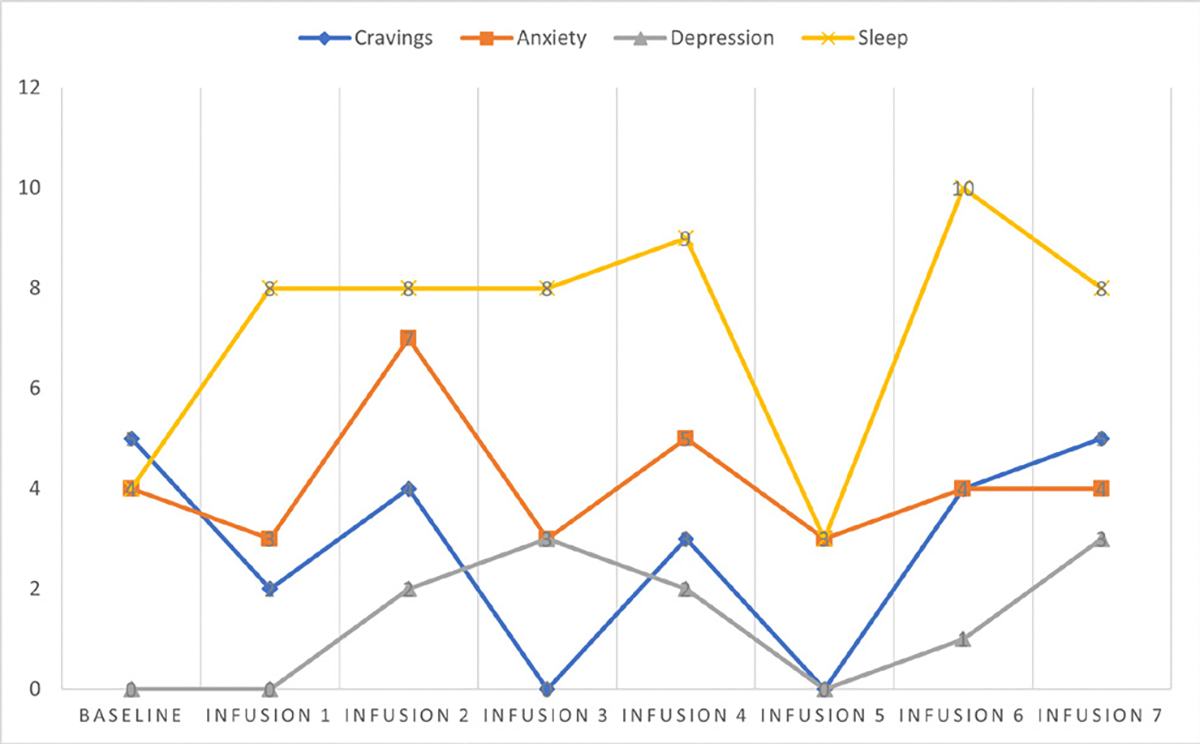
Patient 11 (Female/34/meth/heroin/ETOH).

Cravings 5/10; anxiety 4/10; depression 3/10; and hours of uninterrupted sleep 8 (Table 13 and figure 12).

### Patient 12 (Female/32/meth, ETOH, heroin)

#### Consultation:

32-year-old female patient presents today for complaints of “DOC is meth via IV, last use 30 days ago and alcohol (last use 11 months ago). Started having alcohol at age 5 and progressed to black tar heroin by age 13. The longest sobriety is currently, and this is clients first treatment facility. States 1 OD in 2018 and no hx seizures. Currently “I have 5/10 anxiety, I have insomnia and can’t sleep at night, and I’m irritable everyday due to my cirrhosis and the pain I have in my RLQ.”

#### Baseline:

Blood draw from Right dorsal hand via IV catheter per practitioner’s order. Obtained 4SST 1LAV 1UA and sent it to Integritox. Patient self-rates anxiety 8/10 has worsened since she stopped using drugs. self-rates cravings 6/10, depression 0/10. Patient c/o myalgia to her left ankle due to old Fx, noticeable edema no pitting. Patient reports she never took care of her injury and walked too soon on it. Patient reports her appetite has improved since she got sober. Encouraged patients to increase food intake and snacks. Patient reports drinking 8 – 10 oz of water. Encouraged patients to increase water intake and educated on the importance of proper hydration. Patient was observed relaxing and conversing with peers during infusion. Patient tolerated treatment well with no complaint of discomfort.

#### Patient reports:

Cravings 6/10; anxiety 8/10; depression 0/10; and hours of uninterrupted sleep 6.

#### Infusion 1:

Patient comes into the office today for her 2^nd^ treatment. Patient stated she hasn’t felt much of a difference since the 1^st^ treatment. Patient self-rates have no cravings, anxiety at a level 3 – 4 out of 10 (situational), no depression or pain. Recommended of natural ways to help with anxiety such yoga or meditation. Patient states having a good appetite by eating 3 meals a day and drinking 120 oz of water daily. Patient states have no BM issues. Patient states have a moderate activity level. Patients tolerated treatment well with no pain or discomfort.

#### Patient reports:

Cravings 6/10; anxiety 8/10; depression 0/10; and hours of uninterrupted sleep 6.

#### Infusion 2:

Patient self-rates cravings 0/10, depression 3/10, and anxiety 3/10. Patient c/o LLE myalgia due to an old injury 15 years ago. Patient reports good appetite and staying active. Patient reports drinking 100 oz of water and sleeping 10 h. Patient reports feeling anxious due to her mother still using it. Encouraged patient to stay positive and to focus on her sobriety. Encouraged patients to practice meditation and breathing exercises to help relax. Patient was observed to be cheerful and conversing with peers. Patient tolerated treatment well with no complaint of discomfort.

#### Patient reports:

Cravings 0/10; anxiety 3/10; depression 0/10; and hours of uninterrupted sleep 10.

#### Infusion 3:

Blood drawn from right dorsal hand via IV catheter per practitioner’s order. 4SST 1LAV obtained and sent to Integritox. Patient self-rates cravings 0/10, anxiety 4/10, depression 0/10. Patient c/o mild ankle pain from an old injury. Patient reports good appetite, drinks 64 oz of water and slept 2 h. Patient reports she has been having a difficult time falling asleep. Encouraged patients to practice breathing exercises or meditation in the evening to help relax before bed. Patient was receptive. The patient has been sick for a few days now and she feels that might be part of the reason she hasn’t been sleeping. The patient was observed relaxing and watching TV during infusion. Patient tolerated treatment well with no complaint of discomfort.

#### Patient reports:

Cravings 0/10; anxiety 4/10; depression 0/10; and hours of uninterrupted sleep 2.

#### Practitioner FU:

Client presents to clinic for follow up on NAD. Currently on infusion 4. “1. I have decreased anxiety now 4/10 and before was 10/10. 2. my concentration has already improved and I’m more positive and I feel like a million bucks.”

#### Infusion 4:

Patient comes into the office in great spirits. Patient self-rates her anxiety at a level 6 out of 10 (situational), pain at a level 5 out of 10. Patient states pain is on her right kneecap and pain is continuous. Will be admin 2.2 ml of Traumeel inside Myers, per practitioners’ orders. Recommended natural ways to help with anxiety such as yoga or meditation. Patient states having a good appetite by eating 3 meals a day and drinking approx. 120 – 150 oz of water daily. Patient states having no BM issues and having a moderate activity level. Patient states she is off all her medications. Patients tolerated treatment well with no pain or discomfort.

#### Patient reports:

Cravings 0/10; anxiety 6/10; depression 0/10; and hours of uninterrupted sleep 8.

#### Infusion 5:

Patient reports feeling in a good mood today, she is happy it is Friday and states she is thankful she does not feel sick anymore. Patient denies cravings. Reports anxiety at an 8 – 9 and depression at a 3 – 4, advised patient to discuss with case manager and to help manage feelings through meditation and breathing exercises. Patient admits she wants to have a baby, “I want to do it right this time” advised patient to focus on herself and her sobriety first. Patient agreed, IV site dry and intact, patient tolerated infusion well.

#### Patient reports:

Cravings 0/10; anxiety 8/10; depression 3/10; and hours of uninterrupted sleep 8.

#### Infusion 6:

Patient comes into the office in a good mood but with HBP and c/o pain on legs. Patient self-rates her cravings, anxiety, and depression at a level 0 out of 10. Patient self-rates her pain at a level 7 out of 10 on both of her legs states tingling and soreness sensation. Patient states having a good appetite by eating 3 meals a day and drinking approx. 120 oz of water daily. Patient states having no BM issues and having a high activity level. Patients tolerated treatment well with no pain or discomfort.

#### Patient reports:

Cravings 0/10; anxiety 0/10; depression 0/10; and hours of uninterrupted sleep 10.

#### Infusion 7:

Patient reports feeling okay, she has not been feeling the best and her appetite has been poor. Patient states, “I know I’m dehydrated” patient says she knows she needs to increase her water intake, but she says it is difficult, advise her to carry a water bottle so she can physically see she needs to drink water and how much she drank, she expressed understanding. IV site dry and intact, patient tolerated infusion well.

#### Patient reports:

**Figure 13:**
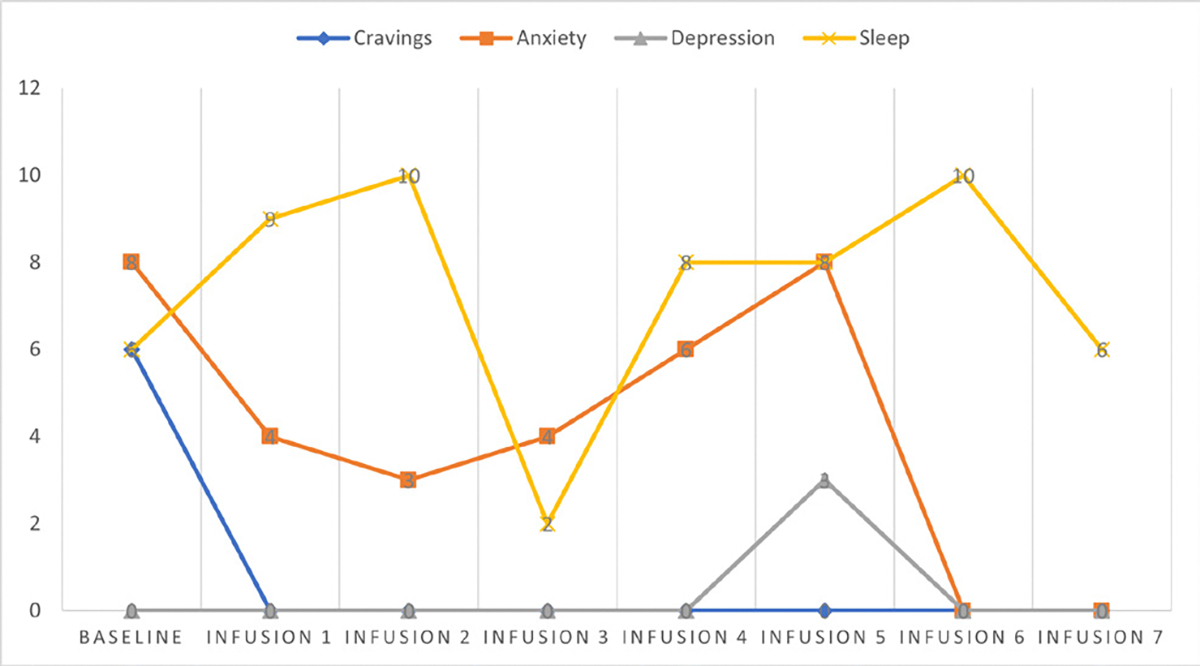
Patient 12 (Female/32/meth, ETOH, heroin).

Cravings 0/10; anxiety 0/10; depression 0/10; and hours of uninterrupted sleep 6 (Table 14 and figure 13).

### Patient 13 (Female/21/meth/benzos)

#### Consultation:

21-year-old female patient presents today for complaints of DOC is meth and xanax using 1/2 bar daily, last use was 1.5 weeks ago, longest sobriety is 28 days and has been to 1 treatment facilities. Since she stopped using, I have experienced every symptom you can think of, now that I’m not using, I feel anxious 9/10, irritable 9/10, can’t sleep which is really, really bad sleeping 3 – 4 h a night, I’m just stressed out. “

#### Baseline:

Patient seems a little quiet and nervous today. Blood drawn from RAC via IV catheter per practitioners’ orders. 4 SST, 1 LAV. NO UA. Patient self-rates her cravings today at a level 6 out of 10, anxiety and depression at a level 5 out of 10 (situational). Patient denies pain. Recommended natural ways to help with anxiety and depression such as yoga or meditation. Patient states having a good appetite by eating 3 meals a day and drinking approx. 8 – 10 glasses of water daily. Patient states having no BM issues and having a moderate activity level. Patients tolerated treatment well with no pain or discomfort.

#### Patient reports:

Cravings 6/10; anxiety 5/10; depression 5/10; and hours of uninterrupted sleep 6.

#### Infusion 1:

Patient is in a good mood today. Patient self-rates her cravings at a level 4 out of 10, anxiety at a level 2 out of 10 (situational), and depression at a level 1 out of 10 (situational), and patient denies pain. Recommended natural ways to help with anxiety and depression such as exercise or yoga. Patient states having a good appetite by eating 3 meals a day and drinking approx. 8 – 10 cups of water daily. Patient states having no BM issues and having a moderate activity level. Patients tolerated treatment well with no pain or discomfort.

#### Patient reports:

Cravings 4/10; anxiety 2/10; depression 1/10; and hours of uninterrupted sleep 8.

#### Infusion 2:

Cravings at level 4 of 10, anxiety and depression at level 7 of 10 due to (emotional/situational/family), advised of different ways to help lower anxiety and depression such as exercise and meditation, eats 1 to 2 meals per day, drinks about 20 oz of water daily, advised to increase water intake, patient reports feeling drained and with no energy, can sleep for up to 5 h, appear dehydrated as shown by dry skin and chapped lips, tolerated treatment well with no pain or discomfort.

#### Patient reports:

Cravings 4/10; anxiety 7/10; depression 7/10; and hours of uninterrupted sleep 5.

#### Infusion 3:

Cravings at level 4 of 10, anxiety and depression at level 7 of 10 due to emotional/situational/family, advised of different ways to help lower anxiety and depression such as exercise and meditation, eats 1 to 2 meals per day, drinks about 20 oz of water daily, advised to increase water intake, patient reports feeling drained and with no energy, can sleep for up to 5 h, appear dehydrated as shown by dry skin and chapped lips, tolerated treatment well with no pain or discomfort.

#### Patient reports:

Cravings 4/10; anxiety 7/10; depression 7/10; and hours of uninterrupted sleep 5.

#### Infusion 4:

Cravings at level 3 of 10, anxiety and depression at level 7 of 10 due to (emotional/situational/family), advised of different ways to help lower anxiety and depression such as exercise and meditation, eats 1 to 2 meals per day, drinks about 20 oz of water daily, advised to increase water intake, patient reports feeling drained and with no energy, can sleep for up to 5 h, appear dehydrated as shown by dry skin and chapped lips, tolerated treatment well with no pain or discomfort.

#### Patient reports:

Cravings 3/10; anxiety 7/10; depression 7/10; and hours of uninterrupted sleep 6.

#### Infusion 5:

Cravings at level 3 of 10, anxiety at level 7 of 10 and depression at level 5 of 10 due to (emotional/situational/family), advised of different ways to help lower anxiety and depression such as exercise and meditation, eats 2 to 3 meals per day, drinks about 30 oz of water daily, advised to increase water intake, patient reports feeling drained and with no energy, can sleep for up to 5 h, appear dehydrated as shown by dry skin and chapped lips, tolerated treatment well with no pain or discomfort.

#### Patient reports:

Cravings 3/10; anxiety 7/10; depression 5/10; and hours of uninterrupted sleep 6.

#### Infusion 6:

Cravings at level 3 of 10, anxiety at level 7 of 10 and depression at level 5 of 10 due to emotional/situational/family, advised of different ways to help lower anxiety and depression such as exercise and meditation, eats 2 to 3 meals per day, drinks about 30 oz of water daily, advised to increase water intake, can sleep for up to 5 h, appears dehydrated as shown by dry skin and chapped lips, tolerated treatment well with no pain or discomfort.

#### Patient reports:

Cravings 3/10; anxiety 7/10; depression 5/10; and hours of uninterrupted sleep 6.

#### Infusion 7:

Patient reports no cravings, anxiety at level 10 of 10 and depression at level 9 of 10, eats 2 or 3 meals per day, drinks 20 oz of water daily, advised to increase water intake, slept for 7 h last night, low energy level, patient reports that infusions help with anxiety and depression, Tolerated treatment well with no pain or discomfort.

#### Patient reports:

**Figure 14:**
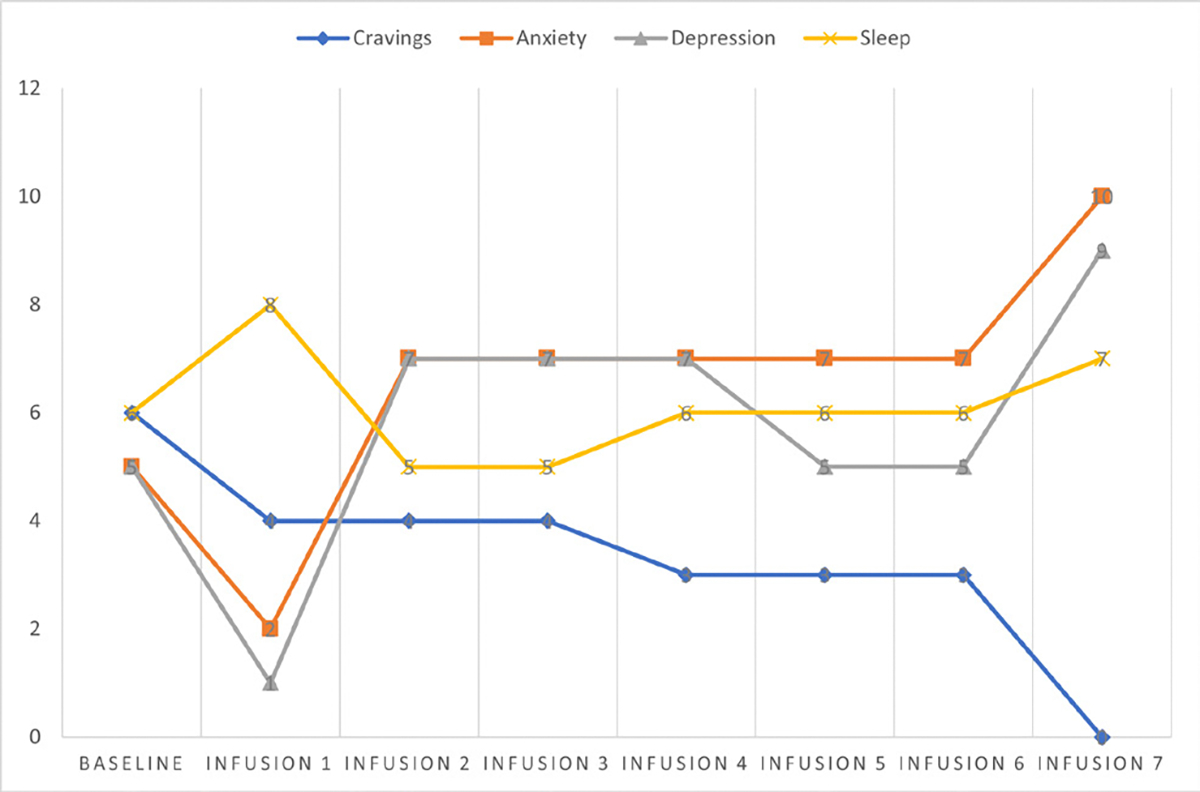
Patient 13 (Female/21/meth/benzos).

Cravings 0/10; anxiety 10/10; depression 9/10; and hours of uninterrupted sleep 7 (Table 15 and figure 14).

### Patient 14 (Female/61/opiates/meth)

#### Consultation:

Patient presents to clinic for NAD consultation. DOC is meth and heroin via smoking 1 g daily. Last use was 22 days ago. The longest sobriety was 90 days 5 years ago and has been using it for 20 years. Has been to 3 treatment facilities. Denies any OD or seizures in the past. Currently “I have a lot of fatigue, and no energy all day. I have insomnia and only sleep 6 h while waking 5 to 6 times a night and I have super high anxiety. I don’t drink any water either.”

#### Baseline:

Patient reports to first infusion with complaints of severe anxiety and restless sleep. Patient reports waking up with night sweats. Encouraged patients to practice meditation to help with anxiety. Patient was receptive and expressed understanding. The patient was observed relaxing on recliner and talking on her phone during infusion.

#### Patient reports:

Cravings 4/10; anxiety 6/10; depression 2/10; and hours of uninterrupted sleep 4.

#### Infusion 1:

Patient reports an increase in her energy levels and her mood has significantly gotten better. Patient reports feeling a great difference with her first infusion. Patient was observed to be cheerful, relaxing on recliner and conversing with peers during infusion. Patient tolerated treatment well with no complaint of discomfort.

#### Patient reports:

Cravings 4/10; anxiety 4/10; depression 0/10; and hours of uninterrupted sleep 5.

#### Infusion 2:

Patient reports increase in anxiety and cravings. Encouraged patients to practice meditation and breathing exercises to help with anxiety. The patient reports her sleep has improved and wakes up with plenty of energy. Patient was observed to be relaxing on recliner and conversing with peers.

#### Patient reports:

Cravings 4/10; anxiety 7/10; depression 3/10; and hours of uninterrupted sleep 5.

#### Infusion 3:

Patient reports her anxiety and depression have been high. Patient has been working with her therapist and working on expressing her emotions rather than holding everything in. Patient has been working on her communication with her daughter. Encouraged the patient to continue working with her therapist and to stay positive. Encouraged the patient to practice meditation and breathing exercises to help ease her anxiety. Patient was receptive.

#### Patient reports:

Cravings 0/10; anxiety 7/10; depression 6/10; and hours of uninterrupted sleep 6.

#### Infusion 4:

Patient reports increased anxiety and difficulty sleeping. Patient reports she feels it’s the heat that wakes her up at night. Encouraged patients to practice meditation and breathing exercises to help with anxiety and to try cooling measures like lighter clothing or a change of bedding to keep comfortable temperature. Patient was receptive. Patient states she has been cutting sugary drinks and watching what she eats to help with her glucose levels. Patient states she is working to schedule her appointment with an endocrinologist to follow up with her recent blood work. Patient was observed relaxing and conversing with peers during infusion.

#### Patient reports:

Cravings 0/10; anxiety 3/10; depression 0/10; and hours of uninterrupted sleep 5.

#### Infusion 5:

Patient reports better sleeping patter since she started infusions. Patient states she’s happy to be feeling better and with more energy. The patient has had a recent medication change to help with her hypertension. Patient states she only had 8 oz of water today but is trying to increase her water intake. Encouraged patients to increase fluid intake and educated on the importance of hydration. Patient was receptive. Patient was observed relaxing and conversing with peers during infusion.

#### Patient reports:

Cravings 0/10; anxiety 3/10; depression 0/10; and hours of uninterrupted sleep 8.

#### Infusion 6:

Patient reports she has noticed a big difference in her sleeping patterns. Patient states to sleep well and have steady energy levels throughout the day. The patient was observed relaxing during infusion.

#### Patient reports:

Cravings 0/10; anxiety 0/10; depression 0/10; and hours of uninterrupted sleep 8.

#### Infusion 7:

Client reports the most notable difference is sleeping through the night. Client states being able to sleep through the night has improved many things like bettering her mood and increasing her energy level. Client states she used to not be able to make it through the day without needing rest/a nap. Client reports she seems to focus better on her treatment and classes. IV site dry and intact, client tolerated infusion well.

#### Patient Reports:

**Figure 15:**
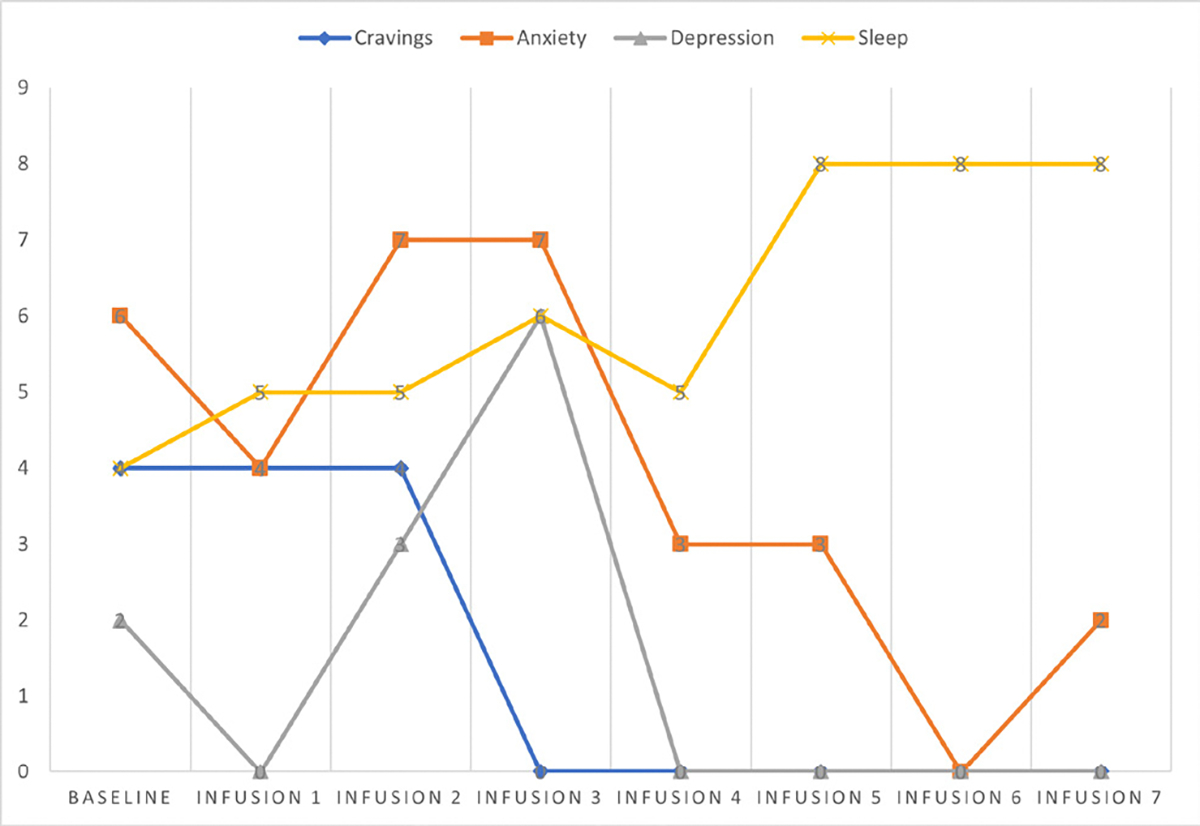
Patient 14 (Female/61/opiates/meth).

Cravings 0/10; anxiety 2/10; depression 0/10; and hours of uninterrupted sleep 8 (Table 16 and figure 15).

### Patient 15 (Female/29/meth)

#### Consultation:

Patient presents to clinic for NAD consult arriving yesterday morning from OK city. PMH denies, denies any surgeries, denies any psych hx. Denies any med use daily. Denies any OD or seizures. DOC is meth via eating 1½ g daily. Last use was yesterday, longest sobriety is 1 year in 2014 – 2015. Has been to 1 other treatment facility in the past. Started using it at age 18. Currently denies any chest pain, no SOB, no abdominal pain, no n/v/d. “I have trouble with hydration and makes me feel weak, I always used to feel like I had more energy while using meth, so I didn’t feel like I needed to hydrate myself. Now I feel the effects of being tired all the time. I also get irritable easily with people around me.”

#### Baseline:

Patient reports this is her first infusion. Patient reported anxiety was at a 6 out of 10 prior to IV insertion, patient reports not liking needles and unsure about starting infusions. After successful IV insertion patient states anxiety decreased to a 3 out of 10, patient reports anxiety and depression is normally around a 3 out of 10, advised patient to work through feelings using breathing exercises and grounding techniques. Advised patient to discuss with case manager, patient expresses understanding.

#### Patient reports:

Cravings 1/10; anxiety 3/10; depression 3/10; and hours of uninterrupted sleep 7.

#### Infusion 1:

Patient reports that her sleep and energy have improved substantially and help with her overall sense of well-being. Patients reports anxiety and depression is a 2 out of 10, advised patients to work through anxiety and depression through breathing exercises and grounding techniques.

#### Patient reports:

Cravings 0/10; anxiety 1/10; depression 2/10; and hours of uninterrupted sleep 8.

#### Infusion 2:

Patient reports anxiety and depression is a 2 out of 10, advised patients to work through anxiety and depression through breathing exercises and grounding techniques. Advised patient to discuss feelings with case manager, patient expressed understanding.

#### Patient reports:

Cravings 0/10; anxiety 1/10; depression 2/10; and hours of uninterrupted sleep 8.

#### Infusion 3:

Patient states she isn’t experiencing any cravings for stimulant, opiates or alcohol. 2 out of 10 patients report anxiety and depression is a 2 out of 10 advised patients to work through anxiety and depression through breathing exercises and grounding techniques. Advised patient to discuss feelings with case manager, patient expressed understanding.

#### Patient reports:

Cravings 0/10; anxiety 1/10; depression 2/10; and hours of uninterrupted sleep 8.

#### Infusion 4:

Patient reports sleeping 8 h at night, patient states she sleeps soundly and only wakes up when she needs to use the restroom but has no issues falling back to sleep. Patient expressed experiencing cravings 7 out of 10, patient states she has meetings every day and talks to her sponsors every day. Advised patient to discuss with case manager as well, patient expresses understanding.

#### Patient reports:

Cravings 0/10; anxiety 1/10; depression 2/10; and hours of uninterrupted sleep 8.

#### Infusion 5:

Patient reports “I have more energy” patient states she sleeps approximately 8 – 10 h an evening and feels rested after sleep. No cravings or opiates or stimulants. Patient states she remains determined to stay sober. Patient states she drinks 1 can of soda daily and about 36 oz of water a day, advised patient to increase water intake, patient expresses understanding.

#### Patient reports:

Cravings 0/10; anxiety 0/10; depression 0/10; and hours of uninterrupted sleep 10.

#### Infusion 6:

Patient reports activity level is “good” patient states she tries to stay active, walks the house dog and goes for runs. Patient states she is sad because she started a new job yesterday and was let go on the same day because there was a red flag on her background check, patient states she is trying her best to be good and goes to her meetings and talks to her sponsor every day, advised patient to focus on her sobriety and her meetings and things will fall into place, advised patient to discuss any issues and feelings with case manager, patient expresses understanding.

#### Patient reports:

Cravings 2/10: anxiety 2/10; depression 1/10; and hours of uninterrupted sleep 9.

#### Infusion 7:

Client states some of her housemates don’t seem to be as focused on their sobriety and it upsets her. Advised client to focus on her sobriety and her meetings and things will fall into place, advised client to discuss any issues and feelings with case manager, client expresses understanding. Patient reports activity level is “good” client states she tries to stay actives, walks to house dog and goes for runs. IV site dry and intact, client tolerated infusion well.

#### Patient reports:

**Figure 16:**
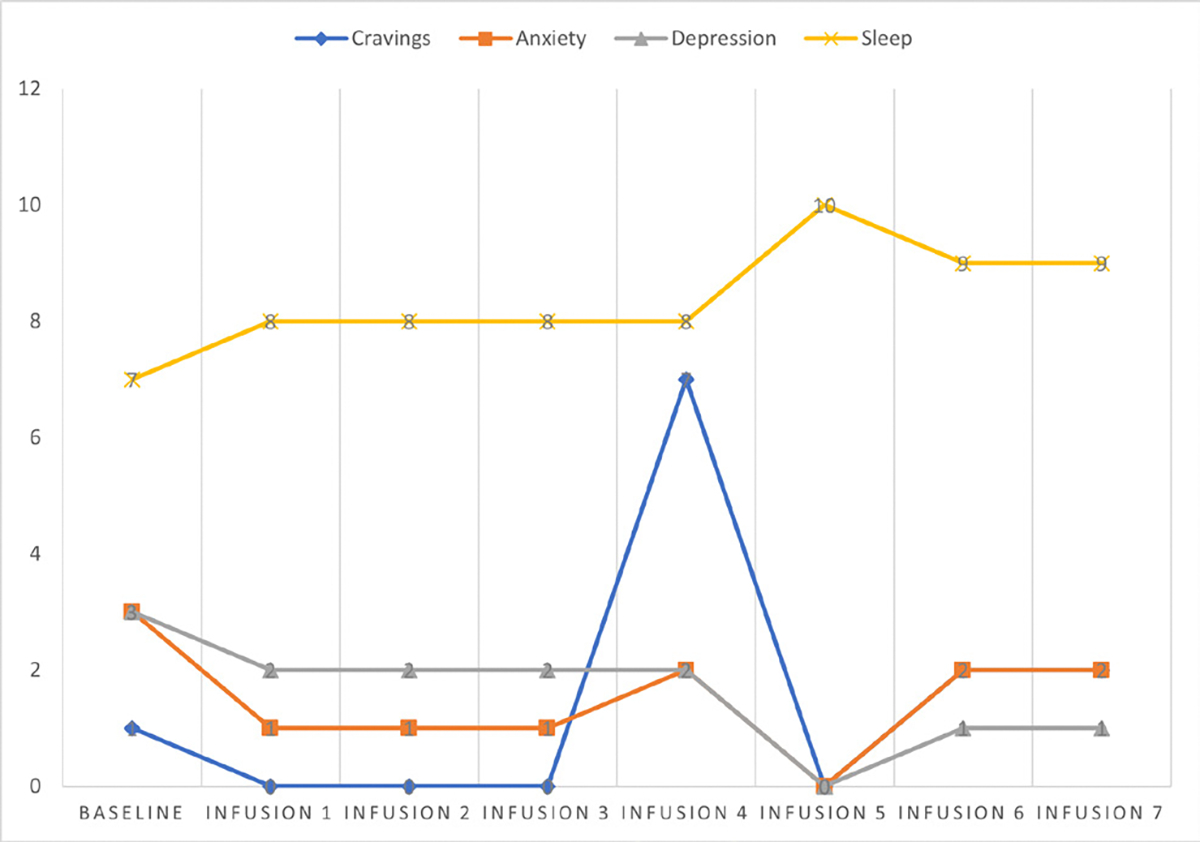
Patient 15 (Female/29/meth).

Cravings 2/10: anxiety 2/10: depression 1/10; and hours of uninterrupted sleep 9 (Table 17 and figure 16).

### Patient 16 (Female/28/meth/ETOH)

#### Consultation:

Patient presents to clinic for NAD consult from OK. DOC was alcohol drinking 12 pack daily and meth via smoking and snorting. Last use was 46 days ago. This is patient’s longest sobriety and has been to 7 treatment facilities. Denies any OD or seizures in the past. Currently “I have pretty much all symptoms, I wake up once every hour and tired throughout the day. I’ve been in my depressed state of my bipolar. I have 2 – 3 glasses of water daily. I get anxiety 1x a day and I take deep breaths to calm myself down.”

#### Baseline:

Patient reports for first treatment infusion. Reports that she is feeling very depressed and anxious. Was drinking heavily prior to coming to detox and feels very fatigued and depleted. Was not eating much when she was drinking heavily. Reports anxiety 10/10 and cravings 9/10. Discussed deep breathing and meditation to help with anxiety and depression. Patient was receptive. Patient relaxed in recliner and chatted with others during infusion.

#### Patient reports:

Cravings 9/10; anxiety 10/10; depression 10/10; and hours of uninterrupted sleep 4.

#### Infusion 1:

Patient reports for second treatment infusion. Reports that she is feeling very depressed and anxious. Reports anxiety 8/10 and cravings 9/10. Advised patient on ways to work through anxiety and depression through breathing exercises and grounding techniques, advised patient to discuss with case manager. Patient expresses understanding. Patient states she feels improvements overall.

#### Patient reports:

Cravings 5/10; anxiety 8/10; depression 8/10; and hours of uninterrupted sleep 4.

#### Infusion 2:

Patient reports anxiety and depression are around 8 out of 10. Advised patient on ways to work through anxiety and depression through breathing exercises and grounding techniques, advised patient to discuss with case manager. Patient expresses understanding. Patient states she feels improvements overall.

#### Patient reports:

Cravings 5/10; anxiety 8/10; depression 8/10; and hours of uninterrupted sleep 4.

#### Infusion 3:

Patient states she had a using dream, and she has them here and there but doesn’t feel like using them. Patients report anxiety and depression is around a 6 out of 10. Advised patient on ways to work through anxiety and depression through breathing exercises and grounding techniques, advised patient to discuss with case manager. Patient expresses understanding.

#### Patient reports:

Cravings 2/10; anxiety 6/10; depression 6/10; and hours of uninterrupted sleep 4.

#### Infusion 4:

Patient reports no cravings for stimulants or alcohol. Patient reports approximately 4 h of sleep a night, states she wakes up frequently to use the restroom and she also has vivid dreams which interrupts her restful sleep. Patients drink about 2 cups of coffee a day, and drink approximately 106 oz of water daily. The patient is on Wellbutrin, lithium and quetiapine. Patient states IV’s have helped decrease her depression and increased her energy level.

#### Patient reports:

Cravings 0/10; anxiety 4/10; depression 3/10; and hours of uninterrupted sleep 4.

#### Infusion 5:

Patient reports anxiety and depression are around a 4 out of 10. Advised patient on ways to work through anxiety and depression through breathing exercises and grounding techniques, advised patient to discuss with case manager. Patient expresses understanding. Patient states she had a using dream, and she has them here and there, but it doesn’t feel like using. The patient states she has chronic lower back pain 4 out of 10 levels, infusions have helped decrease pain slightly and improved mobility by making movement much more tolerable.

#### Patient reports:

Cravings 2/10; anxiety 4/10; depression 3/10; and hours of uninterrupted sleep 4.

#### Infusion 6:

Patient reports getting roughly 7 h of sleep, patient states her sleep is sometimes broken because she often has very vivid dreams. Sometimes her dreams are about using but she doesn’t have a desire to break her sobriety. Patient states due to her using dreams she would rate her cravings at a 2 out of 10, advised patient to discuss with case manager, patient expresses understanding. Patient states she is feeling less depressed.

#### Patient reports:

Cravings 2/10; anxiety 0/10; depression 0/10; and hours of uninterrupted sleep 7.

#### Infusion 7:

Patient reports good activity level, she is walking around her neighborhood while practicing social distancing. Client states her anxiety has improved but she has always been a jumpy person and reported a rating of 1 out of 10, advised client to practicing breathing exercises and grounding techniques and to discuss feelings with case manager, client expresses understanding. IV site dry and intact, client tolerated infusions well.

#### Patient reports:

**Figure 17:**
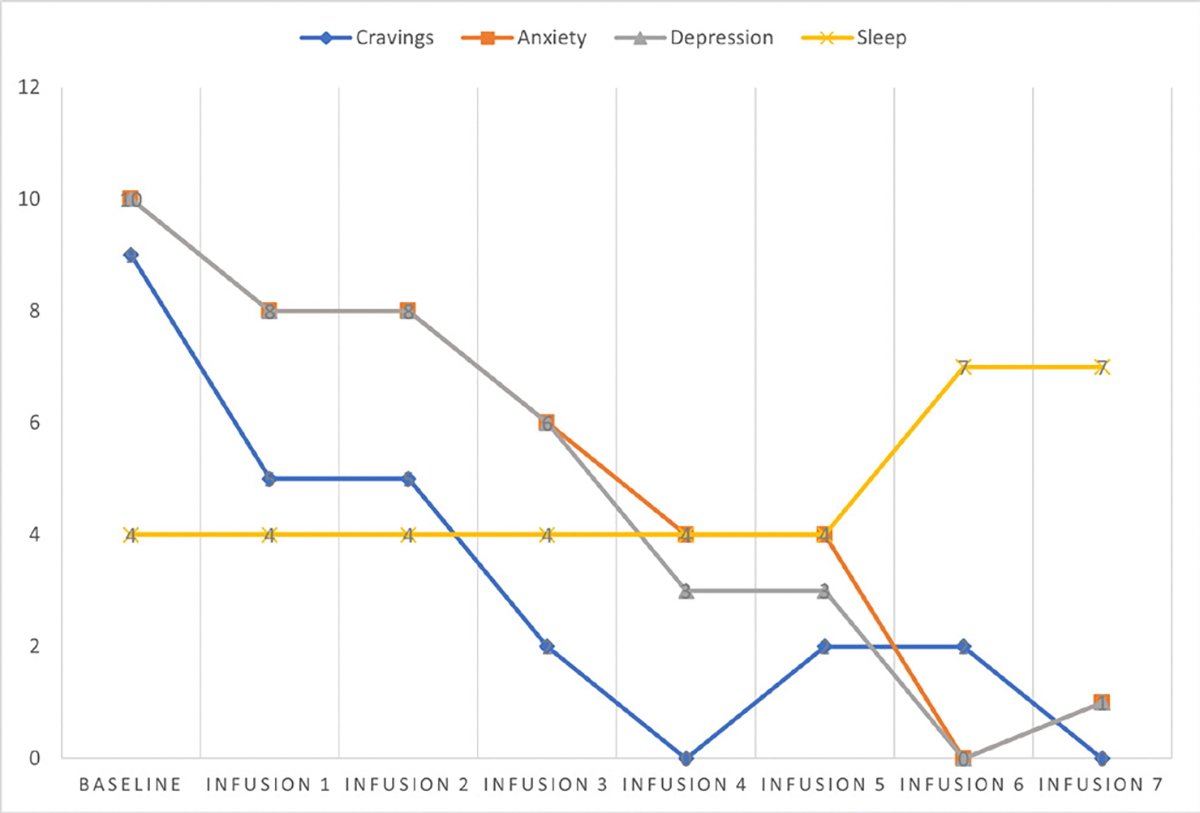
Patient 16 (Female/28/meth/ETOH).

Cravings 0/10; anxiety 1/10; depression 1/10; and hours of uninterrupted sleep 7 (Table 18 and figure 17).

### Patient 17 (Female/36/meth/cocaine)

#### Consultation:

Patient presents to clinic for NAD consult from South Coast BH. DOC is meth, cocaine, last use was 2/12/2020. The longest sobriety is currently. This is a patient’s first treatment facility. Denies any OD or seizures in the past. Currently “I have a hard time concentrating and my memory is really, really bad”. I have a hard time keeping water down, so I am on Zofran.”

#### Baseline:

Patient reports “hating” needles and is proud of herself for coming to her appt. Anxiety at a 5/10 prior to IV insertion, however, patient was “very happy and surprised” once IV cath was inserted and reported a 2 of 10 anxiety level. Currently no other complaints or concerns but overall, she “just wants to WANT to be sober.” Encouraged patient to continue coming to her infusion appts in order to benefit from them the greatest and patient understood.

#### Patient reports:

Cravings 3/10; anxiety 5/10; depression 4/10; and hours of uninterrupted sleep 6.

#### Infusion 1:

Patient reports for infusion treatment reports that she is feeling better than last week. Reports that her sleep and overall energy has improved. Patient reports that she has no cravings and feels more motivated to stay sober than ever. The patient reports her anxiety has decreased to a 1/10 and feels it is more manageable than last week. Patient relaxed in recliner and chatted with others during infusion.

#### Patient reports:

Cravings 0/10; anxiety 1/10; depression 1/10; and hours of uninterrupted sleep 8.

#### Infusion 2:

Patient reports that her vision and sleep have improved. She shows more joy and comfort in her demeanor, and she shows more happiness overall. Patient reports that she has been making positive changes in healthy habits and wants to remain sober. Patient relaxed in recliner and chatted with others during infusion. She ate pizza and tolerated the infusion well, without any complaints of discomfort.

#### Patient reports:

Cravings 0/10; anxiety 2/10; depression 0/10; and hours of uninterrupted sleep 7.

#### Infusion 3:

Patient reports that she slept good last night and feels really energized today. Patient reports that her fibromyalgia has improved, and her pain and inflammation has decreased making it a lot easier to get out of bed. Patient relaxed in recliner and chatted with others during infusion.

#### Patient reports:

Cravings 0/10; anxiety 2/10; depression 2/10; and hours of uninterrupted sleep 7.

#### Infusion 4:

Patient reports that she was bored over the weekend as everyone must stay home during the coronavirus orders. Not much to do around the house but she still tried to stay occupied. The patient denies having any cravings, 0/10 and her anxiety has improved, and rates it at a 2/10. Patient relaxed in recliner and chatted with others during infusion.

#### Patient reports:

Cravings 0/10; anxiety 2/10; depression 0/10; and hours of uninterrupted sleep 8.

#### Infusion 5:

Patient reports that her energy is still maintaining and her decrease in inflammation from her fibromyalgia is less bothersome in the morning. Patient reports that she misses her son and feels bored and anxious with the covid 19 restrictions. Patient has been trying to stay busy, but it has been tough to be in the house with roommates and the same people day after day. Patient relaxed in recliner and chatted with others during the infusion. Patient tolerated treatment well and had no complaints of discomfort.

#### Patient reports:

Cravings 0/10; anxiety 3/10; depression 1/10; and hours of uninterrupted sleep 8.

#### Infusion 6:

Patient states she does not consume caffeinated beverages, and she drinks approximately 24 oz of water daily. Patient reports she gets about 8 h of uninterrupted sleep. Patient states her anxiety has decreased and her mood has improved and feels that has helped her sleep tremendously. Advised patient to increase water intake, patient expresses understanding. Patient states infusions have helped with her vision and overall mood.

#### Patient reports:

Cravings 0/10; anxiety 3/10; depression 4/10; and hours of uninterrupted sleep 8.

#### Infusion 7:

Patient reports cravings at level 0 of 10, anxiety at level 3 of 10 (situational). Depression at level 4 of 10. Advised different ways to reduce anxiety and depression such as exercise and meditation. No fever, pain at level 4 of 10 (Fibromyalgia AM/PM). Sedentary activity level. Patient gets 8 h of sleep per night. Good appetite; eats 2 meals per day. Drink 3 caffeinated drinks/ 24 oz water per day. Patients have been craving less sugar, eating more whole foods and have improved vision since starting infusions. Tolerated treatment well with no pain or discomfort.

#### Patient reports:

**Figure 18:**
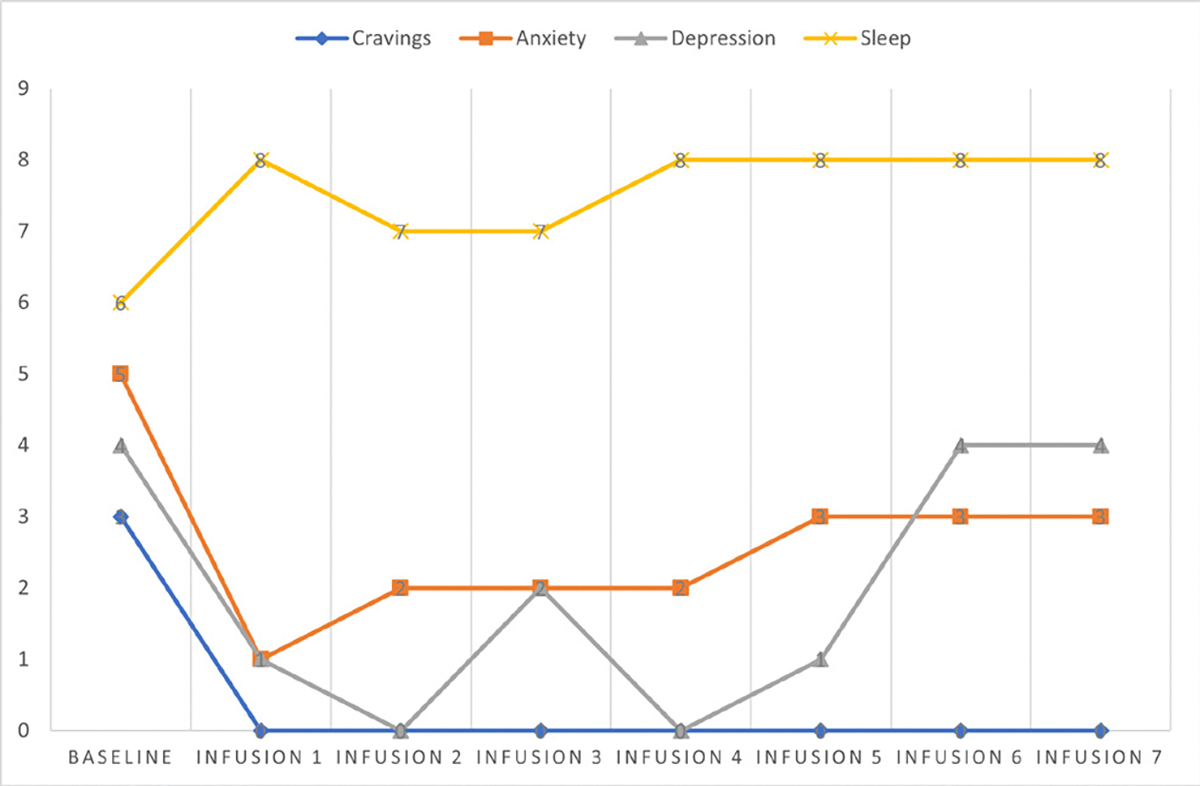
Patient 17 (Female/36/meth/cocaine)

Cravings 0/10; anxiety 3/10; depression 4/10; and hours of uninterrupted sleep 8 (Table 19 and figure 18).

### Patient 18 (Female/30/heroin)

#### Consultation:

Patient presents to clinic for NAD consultation. DOC is opiates, heroin via IV and smoking using 1 – 2 g daily. Last use was 4/8, longest sobriety is 2 years in 2018. Has been to 2 other treatment facilities in the past. 1 OD and 1 seizure in the past. Currently I have “lack of energy, I sweat a lot when I’m sleeping and not drinking water, I get headaches as well. I also have a high level of anxiety that I function on.”

#### Baseline:

Patient reports for first infusion with complaints of high anxiety levels and cravings. Encouraged patients to practice calming techniques like meditation, patient was receptive. Patient was observed relaxing on recliner watching TV during infusion. Patient tolerated treatment well with no complaints of discomfort.

#### Patient reports:

Cravings 8/10; anxiety 6/10; depression 0/10; and hours of uninterrupted sleep 8.

#### Infusion 1:

Patient reports feeling tired and having trouble falling asleep. Encouraged patients to practice meditation to help relax before bedtime. Patient was receptive. Patient is still having cravings and is trying to work through them. Encouraged exercise and journaling to help with the thoughts. The patient was observed relaxing on and talking on her phone during infusion. Patient tolerated treatment well with no complaint of discomfort.

#### Patient reports:

Cravings 7/10; anxiety 6/10; depression 2/10; and hours of uninterrupted sleep 6.

#### Infusion 2:

Patient reports that she has noticed an increase in her overall energy and ability to focus. Patient reports that her sleep has improved, and she is feeling a lot better all around. Patient is excited to see her brother that lives in LA this weekend now that she is off blackout. Patient reports a major reduction in cravings at a 2/10. Patient relaxed in recliner and chatted with others during infusion. Patients tolerated infusions well and had no complaints of discomfort.

#### Patient reports:

Cravings 2/10; anxiety 0/10; depression 0/10; and hours of uninterrupted sleep 6.

#### Infusion 3:

Patient reports having difficulty staying asleep. Encouraged patients to practice meditation and to avoid drinking fluids before bedtime. Patient was receptive and expressed understanding. Patient was observed relaxing on recliner and conversing with peers during infusion. Patient tolerated treatment with no complaint of discomfort.

#### Patient reports:

Cravings 0/10; anxiety 0/10; depression 0/10; and hours of uninterrupted sleep 6.

#### Infusion 4:

Patient reports increase in anxiety due to restless sleep and weird nightmares her sleeping medication is causing. Patient was encouraged to discuss her medication with her provider and also to practice meditation before bed to help with anxiety. Patient was receptive. Patient was observed relaxing on couch and watching TV during infusion. Patient tolerated treatment well with no complaint of discomfort.

#### Patient reports:

Cravings 0/10; anxiety 2/10; depression 0/10; and hours of uninterrupted sleep 7.

#### Infusion 5:

Patient reports moderate level of activity, patient reports she tries to stay active but finds it tough with the quarantine. Advised patients to take walks and even do at home exercise, patient expressed understanding. Patient states she drinks about 1 caffeinated beverage a day and about 3 cups of water, advised patient to increase water intake, patient expressed understanding.

#### Patient reports:

Cravings 0/10; anxiety 0/10; depression 0/10; and hours of uninterrupted sleep 8.

#### Infusion 6:

Patient reports she is happy and noticed improved energy levels. Patient states she is sleeping about 9 h an evening and does not wake up throughout the night, patient reports waking up with improved energy and a sense of calmness. IV site dry and intact, patient tolerated infusion well.

#### Patient reports:

Cravings 0/10; anxiety 0/10; depression 0/10; and hours of uninterrupted sleep 9.

#### Infusion 7:

Patient reports more energy in the morning, experiencing better sleep and smoking less and lowered anxiety. Client reports she notices a lot of differences and it has improved her well-being and patience. Client states she was impatient with the infusions before, but now that she sees improvements, she is okay with the amount of time that needs to be taken on the infusion. IV site dry and intact, client tolerated infusion well.

#### Patient reports:

**Figure 19:**
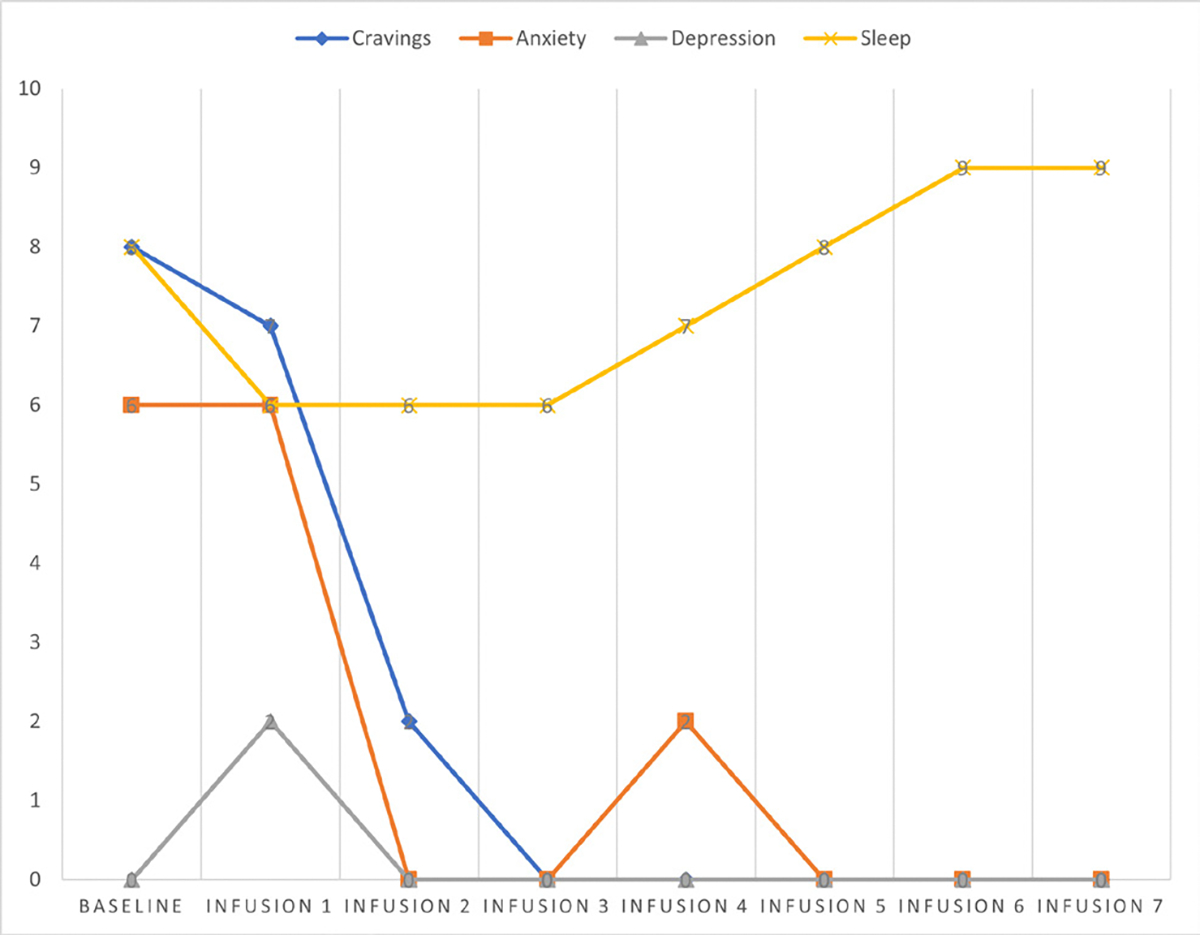
Patient 18 (Female/30/heroin).

Cravings 0/10; anxiety 0/10; depression 0/10; and hours of uninterrupted sleep 9 (Table 20 and figure 19).

### Patient 19 (Female/29/meth/benzos/ETOH/marijuana)

#### Consultation:

Patient presents to clinic for NAD consultation. DOC is meth, benzos, alcohol and marijuana via eating, and meth was IV. Last use was 5/1. The longest sobriety is 17 months, and this is a patient’s first treatment facility. Denies any OD or seizures in the past. Currently “I am irritable all the time and have a really hard time sleeping and I have no energy. I also am nauseated and dehydrated daily.”

#### Baseline:

Patient reports to first infusion. Patient reports increased anxiety and cravings for alcohol. Encouraged patients to practice meditation and breathing exercises to help ease anxiety. The patient was observed relaxing on recliner and on her phone during infusion. Patient tolerated treatment well with no complaints of discomfort. The patient completed treatment and was left in stable condition.

#### Patient reports:

Cravings 4/10; anxiety 3/10; depression 0/10; and hours of uninterrupted sleep 9.

#### Infusion 1:

Patient reports having increased cravings and feeling of restlessness. Covid and being away from home causes her a lot of stress. Patient teaching on ways to meditate and ground herself with deep breathing practices. Patient verbalized understanding. The patient was observed relaxing on couch and talking on her phone during infusion. Patient tolerated treatment well with no complaint of discomfort. The patient completed treatment and was left in stable condition.

#### Patient reports:

Cravings 4/10; anxiety 0/10; depression 0/10; and hours of uninterrupted sleep 8.

#### Infusion 2:

Patient reports increased energy levels. Patient reports staying active and is happy to be able to go out to the beach and get some sun. patient reports getting 8 h of sleep and feeling great. Patient was observed relaxing on recliner and watching TV during infusion. Patient tolerated treatment well with no complaint of discomfort. The patient completed treatment and was left in stable condition.

#### Patient reports:

Cravings 0/10; anxiety 2/10; depression 0/10; and hours of uninterrupted sleep 8.

#### Infusion 3:

Patient reports an increase in her anxiety due to her friend recently leaving treatment. Encouraged the patient to focus on her recovery and to practice meditation to help with anxiety. The patient tolerated Myers and glutathione treatment with no complaint of discomfort. Patient c/o nausea and stomach cramping during NAD infusion. The patient was given ondansetron 4 mg 2tab SL without incidence. Infusion drip was minimized. Patients reported alleviated symptoms after 10 min. patient completed treatment with no other complaint of discomfort and left in stable condition.

#### Patient reports:

Cravings 0/10; anxiety 5/10; depression 5/10; and hours of uninterrupted sleep 6.

#### Infusion 4:

Patient reports an overall well-being. Patient reports not having a BM for 2 days now. Encouraged patients to increase fluid and fiber intake to help with constipation. Patient was receptive and expressed understanding. Patient reports no cramps or discomfort. The patient reports her anxiety has gotten better and she is trying to make better life choices. The patient was observed relaxing on couch and listening to music during infusion. Patient tolerated treatment well with no complaint of discomfort. The patient completed treatment and was left in stable condition.

#### Patient reports:

Cravings 1/10; anxiety 1/10; depression 0/10; and hours of uninterrupted sleep 8.

#### Infusion 5:

Patient reports she feels good, patient admits she has not been in for a while for infusions. Patient reports that sleep was better when she came in regularly, sleep currently is about 6.5 h an evening, patient sometimes experienced broken sleep, but she practices the breathing exercises, and it helps her fall back to sleep. Patient states she drinks about 24 oz of water a day, advised patient to increase water intake, especially during this heat. Patient expressed understanding. IV site dry and intact, patient tolerated infusion well.

#### Patient reports:

Cravings 0/10; anxiety 2/10; depression 0/10; and hours of uninterrupted sleep 6.

#### Infusion 6:

Patient reports feeling “more focused, improved sleep” patient expressed that after her last infusion she felt very tired and that night she slept very well. Currently a patient reports she feels happy today and is in a good mood. Patient drinks about 24 oz of water a day, advised patient to increase water intake, patient expressed understanding. Educated patient on the importance of water intake especially when it is hot outside. IV site dry and intact, patient tolerated infusion well.

#### Patient reports:

Cravings 0/10; anxiety 0/10; depression 0/10; and hours of uninterrupted sleep 8.

#### Infusion 7:

Client reports feeling “more focused, improved sleep” client expressed that after her last infusion she felt very tired and that night she slept very well. Currently a client reports she feels happy today and is in a good mood. Client drinks about 24 oz of water a day, advised client to increase water intake, client expressed understanding. Educated client on the importance of water intake especially when it is hot outside. IV site dry and intact, client tolerated infusion well.

#### Patient reports:

**Figure 20:**
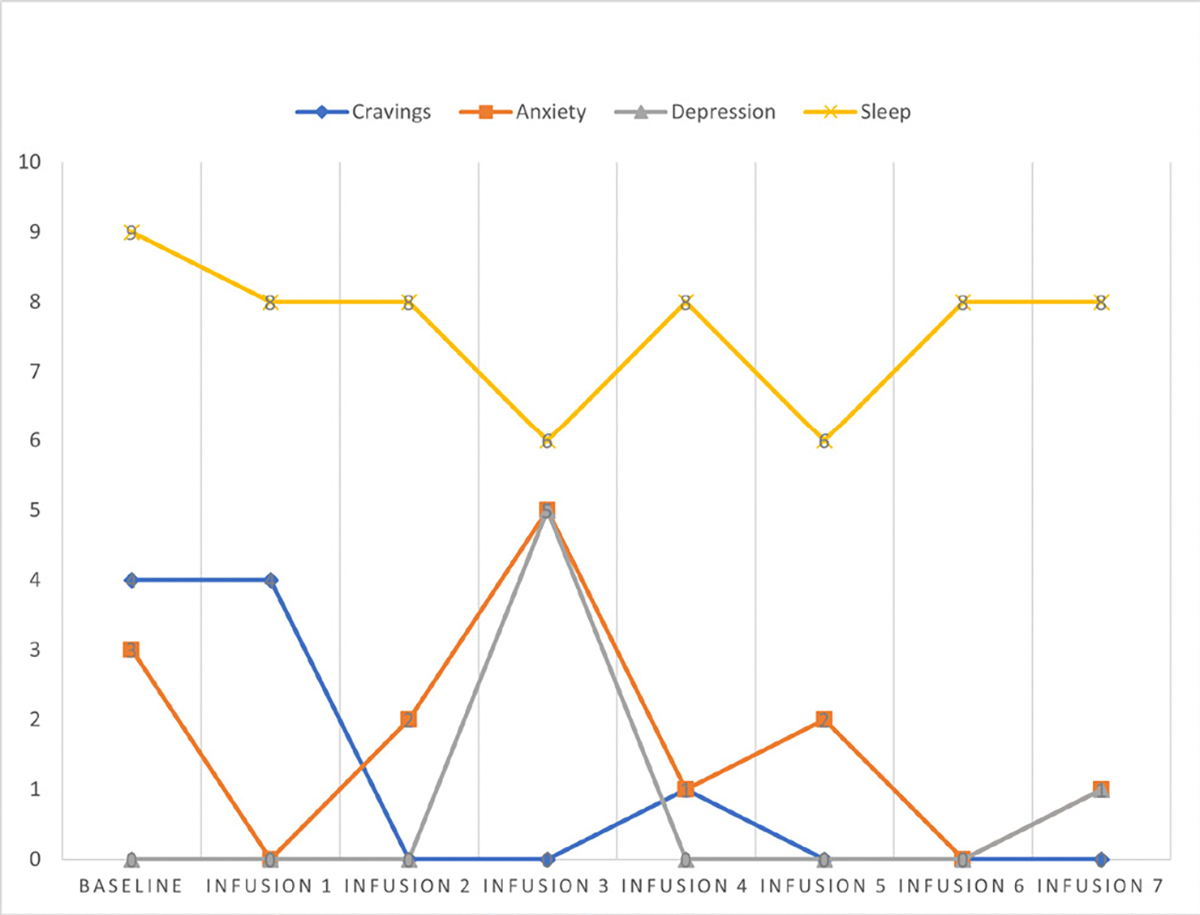
Patient 19 (Female/29/meth benzos/ETOH/marijuana)

Cravings 0/10; anxiety 1/10; depression 1/10; and hours of uninterrupted sleep 8 (Table 21 and figure 20).

### Patient 20 (Female/41/heroin)

#### Consultation:

Patient presents to clinic for NAD consultation. DOC is heroin and meth 1 – 2 g since age of 16. Last use was 1/5, longest sobriety is currently. Has been to 2 other treatment facilities. Denies any seizures and 1 OD on Ambien in 2014. Currently, “I have been experiencing lack of energy, mental processing is better. I sleep 4 h a night. I deal with anger all day but it’s getting better.”

#### Baseline:

Patient reports for first infusion. Patient reports cravings for heroin and meth. Patient states she is nervous about the covid-19 pandemic, she states she has been staying home and not traveling. Patients expressing anxiety and depression is around a 4 out of 10 advised patients on ways to work through anxiety and depression through breathing exercises and grounding techniques. Patient expresses understanding. IV site dry and intact, no signs or symptoms of irritation or infiltration. Patient tolerated infusion well.

#### Patient reports:

Cravings 5/10; anxiety 4/10; depression 4/10; and hours of uninterrupted sleep 7.

#### Infusion 1:

Patient reports sleeping 4 – 5 h a night. Patient states she has difficulty staying asleep and wakes up often to use the restroom. Advised patient on meditation techniques and to not drink fluids so close to bedtime. Patient expresses understanding. Patient reports anxiety due to being in a new environment and from cravings. IV site dry and intact, no signs or symptoms of irritation or infiltration.

#### Patient reports:

Cravings 3/10; anxiety 3/10; depression 0/10; and hours of uninterrupted sleep 5.

#### Infusion 2:

Patient denies having any cravings 0/10. Patient reports 5 h of sleep. Patient states that these are a big improvement. She states she normally sleeps about 3 – 4 h a night and has never had cravings since she began using it. Patient feels her energy has increased and her mood has improved. Patient states he sees a decrease in anxiety and depression. IV site dry and intact, patient tolerated infusion well.

#### Patient reports:

Cravings 0/10; anxiety 2/10; depression 0/10; and hours of uninterrupted sleep 5.

#### Infusion 3:

Patient reports her activity level as “can’t stop won’t stop”, patient expresses that she is trying her best to stay active and busy during this time of quarantine. Patient states her sleep has been amazing at 5 h a night, in the past patient would sleep 3 – 4 h an evening. Blood pressure is elevated, patient states she takes BP meds, advised patient to talk to her doctor about her elevated BP. Patient expresses understanding. IV site dry and intact, patient tolerated infusion well.

#### Patient reports:

Cravings 0/10; anxiety 0/10; depression 0/10; and hours of uninterrupted sleep 5.

#### Infusion 4:

Patient reports “I stay busy” addressing her activity level. Patient states today is her birthday, and she feels positive and energetic. Patient states she is thrilled her sleep has improved, started at 3 – 4 h of sleep a night and now she’s at 5 – 6 h a night. Noticed patients BP was elevated and asked patient if she is on BP medication, patient states she takes it sometimes, advised patient to take medication as directed and monitor her BP and to discuss with her physician, patient expresses understanding. IV site dry and intact, patient tolerated infusion well.

#### Patient reports:

Cravings 0/10: anxiety 0/10; depression 0/10; and hours of uninterrupted sleep 6.

#### Infusion 5:

Patients initial blood pressure prior to infusion is measured at 156/133, asked patient if she took her BP medication today, patient states no because it is controlled and she cannot take it at will, strongly advised patient to discuss with case manager and health care provider about consistently monitoring her blood pressure and to take medications as advised by health care provider, patient expresses understanding. The patient reports her sleep has improved tremendously, she states she slept 7 h last night and was so surprised. Patient reports feeling energized and refreshed after sleep, patient is very happy with results so far. IV site dry and intact, patient tolerated infusion well.

#### Patient reports:

Cravings 0/10; anxiety 2/10; depression 2/10; and hours of uninterrupted sleep 6.

#### Infusion 6:

Today patients BP is 141/92 prior to IV, larger BP cuff was used on patient, patient requires a larger BP cuff. The patient reports her sleep has improved tremendously, she states she slept 7 h last night and was so surprised. Patient reports feeling energized and refreshed after sleep, patient is very happy with results so far. IV site dry and intact, patient tolerated infusion well.

#### Patient reports:

Cravings 0/10; anxiety 2/10; depression 2/10; and hours of uninterrupted sleep 7.

#### Infusion 7:

Patient states “better sleep and no depression” patient appeared to be in a very good mood, she expressed that her activity level has increased, and she is keeping herself busy with housework, workouts and finding new music. Patient states she is happy. The patient reports her sleep has improved tremendously, she states she slept 7 h last night and was so surprised. Patient reports feeling energized and refreshed after sleep, patient is very happy with results so far. IV site dry and intact, patient tolerated infusion well.

#### Patient reports:

**Figure 21:**
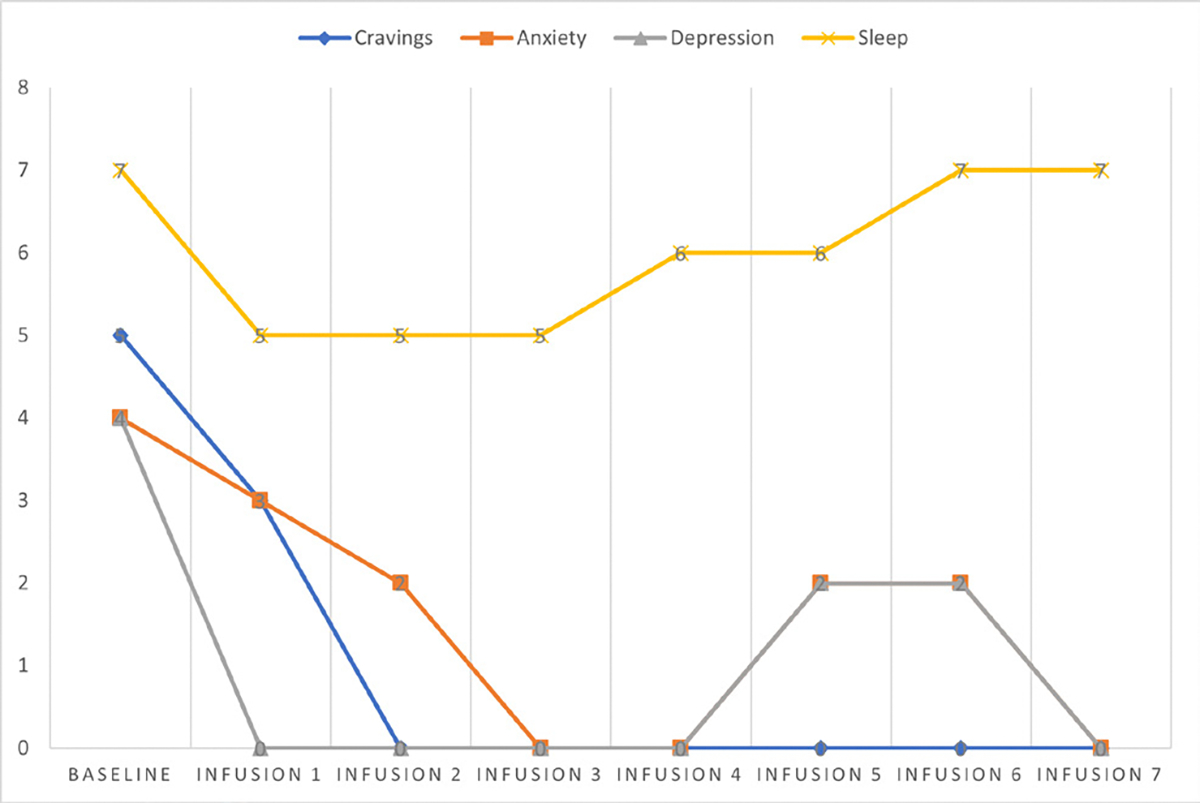
Patient 20 (Female/41/heroin).

Cravings 0/10; anxiety 0/10; depression 0/10; and hours of uninterrupted sleep 7 (Table 22 and figure 21).

### Patient 21 (Female/26/heroin/meth)

#### Consultation:

Patient presents to clinic for NAD consult from South Coast BH. DOC is meth, heroin via IV, using 1 – 2 g. Last use was 2/28. The longest sobriety is currently. This is a patient’s first treatment facility. Denies any seizures or OD I the past. Currently “I have a hard time concentrating, I have really bad anxiety. I have been dehydrated but I eat healthy. Anxiety is my biggest concern it’s a 10/10.”

#### Baseline:

Patient reports anxiety at a 6 of 10 today as she is “terrified” of needles. Explained the process to her and the benefits of infusion and once infusion was initiated, patient’s anxiety dropped to 3 of 10. Although anxiety is currently mild, the patient complains she has trouble throughout her day due to anxiety attacks. Discussed ways to help anxiety (meditation, breathing exercises, etc.). Patient also expressed its hard to be away from home and family and she stills has frequent cravings throughout the day. Discussed journaling and exercise for relief and positive endorphins. Patient tolerated Tx well with no discomfort and left stable with no complications.

#### Patient reports:

Cravings 8/10; anxiety 6/10; depression 3/10; and hours of uninterrupted sleep 5.

#### Infusion 1:

Patient reports high level of activity and feeling well overall. Noticed a lot of relief in cravings after the first infusion. Patient still misses home and her family. Feels a lot of regret. Patient reports anxiety to have decreased. Patient was observed relaxing on couch and conversing with peers during infusion. Patient tolerated treatment well with no complaint of discomfort. The patient completed treatment and was left in stable condition.

#### Patient reports:

Cravings 2/10; anxiety 0/10; depression 3/10; and hours of uninterrupted sleep 8.

#### Infusion 2:

Patient reports she thinks the infusions are helping her with her sleep, patient reports she gets about 7 h of sleep an evening and wakes up feeling rejuvenated and in a good mood. Patient reports she used to be in her head a lot about her feelings and her past, however she is feeling improvements and she’s finally putting herself first. Reports a decrease in cravings of 2/10. IV site dry and intact patient tolerated infusion well.

#### Patient reports:

Cravings 2/10; anxiety 3/10; depression 2/10; and hours of uninterrupted sleep 7.

#### Infusion 3:

Patient reports feeling depressed due to her grandmother passing away yesterday. Patient maintained a positive attitude regardless of her situation. The patient was observed relaxing and talking with peer during infusion. Patient tolerated treatment well with no complaint of discomfort. The patient completed treatment and was left in stable condition.

#### Patient reports:

Cravings 0/10; anxiety 2/10; depression 4/10; and hours of uninterrupted sleep 8.

#### Infusion 4:

Patient reports she is still getting about 7 h of sleep and wakes up feeling rejuvenated and in a good mood. Patient reports that she is handling the situation with her grandma well, really wants to keep her sobriety. She is feeling improvement and she’s finally putting herself first. IV site dry and intact patient tolerated infusion well.

#### Patient reports:

Cravings 0/10; anxiety 0/10; depression 0/10; and hours of uninterrupted sleep 8.

#### Infusion 5:

Patient reports feeling good, she is sleeping better at 8 h and evening of uninterrupted sleep. Patient states she has a history of poor sleep as evidenced by waking up throughout the night to use the restroom and has difficulty falling back to sleep. Advised patient to avoid liquids close to bedtime, patient expressed understanding. The patient states she drinks about 17 oz of water throughout the day, advised the patient to increase water intake and educated the patient on the importance of water intake. IV site dry and intact, patient tolerated infusion well.

#### Patient reports:

Cravings 0/10; anxiety 0/10; depression 0/10; and hours of uninterrupted sleep 8.

#### Infusion 6:

Patient states sleep has been good at around 7 – 8 h an evening; patient wakes up feeling rested and in a good mood. Patient reports that when she does not get enough sleep, she wakes up sluggish and in a bad mood. Good mood and improved energy are good and allows patient to be productive during treatment. IV site dry and intact, patient tolerated infusion well.

#### Patient reports:

Cravings 0/10; anxiety 0/10; depression 0/10; and hours of uninterrupted sleep 7.

#### Infusion 7:

Patient states she feels improved energy and “improved overall well-being”. Patient states she thinks she can see clearer now “if that makes sense” patient states images seemed dull when she was abusing drugs. Patient states her memory is better as well and she thinks she can retain information better. IV site dry and intact, patient tolerated infusion well.

#### Patient reports:

**Figure 22:**
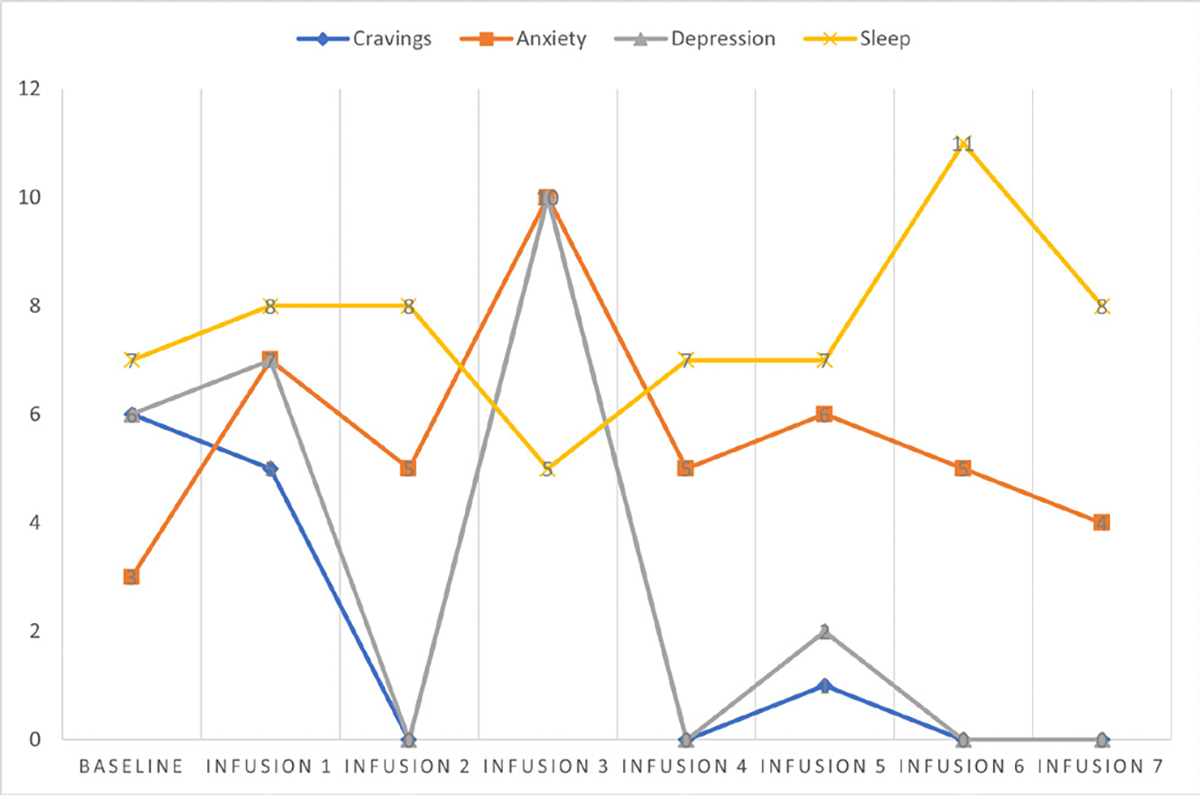
Patient 21 (Female/26/heroin/meth).

Cravings 0/10; anxiety 0/10; depression 0/10; and hours of uninterrupted sleep 8 (Table 23 and figure 22).

### Patient 22 (Male/42/ETOH/benzos)

#### Consultation:

42-year-old male patient presents today for complaints of DOC is alcohol drinking 1/5 daily and Xanax 1 mg 3x a day last use was 7/1. This is my client’s 10^th^ treatment facility. Denies any seizures and several OD in the past. Currently denies any chest pain, no SOB, no abdominal pain, no n/v/d. Currently “I don’t have any motivation or energy to do anything. I experience anxiety daily 3/10 because I am in a safe place. I have difficulty concentrating. I get irritated and angry often and need to walk away. I’m also dehydrated I can’t tell by my skin.”

#### Baseline:

Patient self-rates craving and depression 6/10, anxiety 3/10. Patient c/o left foot pain since January. Patient reports drinking 6 – 8 cups of water and sleeping 7 h. The patient reports he has 3 weeks sober and is happy to continue his path in recovery. Patient was observed relaxing and conversing with peers during infusion. Patient tolerated treatment well with no complaint of discomfort. Patients will have labs drawn on their next visit.

#### Patient reports:

Cravings 6/10; anxiety 3/10; depression 6/10; and hours of uninterrupted sleep 7.

#### Infusion 1:

Patient comes into the office for his 2^nd^ treatment. Patient self-rates his cravings at a level 5 out of 10, anxiety and depression at a level 7 out of 10 (situational), recommended natural ways to help with anxiety and depression such as yoga or meditation. The patient self-rates his pain at a level 4 out of 10, on his left foot and states pain is continuous. Patient states having a moderate appetite by eating 2 meals a day and drinking 120 oz of water. Patient states have no BM issues. The patient states his activity level is low. Patients tolerated treatment well with no pain or discomfort.

#### Patient reports:

Cravings 5/10; anxiety 7/10; depression 7/10; and hours of uninterrupted sleep 8.

#### Infusion 2:

Patient self-rates cravings 0/10, anxiety 5 – 8, and depression 0/10. Patient c/o continuous LLE myalgia. Patient reports drinking 20 oz of water and having a moderate appetite. Patient reports low activity level. Patient reports feeling like he won’t be able to maintain his sobriety if he has to depend on other people. Encouraged patient to communicate with his treatment team and to maintain a positive attitude. Encouraged patients to focus on all the good things and to take it one day at a time. Patient was receptive.

#### Patient reports:

Cravings 0/10; anxiety 5/10; depression 0/10; and hours of uninterrupted sleep 8.

#### Infusion 3:

Patient comes into the office with a worried/stressed look on his face. When asked how things were, he stated he’s had a rough week so far. Was not treated properly at the hospital. Patient self-rates his cravings, anxiety, and depression at a level 10 out of 10 (situational and emotional). Patient states pain at a level 3 out of 10 on his feet and pain comes and goes. Patient states having a poor appetite by only eating 1 meal a day and rinks 3 – 5 water bottles a day. Patient states having a low activity level and having no BM issues. Patients tolerated treatment well with no pain or discomfort.

#### Patient reports:

Cravings 10/10; anxiety 10/10; depression 10/10; and hours of uninterrupted sleep 5.

#### Practitioner FU:

Client presents to clinic for follow up. Currently is infusion 4. States he was placed on topamax and had adverse reactions with severe anxiety and “nervous breakdown”. Client has severe social anxiety and has a hard time putting thoughts together due to frustration and anxiety.

#### Infusion 4:

Patient comes into the office in a better mood today compared to yesterday. Patient seems less anxious and worried. Patient self-rates his anxiety at a level 5 out of 10 (situational), Patient self-rates his pain at a level 1 out of 10, states he has a broken bone, and it only hurts when he puts weight on it. Recommended natural ways to help with anxiety such as yoga or meditation. Patient states having a moderate appetite by eating 1 – 2 meals a day and drinking approx. 60 oz of water daily. Patient states having no BM issues and having a low activity level. Patients tolerated treatment well with no pain or discomfort.

#### Patient reports:

Cravings 0/10; anxiety 5/10; depression 0/10; and hours of uninterrupted sleep 7.

#### Infusion 5:

Patient self-rates cravings 1/10, anxiety 6/10, and depression 2/10. Patient c/o of LLE myalgia that started in January. Patient reports a moderate appetite, drinks 80+ oz of water and sleeps 7 h patient reports he started exercising and is trying to be healthier. Patient reported he has been having a difficult time with his anxiety and recently had an anxiety attack during class. Encouraged patient to communicate with his treatment team and to try meditation or breathing exercises to help ease anxiety. Patient was receptive. The patient was observed watching TV during infusion. Patient tolerated treatment well with no complaint of discomfort.

#### Patient reports:

Cravings 1/10; anxiety 6/10; depression 2/10; and hours of uninterrupted sleep 7.

#### Infusion 6:

Patient is in great spirits today; things are looking up for him and having to adjust to these changes keeps him busy but is excited for them. Patient self-rates his anxiety at a level 5 out of 10 (situational), and pain at a level 6 out of 10 on his left foot and pain is continuous, recommended natural ways to help with anxiety such as yoga or meditation. Patient states having a poor appetite by eating only 1 meal a day and drinking approx. 32 oz of water daily. Patient states having no BM issues and having a low-moderate activity level. Patients tolerated treatment well with no pain or discomfort.

#### Patient reports:

Cravings 0/10; anxiety 5/10; depression 0/10; and hours of uninterrupted sleep 7.

#### Infusion 7:

Patient comes into the office in great spirits. Patient self-rates his anxiety at a level 4 out of 10 (situational) and pain at a level 3 out of 10 on his left foot and pain is continuous. Patient has no fever. Patient states having a poor appetite by only eating 1 meal to a couple of snacks a day and drinking approx. 120 oz of water daily. Patient states having no BM issues and having a high activity level. Patients tolerated treatment well with no pain or discomfort.

#### Patient reports:

**Figure 23:**
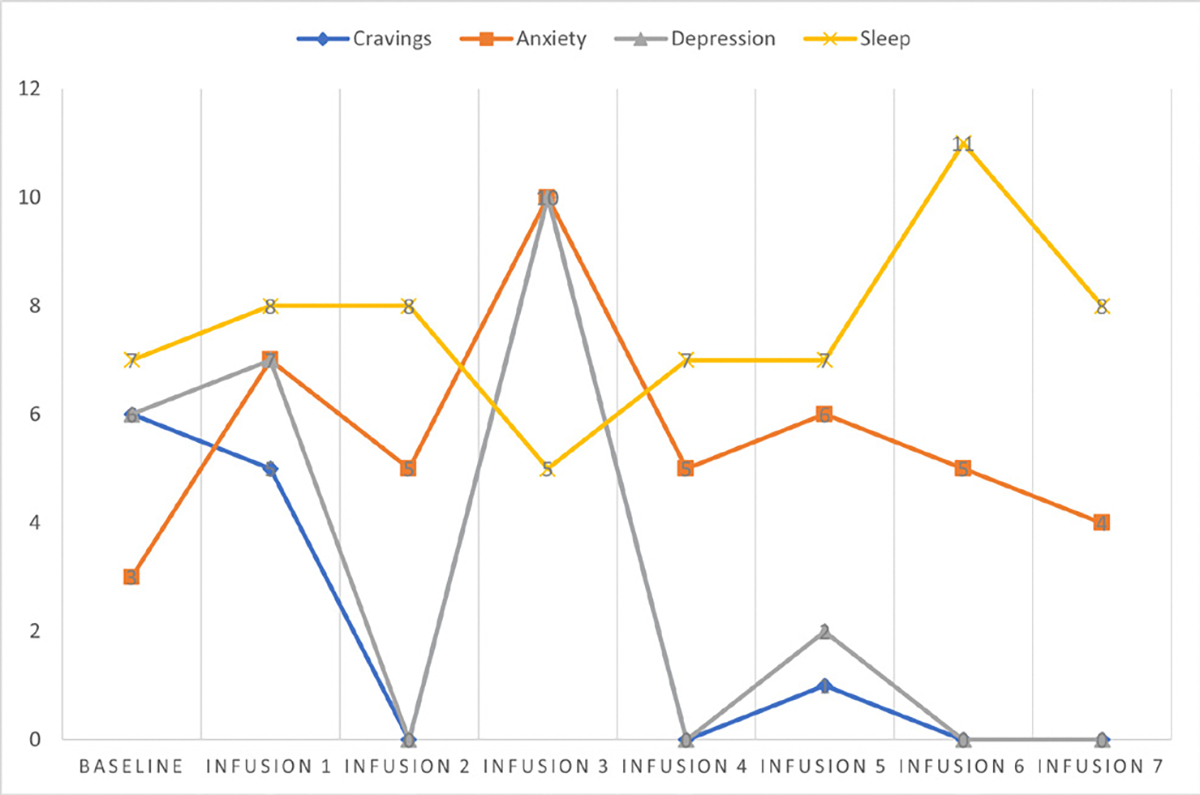
Patient 22 (Male/42/ETOH/benzos).

Cravings 0/10; anxiety 4/10; depression 0/10; and hours of uninterrupted sleep 8 (Table 24 and figure 23).

### Patient 23 (Male/33/meth/ETOH)

#### Consultation:

33-year-old male patient presents today for complaints of “anxiety”. Patients have a long history of drug and alcohol abuse. Patient has a history of drug abuse, states he has tried every kind of drug, but his DOC is speed. He started when he was 12 years old. Last use was on August 14^th^. The longest sobriety was from 2014 – 2016, around 2 years. Patient also has a history of alcohol abuse, started drinking when he was 9 years old. His last drink was on August 15^th^. The longest sobriety was also 2 years from 2014 – 2016. Patient states he also smokes tobacco and chews tobacco, unknown frequency and duration. Patient also mentioned he used to smoke marijuana, started when he was around 9 years old. His longest sobriety was around 2 years.

#### Baseline:

Blood drawn from L AC via IV catheter per practitioners’ orders. 4 SST, 1 LAV, 1 UA. BW sent to INTEGRITOX. Patient self-rates his cravings at 8/10, anxiety at 10/10 (situational), and depression (situational/emotional) at 1/10. Recommended natural ways to help with anxiety and depression such as yoga or meditation. Patient denies pain, has no fever, states eating 3 – 4 meals a day and drinks 64 oz of water daily. Patient denies BM issues and reports having a moderate activity level. Patients tolerated treatment well with no pain or discomfort.

#### Patient reports:

Cravings 8/10; anxiety 10/10; depression 1/10; and hours of uninterrupted sleep 8.

#### Infusion 1:

Patient is in a good mood, a little anxious. Patient states needles make him nervous. Patient self-rates his cravings at 3/10, depression at 1/10 (situational/emotional), and Patient denies pain at the moment. Patient states he eats 3 meals a day and drinks approx. 60 oz of water daily. Patient states having diarrhea and having a moderate activity level. Patients tolerated treatment well with no pain or discomfort.

#### Patient reports:

Cravings 3/10; anxiety 0/10; depression 1/10; and hours of uninterrupted sleep 8.

#### Infusion 2:

Patient comes into the office in a great mood. Patient wanted to know blood results; his BW was not on file. Blood drawn from R AC via IV catheter per practitioners’ orders. 5 SST, 2 LAV, 1 UA. Patient wishes to get tested for STD panel. Salma, NP was notified. Patient self-rates his cravings and anxiety at 1/10 (situational). Recommended natural ways to help with anxiety such as yoga or meditation. Patient states eating 4 meals a day and drinking approx. 60 oz of water daily. Patient states having diarrhea and having a moderate activity level. Patients tolerated treatment well with no pain or discomfort.

#### Patient reports:

Cravings 1/10; anxiety 1/10; depression 0/10; and hours of uninterrupted sleep 8.

#### Infusion 3:

Patient comes into the office in a good mood. Patient self-rates his cravings at 1/10. The patient denies pain at the moment. Patient states he eats 3 – 4 meals a day and drinks approx. 32 – 64 oz of water a day. Patient denies BM issues and states having a moderate activity level. The patient mentioned that he woke up today with less cravings than usual. Patients tolerated treatment well with no pain or discomfort.

#### Patient reports:

Cravings 1/10; anxiety 0/10; depression 0/10; and hours of uninterrupted sleep 8.

#### Infusion 4:

Patient self-rates anxiety 2/10, depression and cravings 1/10. Patient denies myalgia. Patient reports a good appetite 3 – 4 meals a day, drinks 50 oz of water daily and sleeps over 8 h. Patient reports moderate activity levels. Patient reports his cravings have been decreasing but recently had a slip and drank a beer but was able to stop himself from taking it any further. Patient states he is proud to have been able to not drink more. The patient was observed relaxing on couch during infusion. Patient tolerated treatment well with no complaint of discomfort.

#### Patient reports:

Cravings 1/10; anxiety 2/10; depression 1/10; and hours of uninterrupted sleep 8.

#### Infusion 5:

Patient self-rates anxiety 1 – 2/10, depression and cravings 1/10. Patient denies myalgia. Patient reports to be feeling great with his energy levels improving. Patient reports his mood has been better as well. Patient reports good appetite, sleeps 8 h and drinks 40 – 60 oz of water daily. No GI complaints. Patient was observed cheerfully conversing with staff and relaxing watching TV during infusion. Patient tolerated treatment well with no complaint of discomfort.

#### Patient reports:

Cravings 1/10; anxiety 1/10; depression 1/10; and hours of uninterrupted sleep 8.

#### Infusion 6:

Patient self-rates cravings 1/10, depression and anxiety 0/10. Patient denies myalgia. Patient reports getting 8 h of sleep, good appetite, drinking 40 – 60 oz of water. No GI complaints. Patient reports his energy levels, and his concentration has improved. Patient states he has a sponsor and is looking forward to making changes in his life. The patient was observed relaxing on couch during infusion. Patient c/o nausea after 10 min into PAWS support infusion. The patient was given ondansetron 4 mg 2tab SL without incidence. Patients reported alleviated symptoms after 5 min of medication administration. Patient completed and tolerated treatment well with no other complaints of discomfort.

#### Patient reports:

Cravings 1/10; anxiety 0/10; depression 0/10; and hours of uninterrupted sleep 8.

#### Infusion 7:

Patient is in a good mood today. The patient denies pain at the moment. Patient states that treatment has helped him physically and mentally. Patient states he eats 3 – 4 meals a day and drinks approx. 30 – 60 oz of water daily. Educated patient on importance of staying hydrated patient was receptive. Patient denies BM issues and states having a moderate activity level. Patients tolerated treatment well with no pain or discomfort.

#### Patient reports:

**Figure 24:**
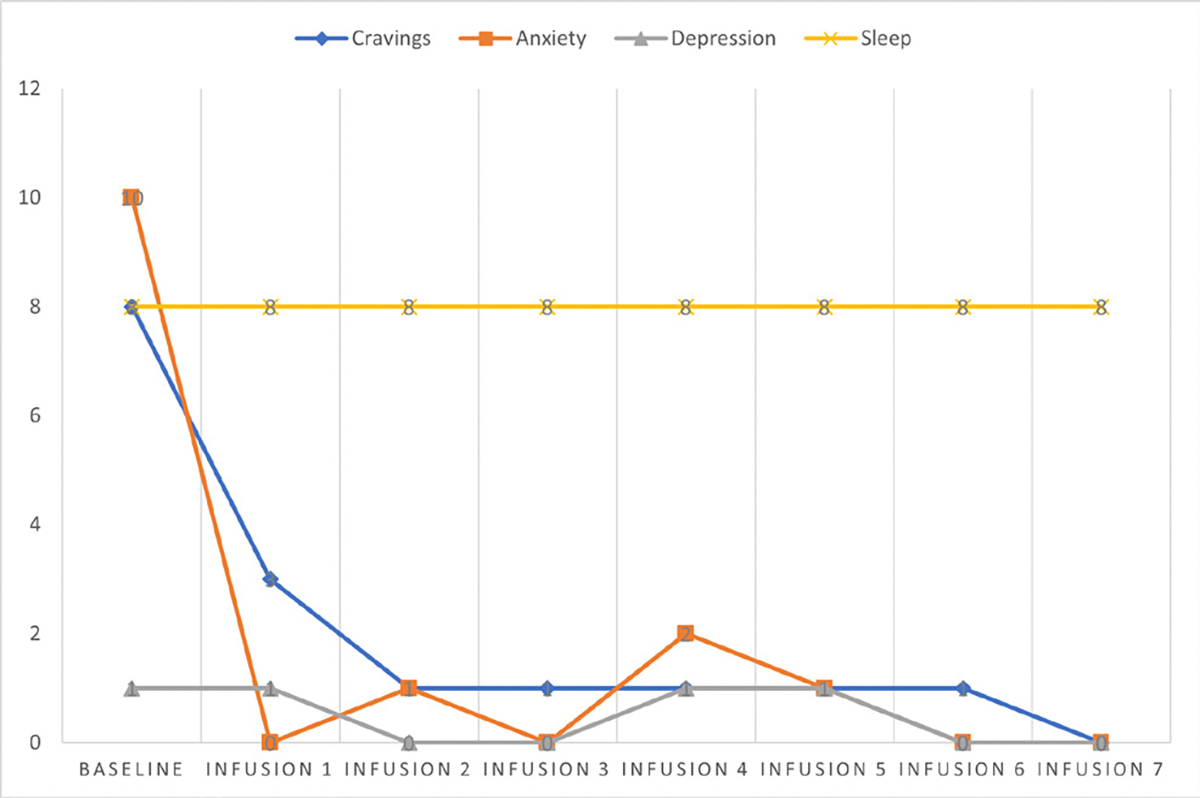
Patient 23 (Male/33/meth/ETOH).

Cravings 0/10; anxiety 0/10; depression 0/10; and hours of uninterrupted sleep 8 (Table 25 and figure 24).

### Patient 24 (Male/42 ETOH)

#### Consultation:

42-year-old male patient presents today for complaints of “Client presents to clinic for H&P for detox arriving yesterday afternoon from OK. PMH of asthma, mandibular reduction, psych hx of anxiety. Take albuterol inhaler, prozac 40 mg daily, testosterone 2 ml weekly. DOC is alcohol drinking 1/2 gallon daily, was hospitalized for 9 days for withdrawals, was having delirium tremors, heart palpitations and seizures. Was taking 1 mg 2x day for 1.5 years. My last drink was 6/2, and last Xanax was yesterday. The longest sobriety is 2 years in 2019. Started alcohol addiction at age 16. Has been to > 10 treatment facilities. Denies any chest pain, no SOB, no n/v/d. + tremors, no cold/hot sweats. Currently I have trouble concentrating and I know I’m dehydrated from all the alcohol. I do have a lot of tremors in my body from the residual alcohol withdrawal. Denies any OD and several seizures in the past from alcohol withdrawal.”

#### Baseline:

Blood drawn from right AC from IV catheter per practitioner’s order. 4SST 2LAV 1UA obtained and sent to Integritox. This is client’s first infusion, client expressed he has some anxiety, anxiety is rated at a 3 out of 10 because client is new. Client states he doesn’t like needles and feels nervous about infusion, anxiety decreased after IV insertion and blood draw. Client states, ‘it wasn’t bad’ IV site dry and intact, client tolerated infusion well.

#### Patient reports:

Cravings 5/10; anxiety 3/10; depression 0/10; and hours of uninterrupted sleep 7.

#### Infusion 1:

Client reports having a good weekend overall. Client did report he dislocated his right shoulder while playing with a dog, client admits it happens to him often dislocating his shoulder Client reports he currently feels soreness and some aches, but it is manageable. Client reports drinking about 64 oz of water a day, advised client to increase water intake, client expressed understanding. IV site dry and intact, client tolerated infusion well.

#### Patient reports:

Cravings 3/10; anxiety 2/10; depression 0/10; and hours of uninterrupted sleep 7.

#### Infusion 2:

Client reports having a good weekend overall. Client reports he currently feels soreness and some aches, but it is manageable. Client states today is the first day without the stabilizer client reports drinking about 64 oz of water a day, advised client to increase water intake, client expressed understanding. IV site dry and intact, client tolerated infusion well.

#### Patient reports:

Cravings 1/10; anxiety 2/10; depression 0/10; and hours of uninterrupted sleep 5.

#### Infusion 3:

Client said he noticed he is experiencing less tremors and improved mental clarity. The client admits that he is very spiritual to begin with and he meditates often, and it helps with any anxiety and depression he experiences. Client denies currently experiences with cravings, anxiety or depression/client drinks about 8 cups of water a day, advised client to increase water intake. IV site dry and intact, client tolerated infusion well.

#### Patient reports:

Cravings 0/10; anxiety 0/10; depression 0/10; and hours of uninterrupted sleep 7.

#### Practitioner FU:

Client presents to clinic for NAD follow up. Currently on infusion number 5. States “1. I have GAD but my anxiety seems to be at bay right now. 2. I have more energy I’ve noticed and 3. my mind seems to be clearer and able to sit through my programming better.”

#### Infusion 4:

Client denies current experiences with cravings, anxiety or depression/client drinks about 8 cups of water a day, advised client to increase water intake. Client expressed he noticed he is experiencing less tremors and improved mental clarity. The client admits that he is very spiritual to begin with and he meditates often, and it helps with any anxiety and depression he experiences. IV site dry and intact, client tolerated infusion well.

#### Patient reports:

Cravings 0/10; anxiety 0/10; depression 0/10; and hours of uninterrupted sleep 6.

#### Infusion 5:

Client reports his Right shoulder pain level is a 2 out of 10, client states it is tolerable and pain does not get in the way of his daily activities. Client denies cravings, anxiety and depression. Client admits he drinks about 8 cups of water a day, advised and educated client on importance of water intake and to increase water intake. Client expressed understanding. IV site dry and intact, client tolerated infusion well.

#### Patient reports:

Cravings 0/10; anxiety 0/10; depression 0/10; and hours of uninterrupted sleep 8.

#### Infusion 6:

Patient reports feeling good, sleep is okay at 6 – 7 h an evening of uninterrupted sleep. Patient claims he feels good level of energy after sleep. Pain is self-rated at a 2 out of 10. The patient dislocated his shoulder a several weeks back and surgery is needed, surgery scheduled for 09/18/2020. Patient denies cravings and depression. Anxiety is self-rated at a 2 out of 10 patients expressed that he used meditation and coping mechanisms, and it helps him. IV site dry and intact, patient tolerated infusion well.

#### Patient reports:

Cravings 0/10; anxiety 2/10; depression 0/10; and hours of uninterrupted sleep 7.

#### Infusion 7:

Patient comes into the office in a good mood. Patient self-rates his anxiety at 2/10 (situational), pain at 2/10 on his right shoulder, patient states he has had pain for years. Patient states eating 2 meals a day and drinking approx. 64 oz of water daily. Patient states no BM issues and has a low activity level. Patients tolerated treatment well with no pain or discomfort.

#### Patient reports:

**Figure 25:**
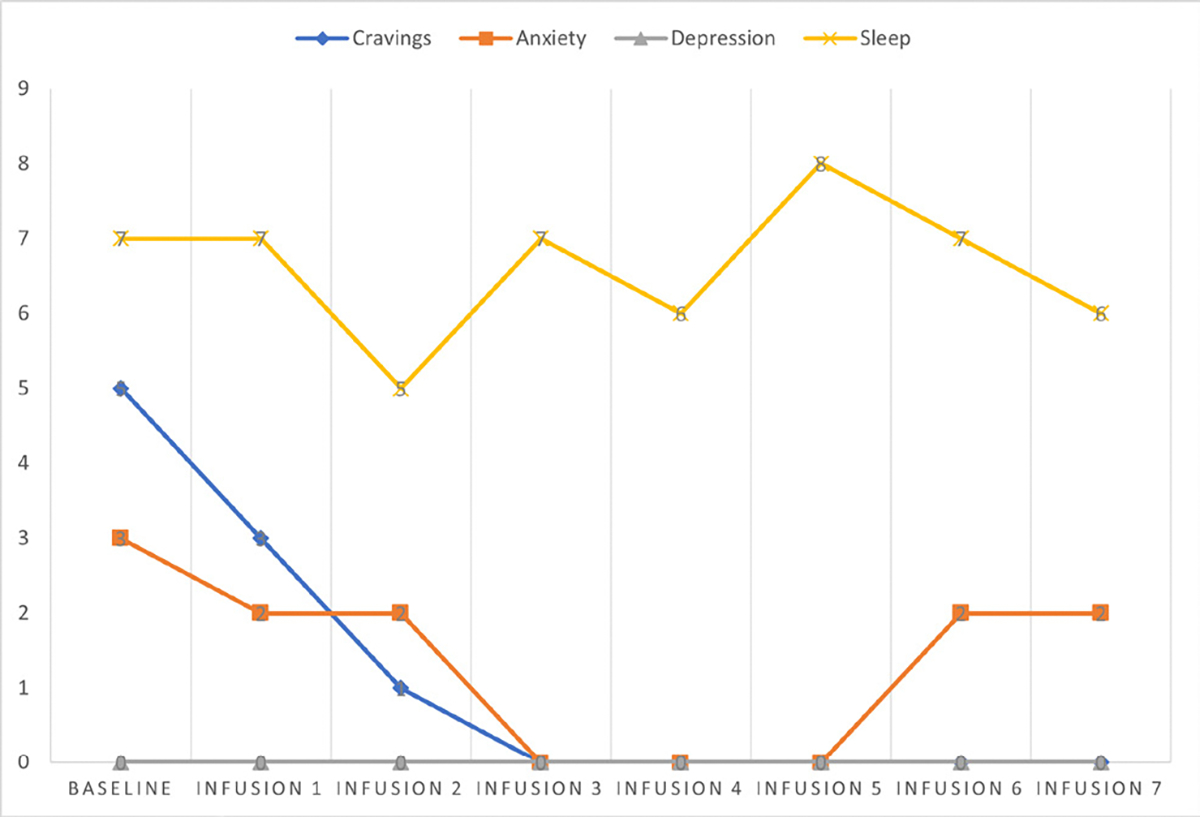
Patient 24 (Male/42/ETOH).

Cravings 0/10; anxiety 2/10; depression 0/10; and hours of uninterrupted sleep 6 (Table 26 and figure 25).

### Patient 25 (Male/33/meth)

#### Consultation:

33-year-old male patient presents today for complaints of DOC is meth via smoking, last use was 6/19, longest sobriety is 2.5 years, and this is clients 3rd treatment facility. Currently I have a hard time concentrating my whole life, that’s why I did meth so I can concentrate on one thing at a time. I have subtle anger. I want to rehydrate myself and get my insides good.

#### Baseline:

Patient reports to first infusion. Blood drawn from left AC from IV catheter per practitioner’s order. 4SST 1LAV 1UA obtained and sent to integritox. Patient self-rates anxiety and cravings 5/10, depression 2 – 3/10. Patient denies myalgia. Patient reports drinking 4 – 5 cups of water, good appetite and getting about 8 h of sleep and staying moderately active. Encouraged patients to increase fluid intake and educated them on the importance of proper hydration. patient was receptive. Patient was observed relaxing and conversing with peers during infusion. Patient tolerated treatment well with no complaint of discomfort.

#### Patient reports:

Cravings 5/10; anxiety 5/10; depression 3/10; and hours of uninterrupted sleep 8.

#### Infusion 1:

Patient self-rates cravings 0/10, anxiety 3/10, and depression 2/10. Patient denies myalgia. Patient reports feeling great after the first infusion. patient states he had restful sleep and woke up feeling great. The patient drinks 120+ oz of water and stays moderately active. The patient was observed relaxing and watching TV during infusion. Patient tolerated treatment well with no complaint of discomfort.

#### Patient reports:

Cravings 0/10; anxiety 3/10; depression 2/10; and hours of uninterrupted sleep 8.

#### Infusion 2:

Patient self-rates cravings 0/10, depression 2/10, and anxiety 3/10. Patient denies myalgia. Patient reports 8 h of uninterrupted sleep, good appetite and drinking 120 oz of water. Patient reports he feels amazing and with steady energy throughout the day. Patient reports he gets restful sleep but wakes up to use the restroom. Encouraged patients to avoid drinking water late in the evening before going to bed to avoid frequent awakening to use restroom. Patient was receptive. The patient was observed relaxing during infusion. Patient tolerated treatment well with no complaint of discomfort.

#### Patient reports:

Cravings 0/10; anxiety 3/10; depression 2/10; and hours of uninterrupted sleep 8.

#### Infusion 3:

Patient self-rates depression and anxiety 3/10 situational, cravings 0/10. Patient denies myalgia. Patient reports drinking 80 oz of water and sleeping 8 h. Patient reports feeling a difference in his energy and sleeping a lot better since he started infusions. The patient was observed relaxing during infusion. Patient tolerated treatment well with no complaint of discomfort.

#### Patient reports:

Cravings 0/10; anxiety 3/10; depression 3/10; and hours of uninterrupted sleep 8.

#### Practitioner FU:

Client presents to clinic for follow up on NAD. Currently on infusion 4 and states he has more energy and not getting tired throughout the day. “I have very little anxiety, and my depression feels like it’s gone. I’m also able to sleep better at night sleeping 8 h straight.”

#### Infusion 4:

Client expressed he is feeling good. Client denies cravings, or pain. The client admits he does have anxiety and depression. Client self-rated anxiety and depression at 2 out of 10, advised client to practice meditation and breathing techniques, client expressed understanding. IV site dry and intact, client tolerated infusion well.

#### Patient reports:

Cravings 0/10; anxiety 0/10; depression 0/10; and hours of uninterrupted sleep 8.

#### Infusion 5:

Client reports feeling good today. When asked what client is planning to do this weekend, client responded “I’m not here for a vacation” agreed with client but did tell client sometimes getting out and having nature walks/hike is beneficial and healing and client agreed. Client denies cravings and reports his anxiety and depression at a 2 – 3 out of 10. Advised client to meditate and practice breathing exercises. IV site dry and intact, client tolerated infusion well.

#### Patient reports:

Cravings 0/10; anxiety 2/10; depression 3/10; and hours of uninterrupted sleep 8.

#### Infusion 6:

Patient comes into the office for his 7^th^ treatment. Patient self-rates his depression at a level 2 out of 10. Patient denies pain. Patient states having a good appetite by eating 3 meals a day and drinking approx. 100 oz of water daily. Patient states having no BM issues and having slept for 9 h last night. Patients tolerated treatment well with no pain or discomfort.

#### Patient reports:

Cravings 0/10; anxiety 0/10; depression 2/10; and hours of uninterrupted sleep 9.

#### Infusion 7:

Patient states, “I feel good, I just got a tattoo yesterday, I’m happy” patient denies cravings and pain. Patient self-rates anxiety and depression at a 3 out of 10 and a 4 out of 10. Advised patient to practice meditation and breathing exercises, patient expressed understanding. IV site dry and intact, patient tolerated infusion well.

#### Patient reports:

**Figure 26:**
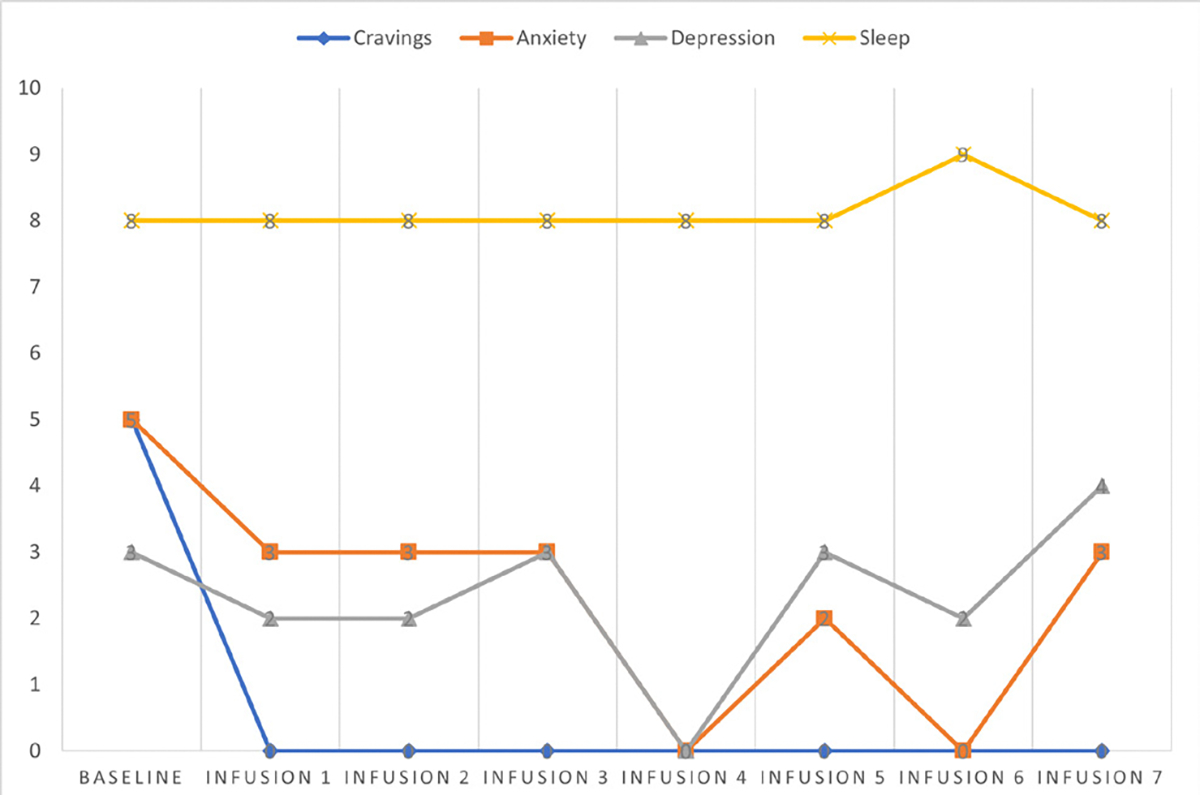
Patient 25 (Male/33/meth).

Cravings 0/10; anxiety 4/10; depression 3/10; and hours of uninterrupted sleep 8 (Table 27 and figure 26).

### Patient 26 (Male/35/heroin/opiates)

#### Consultation:

35-year-old male patient presents today for complaints of DOC is heroin via IV and opiate tabs, last use was 7/6, longest sobriety is 2 years, and has been to 1 other treatment facility. 3 OD in the past and no seizures. Currently “1. I have trouble sleeping, I sleep 3 – 6 h waking throughout the night with night sweats. 2. I have trouble concentrating, 3. I am irritable all the time and no anger 4. I also experience anxiety.”

#### Baseline:

Patient reports cravings and anxiety at a level 6 out of 10, no depression and pain at a level 2 out of 10 on his knees and pain is continuous. Recommended natural ways to help with anxiety such as yoga or meditation. Patient states having a good appetite by eating 3 meals a day and drinking 6 – 8 cups of water daily. No BM issues. Patient states having slept for 7 h last night. Patients tolerated treatment well with no pain or discomfort.

#### Patient reports:

Cravings 6/10; anxiety 1/10; depression 0/10; and hours of uninterrupted sleep 7.

#### Infusion 1:

Patient self-rates cravings, depression 0/10, anxiety 3/10. Patient denies myalgia. Patient reports drinking 32 oz of water and sleeping 7 h. Encouraged patient to increase water intake. Patient was receptive. Patient was observed relaxing during infusion and watching TV. Patient tolerated treatment well with no complaint of discomfort.

#### Patient reports:

Cravings 0/10; anxiety 2/10; depression 0/10; and hours of uninterrupted sleep 7.

#### Infusion 2:

Patient comes into the office for his treatment. Patient self-rates his cravings at a level 2 out of 10, depression at a level 3 out of 10 (situational), and no pain. Recommended natural ways to help with depression such as yoga or meditation. Patient reports having a good appetite by eating 3 meals a day and drinking approx. 32 oz of water daily. Patient reports no BM issues and has a moderate activity level. No med changes. Patients tolerated treatment well with no pain or discomfort.

#### Patient reports:

Cravings 2/10; anxiety 0/10; depression 3/10; and hours of uninterrupted sleep 7.

#### Infusion 3:

Patient self-rates cravings 2/10, anxiety 1/10, depression 1/10. Patient denies myalgia. Patient reports drinking 32 oz of water and sleeping 8 h. Encouraged patient to increase water intake and educated on the importance of proper hydration. Patient was receptive. Patient reports to notice a difference in his energy and his anxiety has improved. The patient was observed relaxing and watching TV during infusion. Patient tolerated treatment well with no complaint of discomfort.

#### Patient reports:

Cravings 2/10; anxiety 1/10; depression 1/10; hours of uninterrupted sleep 8.

#### Practitioner FU:

Client presents to clinic for NAD follow up. Currently on infusion 4, states he has had 3 NAD and 4^th^ Myers. “My sluggishness has improved and I’m more motivated when I wake up until I go to sleep. I look forward to all my infusions.”

#### Infusion 4:

Patient self-rates anxiety and cravings 3/10, and depression 1/10. Patient denies myalgia. Patient reports to be feeling tired and low in energy. Patient reports drinking 32 oz of water and sleeping 7 h. Encouraged patient to increase water intake and educated on the importance of proper hydration, particularly when exercising and being out in hot weather. Patient was receptive and expressed understanding. Patient was observed watching TV and conversing with peers during infusion. Patient tolerated treatment well with no complaint of discomfort.

#### Patient reports:

Cravings 3/10; anxiety 3/10; depression 1/10; and hours of uninterrupted sleep 7.

#### Infusion 5:

Patient self-rates cravings and depression 0/10, anxiety 2/10 situational. Patient denies myalgia. Patient reports good appetite, drinks 48 oz slept 7 h no GI complaints. Encouraged patients to increase their water intake. Patient was receptive. Patient reports he feels great with more energy and his mind is less foggy. The patient was observed relaxing listening to music during infusion. Patient tolerated treatment well with no complaint of discomfort.

#### Patient reports:

Cravings 0/10; anxiety 2/10; depression 0/10; and hours of uninterrupted sleep 7.

#### Infusion 6:

Patient self-rates cravings, anxiety and depression 0/10. Patient c/o of left shoulder pain from working out. Encouraged patients to warm up and stretch before exercise. Patient reports drinking 48 oz of water and sleeping 7 h. Patient reports feeling a bit tired today. Encouraged patients to increase their water intake. The patient was observed relaxing during infusion. Patient tolerated treatment well with no complaint of discomfort.

#### Patient reports:

Cravings 0/10; anxiety 0/10; depression 0/10; and hours of uninterrupted sleep 7.

#### Infusion 7:

Patient reports for infusion as ordered by the practitioner. Patient reports that he is getting a lot better sleep and feels rested in the morning. Patients also report better energy that is consistent throughout the day. Patient reports that his 0/10 cravings and 1/10 anxiety. That his anxiety is mostly situational, over a girl. Reports needing to decide and possibly end the relationship, so this does not continue to impact him negatively in the future. Patients tolerated infusions well and had no complaints of discomfort. Patient was observed relaxing in recliner and chatting with others during infusion. The patient completed the infusion and left stable.

#### Patient reports:

**Figure 27:**
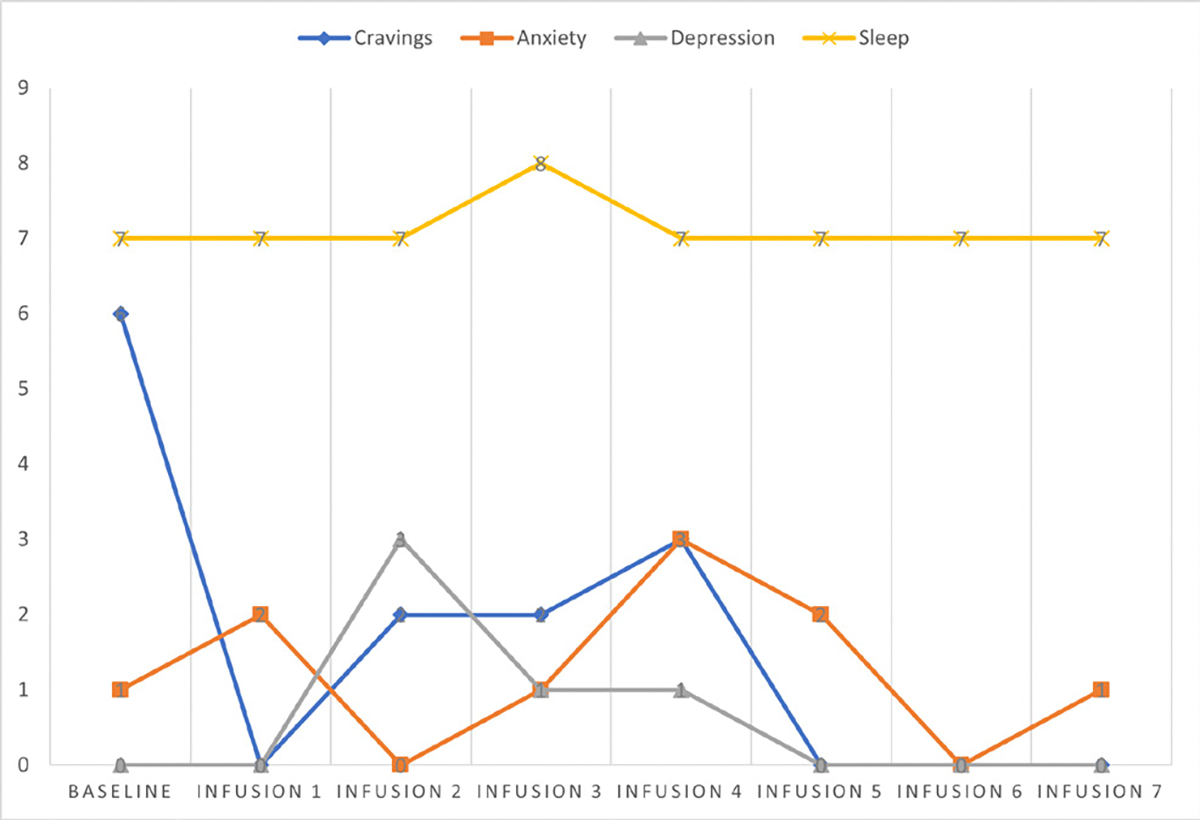
Patient 26 (Male/35/heroin/opiates).

Cravings 0/10; anxiety 1/10; depression 0/10; and hours of uninterrupted sleep 7 (Table 28 and figure 27).

### Patient 27 (Male/44/heroin/meth)

#### Consultation:

44-year-old male patient presents today for complaints of DOC is heroin and meth via smoking and muscle shots and snorting. Last use was 5/31. The longest sobriety is 7 years up until 2016. This is the client’s first inpatient facility. 1 OD in 2005 or seizures in the past. Currently “I have anxiety 7/10 daily, I sleep very little lately. My concentration is all over the place and it is hard to focus. Denies any cravings. I do get fatigued really easily.”

#### Baseline:

Blood drawn from right AC via IV catheter per practitioner’s order. 3SST 1LAV 1UA obtained and sent to integritox. Patient reports to first infusion with complaints of insomnia and fatigue. Patient states, “I get very little sleep recently and I wake up feeling tired” patient states he has 30 days sober and has had longer sobriety of 7 years. Patient is happy to get back on track for his health. The patient was observed cheerful and conversing with peers during infusion. Patient tolerated treatment well with no complaint of discomfort.

#### Patient reports:

Cravings 4/10; anxiety 7/10; depression 0/10; and hours of uninterrupted sleep 3.

#### Infusion 1:

Patient self-rates anxiety, depression, and cravings 0/10. Patient denies pain. Patient reports he has been keeping himself busy and had not made time to come to infusions. The patient reported he did feel more energy after his first infusion. Patient reports drinking 8 – 10 cups of water and staying active with a good appetite. Patient was observed relaxing and conversing with peers during infusion. Patient tolerated treatment well with no complaint of discomfort.

#### Patient reports:

Cravings 0/10; anxiety 0/10; depression 0/10; and hours of uninterrupted sleep 5.

#### Infusion 2:

Client reports sleep has been great at 7 h an evening. Client reports he sleeps through the night and if he were to wake up, he has no issues falling right back to sleep. Energy level has improved, client also admits he is now on a vegan diet and is actively working on his health and weight loss. Client drinks about 180 oz of water a day and is observed to be very focused. IV site dry and intact, client tolerated infusion well.

#### Patient reports:

Cravings 0/10; anxiety 1/10; depression 0/10; and hours of uninterrupted sleep 5.

#### Infusion 3:

Patient self-rates anxiety, cravings, and depression 0/10. Patient denies myalgia. Patient reports getting 8 h of uninterrupted sleep and maintain an active lifestyle. Patient reports feeling great and noticing a difference in his energy levels. patient reports to feel healthy and runs every day. Patient drinks about 180 oz of water daily and has a good appetite. The patient was observed to be cheerful and conversing with peers during infusion. Patient tolerated treatment well with no complaint of discomfort.

#### Patient reports:

Cravings 0/10; anxiety 0/10; depression 0/10; and hours of uninterrupted sleep 8.

#### Infusion 4:

Client reports he has been having high cravings to use heroin because his sister called him the other day and asked for money to purchase drugs, client refused and since that day he has been thinking of using. Client expressed that he discussed with case manager and therapist about his cravings and is currently trying to work through it. The client said he is no longer on vegan diet but is trying to eat healthy. Client drinks about 100 oz of water daily, advised client to increase water intake, client expressed understanding. IV site dry and intact, client tolerated infusion well.

#### Patient reports:

Cravings 8/10; anxiety 0/10; depression 0/10; and hours of uninterrupted sleep 8.

#### Infusion 5:

Patient self-rates anxiety, depression and cravings 0/10. Patient denies myalgia. Patient reports drinking 150 oz of water and sleeping 4 – 5 h. Good appetite, no GI issues. Patient states he maintains an active lifestyle and is happy to take care of his hepatitis C. Patient had blood drawn from Right ac via IV catheter per practitioner order. 4SST 3LAV 1 blue obtained and sent to lab. Patient reports he feels increased energy and an overall well-being. Patient was observed relaxing and talking with peers during infusion. Patient tolerated treatment well with no complaint of discomfort.

#### Patient reports:

Cravings 0/10; anxiety 0/10; depression 0/10; and hours of uninterrupted sleep 5.

#### Infusion 6:

Client denies any cravings or pain. Client states he is trying to refocus on himself, client was getting into relationships with women, and it wasn’t working out. Client expressed that he discussed with case manager and therapist about his cravings and is currently trying to work through it. The client said he is no longer on vegan diet but is trying to eat healthy. Client drinks about 100 ounces of water daily, advised client to increase water intake, client expressed understanding. IV site dry and intact, client tolerated infusion well.

#### Patient reports:

Cravings 0/10; anxiety 3/10; depression 3/10; and hours of uninterrupted sleep 8.

#### Infusion 7:

Client denies any cravings, anxiety, and depression or pain. The client said he is no longer on vegan diet but is trying to eat healthy. Client appears to be focused on his sobriety. The client was focusing more on girls the past several weeks but vocalized that he needs to “stay on track” client drinks about 100 oz of water daily, advised client to increase water intake, client expressed understanding. IV site dry and intact, client tolerated infusion well.

#### Patient reports:

**Figure 28:**
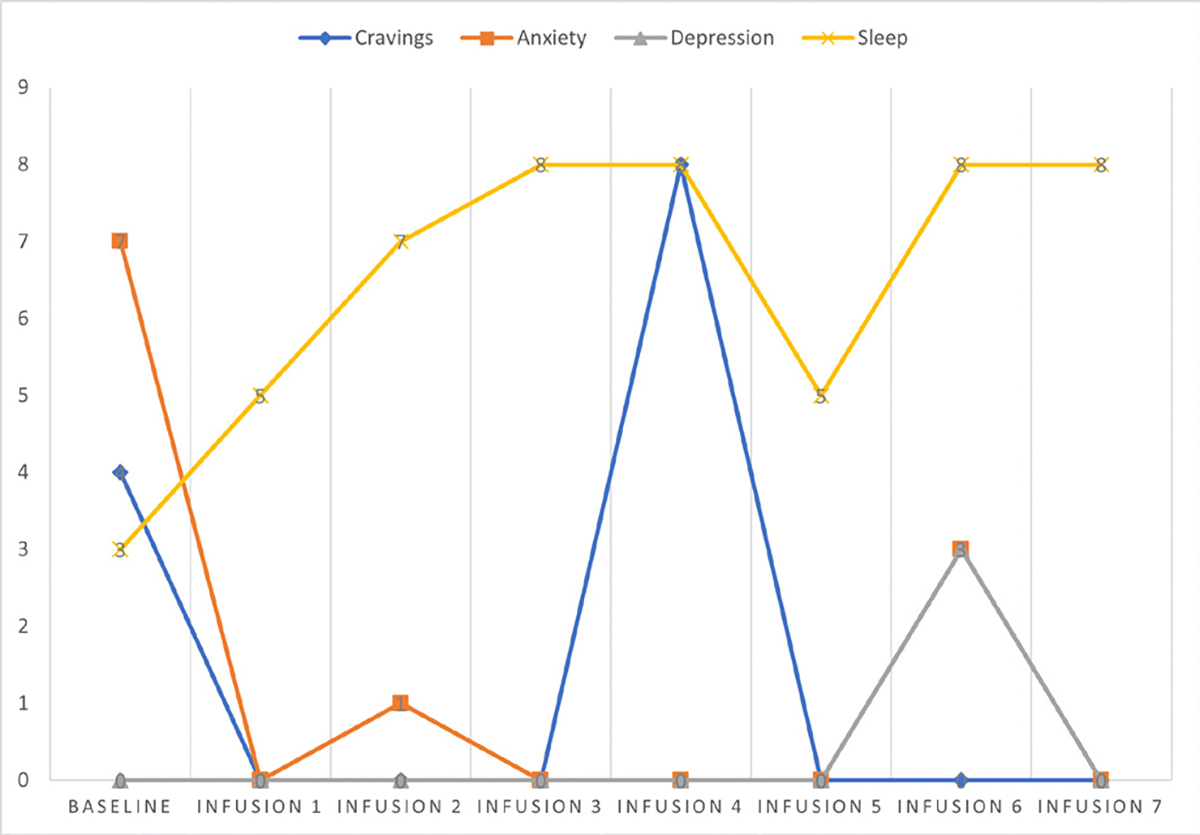
Patient 27 (Male/44/heroin/meth).

Cravings 0/10; anxiety 0/10; depression 0/10; and hours of uninterrupted sleep 8 (Table 29 and figure 28).

### Patient 28 (Male/40/meth)

#### Consultation:

40-year-old male patient presents today for complaints of DOC is meth via snorting and smoking, last use was 7/15, longest sobriety is 17 months, and this is clients first treatment facility. Denies any OD or seizures in the past. Currently with 1. fatigue, lack of energy, 2. anxiety 7/10 and 3. malnutrition/dehydration for the last 4 – 5 years. Had no appetite to eat due to depression and situational.

#### Baseline:

Blood draw from right ac via IV catheter per practitioner’s order. Obtained 4SST 1LAV 1UA and sent it to integritox. Patient self-rates cravings 4/10, anxiety 7/10 situational, depression 0/10. Patient c/o lower back pain 3/10 due to an old work injury in his 20s. Patient reports sleeping 6 h and drinking 8 – 10 cups of water. Encouraged patients to increase water intake and educated on the importance of proper hydration. Patient was receptive. Patient was observed relaxing and conversing with peers during infusion. Patient tolerated treatment well with no complaint of discomfort.

#### Patient reports:

Cravings 4/10; anxiety 7/10; depression 0/10; and hours of uninterrupted sleep 6.

#### Infusion 1:

Patient self-rates his cravings at a level 7 out of 10, anxiety, and depression at a level 7 out of 10 (situational), recommended of natural ways to help with anxiety and depression such as yoga or meditation. Patient reports having no pain. Patient reports having a good appetite eating 3 meals a day and drinking 8 – 10 cups of water. Patient reports having slept for 6 h last night and has a high activity level. Patients tolerated treatment well with no pain or discomfort.

#### Patient reports:

Cravings 7/10; anxiety 7/10; depression 7/10; and hours of uninterrupted sleep 6.

#### Infusion 2:

Patient self-rates cravings 7/10, anxiety 10/10 situational, and depression 0/10. Patient denies myalgia. Patient reports he feels anxious due to missing his baby daughter. Encouraged patients to stay positive and to practice meditation or breathing exercises to help decrease their anxiety. Patient was receptive. Patient reports drinking 8 – 9 cups of water and sleeping 6 – 7 h. Encouraged patient to increase water intake. Patient was observed conversing with peers during infusion. Patient tolerated treatment well with no complaint of discomfort.

#### Patient reports:

Cravings 7/10; anxiety 10/10; depression 0/10; and hours of uninterrupted sleep 7.

#### Infusion 3:

Patient comes into the office for his 4^th^ treatment. Patient self-rates his cravings, anxiety, depression and pain at a level 0 out of 10. Patient states having a good appetite by eating 3 meals a day and drinking approx. 64 oz of water daily. Patient reports no BM issues and has a high activity level. Patients tolerated treatment well with no pain or discomfort.

#### Patient reports:

Cravings 0/10; anxiety 0/10; depression 0/10; and hours of uninterrupted sleep 7.

#### Infusion 4:

Patient reports feeling good today. Patient appears to be in a good mood, good mood is evidenced by client joking around and talkative nature. Blood drawn from left AC from IV catheter per practitioner’s order. 4SST 2LAV obtained and sent to integritox. Patient denies cravings, depression and anxiety. Patients reports anxiety experienced at a 6 – 7 out of 10, advised patient to practice meditation and breathing exercises. Patient expressed understanding. IV site dry and intact, patient tolerated infusion well.

#### Patient reports:

Cravings 0/10; anxiety 6/10; depression 0/10; and hours of uninterrupted sleep 7.

#### Infusion 5:

Patient reports “improved cognitive function and mood is better” patient claims he normally experiences anger, but he has been working hard at how he reacts to situations and patient reports mood seems more stable. Patient denies cravings, depression or pain. A patient self-rates his anxiety at a 5 out of 10, advised patients to practice meditation and breathing exercises. IV site dry and intact, patient tolerated infusion well.

#### Patient reports:

Cravings 0/10; anxiety 5/10; depression 0/10; and hours of uninterrupted sleep 7.

#### Infusion 6:

Patient comes into the office in great spirits. Patient self-rates his anxiety at a level 4 out of 10 (situational), and patient denies pain. Recommended natural ways to help with anxiety such as yoga or meditation. Patient states having a good appetite by eating 3 meals a day and drinking approx. 64 oz of water daily. Patient states having no BM issues and having a high activity level. Patients tolerated treatment well with no pain or discomfort.

#### Patient reports:

Cravings 0/10; anxiety 4/10; depression 0/10; and hours of uninterrupted sleep 7.

#### Infusion 7:

Patient reports feeling good overall, patient says, “I slept good last night, I feel more energy.” Patient denies cravings, anxiety, depression and pain. The patient slept 8 h last night and feels rested. Patient reports water consumption at around 64 oz a day, educating client on importance of water intake, patient expressed understanding. IV site dry and intact, patient tolerated infusion well.

#### Patient reports:

**Figure 29:**
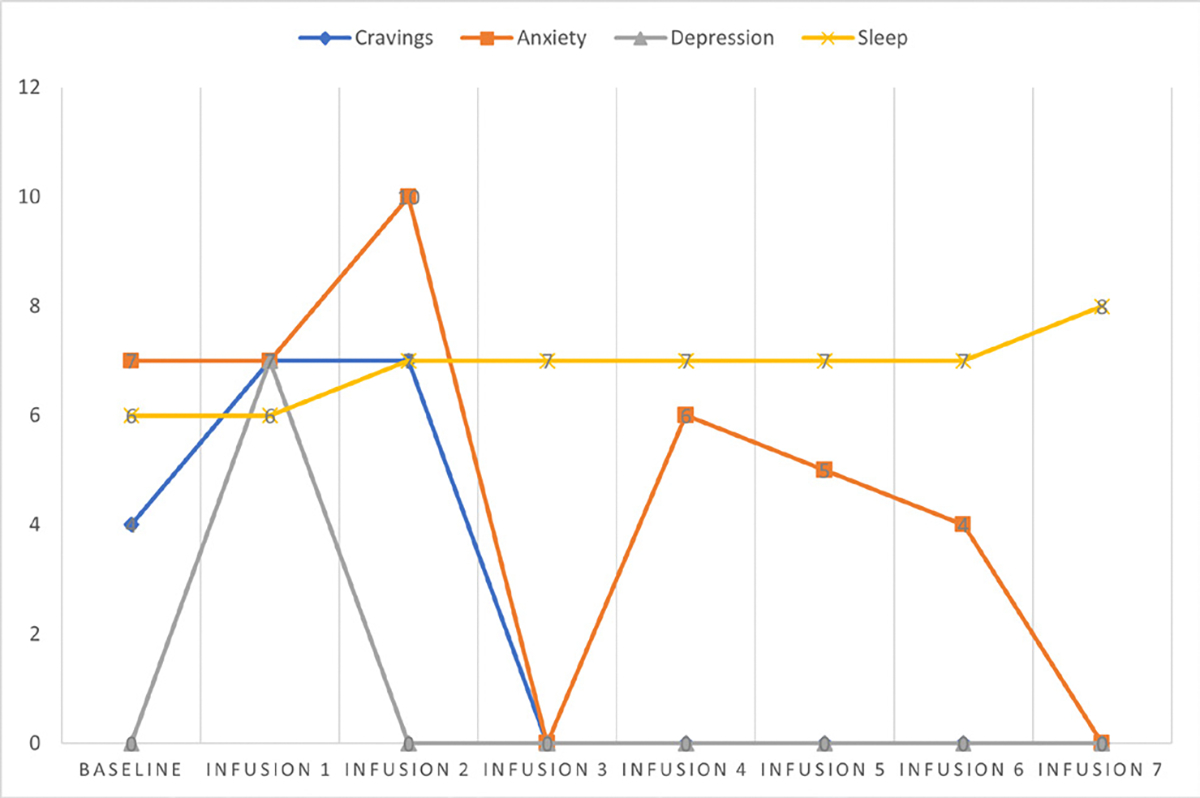
Patient 28 (Male/40/meth).

Cravings 0/10; anxiety 0/10; depression 0/10; and hours of uninterrupted sleep 8 (Table 30 and figure 29).

### Patient 29 (Male/36/meth/ETOH)

#### Consultation:

36-year-old male patient presents today for complaints of DOC is alcohol 1 pint daily, and meth via IV. Last use being 5/4/2020. The longest sobriety is 5 months since age 13. Has been to 3 treatment facilities. Denies any OD or seizures in the past. Currently “I have a lot of fatigue which I’ve always. I have a hard time concentrating all the time and with anxiety 8/10. I drink 24 oz of water daily and feel dehydrated.”

#### Baseline:

Patient reports to first infusion with c/o anxiety self-rated 4/10. Patient reports getting a full 8 h of uninterrupted sleep. Patient reports drinking minimal water. Encouraged patients to increase fluid intake and to practice meditation to help with anxiety. Patient was receptive. The patient was observed to be cheerful and conversing with peers during infusion. Patient tolerated treatment well with no complaint of discomfort. Patient completed treatment and left in stable condition.

#### Patient reports:

Cravings 5/10; anxiety 4/10; depression 0/10; and hours of uninterrupted sleep 8.

#### Infusion 1:

Patient self-rates cravings, depression, and anxiety 0/10. Patient denies pain. The patient reports a good appetite but has only been able to have liquid meals due to having his jaw wired because of a recent injury. Patient reports being frustrated with the limited options he has to eat. Educated patient on different alternatives he can try to meet his daily caloric intake. Patient was receptive. Patient reports getting back on track after relapsing and going through the incident that led to his injury. Patient reports having diarrhea r/t not being able to eat solid food. The patient was observed relaxing and watching TV during infusion. Patient tolerated treatment well with no complaint of discomfort.

#### Patient reports:

Cravings 0/10; anxiety 0/10; depression 0/10; and hours of uninterrupted sleep 8.

#### Infusion 2:

Patient self-rates anxiety, depression, cravings 0/10. Patient denies myalgia. Patient reports 8 h of uninterrupted sleep and drinking no water. Patient reports it’s hard for him to drink and eat with his jaw being wired down. Patient did report he was able to eat more today. He is excited to get the wires removed in 2 weeks. Patient reports he feels less tired during the day since he started the infusions. The patient was observed relaxing and watching TV during infusion. Patient tolerated treatment well with no complaint of discomfort.

#### Patient reports:

Cravings 0/10; anxiety 0/10; depression 0/10; and hours of uninterrupted sleep 8.

#### Infusion 3:

Patient self-rates anxiety, cravings, and depression 0/10. The patient denies pain but does state he has discomfort in his jaw due to it being wired shut. Patient c/o diarrhea due to only being able to have fluids. Patient reports sleeping 8 h and drinking 60 oz water. Patient reports he feels great, and he feels rested in the mornings. The patient was observed relaxing watching TV during infusion. Patient tolerated treatment well with no complaint of discomfort.

#### Patient reports:

Cravings 0/10; anxiety 0/10; depression 0/10; and hours of uninterrupted sleep 8.

#### Practitioner FU:

Client presents to clinic for NAD follow up. Currently on infusion 4. States he has a significant improvement with energy and not falling asleep during group, “I have been able to focus and take in a lot of the information that is given to me. I still have a little anxiety, but I feel like I’m naturally an anxious person. I also have noticed I haven’t had any cravings for drugs.”

#### Infusion 4:

Client reports he feels good today, client had a good weekend, he went to the beach and client appears to be in a good mood. Good mood is evidenced by client’s willingness to engage in conversations and joking around. Patient self-rates anxiety, cravings, and depression 0/10. The patient denies pain but does state he has discomfort in his jaw due to it being wired shut. IV site dry and intact, client tolerated infusion well.

#### Patient reports:

Cravings 0/10; anxiety 0/10; depression 0/10; and hours of uninterrupted sleep 8.

#### Infusion 5:

Client denies any cravings and denies pain. Client reports he feels good today, client had a good weekend, he went to the beach and client appears to be in a good mood. A good mood is evidenced by a client’s willingness to engage in conversations and joking around. IV site dry and intact, client tolerated infusion well.

#### Patient reports:

Cravings 0/10; anxiety 2/10; depression 2/10; and hours of uninterrupted sleep 8.

#### Infusion 6:

Client reports he feels good today, client had a good weekend, he went to the beach and client appears to be in a good mood. A good mood is evidenced by a client’s willingness to engage in conversations and joking around. The client denies any cravings and denies pain. Client reports he will be removing the wires in his mouth soon and he’s excited. IV site dry and intact, client tolerated infusion well.

#### Patient reports:

Cravings 0/10; anxiety 0/10; depression 0/10; and hours of uninterrupted sleep 8.

#### Infusion 7:

Client reports he feels good today, client had a good weekend, he went to the beach and client appears to be in a good mood. A good mood is evidenced by a client’s willingness to engage in conversations and joking around. Client denies cravings, depression or pain. Client drinks about 30 oz of water a day, advised client to increase water intake and to incorporate at home workouts since client expressed, he has not been very active at home. Client expressed agreement. IV site dry and intact, client tolerated infusion well.

#### Patient reports:

**Figure 30:**
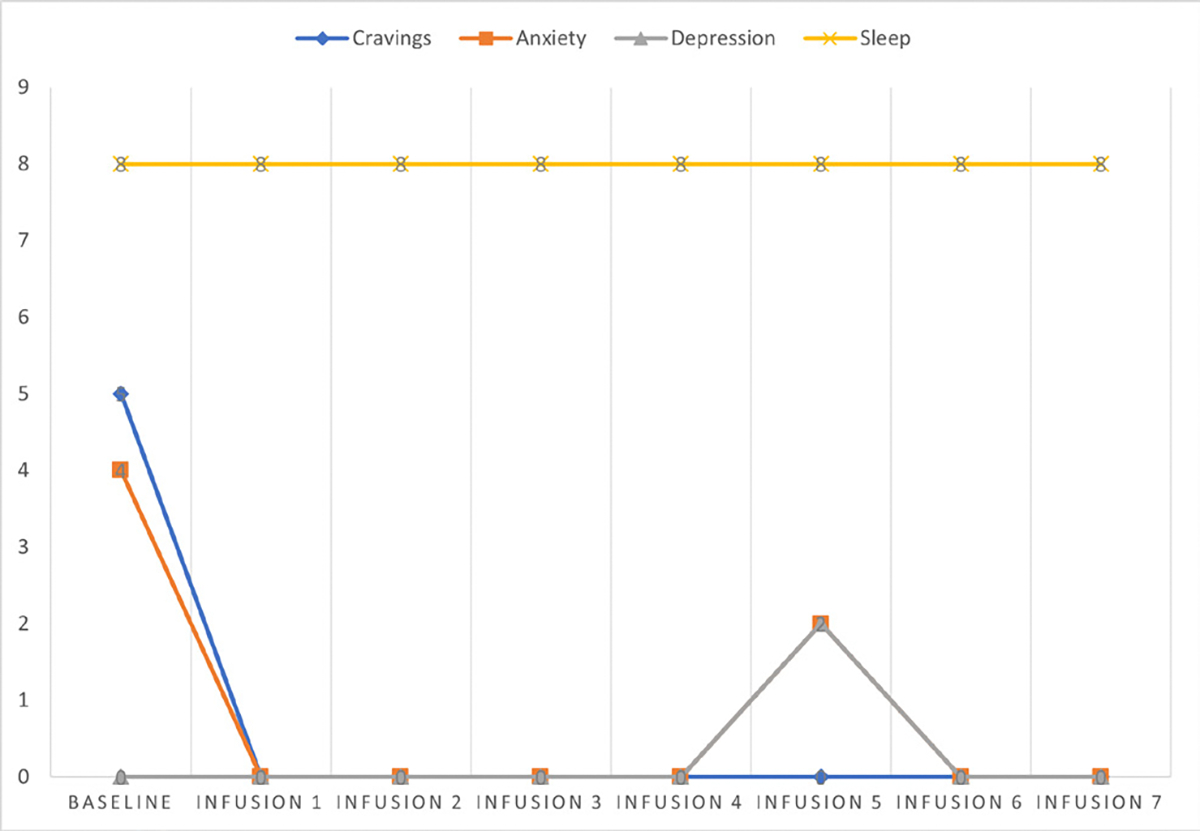
Patient 29 (Male/36/meth/ETOH).

Cravings 0/10; anxiety 0/10; depression 0/10; and hours of uninterrupted sleep 8 (Table 31 and figure 30).

### Patient 30 (Male/meth/ETOH)

#### Consultation:

32-year-old male patient presents today for complaints of “anxiety, depression, and dehydration”. Patient has a history of drug and alcohol abuse. Patient has a history of methamphetamine abuse started using at 16 years old. Last use was October 7^th^. His longest sobriety was 1 year, however, states he just relapsed in May. Patient also has a history of alcohol abuse, states he used to drink 5xs a week. His drink of choice was whiskey. Patient states his last drink was October 6^th^. His longest sobriety was around 1 year. Patient used to smoke tobacco, but quit 2 days ago, currently uses patches and lozenges. Patient is currently in rehabiliation.

#### Baseline:

Patient had blood drawn from left ac via IV catheter per practitioners’ order. 4SST 1LAV 1UA obtained and sent to integritox. Patient self-rates cravings and anxiety 1/10, depression 3/10. Patient denies myalgia. Patient reports good appetite, water intake 16 oz, sleep 6 h, moderate activity level. last BM 10/27. Encouraged patients to increase water intake and educated on the importance of proper hydration. Advised patient to increase fiber in his diet to help aid with constipation. Patient was receptive. Patients report irregular sleep and frequently feeling tired. The patient was observed relaxing during infusion. Patient tolerated treatment well with no complaint of discomfort.

#### Patient reports:

Cravings 1/10; anxiety 1/10; depression 3/10; and hours of uninterrupted sleep 6.

#### Infusion 1:

Patient self-rates his cravings, anxiety and depression at 1/10 today, patient denies pain at the moment. Patient states he eats 3 meals a day and drinks approx. 32 oz of water daily. Educated patient on importance of staying hydrated, patient was receptive. Patient denies BM issues and states having a moderate activity level. Patients tolerated treatment well with no pain or discomfort.

#### Patient reports:

Cravings 1/10; anxiety 1/10; depression 1/10; and hours of uninterrupted sleep 7.

#### Infusion 2:

Patient self-rates anxiety 6/10, cravings and depression 0/10. Patient denies myalgia. Patient reports 4 h of sleep, water intake 24 oz, moderate activity level, good appetite. Patient reports feeling unrested and tired all day. Encouraged patients to increase water intake and to try light exercise in the evenings to help with sleep. Patient reports to be more active. Patient was observed relaxing and conversing with peers during infusion. Patient tolerated treatment well with no complaint of discomfort.

#### Patient reports:

Cravings 0/10; anxiety 6/10; depression 0/10; and hours of uninterrupted sleep 4.

#### Infusion 3:

Patient seen by NP Salma Nawabi for follow up. The patient self-rates his anxiety today at 5/10, and patient denies pain. Patient reports eating 3 meals a day and rinks approx. 24 – 36 oz of water a day. Educated patient on importance of staying hydrated, patient was receptive. Patient denies BM issues and reports having a moderate activity level. Patients tolerated treatment well with no pain or discomfort.

#### Patient reports:

Cravings 0/10; anxiety 5/10; depression 0/10; and hours of uninterrupted sleep 5.

#### Practitioner FU:

Client presents to clinic for follow up of infusion. Currently on infusion 4. States “I don’t feel any negative effects, I feel really good when I wake up and have energy throughout the day. No anxiety today 0/10.”

#### Infusion 4:

Patient self-rates anxiety, depression and cravings 0/10. Patient denies myalgia. Patient reports to be feeling good and focusing on his recovery. Patient reports good appetite, moderate activity level, water intake 30 oz, no GI complaints. Encouraged patients to increase water intake and reminded him of the benefits of proper hydration. Patient was receptive. Patient was observed relaxing on recliner and conversing with peers during infusion. Patient tolerated treatment well with no complaint of discomfort.

#### Patient reports:

Cravings 0/10; anxiety 0/10; depression 0/10; and hours of uninterrupted sleep 8.

#### Infusion 5:

Patient self-rates his cravings, anxiety, depression, and pain at 0/10 today. Patient states he eats 3 meals a day and drinks approx. 24 oz of water a day. Patient denies BM issues and reports having a moderate activity level. Patients tolerated treatment well with no pain or discomfort.

#### Patient reports:

Cravings 0/10; anxiety 0/10; depression 0/10; and hours of uninterrupted sleep 6.

#### Infusion 6:

Patient reports sleep at 6 h of uninterrupted sleep an evening. Patient states he feels tired after sleep. Advised patients to practice meditation and breathing exercises to help promote better sleep, patient expressed understanding. Water intake is fair at 24 oz of water a day, advised and educated patients to increase water intake and importance of hydration. Patient is observed relaxed incliner eating his lunch. IV site dry and intact, he tolerated infusion well.

#### Patient reports:

Cravings 0/10; anxiety 0/10; depression 0/10; and hours of uninterrupted sleep 6.

#### Infusion 7:

Patient reports he feels good overall. Mental clarity is better because he is able to retain information and think more clearly. Patient denies cravings, anxiety depression and pain. Water intake is improving but overall poor at 24 oz of water. Advised patient to increase water intake especially if he is active. IV site dry and intact, he tolerated infusion well.

#### Patient reports:

**Figure 31:**
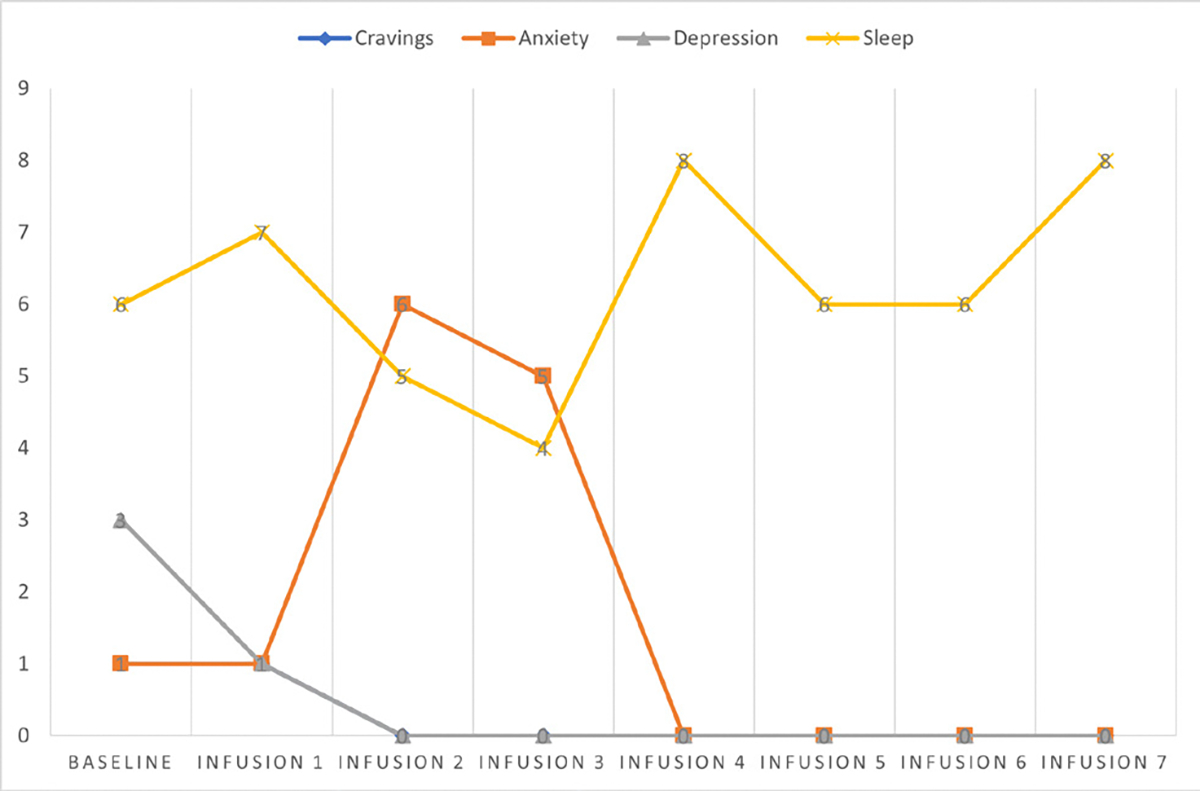
Patient 30 (Male/meth/ETOH).

Cravings 0/10; anxiety 0/10; depression 0/10; and hours of uninterrupted sleep 8 (Table 32 and figure 31).

### Patient 31 (Male/28/meth/heroin)

#### Consultation:

28-year-old male patient presents today for complaints of DOC is meth and heroin via IV 2 g daily, last use was 5/7. The longest sobriety is 100 days, and this is clients second treatment facility. States 1 OD in the past and no seizures in the past. Currently “I am irritable all the time and have a really hard time sleeping and I have no energy. I also am nauseated and dehydrated daily.”

#### Baseline:

Patient reports to first infusion with complaints of high anxiety and restless sleep. The patient reports high cravings and does have 2 weeks sober. The patient was observed relaxing during infusion. Patient tolerated treatment well with no complaint of discomfort. The patient completed treatment and was left in stable condition.

#### Patient reports:

Cravings 10/10; Anxiety 4/10; depression 0/10; and hours of uninterrupted sleep 6.

#### Infusion 1:

Patient reports to infusion. Patient reports drinking 48 oz of water and getting 8 h of uninterrupted sleep. Patient reports no anxiety. The patient was observed to be cheerful and conversing with his peers during infusion. Patient tolerated treatment well with no complaint of discomfort. The patient completed treatment and was left in stable condition.

#### Patient reports:

Cravings 6/10; anxiety 2/10; depression 0/10; and hours of uninterrupted sleep 8.

#### Infusion 2:

Client expressed that needles are a “huge trigger” for him, so he is very nervous. Client appears to have a lot of large veins that are usable, clients prefer his left AC because that is what’s “comfortable” client admits his anxiety is around an 8 out of 10, advised client to meditate and use breathing exercises during times of stress, client expressed understanding. Anxiety decreased to a 2 out of 10 after IV insertion. IV site dry and intact, client tolerated infusion well.

#### Patient reports:

Cravings 0/10; anxiety 8/10; depression 0/10; and hours of uninterrupted sleep 8.

#### Infusion 3:

Client states he feels great. Client reports that he does not experience any cravings for drugs or alcohol, client states he feels spiritually fit and is happy to say he is one of the main people who hosts meetings at least 3 times a week for his peers. Client states being able to be responsible for something allows him to focus on helping others which also helps himself and reminds him to stay focused. IV site dry and intact, client tolerated infusion well.

#### Patient reports:

Cravings 0/10; anxiety 0/10; depression 0/10; and hours of uninterrupted sleep 8.

#### Infusion 4:

Patient self-rates anxiety, depression, cravings 0/10. Patient denies pain. Patient reports feeling great and with a lot of energy. Patient states he is happy with his recovery and has started meetings with his peers. Patient states he drinks a liter of water daily and about 6 h of sleep. Patient was observed relaxing on recliner and conversing with peers during infusion. Patient tolerated treatment well with no complaint of discomfort.

#### Patient reports:

Cravings 0/10; anxiety 0/10; depression 0/10; and hours of uninterrupted sleep 6.

#### Infusion 5:

Patient self-rates anxiety, cravings, and depression 0/10. Patient denies any myalgia. Patient reports he feels great with infusions. Patient states he feels more energy and he is always in a good mood. Patient reports drinking 1 L of water and living a highly active lifestyle. Patient reports no GI problems. Patient was observed relaxing on couch and conversing with peers during infusion. Patient tolerated treatment well with no complaint of discomfort.

#### Patient reports:

Cravings 0/10; anxiety 0/10; depression 0/10; and hours of uninterrupted sleep 5.

#### Infusion 6:

Client reports feeling good, “I feel refreshed” client admits he does not drink too much water so receiving infusions has made him feel better because he feels like he’s hydrating himself. Advised client to increase water intake and educated client on the importance of water intake. IV site dry and intact, client tolerated infusion well.

#### Patient reports:

Cravings 0/10; anxiety 0/10; depression 0/10; and hours of uninterrupted sleep 6.

#### Infusion 7:

Client states he feels good. Client does not have cravings. Client did admit to not feeling his best, client hinted that it might be because he lost focus on his sobriety for a minute due to receiving money and spending it and choosing to use his time to go out and hang out rather than attend meetings and working on himself and the steps. The client is working with his sponsor and trying to do better. IV site dry and intact, client tolerated infusion well.

#### Patient reports:

**Figure 32:**
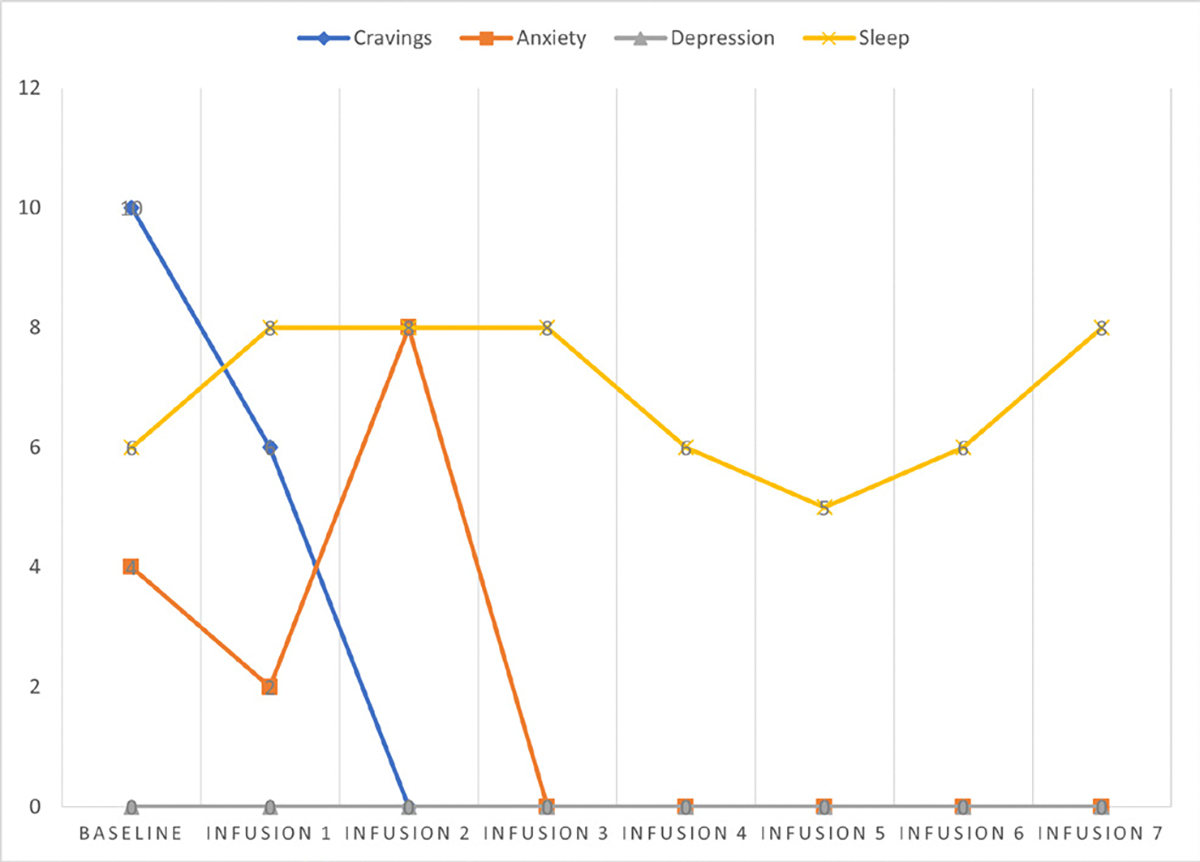
Patient 31 (Male/28/meth/heroin).

Cravings 0/10; anxiety 0/10; depression 0/10; and hours of uninterrupted sleep 8 (Table 33 and figure 32).

### Patient 32 (Male 23/heroin/ETOH/crack)

#### Consultation:

23-year-old male patient presents today for complaints of DOC is heroin via IV 1 g daily, crack and alcohol 1/2 of 1.5 daily. Last use was 50 days ago, and the longest sobriety is 1 year. This is the client’s 5^th^ treatment facility. Currently states “I have very little energy without the crack, I’m super irritable coming off of alcohol and I really have a hard time concentrating, also don’t drink any water.”

#### Baseline:

Blood drawn from left AC from IV catheter per practitioner’s order. 4SST 1LAV 1UA obtained and sent to integritox. Patient reports to first infusion with c/o restless sleep. Patient self-rates anxiety, cravings and depression to be 5/10. Encouraged patients to practice meditation and breathing exercises to help ease anxiety. Encouraged patient to talk to his treatment team and therapist. Patient was receptive. Patient reports drinking about 5 – 8 cups of water daily, encouraged patients to increase water intake. Patient was observed relaxing and conversing with peers during infusion. Patient tolerated treatment well with no complaint of discomfort.

#### Patient reports:

Cravings 5/10; anxiety 5/10; depression 5/10; and hours of uninterrupted sleep 5.

#### Infusion 1:

Patient reports cravings at a level 4 out of 10, anxiety at a level 3 out of 10, depression at a level 6 out of 10 and no pain. Patient comes in today for his 2^nd^ treatment c/o an upset stomach. Patient has not had a meal today only a snack. Will be administering ZOFRAN ODT after MYERS before NAD treatment. Recommended natural ways to help with anxiety and depression such as yoga or meditation. Patient states having a good appetite otherwise from having an upset stomach. Patient states drinking approx. 8 cups of water daily. Patient states no BM issues. Patient states having slept for 5 h last night. Patient states have a moderate activity level. Patients tolerated treatment well with no pain or discomfort.

#### Patient reports:

Cravings 4/10; anxiety 3/10; depression 6/10; and hours of uninterrupted sleep 5.

#### Infusion 2:

Patient self-rates cravings, depression and anxiety 4/10. Patient c/o of lower back pain 2/0 off and on. Patient reports to have slept really well after yesterday’s infusion and is looking forward to sleeping in on the weekend. Patient reports drinking 8 oz of water and sleeping 7 h. Encouraged patient to increase water intake and educated on the importance of proper hydration. Patient was receptive. The patient was observed relaxing during infusion. Patient tolerated treatment well with no complaint of discomfort.

#### Patient reports:

Cravings 4/10; anxiety 4/10; depression 4/10; and hours of uninterrupted sleep 6.

#### Infusion 3:

Client reports feelings okay today. Client says he’s been doing pretty well on the infusions and didn’t feel nausea last infusion. Client admits he is experiencing cravings, anxiety and depression. Cravings are self-rated at a 4 out of 10. Anxiety and depression are self-rated at a 3 out of 10 and a 4 out of 10 respectively. Advised client to practice meditation and breathing techniques and to discuss feelings with treatment team, client expressed understanding. IV site dry and intact, client tolerated infusion well.

#### Patient reports:

Cravings 4/10; anxiety 3/10; depression 4/10; and hours of uninterrupted sleep 5.

#### Infusion 4:

Client reports he feels okay today. Client says he tolerated infusion fine last time. Client admits he is experiencing cravings, anxiety and depression currently. Client denies pain. The client self-rated his cravings at an 8 out of 10. Client reports anxiety and depression at a self-rated 6 out of 10 and a 5 out of 10 respectively. Advised client to practice meditation and breathing techniques and to discuss with case manager and therapist, client expressed understanding. IV site dry and intact, client tolerated infusion well.

#### Patient reports:

Cravings 8/10; anxiety 6/10; depression 5/10; and hours of uninterrupted sleep 4.

#### Infusion 5:

Client reports doing well overall, but he has anxiety due to housemate situations. The client self-rated his anxiety at a 6 out of 10, client says his roommate was gone for almost a week due to a relapse now he’s returning, and the roommate’s relapse is what is giving him anxiety. Client states he has already discussed it with his treatment team. IV site dry and intact, client tolerated infusion well.

#### Patient reports:

Cravings 2/10; anxiety 6/10; depression 5/10; and hours of uninterrupted sleep 7.

#### Infusion 6:

Patient reports feeling okay, “my knuckles hurt, on and off, they are dry right now” advised patient to see physician if an emollient is recommended for him, patient expressed understanding. Patients say he stays hydrated by drinking about 120 oz of water a day. IV site dry and intact, patient tolerated infusion well.

#### Patient reports:

Cravings 0/10; anxiety 0/10; depression 2/10; and hours of uninterrupted sleep 7.

#### Infusion 7:

Patient reports sleep is okay at 7 h an evening, but he wakes up sometimes, advised patient to avoid liquids close to bedtime to avoid using the restroom in the middle of the night and to practice meditation. Water intake is about 32 oz a day, educated patients on importance of water intake and advised patients to increase water intake, he expressed understanding. IV site dry and intact, patient tolerated infusion well.

#### Patient reports:

**Figure 33:**
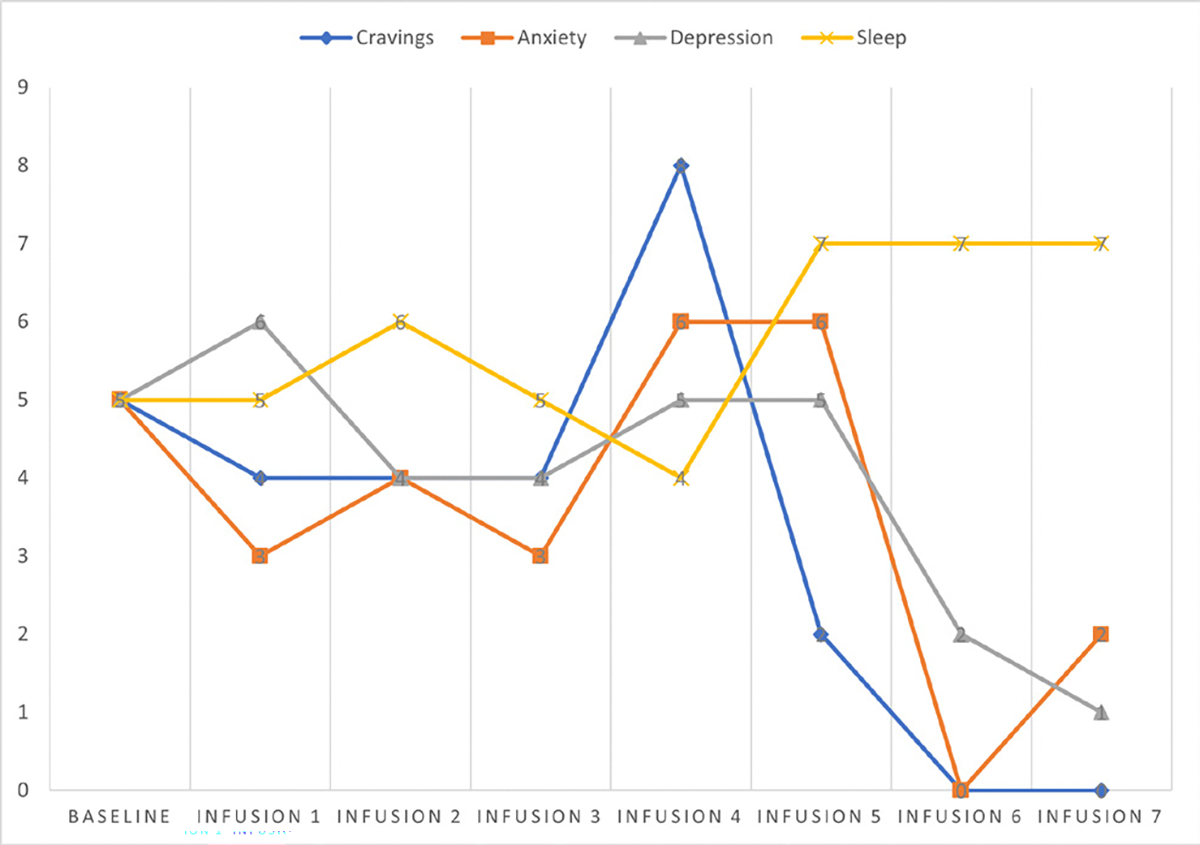
Patient 32: (Male/23/heroin/ETOH/crack).

Cravings 0/10; anxiety 2/10; depression 1/10; and hours of uninterrupted sleep 7 (Table 34 and figure 33).

### Patient 33 (Male/31/meth)

#### Consultation:

31-year-old male patient presents today for complaints of Drug use: meth. He injects or smokes 1 – 2 g meth per day. He usually injects. He last injected yesterday at about 12 noon. He has never been to treatment. He has been using meth since he was 15. By 16, he used it every day. The longest he has been clean was a month at a time when he was in jail. He also uses MJ and Xanax 2 nights ago, he took some Xanax, but this is not something he uses often. He only took it because he was anxious about coming to detox. Currently “I have a lot of irritability and anger my whole life, and it bothers me because I don’t want to be an angry person. I have anxiety when I am around people. I know I don’t eat well and drink water so I’m so excited to turn my life around, I’m doing this on my own and don’t know what I’m like sober.”

#### Baseline:

Blood drawn from right AC from IV catheter per practitioner’s order. 4SST 1LAV 1UA obtained and sent to Integritox. Client reports this is his first infusion, client expressed that he is in treatment for the first time, and he is grateful for the opportunity. Client states sleep is okay, but he wakes up frequently and has a tough time falling back to sleep. Advised client to practice meditation and breathing exercises. IV site dry and intact, client tolerated infusion well.

#### Patient reports:

Cravings 7/10; anxiety 3/10; depression 0/10; and hours of uninterrupted sleep 8.

#### Infusion 1:

Client reports feeling so happy to be in California. The client appears to be grateful for the opportunity to be in a treatment and work on his sobriety. Client reports he is still new, so he does still feel anxiety. Anxiety is rated at a 6 out of 10, advises clients to practice breathing techniques and grounding exercises. IV site dry and intact, client tolerated infusion well.

#### Patient reports:

Cravings 3/10; anxiety 6/10; depression 0/10; and hours of uninterrupted sleep 7.

#### Infusion 2:

Client reports he is still new, so he does still feel anxiety. Anxiety is rated at a 6 out of 10, advises clients to practice breathing techniques and grounding exercises. Client continues to show gratitude for being in treatment and excitement to be sober. Client reports feeling so happy to be in California. The client appears to be grateful for the opportunity to be in a treatment and work on his sobriety. IV site dry and intact, client tolerated infusion well.

#### Patient reports:

Cravings 1/10; anxiety 6/10; depression 4/10; and hours of uninterrupted sleep 8.

#### Infusion 3:

Client self-rated his anxiety at a 6 out of 10, advised client to practice breathing techniques and grounding exercises. Client continues to show gratitude for being in treatment and excitement to be sober. Client reports feeling so happy to be in California. The client appears to be grateful for the opportunity to be in a treatment and work on his sobriety. IV site dry and intact, client tolerated infusion well.

#### Patient reports:

Cravings 0/10; anxiety 6/10; depression 3/10; and hours of uninterrupted sleep 5.

#### Infusion 4:

Client reports he isn’t doing well today, client had dream about his father (it was a good) client reports his father passed away a little while ago but it made client sad. Client states his niece is also going into surgery for her lung cancer and it made him sad. The client recognizes it is okay to be sad but it’s good when he can talk about it and receive feedback. IV site dry and intact, client tolerated infusion well.

#### Patient reports:

Cravings 0/10; anxiety 6/10; depression 8/10; and hours of uninterrupted sleep 4.

#### Practitioner FU:

Client presents to clinic for NAD follow up, currently on infusion 5. States “I have been able to sit in grieving groups and talk about my dad which I have never done in my life, 1. It felt so good to cry and get my emotions out for the first time. 2. my skin feels so much better, 3. my parents have noticed I am calmer when I talk to them, I feel so much better about my anger already.”

#### Infusion 5:

Client reports feeling better today. The client expresses gratitude for being in the program and for the fortune of receiving infusion therapy. Client denies cravings. Client admits his anxiety and depression is around a 5 out of 10, client says he works through his feelings by meditating and breathing, client admits anxiety is normally a 9 out of 10 prior to meditation. Advised client to discuss with case manager and therapist, client expressed understanding. IV site dry and intact. client tolerated infusion well.

#### Patient reports:

Cravings 0/10; anxiety 5/10; depression 4/10; and hours of uninterrupted sleep 6.

#### Infusion 6:

Client reports overall he feels like he’s doing very well in his recovery. Client reports he will be moving houses soon and the move is increasing his anxiety because he normally overthinks things, he knows everything will be okay, but the anxiety is getting the best of him at times. Client denies depression. Client self-rates his anxiety and his cravings at a 2 out of 10 and self-rates his pain at an 8 out of 10 for his lower back. Client says he talks to his housemates and his treatment team and sometimes it helps with anxiety, but he just needs to “go through the waves” client says he does not want to relapse, but he thinks about using from time to time without action. IV site dry and intact, client tolerated infusion well.

#### Patient reports:

Cravings 2/10; anxiety 2/10; depression 0/10; and hours of uninterrupted sleep 4.

#### Infusion 7:

Client reports he is doing well overall, and he believes his strength to remain sober is there. Client self-rated his cravings at a 1 out of 10, client says he meditates when he has cravings, and it helps a lot. Client reports anxiety and depression is a 1 out of 10. Advised client to practice breathing and meditation and to discuss with case manager, client expressed understanding. IV site dry and intact, client tolerated infusion well.

#### Patient reports:

**Figure 34:**
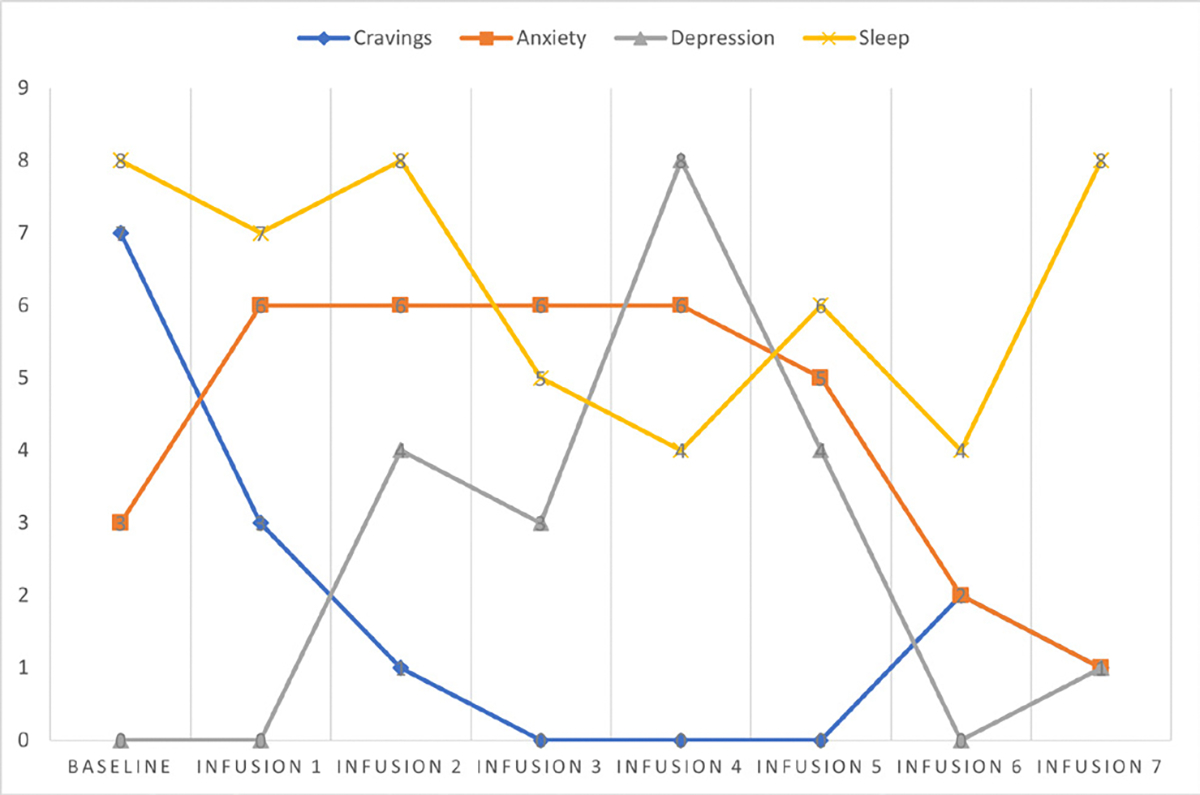
Patient 33 (Male/31/meth).

Cravings 1/10; anxiety 1/10; depression 1/10; and hours of uninterrupted sleep 8 (Table 35 and figure 34).

### Patient 34 (Male/30/ETOH/marijuana)

#### Consultation:

30-year-old male patient presents today for complaints of DOC is marijuana and alcohol drinking 30 pack + daily last use was 1 month ago. This is the client’s first treatment facility. Denies any seizures or OD in the past. Currently denies any chest pain, no SOB, no abdominal pain, no n/v/d. “I don’t have any motivation or energy to do anything. I experience anxiety daily and try to take meds 3x a day. I have difficulty concentrating. I get irritated and angry often and need to walk away.”

#### Baseline:

This is patient first infusion. Blood will be drawn on my next visit as today is friday and there are no schedule lab pick-ups. Patient reports feeling okay, he says he likes being in treatment and feels positive. Patient appears to be reserved and lethargic. Patients’ responses to questions are delayed and when asked, the client confirms that he experiences brain fog. Patient states sleep is good at 9 h and evening because he takes sleeping medication, patient confessed that he does feel groggy when he wakes up after sleeping-on-sleeping meds. Cravings for alcohol are around 5 out of 10, patient reports he sometimes daydreams about it, advised patient to use breathing techniques and grounding exercises to help as well as to discuss thoughts with case manager, client expressed understanding. IV site dry and intact, patient tolerated infusion well.

#### Patient reports:

Cravings 5/10; anxiety 3/10; depression 5/10; and hours of uninterrupted sleep 8.

#### Infusion 1:

Patient reports he didn’t experience any difference after his first infusion. The patient reports the only difference is that he became thirstier and wanted to drink water, the patient wondered if that was normal. Asked the client how much water he normally drinks, and the client states he drinks a lot of coffee and about 5 cups of water. The patient is 6’4” and he is around 200 lbs., advised patient for his height and weight he needs to drink more water than 5 cups, educated patient on the importance of water intake, patient expressed understanding. IV site dry and intact, patient tolerated infusion well.

#### Patient reports:

Cravings 3/10; anxiety 3/10; depression 3/10; and hours of uninterrupted sleep 8.

#### Infusion 2:

Blood drawn from Right Forearm from IV catheter per practitioner’s order. 4SST 1LAV 1UA obtained and sent to integritox. Patient states he is feeling okay. The patient appears to be in a better mood, improved mood is evidenced by patient’s willingness to talk to engage in conversation. Patient joked around with staff; improved mood is observed. Patient admits his depression is around a 3 out of 10, advised patient to practice breathing techniques and meditation, advised patient to discuss with therapist and case manager, client expressed understanding. IV site dry and intact, patient tolerated infusion well.

#### Patient reports:

Cravings 0/10; anxiety 0/10; depression 3/10; and hours of uninterrupted sleep 8.

#### Infusion 3:

Patient reports not feeling a big difference yet. Patient appears to be more open and in a better improve, improvements are evidenced by patient’s enthusiasm to talk and share, patient willingness to engage in conversation. Patient states he is sleeping very well at 8 h an evening. Patient drinks about 5 cups of water, advised client to increase water intake, especially if he drinks a lot of caffeinated beverages. IV site dry and intact, patient tolerated infusion well.

#### Patient reports:

Cravings 0/10; anxiety 3/10; depression 3/10; and hours of uninterrupted sleep 8.

#### Practitioner FU:

Client presents to clinic for NAD follow up. Currently on infusion number 4. States he has less anxiety and depression but doesn’t know if it’s from NAD or his new meds “kicking in”. “I have a little bit of cravings for alcohol but has gotten better. I haven’t noticed much else yet.”

#### Infusion 4:

Patient reports not feeling a big difference yet. Patient appears to be more open and in a better improve, improvements are evidenced by patient enthusiasm to talk and share, patient willingness to engage in conversation. Patient drinks about 5 cups of water, advised patient to increase water intake, especially if he drinks a lot of caffeinated beverages. IV site dry and intact, patient tolerated infusion well.

#### Patient reports:

Cravings 0/10; anxiety 3/10; depression 3/10; and hours of uninterrupted sleep 8.

#### Infusion 5:

Patient states he noticed, if he were to compare it from his first visit, patient’s anxiety and depression has decreased significantly. Patient also denies any cravings. Patient reports he does not take anxiety medication, but he noticed a change and the improvement makes him happy and optimistic. IV site dry and intact, patient tolerated infusion well.

#### Patient reports:

Cravings 0/10; anxiety 3/10; depression 3/10; and hours of uninterrupted sleep 8.

#### Infusion 6:

Patient states he is really noticing his mood and energy has improved, improvements are evidenced by patient’s willingness to engage in conversation and share his thoughts, patient states it is helping him in social settings. Patients report anxiety and depression at 3 out of 10 but it has been a big improvement overall. IV site dry and intact, patient tolerated infusion well.

#### Patient reports:

Cravings 0/10; anxiety 3/10; depression 3/10; and hours of uninterrupted sleep 8.

#### Infusion 7:

Patient reports anxiety and depression at a 3 out of 10 but it has been a big improvement; overall, but his overall anxiety and depression has decreased. Patient states he is really noticing his mood and energy has improved, improvements are evidenced by patient willingness to engage in conversation and share his thoughts, patient states it is helping him in social settings. IV site dry and intact, patient tolerated infusion well.

#### Patient reports:

**Figure 35:**
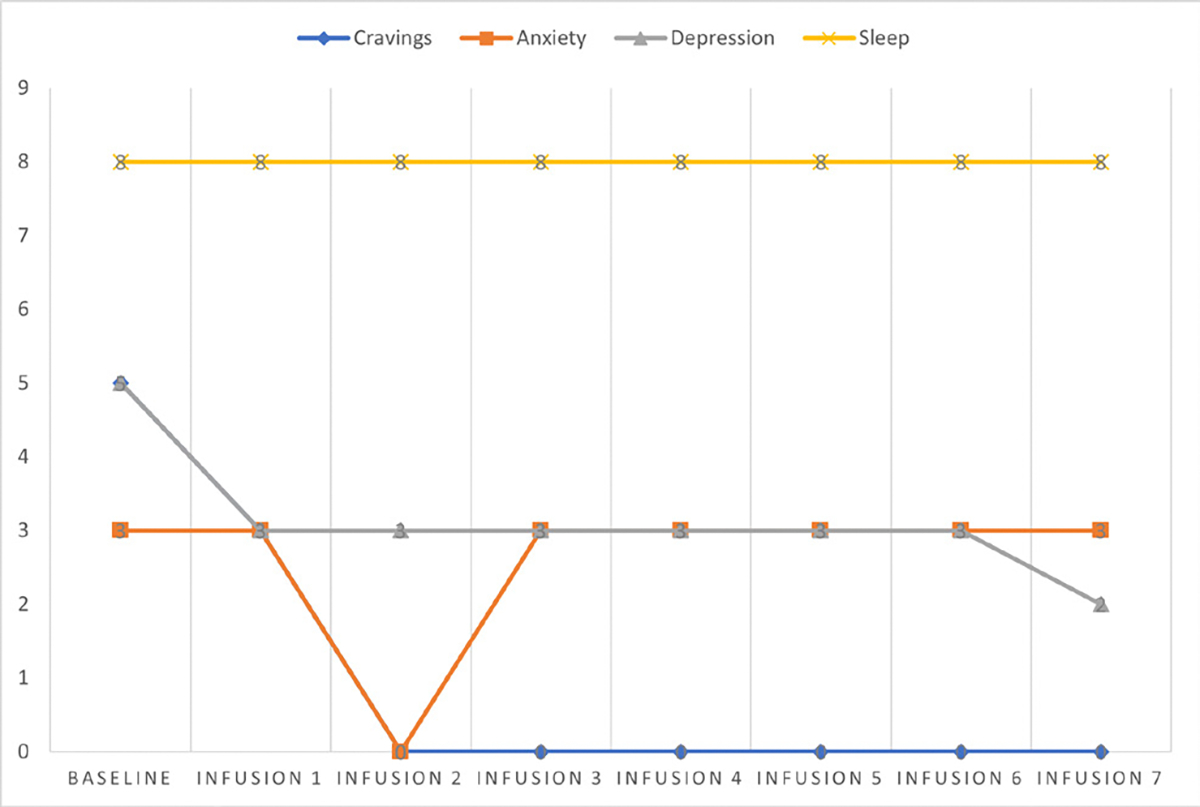
Patient 34 (Male/30/ETOH/marijuana).

Cravings 0/10; anxiety 3/10; depression 2/10; and hours of uninterrupted sleep 8 (Table 36 and figure 35).

### Patient 35 (Male/37/meth)

#### Consultation:

37-year-old male patient presents today for complaints of DOC is meth via IV. Last use was 6/10 and longest sobriety is 3 years in 25 years, and this is client’s 3rd treatment facility. No OD in the past and no seizures in the past. Currently states “I have no cravings, but I do feel like my brain is really foggy and hard to concentrate, and I’m really anxious and can’t sleep, my thoughts keep racing.”

#### Baseline:

Blood drawn from Left AC from IV catheter per practitioner’s order. 4SST 1LAV 1UA obtained and sent to integritox. Patient self-rates cravings 5/10, anxiety 6/10, depression 7/10 situational. Patient reports drinking 8 cups of water. Encouraged patients to increase fluid intake. Patient was receptive. Patient reports sleeping 4 h and waking up feeling tired. Encouraged patients to practice meditation to help ease anxiety and help relax before bed. Patient was receptive. Patient was observed relaxing and conversing with peers during infusion. Patient tolerated treatment well but did c/o mild nausea. The patient reported alleviated symptoms after 5 min. Patient completed treatment with no other complaints.

#### Patient reports:

Cravings 5/10; anxiety 6/10; depression 7/10; and hours of uninterrupted sleep 4.

#### Infusion 1:

Patient self-rates depression and cravings 0/10, anxiety 3/10 situational. Patient reports only 8 cups of water, encouraged patient to increase fluid intake and educated on importance of hydration specially with hot weather. Patient was receptive. Patient reports getting a full 8 h of sleep and feeling well rested. The patient was observed relaxing during infusion. Patient tolerated treatment well with no complaint of discomfort.

#### Patient reports:

Cravings 0/10; anxiety 3/10; depression 0/10; and hours of uninterrupted sleep 8.

#### Infusion 2:

Client denies any cravings or pain, client self-rates his anxiety at a 7 out of 10 and his depression at a 4 out of 10. Client expressed that he discussed with case manager and therapist about his feelings and is currently trying to work through it. Client drinks about 100 oz of water daily, advised client to increase water intake, client expressed understanding. IV site dry and intact, client tolerated infusion well.

#### Patient reports:

Cravings 0/10; anxiety 7/10; depression 4/10; and hours of uninterrupted sleep 8.

#### Infusion 3:

Client reports feeling anxiety and self-rated anxiety at 2 out of 10. Advised client to use meditation techniques and breathing exercises to help him work through anxiety. Client expressed understanding. Client denies cravings, depression or pain. Sleep is reported as good at 8 h an evening. Client admits drinking about 80 oz of water a day, client says he tries his best to drink water. IV site dry and intact, client tolerated infusion well.

#### Patient reports:

Cravings 0/10; anxiety 2/10; depression 0/10; and hours of uninterrupted sleep 8.

#### Infusion 4:

Client reports feeling down, client expressed that he is sad because some of his friends have overdosed. Client says he wants to remain “clean” client is sad people around him aren’t doing well. Advised client to focus on his own sobriety first and foremost. IV site dry and intact, client tolerated infusion well.

#### Patient reports:

Cravings 0/10; anxiety 4/10; depression 6/10; and hours of uninterrupted sleep 6.

#### Infusion 5:

Client reports he feels good. Client says the mental clarity is improving and he is able to retain information better and he feels clear headed. Client denies signs or symptoms or cravings, anxiety, depression or pain. Client says he drank 8 cups of water but knows he should increase his water intake. Sleep is good at 8 h an evening. IV site dry and intact, client tolerated infusion well.

#### Patient reports:

Cravings 0/10; anxiety 0/10; depression 0/10; and hours of uninterrupted sleep 8.

#### Infusion 6:

Client reports he feels good. Client denies signs or symptoms or cravings, anxiety, depression or pain. Client says his mental clarity is improving and he is able to retain information better and he feels clear headed. Client says he drank 8 cups of water but knows he should increase his water intake. Sleep is good at 8 h an evening. IV site dry and intact, client tolerated infusion well.

#### Patient reports:

Cravings 0/10; anxiety 0/10; depression 0/10; and hours of uninterrupted sleep 8.

#### Infusion 7:

Patient self-rates no cravings, anxiety, depression, or pain. Patient states have no BM issues. Patient reports having a high activity level. Patient reports having a good appetite by eating 3 meals a day and drinking 8 glasses of water daily. Patients tolerated treatment well with no pain or discomfort.

#### Patient reports:

**Figure 36:**
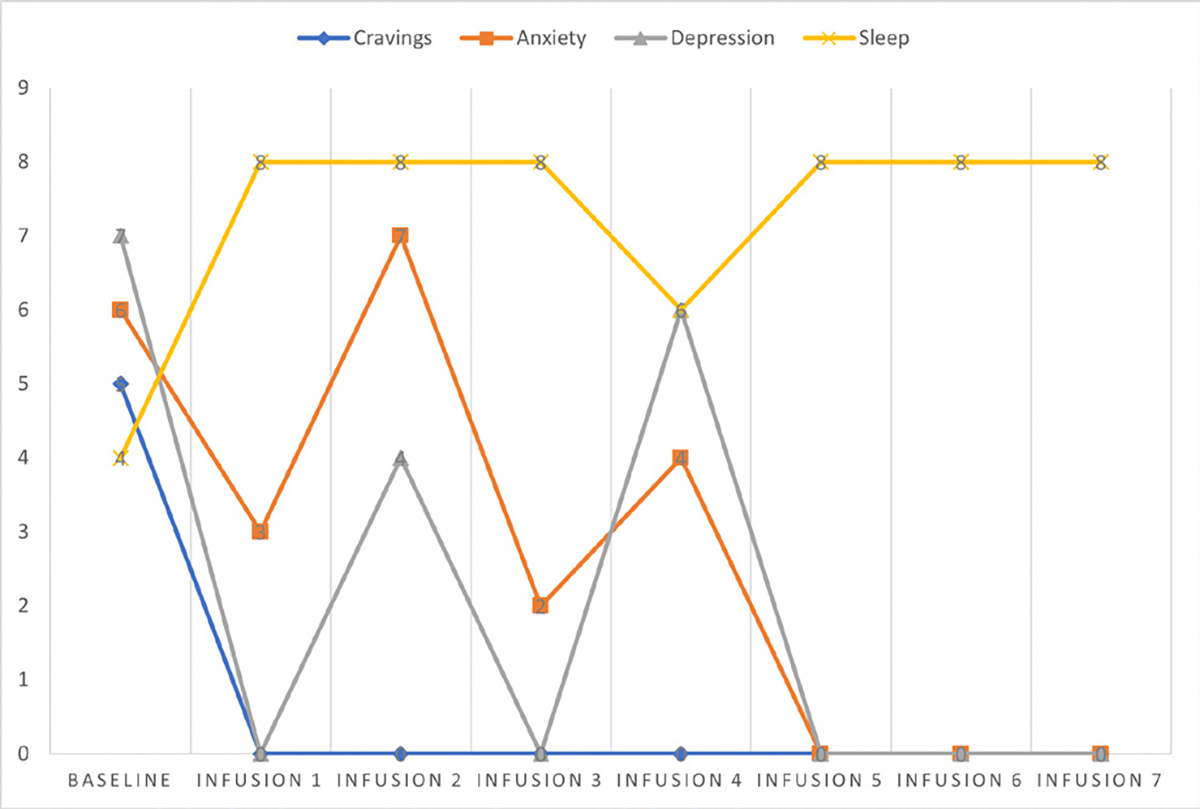
Patient 35 (Male/37/meth).

Cravings 0/10; anxiety 0/10; depression 0/10; and hours of uninterrupted sleep 8 (Table 37 and figure 36).

### Patient 36- (Male/30/heroin/meth/duster/ETOH)

#### Consultation:

30-year-old male patient presents today for complaints of “DOC is heroin via IV 1 g daily, meth, duster and alcohol 1 L every 2 days. Last use was 20 days ago, and longest sobriety is 2 years. This is the client’s 4^th^ treatment facility. Denies any OD or seizures in the past. Currently states “I have very little energy without meth, I’m super irritable and short tempered coming off of alcohol and I really have a hard time concentrating, also don’t drink any water.”

#### Baseline:

Blood drawn from Right AC from IV catheter per practitioner’s order. 4SST 1LAV 1UA obtained and sent to Integritox. Patient reports to first infusion with c/o of difficulty sleeping. Patient self-rates anxiety, cravings and depression 4/10. Patient reports to keep himself very active and drinks about 2 L of water daily. Encouraged patients to continue healthy habits and to avoid drinking fluids late in the evening to avoid waking up to use the restroom. Patient was receptive. The patient was observed relaxing and listening to music during infusion. Patient tolerated treatment well with no complaint of discomfort.

#### Patient reports:

Cravings 4/10; anxiety 4/10; depression 4/10; and hours of uninterrupted sleep 4.

#### Infusion 1:

Patient self-rates anxiety, cravings and depression to be 0/10. Patient denies pain. patient reports he only slept 4 h. Encouraged patient to practice meditation to help relax before bed. Patient was receptive. The patient was observed to be cheerful and conversing with peers during infusion. Patient tolerated treatment well with no complaint of discomfort.

#### Patient reports:

Cravings 0/10; anxiety 0/10; depression 0/10; and hours of uninterrupted sleep 4.

#### Infusion 2:

Patient self-rates depression, anxiety 0/10 and Cravings 4/10. Patient denies myalgia. Patient reports he has been having high cravings all weekend. Patient states he has been struggling and thinking of using it constantly. Encouraged patient to communicate with his CM and treatment team and to remain positive about his recovery. Patient was receptive. Patient reports drinking 1 L of water daily and sleeping 8 h. Patient was observed restless during infusion getting up to walk around and conversing with his peers. Patient tolerated treatment well with no complaint of discomfort.

#### Patient reports:

Cravings 4/10; anxiety 0/10; depression 0/10; and hours of uninterrupted sleep 8.

#### Infusion 3:

Patient self-rates anxiety, depression and cravings 0/10. Patient denies myalgia. Patient reports drinking 4 cups of water, having a good appetite and sleeping 8 h patient reports he feels more energized and less foggy in the mornings. Patients maintain a moderately active lifestyle. Encouraged patients to increase fluid intake. The patient was observed to be more at ease today relaxing and sitting down during infusion. Patient tolerated treatment well with no complaint of discomfort.

#### Patient reports:

Cravings 0/10; anxiety 0/10; depression 0/10; and hours of uninterrupted sleep 8.

#### Infusion 4:

Client denies cravings, anxiety, depression or pain. The client appears to be nervous about IV insertion, anxiety started at a 6 out of 10, post IV insertion, anxiety decreased to a 0 out of 10. The client appears to be in a good mood. A good mood is evidenced by clients joking around and participating in conversations with staff and other clients. Client states he drinks 1 cup of water a day, advised client to increase water intake. client expressed understanding. IV site dry and intact, client tolerated infusion well.

#### Patient reports:

Cravings 0/10; anxiety 0/10; depression 0/10; and hours of uninterrupted sleep 8.

#### Infusion 5:

Patient reports energy level and activity level as high. Patient says, “I feel pretty okay today” patient denies cravings, depression and pain. Patient self-rates his anxiety at a 4 out of 10, advised patient to practice meditation and breathing techniques, patient expressed understanding. IV site dry and intact, patient tolerated infusion well.

#### Patient reports:

Cravings 0/10; anxiety 4/10; depression 0/10; and hours of uninterrupted sleep 8.

#### Infusion 6:

Patient reports feeling “very good, good energy” sleep is reported to have improved, sleep is reported at 8 h an evening and patient states he wakes up with energy. Patient denies cravings, anxiety, depression or pain. Water intake is low at 15 oz a day, advised and educated patient on importance of increasing water intake, patient expressed understanding. Patient is observed relaxed in recliner, IV site dry and intact.

#### Patient reports:

Cravings 0/10; anxiety 0/10; depression 0/10; and hours of uninterrupted sleep 8.

#### Infusion 7:

Patient appears to be in a good mood, talking about he is looking forward to the infusion and he is happy about his recovery. Patient denies cravings, anxiety, depression and pain. He admits that sleep is bad at 1 – 2 h intervals. “I wake up about 4 times a night and I eat” patient says he is taking Seroquel 800 mg although he is prescribed 400 mg, advised patient to discuss with his treatment team and he should follow doctors’ orders. Patient says he understands. IV site dry and intact, patient tolerated infusion well.

#### Patient reports:

**Figure 37:**
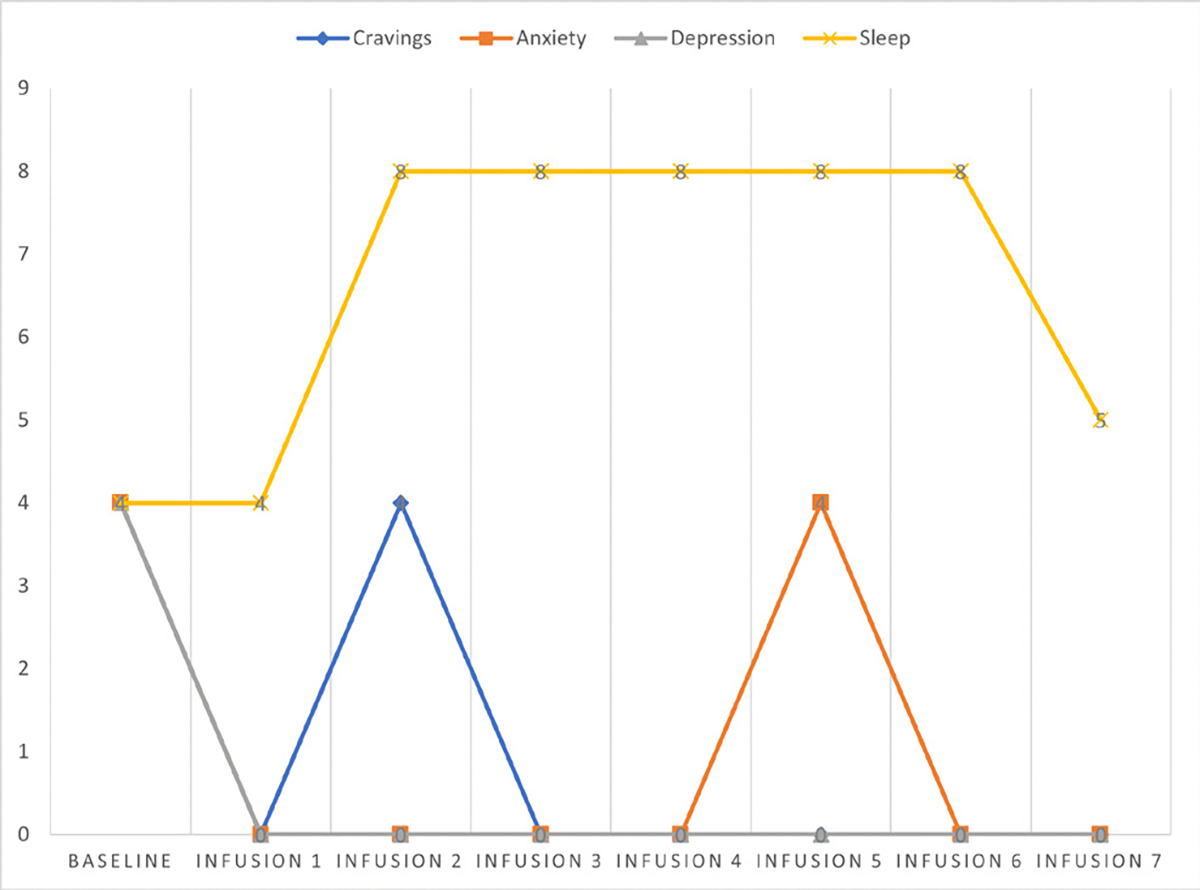
Patient 36 (Male/30/heroin/meth/duster/ETOH).

Cravings 0/10; anxiety 0/10; depression 0/10; and hours of uninterrupted sleep 5 (Table 38 and figure 37).

### Patient 37 (Male/31/heroin/meth/benzos)

#### Consultation:

31-year-old male patient presents today for complaints of “DOC is meth, heroin via IV and smoking using 3 – 4 g daily and Xanax using 2 – 6 mg daily. Last use was 8/3, longest sobriety is 9 months, and this is client’s 1st treatment facility. States 2 OD and 3 seizures in the past. Currently “I have no energy daily and very tired all the time, anxiety 3/10 and, difficulty concentrating and nausea constant with abdominal pain, hx of precancerous polyps and have constant diarrhea since April. Has colonoscopy scheduled for 9/10.”

#### Baseline:

Patient self-rates cravings 5/10, anxiety 0/10, and anxiety 3/10. Patient c/o of left shoulder pain since April. Patient reports good appetite, drinks half a gallon of water and sleeps 1 – 4 h interrupted sleep. Patient c/o of diarrhea r/t precancerous polys. Patient has scheduled colonoscopy next month. Encouraged patients to practice meditation or breathing exercises before bed to help relax and to limit drinking water late in the evening to avoid waking up to use restroom. Patient was receptive. Patient was observed relaxing during infusion and watching TV. Patient tolerated treatment well with no complaint of discomfort.

#### Patient reports:

Cravings 5/10; anxiety 3/10; depression 0/10; and hours of uninterrupted sleep 2.

#### Infusion 1:

Patient is in a great mood today. Patient self-rates his cravings, anxiety, and depression at a level 0 out of 10, pain at a level 8 out of 10 on his left shoulder and has had it since April. Patient states having a good appetite by eating 2 meals a day and drinking approx. 32 oz of water daily. Encouraged patients to drink more water. Patient states having diarrhea, will be eliminating magnesium from his Myers bag. Patient states having a low activity level. Patients tolerated treatment well with no pain or discomfort.

#### Patient reports:

Cravings 0/10; anxiety 0/10; depression 0/10; and hours of uninterrupted sleep 3.

#### Infusion 2:

Blood drawn from right AC via IV catheter per practitioners’ order. 4SST 1LAV 1UA obtained and sent to integritox. Patient self-rates cravings and anxiety 2/10, depression 0/10. Patient c/o left throbbing shoulder pain since April. Patient reports moderate appetite, drinking 24 oz of water and sleeping 4.5 h. Patient reports his anxiety has decreased and his energy levels have gotten better. The patient was observed relaxing and watching TV during infusion. Patient tolerated treatment well with no complaint of discomfort.

#### Patient reports:

Cravings 2/10; anxiety 2/10; depression 0/10; and hours of uninterrupted sleep 4.

#### Infusion 3:

Patient comes into the office in a good mood. The patient self-rates his anxiety at 5/10 (situational) and patient denies pain at the moment. Patient states having a good appetite by eating 3 meals a day and drinking approx. 24 oz of water. Told the patient importance of drinking more water, patient was receptive. Patient states having no BM issues and having a moderate activity level. Patients tolerated treatment well with no pain or discomfort.

#### Patient reports:

Cravings 0/10; anxiety 5/10; depression 0/10; and hours of uninterrupted sleep 7.

#### Practitioner FU:

Patient presents for follow up visit. He is on his fifth infusion. He states his anxiety and depression have improved. His sleep has also improved. He has more energy, but still would like more energy. He does have some cravings and states this is due to his recent surgery he had to remove some cancerous polyps from his colon 1 week ago.

#### Infusion 4:

Patient reports feeling good overall. He currently is 2 months sober “but I don’t count” patient denies cravings for drugs and alcohol and denies depression. Anxiety is self-rated at a 3 out of 10 which is an improvement per patient. Advised him to practice coping mechanisms like breathing exercises and grounding techniques to help with anxiety, patient expressed understanding. Patient tolerated infusion well. IV site dry and intact.

#### Patient reports:

Cravings 0/10; anxiety 3/10; depression 0/10; and hours of uninterrupted sleep 6.

#### Infusion 5:

Patient reports he feels “amazed” patient reports having a great weekend, he stayed active and involved by playing volleyball with his peers and sky diving and patient states he attended a couple of meetings. He denies cravings, anxiety and depression. Patient self-rates pain at a 3 out of 10 because he is sore but no physical pain, active weekend gave him sore muscles. IV site dry and intact, he tolerated infusion well.

#### Patient reports:

Cravings 0/10; anxiety 0/10; depression 0/10; and hours of uninterrupted sleep 8.

#### Infusion 6:

Patient reports he had a good weekend and a good house meeting last night. He says he noticed his energy has improved and his focus has gotten better. Better focus allows him to better pay attention during meetings and group sessions. Patient reported sleep is good at 7 h an evening. He denies cravings, depression and pain. Anxiety is self-rated at 3 out of 10 but he his using coping mechanisms to help him. IV site dry and intact, he is tolerating infusion well.

#### Patient reports:

Cravings 0/10; anxiety 3/10; depression 0/10; and hours of uninterrupted sleep 7.

#### Infusion 7:

Patient appears to be in good mood. Good mood is evidenced by patients smiling and joking around with staff and other patients. Patient reports of cravings, anxiety and depression are low and self-rated at a 1 out of 10. Patient said he feels proud he feels good overall. Pain is self-rated at a 3 out of 10 because patient did and exercises, advised him to increase water intake to help with muscle recovery. IV site dry and intact, he tolerated infusion well.

#### Patient reports:

**Figure 38:**
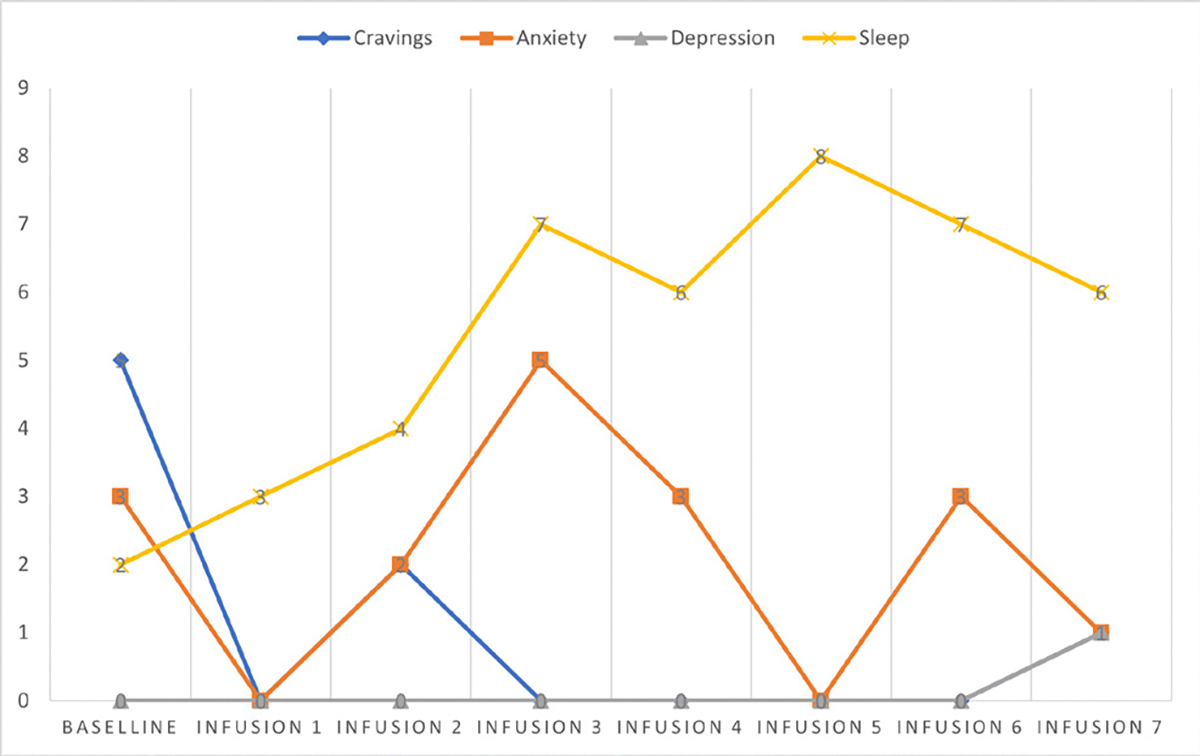
Patient 37 (Male/31/heroin/meth/benzos).

Cravings 1/10; anxiety 1/10; depression 1/10; and hours of uninterrupted sleep 6 (Table 39 and figure 38).

### Patient 38 (Male/32/meth)

#### Consultation:

32-year-old male patient presents today for complaints of DOC is meth via IV and smoking. Last use was 8/6, longest sobriety is 1 year in 2016, and this is client’s 1^st^ treatment facility. States had no OD and no seizures in the past. Currently “I have no energy daily, ++anxiety 8/10 and difficulty concentrating.”

#### Baseline:

Blood drawn from Left AC Via IV catheter per practitioner order. 4SST 1LAV 1UA obtained and sent to integritox. Patient self-rates anxiety 3/10, cravings 6/10 and depression 1/10. Patient denies myalgia. Encouraged patient to communicate his feelings to his treatment team and therapist and also practice meditation or breathing exercises. Patient was receptive. Patient reports drinking 9 cups of water and sleeping 8 h. The patient was observed relaxing talking with peers during infusion. Patient tolerated treatment well with no complaint of discomfort.

#### Patient reports:

Cravings 6/10; anxiety 3/10; depression 1/10; and hours of uninterrupted sleep 8.

#### Infusion 1:

Patient is in great spirits today. Patient self-rates his cravings today at a level 3 out of 10, anxiety and depression at a level 2 out of 10 (situational), recommended natural ways to help with anxiety such as yoga or meditation. Patient denies pain has no fever. Patient states having a good appetite by eating 3 meals a day and drinking approximately 9 glasses of water daily. Encouraged patients to drink more water. Patient states having no BM issues and having a moderate activity level. Patients tolerated treatment well with no pain or discomfort.

#### Patient reports:

Cravings 3/10; anxiety 2/10; depression 2/10; and hours of uninterrupted sleep 6.

#### Infusion 2:

Patient reports that the infusions have been helping with his cravings. Reports he feels a little stressed as his girlfriend is pregnant as of recently. Feels more motivated to stay sober but reports that he is still only sleeping 4 h at night. Patient teaching on stretching and meditation to ease some of his stress, as well as practicing deep breathing. Patient acknowledged understanding. Patients tolerated infusion well and had no complaints of discomfort. Patient was observed relaxing in recliner and watching movies during infusion. The patient completed treatment and left stable.

#### Patient reports:

Cravings 1/10; anxiety 1/10; depression 1/10; and hours of uninterrupted sleep 4.

#### Infusion 3:

Patient self-rates cravings 1/10, anxiety and depression 2/10. Patient reports wrist pain/soreness self-rated 1/10. Patient reports good appetite, no GI problems, drinking 5 cups of water and sleeping 3.5 h. Patient reports having a difficult time sleeping r/t stress with personal problems. patient reports he has been trying meditation and reading the big book. Encouraged patient to continue with meditation and also to increase his fluid intake. Patient was receptive. Patient would like to try ALA on next visit if his sleep does not improve. The provider was made aware. The patient was observed relaxing and watching TV during infusion. Patient tolerated treatment well with no complaint of discomfort.

#### Patient reports:

Cravings 1/10; anxiety 2/10; depression 2/10; and hours of uninterrupted sleep 4.

#### Practitioner FU:

Patient presents to clinic for follow up. Infusion 4. Currently states he is mentally in a good place “I have noticed that I actually can notice when I’m upset and how to handle it, or if I’m stressed. I’m aware of feelings. I feel a little less anxious as well.”

#### Infusion 4:

Patient comes into the office in a good mood. Patient self-rates his anxiety at 2/10 (situational), depression at 1/10 (situational/emotional), and patient denies pain at the moment. Recommended natural ways to help with anxiety and depression such as yoga or meditation. Patient has no fever. Patient states having a good appetite by eating 2 meals a day and drinking approx. 6 cups of water daily. Told patient the importance of drinking more water, patient was receptive. Patient states having no BM issues and having a low activity level. Patients tolerated treatment well with no pain or discomfort.

#### Patient reports:

Cravings 0/10; anxiety 2/10; depression 1/10; and hours of uninterrupted sleep 6.

#### Infusion 5:

Patient comes into the office in a good mood. Patient self-rates his anxiety, depression (situational) and pain at 1/10. The patient states pain is continuous and believes he hyperextended knee. Patient has no fever. Patient states he eats 3 meals a day and drinks 6 – 7 cups of water daily, encouraged patient to drink more water, patient was receptive. Patient denies BM issues. Patients tolerated treatment well with no pain or discomfort.

#### Patient reports:

Cravings 0/10; anxiety 1/10; depression 1/10; and hours of uninterrupted sleep 7.

#### Infusion 7:

Patient comes into the office in a good mood. Patient self-rates his anxiety at 1/10 (situational) and pain at 2/10 on his right knee and upper back, has had pain for 2 weeks now. Recommended natural ways to help with anxiety such as exercise or mediation. Patient has no fever. Patient states eating 3 meals a day and drinking approx. 1 gallon of water daily. Patient states having no BM issues and having a moderate activity level. Patients tolerated treatment well with no pain or discomfort.

#### Patient reports:

Cravings 0/10; anxiety 1/10; depression 0/10; and hours of uninterrupted sleep 8.

#### Infusion 7:

Patient comes into the office in a good mood. Recommended natural ways to help with anxiety such as exercise or mediation. Patient states eating 3 meals a day and drinking approx. 1 gallon of water daily. Patient states having no BM issues and having a moderate activity level. Patients tolerated treatment well with no pain or discomfort.

#### Patient reports:

**Figure 39:**
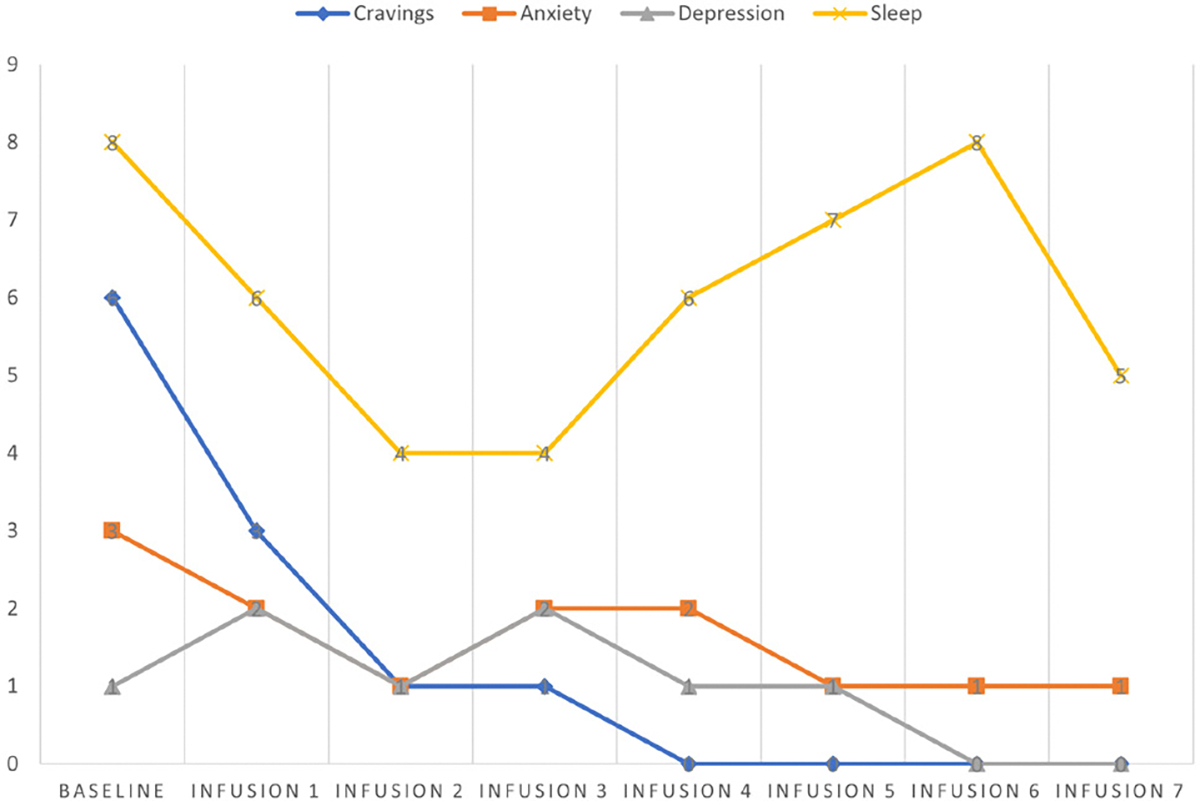
Patient 38 (Male/32/meth).

Cravings 0/10; anxiety 1/10; depression 0/10; and hours of uninterrupted sleep 5 (Table 40 and figure 39).

### Patient 39 (Male/41/meth)

#### Consultation:

41-year-old male patient presents today for complaints of DOC is meth via smoking and IV 1 g daily. Last use was 6/11 and longest sobriety is 4 years. This is the client’s first treatment facility. Currently states “I have very little energy without the meth, I’m super irritable and I really have a hard time concentrating, also don’t drink any water.”

#### Baseline:

Blood drawn from Right dorsal hand from IV catheter per practitioner’s order. 4SST 1LAV 1UA obtained and sent to integritox. Patient self-rates anxiety 3/10 situational. Patient reports chronic lower back pain 4/10. Patient reports getting 8 h of sleep and drinking 3 cups of water. Encouraged patients to increase fluid intake, educated on the importance of hydration. Patient was receptive. The patient was observed relaxing and talking with staff during infusion. Patient tolerated treatment well with no complaint of discomfort.

#### Patient reports:

Cravings 5/10; anxiety 3/10; depression 0/10; and hours of uninterrupted sleep 8.

#### Infusion 1:

Patient self-rates anxiety, cravings, and depression 0/10. Patient denies pain. Patient reports sleeping great last night and waking up with energy. Patient reports drinking 32 oz of water. Encouraged patients to increase their water intake. The patient was receptive and stated he did try drinking more today. The patient was observed cheerful and conversing with peers during infusion. patient tolerated treatment well with no complaint of discomfort.

#### Patient reports:

Cravings 0/10; anxiety 0/10; depression 0/10; and hours of uninterrupted sleep 8.

#### Infusion 2:

Patient self-rates anxiety, cravings, and depression 0/10. Patient self-rates chronic back pain 3/10. Patient reports drinking 30 oz of water. Encouraged patients to increase water intake and educated them on the importance of hydration. The patient reports sleeping 8 h of sleep and waking up feeling energized and clear minded. Patient denies any GI problems. The patient was observed to be cheerful and conversing with peers during infusion. Patient tolerated treatment well with no complaint of discomfort.

#### Patient reports:

Cravings 0/10; anxiety 0/10; depression 0/10; and hours of uninterrupted sleep 8.

#### Infusion 3:

Patient self-rates anxiety, cravings, and depression 0/10. Patient c/o chronic lower back pain 3/10. Drink 8 cups of water and sleep about 6 h. Patient reports feeling an increase in his energy levels and feels a decrease of pain in his joints. patient is happy and excited to continue with his treatments. The patient was observed relaxing during infusion. Patient tolerated treatment well with no complaint of discomfort.

#### Patient reports:

Cravings 0/10; anxiety 0/10; depression 0/10; and hours of uninterrupted sleep 6.

#### Infusion 4:

Patient comes in for his 5^th^ treatment. Patient self-rates his anxiety at a level 3 out of 10 (situational), recommended natural ways to help with anxiety such as yoga or meditation. Patient reports his pain at a level 3 out of 10, states it is his lower back and he has had this chronic pain for 15 years. Patient states eating 3 meals a day and drinking approx 32 oz of water daily. Patient states no BM issues. Patient smokes cigarettes 2 packs a day and has a high activity level. Patients tolerated treatment well with no pain or discomfort.

#### Patient reports:

Cravings 0/10; anxiety 3/10; depression 0/10; and hours of uninterrupted sleep 8.

#### Infusion 5:

Patient reports he has been experiencing some cravings and it gives him anxiety. Patient states it’s not enough to make him want to use it, but he thinks about it sometimes. Patient self-rated his cravings at a 4 out of 10 and anxiety a 4 out of 10. Advised patient to discuss with his treatment team and to practice meditation and breathing techniques, patient expressed understanding. IV site dry and intact, patient tolerated infusion well.

#### Patient reports:

Cravings 4/10; anxiety 4/10; depression 0/10; and hours of uninterrupted sleep 7.

#### Infusion 6:

Patient reports “I believe infusions helped me get off my medication” patient appears to be in good mood, good mood is evidenced by patient smiling and engaging in conversation with staff and other patients. He denies cravings and depression. The patient admits he has some anxiety and self-rates his anxiety at a 4 out of 10, patient expressed that he used coping mechanisms and grounding that is taught in class, and it helps him. IV site dry and intact, patient tolerated infusion well.

#### Patient reports:

Cravings 0/10; anxiety 4/10; depression 0/10; and hours of uninterrupted sleep 6.

#### Infusion 7:

Patient reports feeling okay. Patient appears to be in good mood as evidenced by patient talkative nature and engaging in conversation and joking with other clients and staff. Patient reports “less cravings, more energy and faster recovery when sick.” IV site dry and intact, patient tolerated infusion well.

#### Patient reports:

**Figure 40:**
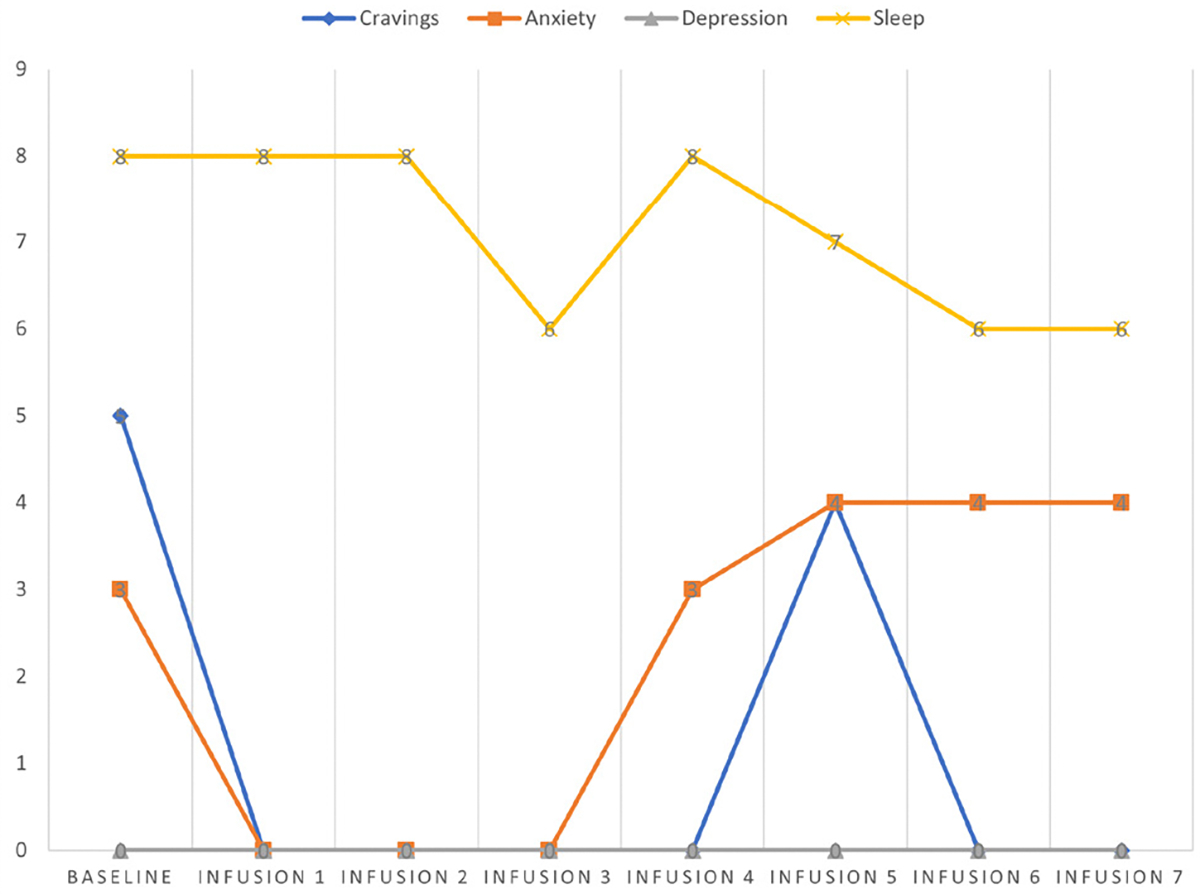
Patient 39 (Male/41/meth).

Cravings 0/10; anxiety 4/10; depression 0/10; and hours of uninterrupted sleep 6 (Table 41 and figure 40).

### Patient 40 (Male/25/heroin/meth)

#### Consultation:

25-year-old male patient presents today for complaints of DOC is heroin and meth IV using ½ g, last use being yesterday at 9 am. The longest sobriety is 6 months, and this is the client’s first treatment facility. Denies any seizures or OD in the past. Currently with back pain, cold chills, no chest pain, no SOB, no abdominal pain, no n/v/d. “I have a lot of anxiety, and my depression is surfacing not being high. I have digestion issues when I eat and feel dehydrated and nauseous all the time.”

#### Baseline:

This is client first infusion, his cravings are around a 7 out of 10 for heroin and meth, last time he used to be 10 days ago. Client reports anxiety at 6 out of 10, advised client to practice breathing exercises and grounding techniques advised client to discuss with case manager, client expressed understanding. IV site dry and intact, client tolerated infusion well

#### Patient reports:

Cravings 7/10; anxiety 5/10; depression 6/10; and hours of uninterrupted sleep 4.

#### Infusion 1:

Client reports this is his 2^nd^ infusion, and he felt okay after his first infusion. The client is experiencing a lot of anxiety and depression. Anxiety is rated at an 8 out of 10 and depression is a 6 out of 10. Advised client to practice grounding techniques and breathing exercises and to discuss with case manager, client expressed understanding. Client reports he told them about his shoulder pain as well and he is monitoring it for any changes. The client admits he is aware he is 2 weeks sober, and he is still going through withdrawals. IV site dry and intact, client tolerated infusion well.

#### Patient reports:

Cravings 0/10; anxiety 8/10; depression 6/10; and hours of uninterrupted sleep 4.

#### Infusion 2:

Client reports his anxiety is around 6 out of 10 and his depression is around a 4 out of 10, client states he has talked about his emotions with her case manager. Client reports he feels good overall, he had a visitor this weekend and it was nice to see someone outside of treatment. Client states his sleep has been pretty good, he sleeps about 5 h an evening, but he sleeps through it and does wake up with energy. IV site dry and intact, client tolerated infusion well.

#### Patient reports:

Cravings 0/10; anxiety 6/10; depression 4/10; and hours of uninterrupted sleep 8.

#### Infusion 3:

Client reports he feels good overall, he had a visitor this weekend and it was nice to see someone outside of treatment. Client states his sleep has been pretty good, he sleeps about 5 h an evening, but he sleeps through it and does wake up with energy. IV site dry and intact, client tolerated infusion well.

#### Patient reports:

Cravings 0/10; anxiety 2/10; depression 2/10; and hours of uninterrupted sleep 6.

#### Infusion 4:

Client reports he feels good overall, he had a good weekend, client went to the beach with his housemates, and it was nice to bond and do something outside of treatment. Client states his sleep has been pretty good, he sleeps about 5 h an evening, but he sleeps through it and does wake up with energy. IV site dry and intact, client tolerated infusion well.

#### Patient reports:

Cravings 0/10; anxiety 1/10; depression 1/10; and hours of uninterrupted sleep 5.

#### Infusion 5:

Client reports he actually feels good, client states his mood has been off and his hormones are trying to stabilize. Client denies cravings and depression. Client admits he still has anxiety and self-rated at 6 out of 10, advised client to practice meditation and breathing exercises and to discuss with case manager. Client expressed understanding. IV site dry and intact, client tolerated infusion well.

#### Patient reports:

Cravings 0/10; anxiety 6/10; depression 0/10; and hours of uninterrupted sleep 5.

#### Infusion 6:

Client came in with a headache, client rated his headache at a 7 out of 10. Client reports other than his headache he actually feels good, client states his mood has been off and his hormones are trying to stabilize. Client denies cravings and depression. The client admits he still has anxiety and is self-rated at 5 out of 10, advised client to practice meditation and breathing exercises and to discuss with case manager. Client expressed understanding. IV site dry and intact, client tolerated infusion well.

#### Patient reports:

Cravings 0/10; anxiety 5/10; depression 0/10; and hours of uninterrupted sleep 8.

#### Infusion 7:

Client reports he has a headache again, not as bad as last time but headache is present. Client self-rated headache at a 4 out of 10, client reports he felt better after infusion last time. Client admits he does not drink too much water, client consumes about 2 – 3 cups of water daily and caffeine intake is more. Educated client on the importance of water intake. Headache was relieved after infusions, rated at a 1 out of 10. IV site dry and intact, client tolerated infusion well.

#### Patient reports:

**Figure 41:**
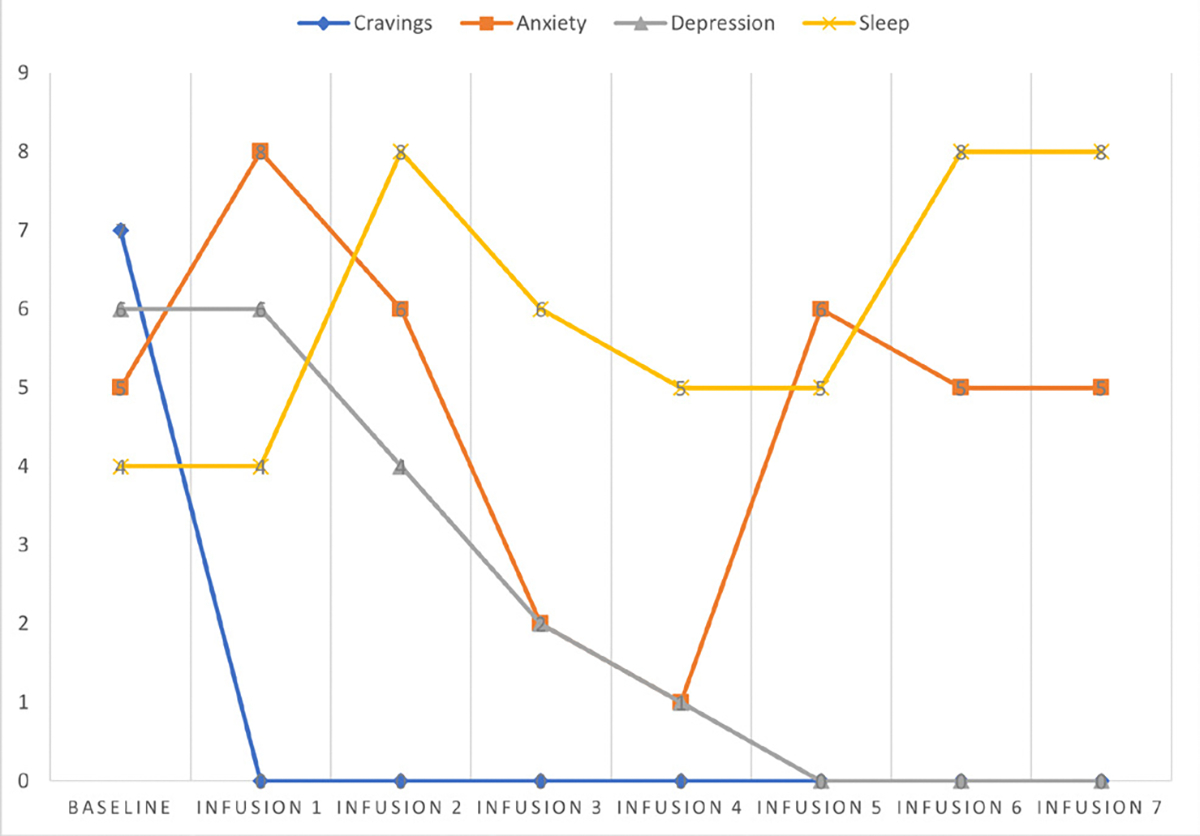
Patient 40 (Male/25/heroin/meth).

Cravings 0/10; anxiety 5/10; depression 0/10; and hours of uninterrupted sleep 8 (Table 42 and figure 41).

### Patient 41 (Male/42/meth)

#### Consultation:

Patient is being seen for NAD consultation. Drug use: meth. He smokes 1 – 2 GM of meth per day. He also takes Xanax a couple of times per month. He last used meth yesterday morning. He last took 2 mg Xanax yesterday morning as well. He was last in treatment in Feb 2019. He relapsed about 4 months ago. In Feb 2019, he spent 30 days in treatment and was treated for the same DOC. Currently “I have a lot of shaking and fatigue, I’m irritable and angry. I haven’t been able to eat properly and feel weak. My anxiety is making me crazy because I don’t want to start a cycle of treatment centers over and over in my life.”

#### Baseline:

Patient reports for first infusion treatment. Reports that he has been in detox for 7 days. Reports his DOC was meth. Reports that during his substance abuse he was not eating very much or leading healthy habits at all. Feels very depleted from years of use and currently feels very fatigued and run down. Reports that he is very tired and wants to sleep all day and is not motivated at all. Patient relaxed in recliner and slept during infusion. Patient tolerated treatment well and had no complaints of discomfort. The patient completed treatment and left stable.

#### Patient reports:

Cravings 5/10; anxiety 6/10; depression 6/10; and hours of uninterrupted sleep 10.

#### Infusion 1:

Patient reports cravings are low; patient states he sometimes has using dreams but try to focus more on sobriety. Patient states anxiety and depression is around a 6 out of 10, patient reports feeling nervous about infusion, after last infusion patient reports sleeping well, but he normally sleeps well, he states he felt his sleep was deeper. IV site dry and intact, no signs or symptoms of infiltration or irritation.

#### Patient reports:

Cravings 2/10; anxiety 6/10; depression 6/10; and hours of uninterrupted sleep 10.

#### Infusion 2:

Patient reports cravings are low; patient states he sometimes has using dreams but try to focus more on sobriety. Patient states anxiety and depression is around a 5 out of 10, patient reports feeling nervous about infusion, advised patient on ways to work through anxiety and depression through breathing techniques and grounding techniques, advised patient to discuss with case manager. Patient reports sleeping well, but he normally sleeps well, he states he felt his sleep was deeper. IV site dry and intact, no signs or symptoms of infiltration or irritation.

#### Patient reports:

Cravings 2/10; anxiety 5/10; depression 5/10; and hours of uninterrupted sleep 10.

#### Infusion 3:

Patient reports sleeping well, but he normally sleeps well, he states he felt his sleep was deeper. Patient states anxiety and depression is around a 4 out of 10 and has overall improved, patient reports feeling nervous about infusion, advised patient on ways to work through anxiety and depression through breathing techniques and grounding techniques, advised patient to discuss with case manager. IV site dry and intact, no signs or symptoms of infiltration or irritation.

#### Patient reports:

Cravings 2/10; anxiety 4/10; depression 4/10’; and hours of uninterrupted sleep 10.

#### Infusion 4:

Patient states anxiety and depression has decreased and is around a 2 out of 10 and has overall improved, patient reports feeling nervous about infusion, advised patient on ways to work through anxiety and depression through breathing techniques and grounding techniques, advised patient to discuss with case manager. IV site dry and intact, no signs or symptoms of infiltration or irritation.

#### Patient reports:

Cravings 2/10; anxiety 2/10; depression 2/10; and hours of uninterrupted sleep 10.

#### Infusion 5:

Patient reports low activity level. Activity level is low due to covid19 and quarantine order, advised patient to practice at home workouts and to do chores around the house, patient expressed understanding. Patient states sleep is not the best and wakes up often throughout the night to use the restroom and to walk around. Advised patient to practice breathing exercises and meditation and to avoid liquids prior to bed, patient expresses understanding. IV site dry and intact, patient tolerated infusion well.

#### Patient reports:

Cravings 0/10; anxiety 2/10; depression 2/10; and hours of uninterrupted sleep 2.

#### Infusion 6:

Patient reports low activity level. Patient states he is trying to increase exercise per our last conversation but is having difficulty, he states he will work on its patient states sleep is not the best and wakes up often throughout the night to use the restroom and to walk around. Advised patients to practice breathing exercises and meditation and to avoid liquids prior to bed, patient expressed understanding. IV site dry and intact, patient tolerated infusion well.

#### Patient reports:

Cravings 0/10; anxiety 2/10; depression 2/10; and hours of uninterrupted sleep 10.

#### Infusion 7:

Patient reports he is trying to increase his level of physical activity but is finding it hard to find a good routine that works for him, encouraged patient to start with stretches and low-level exercise that can be done indoors. Patient was receptive. Patient reports restless sleep and wakes up often throughout the night to use the restroom and to walk around. Encouraged patient to practice breathing exercises and meditation and to avoid fluids prior to bedtime, patient was receptive. Patient reports recent medication changes that may have affected his sleeping. Patient was started on wellbutrin for about a week now. Patient was observed watching TV and relaxing on recliner. Patient tolerated treatment well with no complaint of discomfort. The patient completed treatment and was left in stable condition.

#### Patient reports:

**Figure 42:**
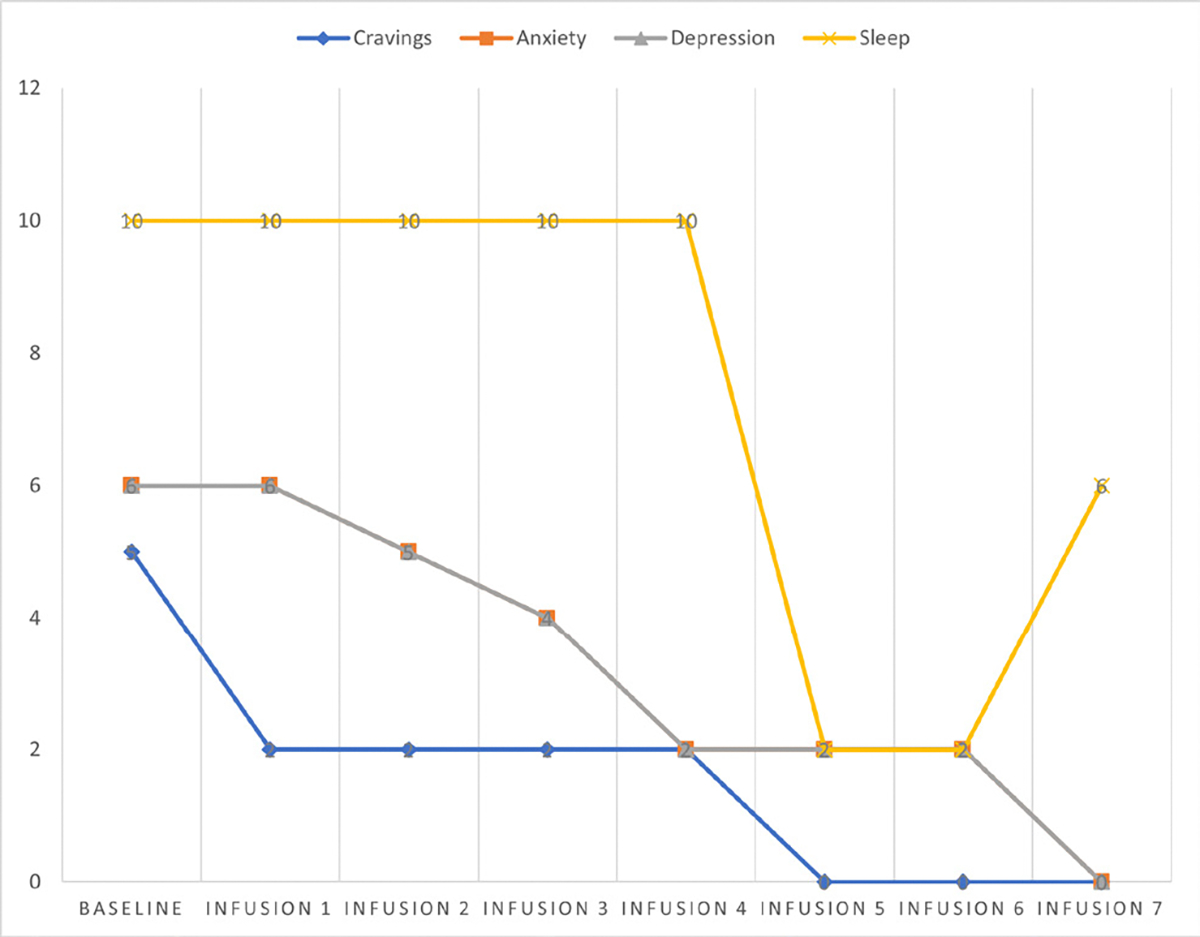
Patient 41 (Male/42/meth).

Cravings 0/10; anxiety 0/10; depression 0/10; and hours of uninterrupted sleep 6 (Table 43 and figure 42).

### Patient 42 (Male/29/meth)

#### Consultation:

Patient presents to clinic for NAD consultation. DOC is meth via smoking and snorting. Last use was yesterday, patient arrived yesterday from ok as well. The longest sobriety is 5 months. Has been to 1 other treatment facility. Denies any OD or seizures in the past. Currently “I am extremely exhausted, and I just want to sleep all day. Also having body aches, I haven’t been drinking many fluids.”

#### Baseline:

Patient reports this is his first infusion, patient just arrived about 2 days ago. He states he is doing well and is happy to start his journey toward sobriety and being clean. Patient currently experiencing body aches and states his cravings are at a 6 out of 10, advised patient to practice yoga and meditation, patient expressed understanding. IV site dry and intact, patient tolerated infusion well.

#### Patient reports:

Cravings 6/10; anxiety 0/10; depression 4/10; and hours of uninterrupted sleep 4.

#### Infusion 1:

Patient reports this is his second infusion, and he already notices improvements. Patient currently experiencing body aches and states his cravings are at a 2 out of 10, advised patient to practice yoga and meditation, patient expressed understanding. He states he is doing well and is happy to start his journey toward sobriety and being clean. IV site dry and intact, patient tolerated infusion well.

#### Patient reports:

Cravings 2/10; anxiety 0/10; depression 6/10; and hours of uninterrupted sleep 8.

#### Infusion 2:

Patient states he is doing well and is happy to start his journey toward sobriety and being clean. Patient expresses desire to stay in California and build a sober life here. Evaluation of patient is that he seems very grateful. Patient reports he already notices improvements. Patient states he is still experiencing body aches due to detox and states his cravings are at a 2 out of 10, advised patient to practice yoga and meditation, patient expressed understanding. IV site dry and intact, patient tolerated infusion well.

#### Patient reports:

Cravings 2/10; anxiety 0/10; depression 0/10; and hours of uninterrupted sleep 8.

#### Infusion 3:

Patient states he is doing well and is happy, patient reports he had a good weekend, and he played soccer at the park with his housemates. Evaluation of patient is that he seems very grateful. Patient reports he already notices improvements. Patient states he is still experiencing body aches due to detox and states his cravings are at a 1 out of 10, advised patient to practice yoga and meditation, patient expressed understanding. IV site dry and intact, patient tolerated infusion well.

#### Patient reports:

Cravings 1/10; anxiety 0/10; depression 0/10; and hours of uninterrupted sleep 8.

#### Infusion 4:

Patient reports he already notices improvements. Patient states he is very grateful. Patient states he is still experiencing body aches due to detox, but they are improving a lot since starting the infusions and states his cravings are at a 1 out of 10, advised patient to practice yoga and meditation, patient expressed understanding. IV site dry and intact, patient tolerated infusion well.

#### Patient reports:

Cravings 1/10; anxiety 0/10; depression 0/10; and hours of uninterrupted sleep 8.

#### Infusion 5:

Patient reports activity level is normal; patient states this weekend he mainly stayed indoors watching TV because it was hot outside. Patient reports feeling rested and energized. Patient states he sleeps about 8 h an evening and feels rested after sleep. The patient denies having any cravings and is feeling “really good”. IV site dry and intact, patient tolerated infusion well.

#### Patient reports:

Cravings 0/10; anxiety 0/10; depression 0/10; and hours of uninterrupted sleep 8.

#### Infusion 6:

Patient reports he drinks about 12 caffeinated beverages daily and about 24 – 36 oz of water daily. Advised patient to decrease caffeine intake and to increase water intake, educated patient on the importance of water intake, patient expressed understanding. Sleep is about 7 h an evening, patient states he does not feel rested after sleep, he tosses and turns at night. Advised patient to practice breathing techniques and meditation prior to bed, patient expressed understanding. IV site dry and intact, patient tolerated infusion well.

#### Patient reports:

Cravings 0/10; anxiety 0/10; depression 0/10; and hours of uninterrupted sleep 7.

#### Infusion 7:

Patient reports he had a good weekend. Patient states he is happier because he was able to go outside. Patient states his house took the Patient hiking; he went on an 18 mile hike to a waterfall. Patient states he drinks no caffeinated beverages. Patient reports drinking 50 oz of water, advised patient to increase water intake, especially if he will go on big hike and other activities. IV site dry and intact, patient tolerated infusion well.

#### Patient reports:

**Figure 43:**
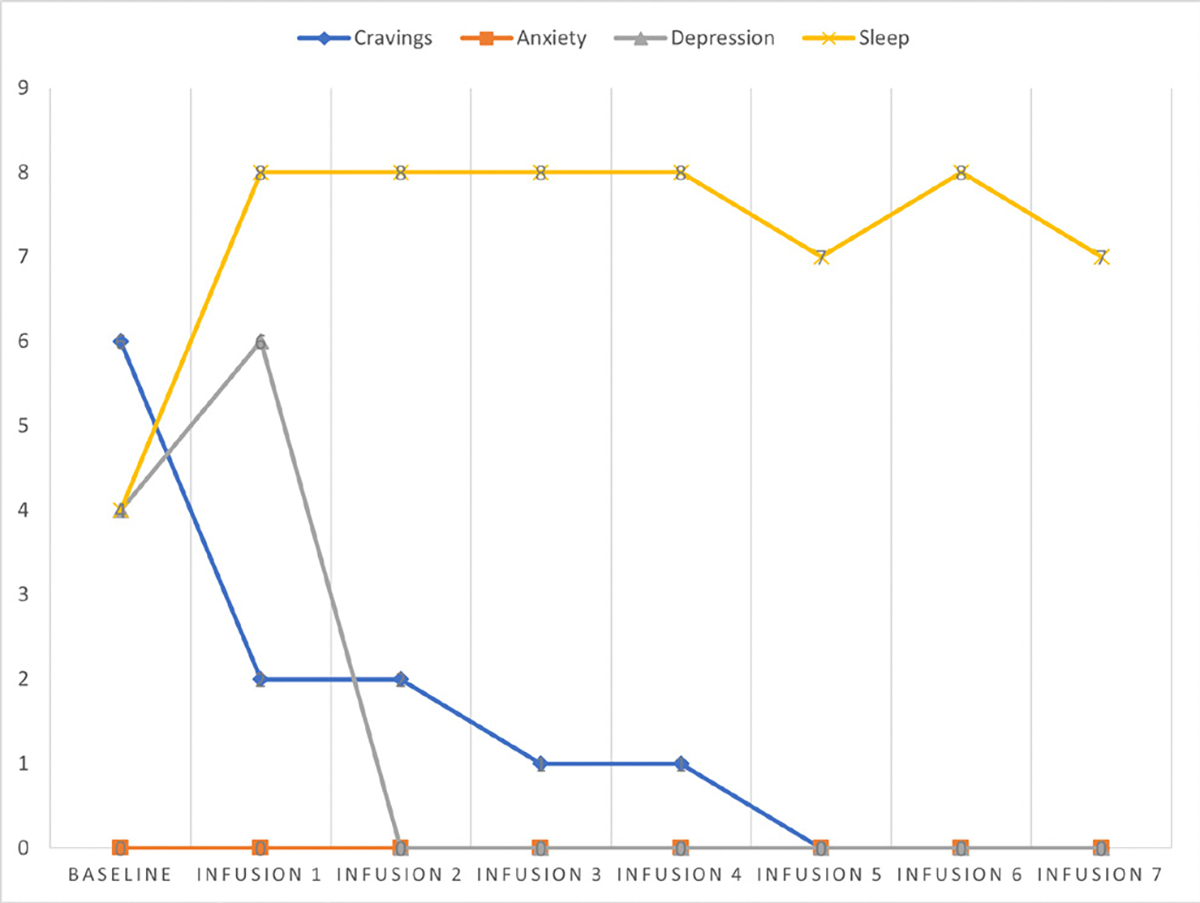
Patient 42 (Male/29/meth).

Cravings 0/10; anxiety 0/10; depression 0/10; and hours of uninterrupted sleep 7 (Table 44 and figure 43).

### Patient 43 (Male/40/meth)

#### Consultation:

Patient presents to clinic for NAD consultation. Denies any PMH, recent right-hand surgery, psych hx of anxiety and PTSD. Denies any seizures or OD in the past. DOC is meth via IV and smoking and ingesting as well as weed., started meth at age 14, last use was 1/26/2020. This is the patient’s first treatment facility. Currently, “I am having anxiety attacks 8/10 on occasions throughout the day and have been placed on valium as a maintenance to keep my anxiety levels down. I’m experiencing fatigue as well as difficulty concentrating.”

#### Baseline:

Patient reports for first infusion treatment as ordered by practitioner. Patients report severe anxiety and insomnia. Patient 7 days sober from meth and physically feels depleted and fatigued. Patient wants to better his life for his 1-year-old daughter. Patient relaxed in recliner and chatted with others. Patient tolerated treatment well and had no complaints of discomfort. The patient completed treatment and left stable.

#### Patient reports:

Cravings 8/10; anxiety 10/10; depression 9/10; and hours of uninterrupted sleep 3.

#### Infusion 1:

Patient reports that his anxiety has improved slightly since his infusion yesterday. Patient also reports that his sleep improved last night, and he only awoke a few times last night. Patient is much more relaxed today and was able to sit still. Patient relaxed in recliner and chatted with others. Patient tolerated treatment well and had no complaints of discomfort. The patient completed treatment and left stable.

#### Patient reports:

Cravings 4/10; anxiety 7/10; depression 7/10; and hours of uninterrupted sleep 5.

#### Infusion 2:

Patient reports his cravings have improved. Patient also reports that his sleep improved last night, and he only awoke a few times last night. The patient seemed to be in a happy mood and joked around while receiving infusion. IV site dry and intact, no signs or symptoms or irritation or infiltration. Patient tolerated infusion well.

#### Patient reports:

Cravings 4/10; anxiety 7/10; depression 7/10; and hours of uninterrupted sleep 6.

#### Infusion 3:

Patient reports that his anxiety has still decreased and is more situational at a 2–4/10, and he is waking less throughout the night and sleeping longer. Patient reports that he feels like his energy is increasing and his overall sense of wellbeing. Patient reports that he has had no cravings for the first time in a long time. Patient relaxed in recliner and chatted with others during infusion. Patient tolerated treatment well and had no complaints of discomfort. The patient completed treatment and left stable.

#### Patient reports:

Cravings 0/10; anxiety 4/10; depression 2/10; and hours of uninterrupted sleep 8.

#### Infusion 4:

Patient reports that his anxiety is still improving as well as his sleep. He discontinued the valium and has felt ill more fatigued the last 3 days. He has been trying to increase his fluids to help flush it from his system. Patient denies any cravings and rates it 0/10. Patient relaxed in recliner and chatted with others during infusion. Patients tolerated infusion well and had no complaints of discomfort. The patient completed treatment and left stable.

#### Patient reports:

Cravings 0/10; anxiety 2/10; depression 2/10; and hours of uninterrupted sleep 6.

#### Infusion 5:

Patient reports that he is feeling less depleted and fatigued overall. Reports that his energy is improving since stopping the valium. Patient denies any cravings and reports that his sleep is continuing to improve. Patient reports that he awoke rested today which was the first time in a long time. Patient relaxed in recliner and chatted with others during infusion. Patient tolerated treatment well and had no complaints of discomfort. The patient completed treatment and left stable.

#### Patient reports:

Cravings 0/10; anxiety 3/10; depression 2/10; and hours of uninterrupted sleep 7.

#### Infusion 6:

Patient reports that he has had a decrease in anxiety and an overall increase in his energy and general sense of wellbeing. It has been hard on everyone in the house the last few days with the covid19 and trying to stay inside with roommates and nowhere to really go. He has been trying to remain calm and patient through the process. Patient relaxed in recliner and watched movies during infusion. Patient had no complaints of discomfort. The patient completed treatment and left stable.

#### Patient reports:

Cravings 0/10; anxiety 1/10; depression 0/10; and hours of uninterrupted sleep 3.

#### Infusion 7:

Patient reports that he has been feeling home sick and misses his daughter and family a lot. Reports it’s been tough to be away from them but especially right now with the covid19 going on it makes it extra tough. And also, extra tough to be stuck in the house with a bunch of a roommate and not in his home. Reports that his anxiety has decreased overall but has been slightly elevated with being homesick and stuck inside all day. Patient relaxed in recliner and chatted with others during the infusion. Patients tolerated infusion well and had no complaints of discomfort. The patient completed treatment and left stable.

#### Patient reports:

**Figure 44:**
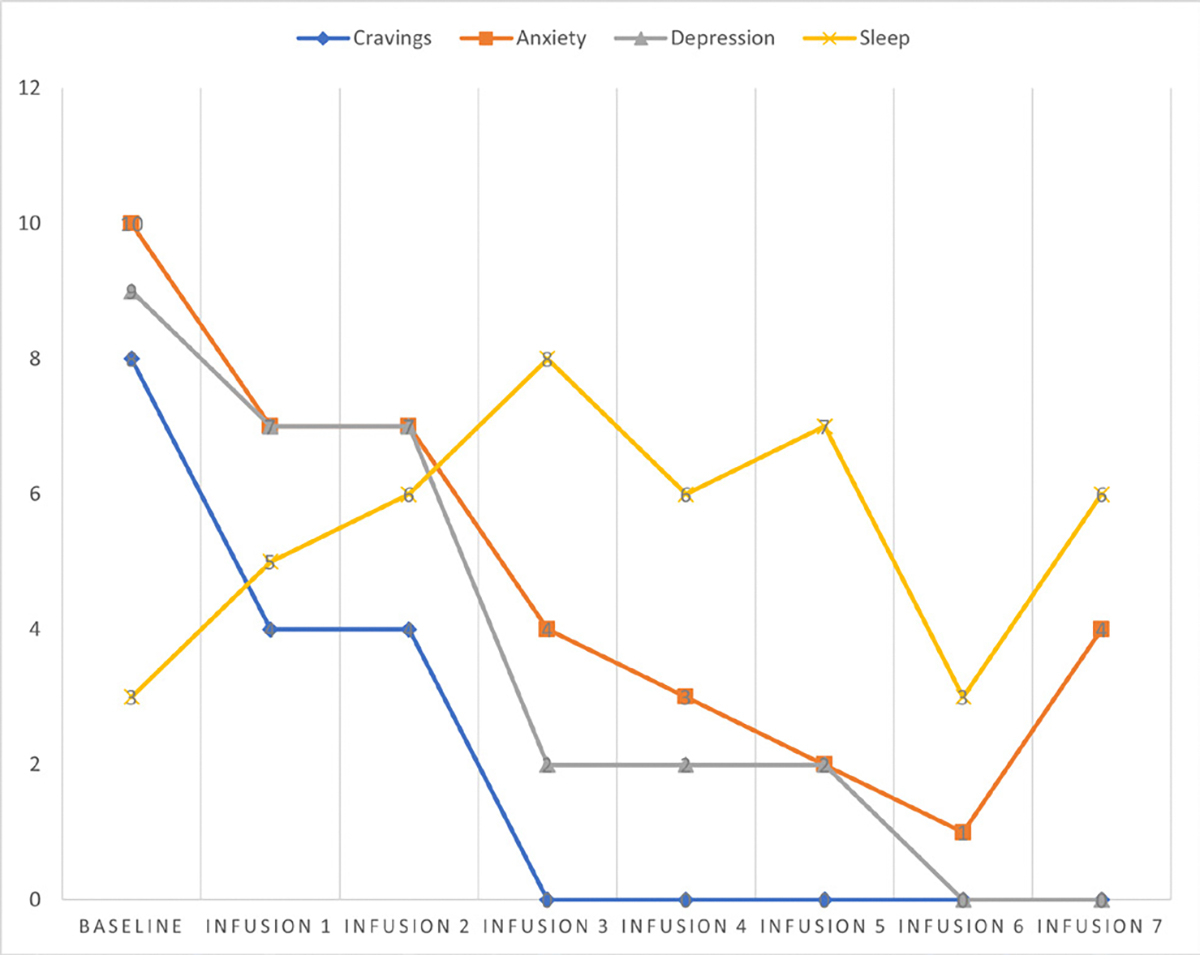
Patient 43 (Male/40/meth).

Cravings 0/10; anxiety 4/10; depression 0/10; and hours of uninterrupted sleep 6 (Table 45 and figure 44).

### Patient 44 (Male/24/heroin/meth)

#### Consultation:

Patient presents to clinic from south coast BH for NAD consultation. DOC is heroin via smoking using 1 – 2 g weekly. Using meth as well. Last use was 2/5, longest sobriety is currently, this is patients first treatment facility. Denies any OD or seizures in the past. Currently “I have a hard time sleeping, it’s hard for me to concentrate daily. I’ve been working through my anxiety daily. I don’t drink any water to hydrate, and I feel it.”

#### Baseline:

Patient reports cravings is an 8 out of 10, patient states he does not have a desire to use but sometimes he has using dreams. Patient reports anxiety and depression is a 5 out of 10, advised patient on ways to work through moods with breathing exercises and grounding techniques advised patients to discuss feelings with case manager.

#### Patient reports:

Cravings 8/10; anxiety 4/10; depression 5/10; and hours of uninterrupted sleep 6.

#### Infusion 1:

Patient reports for infusion treatment reports that he is feeling better than last week. Reports that his sleep and overall energy has improved. Patient reports that he is having no cravings and feels more motivated to stay sober than ever. The patient reports his anxiety has decreased to a 3/10 and feels it is more manageable than last week. Patient relaxed in recliner and chatted with others during infusion. Patient tolerated treatment well and had no complaints of discomfort. The patient completed treatment and left stable.

#### Patient reports:

Cravings 0/10; anxiety 4/10; depression 3/10; and hours of uninterrupted sleep 6.

#### Infusion 2:

Patient reports cravings is a 1 – 2 out of 10, patient states he does not have a desire to use but sometimes he has using dreams. Patient reports anxiety and depression decreased to a 3 out of 10, advised patient on ways to work through moods with breathing exercises and grounding techniques. Advised patient to discuss feelings with case manager. Sleep is around 6 h an evening, patient states he wakes up in the middle of the night and going back to sleep is not an issue. Patient states he feels increased energy and feels rested after sleep.

#### Patient reports:

Cravings 2/10; anxiety 3/10; depression 3/10; and hours of uninterrupted sleep 6.

#### Infusion 3:

Patient reports anxiety and depression decreased to a 3 out of 10, advised patient on ways to work through moods with breathing exercises and grounding techniques. Advised patient to discuss feelings with case manager. Patient reports cravings are a 1 – 2 out of 10, patient states he does not have a desire to use but sometimes he has using dreams. Sleep is around 6 h an evening, patient states he wakes up in the middle of the night and going back to sleep is not an issue. Patient states he feels increased energy and feels rested after sleep.

#### Patient reports:

Cravings 2/10; anxiety 3/10; depression 3/10; and hours of uninterrupted sleep 6.

#### Infusion 4:

Patient reports cravings is a 1 out of 10. Patient reports anxiety and depression decreased to a 3 out of 10, advised patient on ways to work through moods with breathing exercises and grounding techniques. Advised patient to discuss feelings with case manager. Sleep is around 6 h an evening, patient states he wakes up in the middle of the night and going back to sleep is not an issue. Patient states he feels increased energy and feels rested after sleep. IV site dry and intact, patient tolerated infusion well.

#### Patient reports:

Cravings 1/10; anxiety 3/10; depression 3/10; and hours of uninterrupted sleep 6.

#### Infusion 5:

Sleep is around 6 h an evening, patient states he wakes up in the middle of the night and going back to sleep is not an issue. Patient reports anxiety and depression decreased to a 3 out of 10, advised patient on ways to work through moods with breathing exercises and grounding techniques. Advised patient to discuss feelings with case manager. Patient expressed understanding. Patient states he feels increased energy and feels rested after sleep. Patient denies any cravings. IV site dry and intact, patient tolerated infusion well.

#### Patient reports:

Cravings 0/10; anxiety 3/10; depression 3/10; and hours of uninterrupted sleep 6.

#### Infusion 6:

Patient reports anxiety and depression decreased to a 2 out of 10, advised patient on ways to work through moods with breathing exercises and grounding techniques. Advised patient to discuss feelings with case manager. Experiencing no cravings, the patient remains focused on sobriety. Patient states he feels increased energy and feels rested after sleep. IV site dry and intact, patient tolerated infusion well.

#### Patient reports:

Cravings 0/10; anxiety 2/10; depression 2/10; and hours of uninterrupted sleep 6.

#### Infusion 7:

Patient reports 8 h of uninterrupted sleep. Patients do not drink caffeinated beverages. Consumes 48 oz of water daily and am currently not taking medications. Patients currently do not experience cravings for opiates or stimulants. Anxiety and depression are 0 out of 10. Patient expresses he is happy he has been receiving more infusions and believes it has improved his health and well-being.

#### Patient reports:

**Figure 45:**
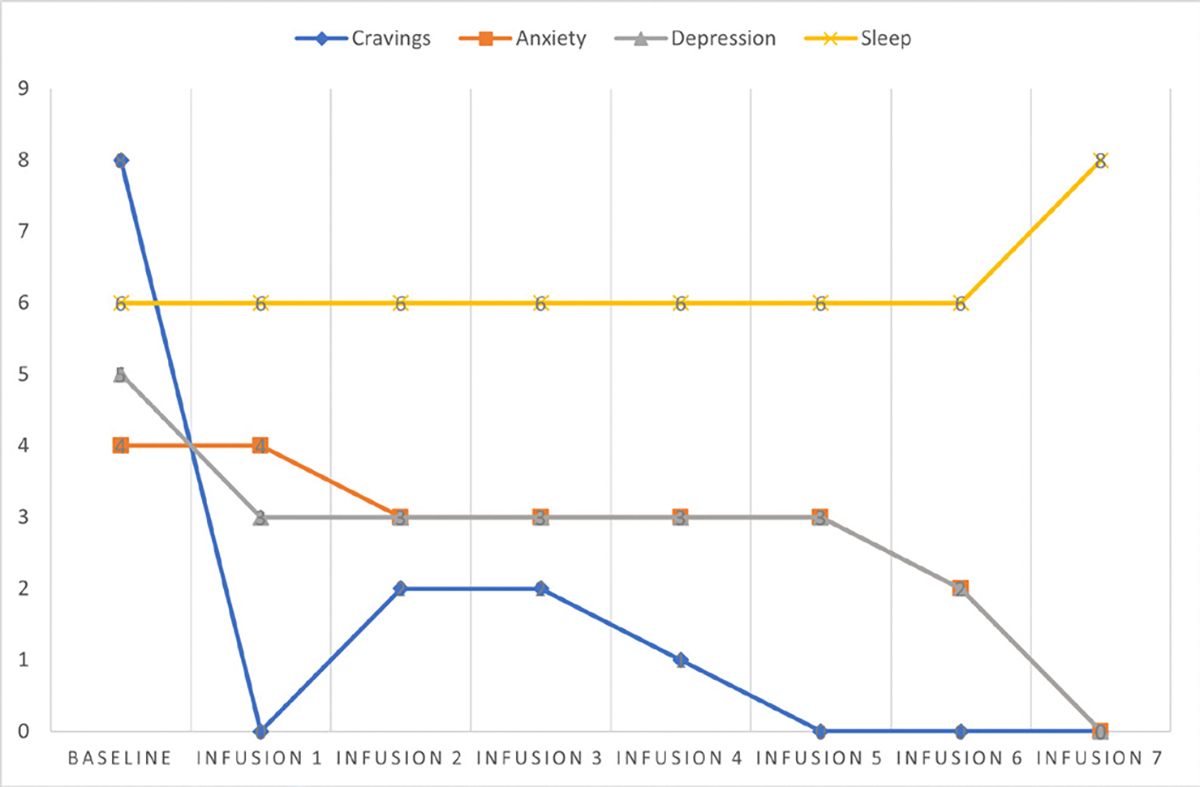
Patient 44 (Male/24/heroin/meth).

Cravings 0/10; anxiety 0/10; depression 0/10; and hours of uninterrupted sleep 8 (Table 46 and figure 45).

### Patient 45 (Male/30/heroin/opiates/benzos)

#### Consultation:

Patient presents to clinic for NAD consultation. DOC is Xanax 10 – 12mg daily, heroin via smoking 3 g a day and oxycontin. Last use 4/1. The longest sobriety is currently 29 days. This is a patient’s 1st treatment facility. Denies any OD or seizures in the past. Currently “I am experiencing high anxiety all day, I still have a lot of energy, last night was the first night I slept. I have cravings throughout the day. I’m noticing +anxiety about getting help for the first time. I am here for the NAD treatments, I researched this and am very excited.”

#### Baseline:

Patient reports to first infusion with complaints of severe anxiety and restless sleep. Encouraged patients to practice meditation and breathing exercises to help ease anxiety. Patient reports cravings are high. Patient was receptive. IV insertion attempted x2 with no success. Advised patients on the importance of hydration. Patient was receptive.

#### Patient reports:

Cravings 7/10; anxiety 7/10; depression 2/10; and hours of uninterrupted sleep 6.

#### Infusion 1:

Patient reports to second infusion with c/o severe anxiety and restless sleep. Patient reports cravings are still high 6/10. Patients report not drinking much water throughout the day. Encouraged patients to hydrate but not too late in the day to avoid frequent awakening to void. Patient was receptive. The patient was observed relaxing on a recliner and watching TV. Patient tolerated treatment well with no complaint of discomfort. The patient completed treatment and was left in stable condition.

#### Patient reports:

Cravings 6/10; anxiety 6/10; depression 0/10; and hours of uninterrupted sleep 6.

#### Infusion 2:

Patient reports feeling more energized and overall wellbeing. Patient reports cravings have decreased 3/10. Patient was observed relaxing during infusion and conversing with peers. Patient tolerated myers and glutathione with no complaints. Patient c/o nausea and over all discomfort 1 h into infusion. The patient was given ondansetron 4 mg 2-tab SL without incidence. Patient reported alleviated nausea. Patients V/S were WNL. Patient left in stable condition.

#### Patient reports:

Cravings 3/10; anxiety 0/10; depression 3/10; and hours of uninterrupted sleep 9.

#### Infusion 3:

Patient reports he is 50 days sober, and he feels amazing and very thankful. Patient expressed that he believes vitamin infusions have been an important tool in his recovery, patient reports he can’t fail himself or everyone who is rooting for him, patient states he didn’t know so many people cared. Patient reports energy is good, and sleep has improved, IV site dry and intact, patient tolerated infusion well.

#### Patient reports:

Cravings 1/10; anxiety 3/10; depression 2/10; and hours of uninterrupted sleep 7.

#### Infusion 4:

Patient expressed that he believes vitamin and NAD infusions have been an important tool in his recovery, patient reports he can’t fail himself or everyone who is rooting for him, patient states he didn’t know so many people cared. Patient denies having any cravings and that it has been years since he’s felt that way. IV site dry and intact, patient tolerated infusion well.

#### Patient reports:

Cravings 0/10; anxiety 1/10; depression 0/10; and hours of uninterrupted sleep 8.

#### Infusion 5:

Patient reports feeling good, slept 8 h last night and not waking up as up at night, patient states he tried to avoid too much liquid prior to bed, and he thinks that helped. Patient states, “more energy and better sleep” improved sleep has put patient in better mood and helped him pay attention and be active in class, IV site dry and intact, patient tolerated infusion well.

#### Patient reports:

Cravings 0/10; anxiety 2/10; depression 0/10; and hours of uninterrupted sleep 8.

#### Infusion 6:

Patient reports he had a good weekend; patient tried his best to stay active this weekend and soak in the sun. Patient experiences anxiety and depression, and he knows it well to get out and soak in vitamin D. Patient states, “better overall wellbeing” IV site dry and intact, patient tolerated infusion well.

#### Patient reports:

Cravings 0/10; anxiety 2/10; depression 0/10; and hours of uninterrupted sleep 8.

#### Infusion 7:

Patient reports feeling good, slept 8 h last night and not waking up as up at night, patient states he tried to avoid too much liquid prior to bed, and he thinks that helped. Patient states, “more energy and better sleep” improved sleep has put patient in better mood and helped him pay attention and be active in class. IV site dry and intact, client tolerated infusion well.

#### Patient reports:

**Figure 46:**
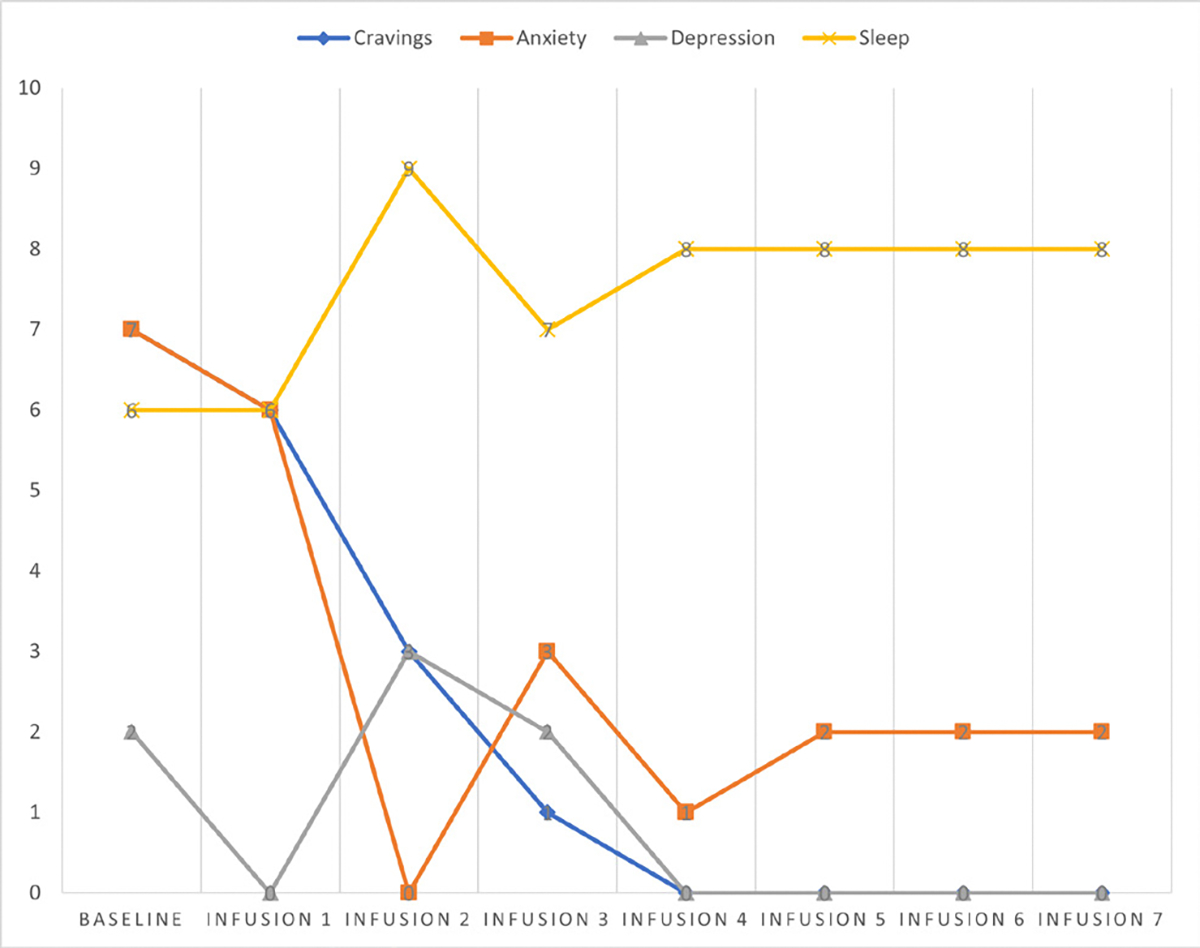
Patient 45 (Male/30/heroin/opiates/benzos).

Cravings 0/10; anxiety 2/10; depression 0/10; and hours of uninterrupted sleep 8 (Table 47 and figure 46).

### Patient 46 (Male/32/heroin)

#### Consultation:

Patient presents to clinic for NAD consultation. DOC is heroin via IV using 1 – 2 g daily. Last use was 4/20. Longest sobriety is currently, and this is patient’s 1st treatment facility. Denies any OD or seizures in the past. Currently “since I stopped using, I have been nauseated and dehydrated. I also have a hard time concentrating and my worst is cravings.”

#### Baseline:

Patient reports to his first infusion. Patient reports sleeping about 8 h a night with the help of medication. Patient reports cravings at an 8/10. Patients report drinking only 24 oz of water daily. Encouraged patients to increase their water intake. Patient was receptive. Patient was observed relaxing on recliner and conversing with peers during infusion. Patient tolerated treatment well with no complaint of discomfort. The patient completed treatment and was left in stable condition.

#### Patient reports:

Cravings 8/10; anxiety 2/10; depression 2/10; and hours of uninterrupted sleep 8.

#### Infusion 1:

Patient reports restless sleep and feeling fatigued throughout the day. Patients report only drinking about 20 oz of water daily. Encouraged patients to increase fluid intake but not too late in the evening to avoid waking up throughout the night and to practice meditation before bed to help relax. Patient was receptive. The patient was observed relaxing on couch with eyes closed during infusion. Patient tolerated treatment well with no complaint of discomfort. The patient completed treatment and was left in stable condition.

#### Patient reports:

Cravings 6/10; anxiety 2/10; depression 0/10; and hours of uninterrupted sleep 4.

#### Infusion 2:

Patient reports feeling more energy since he started infusion treatments. Patient reports increasing his fluid intake. Patient denies having any cravings. The patient was observed relaxing during infusion. Patient tolerated treatment well with no complaint of discomfort. The patient completed treatment and was left in stable condition.

#### Patient reports:

Cravings 0/10; anxiety 0/10; depression 0/10; and hours of uninterrupted sleep 8.

#### Infusion 3:

Patient reports feeling more energy and more alert throughout the day. Patient was observed relaxing on recliner and conversing with peers during infusion. Patient tolerated treatment well with no complaint of discomfort. The patient completed treatment and was left in stable condition.

#### Patient reports:

Cravings 0/10; anxiety 0/10; depression 0/10; and hours of uninterrupted sleep 7.

#### Infusion 4:

Patient reports an increase in his medication to help with his BUE pain. Patient reports he feels the pain all day long. Patient reports getting 8 h of uninterrupted sleep and increasing his water intake. Patient was observed relaxing on recliner during infusion. Patient tolerated treatment well with no complaint of discomfort. Patient completed treatment and left in stable condition.

#### Patient reports:

Cravings 0/10; anxiety 3/10; depression 0/10; and hours of uninterrupted sleep 8.

#### Infusion 5:

Patient reports feeling a decrease in his anxiety levels and is happy that his girlfriend moved into the same house. Patient reports increasing fluids and feeling over all great. Patient was observed listening to music during infusion. Patient tolerated treatment well with no complaint of discomfort. The patient completed treatment and was left in stable condition.

#### Patient reports:

Cravings 0/10; anxiety 3/10; depression 0/10; and hours of uninterrupted sleep 7.

#### Infusion 6:

Patient reports he is feeling good today. Patient states he is happy it is friday and that he now lives in a house with his girlfriend. Patient reports he does not experience cravings for drugs or alcohol. Patient drinks about 2 caffeinated beverages a day and about 64 oz of water, advised patient to increase water intake and educated patient on the importance of water intake, patient expressed understanding. IV site dry and intact, patient tolerated infusion well.

#### Patient reports:

Cravings 0/10; anxiety 0/10; depression 0/10; and hours of uninterrupted sleep 8.

#### Infusion 7:

Patient reports feeling okay. Patient reports girlfriend broke up with him and he is feeling very down. Patient states he does not know how he feels. Client appears to lose focus often, lack of focus is evidenced by the need to repeat questions and comments because patient claims he did not get it, a lot of “huh?” questions from patients. Patient claims his cravings are about a 1 out of 10, advised patient to discuss with case manager, patient expressed understanding. And anxiety 2 out of 10, advised patient to discuss with therapist and to practice meditation and breathing exercises, patient states he understands. IV site dry and intact, patient tolerated infusion well.

#### Patient reports:

**Figure 47:**
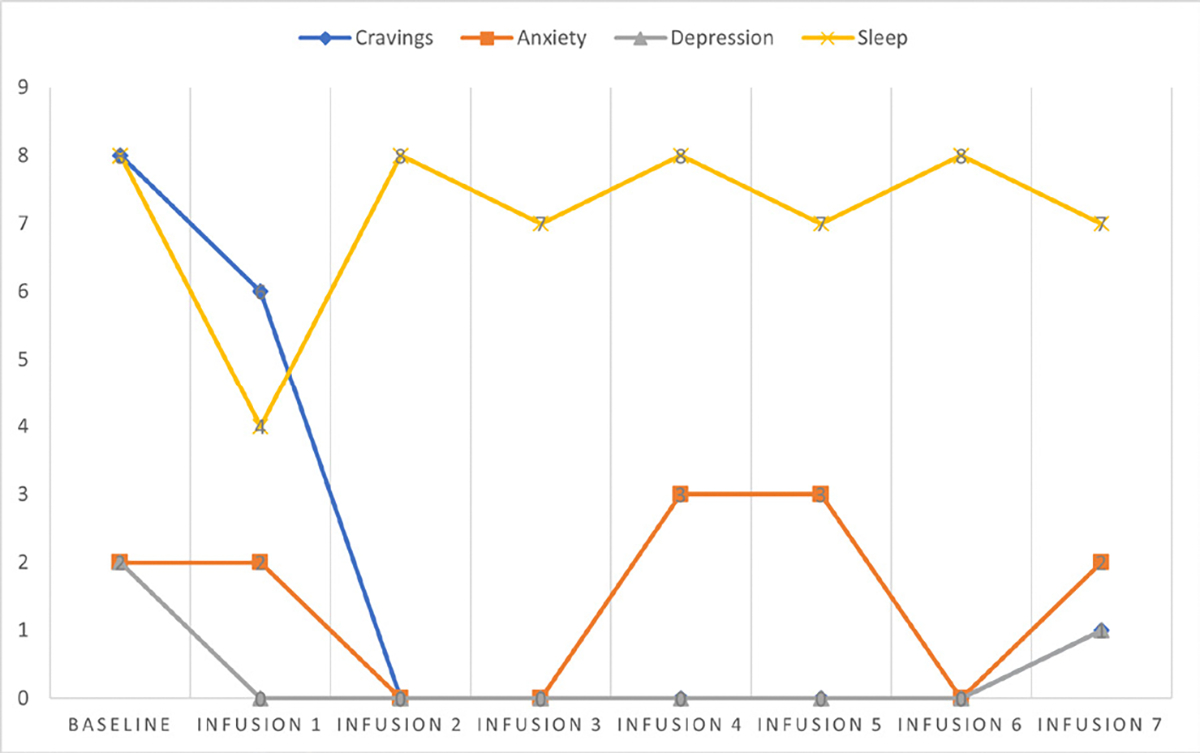
Patient 46 (Male/32/heroin).

Cravings 1/10; anxiety 2/10; depression 1/10; and hours of uninterrupted sleep 7 (Table 48 and figure 47).

### Patient 47 (Male/46/meth)

#### Consultation:

Patient is being seen for NAD consultation. + OD and seizures in the past. DOC is meth via smoking and IV 1 – 2 g daily. Last use was 3/7, longest sobriety is 2 years, and this is patient’s 1st treatment facility. Currently with “hot flashes, cramping and chills. Denies any chest pain, no sob, no abdominal pain, no n/v/d. I am hurting all over and have really bad cravings. I can’t drink enough water and keep my anxiety under control, I’m also very irritable and can’t concentrate.”

#### Baseline:

Patient reports for second infusion and hasn’t noticed much of a change. Reports that he did wake up at 3 and instead of lying there got up and did his daily chores. Feels depleted and overall fatigue. Patient reports cravings at a 5/10. Patient reports that his anxiety is still high, and he feels bored and stuck in the house. Patient relaxed in recliner and chatted with others during infusion. Patients tolerated infusion well and had no complaints of discomfort. The patient completed treatment and left stable.

#### Patient reports:

Cravings 5/10; anxiety 8/10; depression 5/10; and hours of uninterrupted sleep 4.

#### Infusion 1:

Patient reports that he slept on the couch last night and seemed to get better sleep. Reports that he sleeps on the bottom bunk and his roommate wakes a lot throughout the night and wakes him. He has noticed an increase in libido as prior to the treatments he was struggling with ED. Patient has been still struggling with anxiety and depression, but reports it has a lot to do with the work he has been doing with his therapist and his loss of his son. Patient reports they have been changing his BP meds to try to bring it down to the correct rate. Patient relaxed in recliner and watched movies during infusion. Patient tolerated treatment well and had no complaints of discomfort. The patient completed treatment and left stable.

#### Patient reports:

Cravings 3/10; anxiety 3/10; depression 5/10; and hours of uninterrupted sleep 4.

#### Infusion 2:

Patient reports that his tremors have decreased, and his libido is still very elevated. Patient reports that his anxiety and depression are getting better by the day, trying to take his treatment one day at a time and progress through the past pains of his sons’ loss so he can progress forward and continue to make positive changes. Patient reports that the treatment center is still adjusting his BP meds, and monitoring is daily. Patient reports that he got 7 h of sleep and that it is slowly getting better and better. Patient denies any cravings or feelings to use. Patient relaxed in recliner and chatted with others during infusion. Patient tolerated treatment well and had no complaints of discomfort. The patient completed treatment and left stable.

#### Patient reports:

Cravings 0/10; anxiety 5/10; depression 5/10; and hours of uninterrupted sleep 7.

#### Infusion 3:

Patient reports that he awoke energized this morning. Patient reports that his anxiety and depression are still decreasing daily and denies having any cravings. Patient reports that he has been doing a lot of work with his therapist and is making every effort to work through all of his pains to help strengthen him to put his addictions behind him. Patient reports he is feeling better each day. Patient relaxed in recliner and chatted with others during infusion. Patients tolerated infusion well and had no complaints of discomfort. The patient completed treatment and left stable.

#### Patient reports:

Cravings 0/10; anxiety 2/10; depression 5/10; and hours of uninterrupted sleep 7.

#### Infusion 4:

Patient reports feeling depressed today and not having much of an appetite. The patient does report a decrease in his tremors and having a good night’s rest. The patient was observed relaxing in recliner and watching TV during infusion. Patient tolerated treatment well with no complaints of discomfort. The patient completed treatment and was left in stable condition.

#### Patient reports:

Cravings 0/10; anxiety 4/10; depression 7/10; and hours of uninterrupted sleep 7.

#### Infusion 5:

Patient reports an overall increase in sense of well-being. Patient reports increasing his fluid intake and trying to make healthier choices. Patient reports an increase with his anxiety and relates it to therapy. Patient denies any cravings or desire to use. The patient was conversing with peers and relaxing in recliner during infusion. Patient tolerated treatment well with no complaints of discomfort. Patient completed treatment with and left in stable condition.

#### Patient reports:

Cravings 0/10; anxiety 3/10; depression 5/10; and hours of uninterrupted sleep 6.

#### Infusion 6:

Patient reports having had a difficult time falling asleep due to having back and shoulder pain, encouraged patients to perform stretches before going to bed. Patient was observed relaxing on recliner and watching TV during infusion. Patient tolerated treatment well with no complaints of discomfort. The patient completed treatment and was left in stable condition.

#### Patient reports:

Cravings 0/10; anxiety 3/10; depression 5/10; and hours of uninterrupted sleep 7.

#### Infusion 7:

Patient reports back pain has improved which has improved client’s sleep patterns. The patient said he had difficulty sleeping due to back pain and he would wake up in the morning feeling tired and drained. The patient reports energy levels have improved, and he is trying to stay active. The patient denies having any cravings and reports he is doing a lot better emotionally with the loss of his son. IV site dry and intact, client tolerated infusion well.

#### Patient reports:

**Figure 48:**
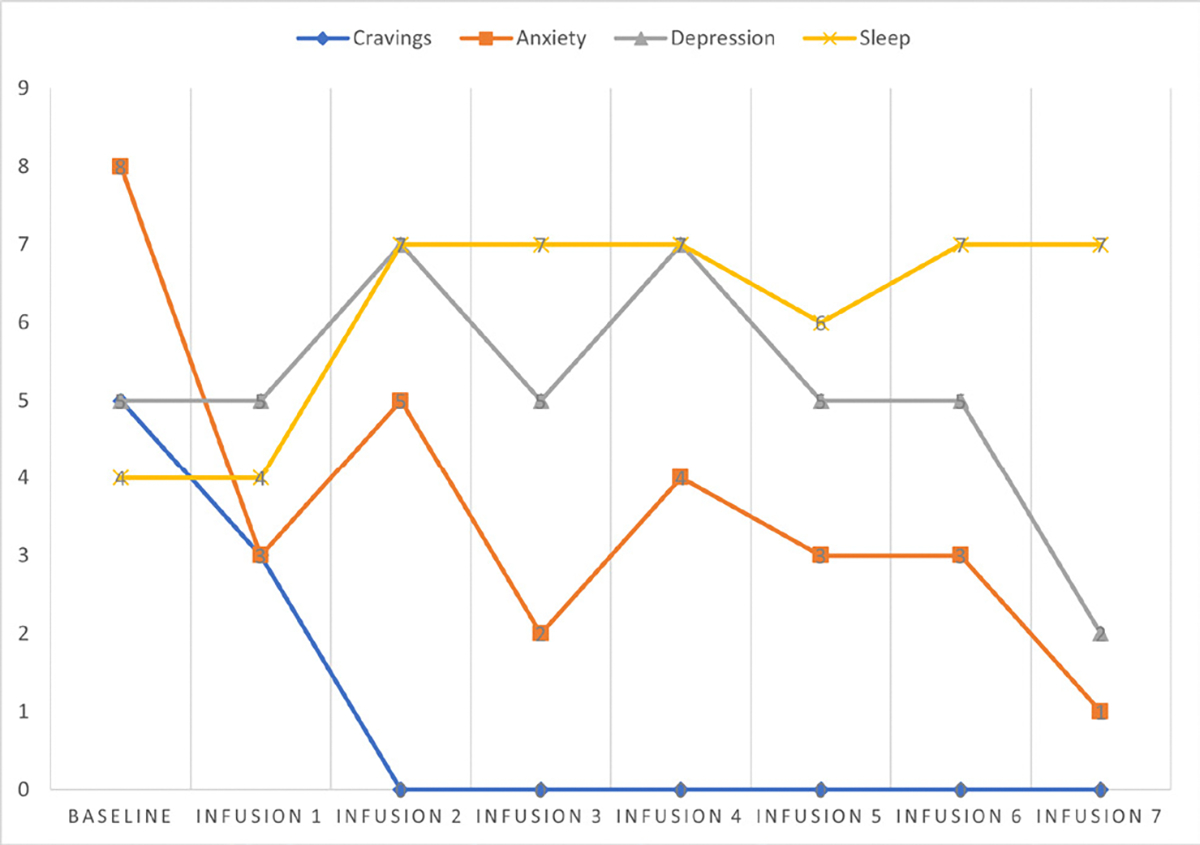
Patient 47 (Male/46/meth).

Cravings 0/10; anxiety 1/10; depression 2/10; and hours of uninterrupted sleep 7 (Table 49 and figure 48).

### Patient 48 (Male/45/ETOH)

#### Consultation:

Patient presents to clinic for NAD consultation. DOC is alcohol drinking 1L of vodka a day/rum. Last drink was 2/16. The longest sobriety is 1.5 years in 2015. Has been to 8 treatment facilities. Denies any seizures in the past. Currently “I have pretty much everything fatigue, and the anxiety is the worse and it keeps me awake at night. Today is the first day I have been able to eat a little.” Denies any abdominal pain, no chest pain. Denies any cravings.

#### Baseline:

Patient reports cravings are currently a 5 of 10 with depression/anxiety a 3 of 10, which he reports as “mild” in comparison to an average day. Discussed benefits of infusions and encouraged him to stay consistent with the doctor’s order so that he may benefit from them the greatest. Tolerated Myers well without discomfort and left stable with no complications

#### Patient reports:

Cravings 5/10; anxiety 3/10; depression 5/10; and hours of uninterrupted sleep 5.

#### Infusion 1:

Patient reports cravings at level 4 of 10, anxiety and depression at level 5 of 10 due to being away from home and family and being in treatment sharing a home with house mates. Advised different ways to treat anxiety and depression such as exercise and meditation. Sleeps for 7 h a day. Mental clarity and energy levels are low. Looking forward to having no cravings. Tolerated treatment well with no pain or discomfort.

#### Patient reports:

Cravings 4/10; anxiety 5/10; depression 5/10; and hours of uninterrupted sleep 7.

#### Infusion 2:

Patient reports less anxiety less than he had prior to infusion but still periodically present. Discussed ways to help anxiety (meditation, breathing exercises, etc.) and patient understood. Tolerated Tx well with no discomfort and left stable with no complications.

#### Patient reports:

Cravings 3/10; anxiety 2/10; depression 3/10; and hours of uninterrupted sleep 7.

#### Infusion 3:

Patient reports no cravings, anxiety at level 4 of 10 due to having to move houses soon. No depression advised different ways to treat anxiety and depression such as exercise and meditation. Patients sleep for 6 h a day. Mental clarity and energy levels have increased since starting infusions. Tolerated treatment well with no pain or discomfort.

#### Patient reports:

Cravings 0/10; anxiety 4/10; depression 0/10; and hours of uninterrupted sleep 6.

#### Infusion 4:

Patient reports no cravings, anxiety or depression at level 4 of 10 due to being in treatment and feeling homesick. Advised different ways to treat anxiety and depression such as exercise and meditation. Sleeps for 7 h a day. No changes in energy levels or mental clarity yet no pain. Tolerated treatment well with no pain or discomfort.

#### Patient reports:

Cravings 0/10; anxiety 4/10; depression 4/10; and hours of uninterrupted sleep 7.

#### Infusion 5:

Patient reports feeling more rested lately as he’s been able to sleep better than when he first started infusions. Noticed more energy throughout the day which helps decrease brain fog. Tolerated Tx well without discomfort and left stable with no complications

#### Patient reports:

Cravings 0/10; anxiety 3/10; depression 3/10; and hours of uninterrupted sleep 5.

#### Infusion 6:

Patient reports no cravings, anxiety and depression at level 4 of 10 mostly due to feeling homesick and getting used to having new roommates constantly. Advised different ways to reduce anxiety and depression such as exercise and meditation. Sleeps for 7 h a day. No pain no changes in mental clarity or energy levels yet. Tolerated treatment well with no pain or discomfort.

#### Patient reports:

Cravings 0/10; anxiety 4/10; depression 4/10; and hours of uninterrupted sleep 7.

#### Infusion 7:

Patient reports that he has felt a lot better in his recovery process this time with the treatments. Feels less depleted and overall detox has improved. Patient reports that his energy and sleep have also improved. The patient reports that he is able to think better, and his overall brain fog has gone away. Patient relaxed and watched movies during the infusion. Patient tolerated treatment well and had no complaints of discomfort. The patient completed treatment and left stable.

#### Patient reports:

**Figure 49:**
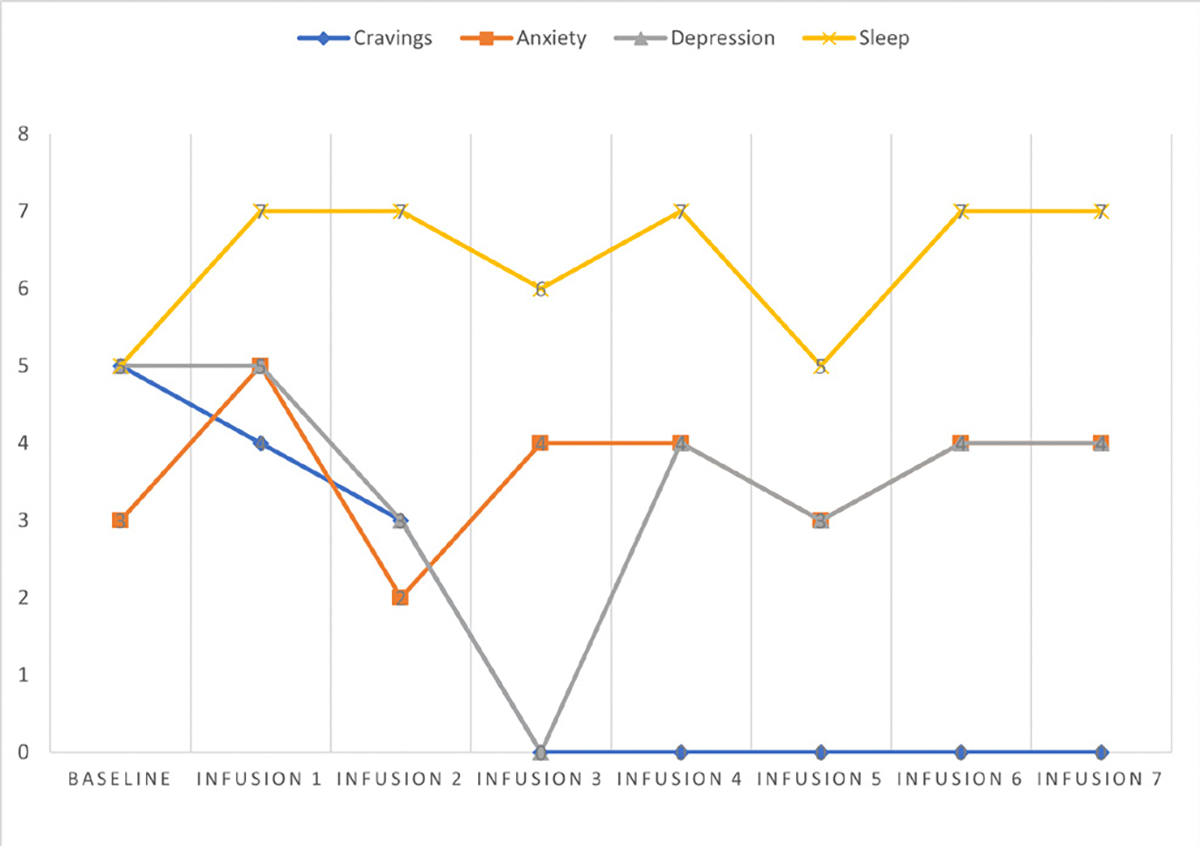
Patient 48 (Male/45/ETOH).

Cravings 0/10; anxiety 4/10; depression 4/10; and hours of uninterrupted sleep 7 (Table 50 and figure 49).

### Patient 49 (Male/28/meth/ETOH)

#### Consultation:

Patient presents to clinic for NAD consultation. DOC is meth via smoking and alcohol drinking 2x a week. Last use was 40 days ago. Denies any OD or seizures in the past. The longest sobriety is 9 months in 2018, and this is patient’s 1^st^ treatment facility. Currently states” I have a lot of irritability and I’m really tired but manageable. I’m severely dehydrated. I also get severe anxiety, and I was placed on anxiety meds at a high dose.”

#### Baseline:

Patient reports that he quit smoking 3 days ago and that he has been doing well with it. Patient reports that he has been sober for a month now and feels more spiritual and in-tune with his needs and the things that have pushed him down the wrong path. Patient reports that he has been sleeping well and working on healthier habits including nutrition, exercise and hydration. Patient reports his anxiety is general and comes and goes with the work he has been doing in group. Patient relaxed in recliner and chatted with others during infusion. Patient tolerated treatment well and had no complaints of discomfort. The patient completed treatment and left stable.

#### Patient reports:

Cravings 8/10; anxiety 3/10; depression 0/10; and hours of uninterrupted sleep 8.

#### Infusion 1:

Patient reports getting good sleep and decrease in anxiety. Patient reports having a good appetite and working on making healthier choices for his wellbeing. The patient was observed relaxing and watching TV during infusion. Patient tolerated treatment well with no complaints of discomfort. The patient completed treatment and was left in stable condition.

#### Patient reports:

Cravings 3/10; anxiety 2/10; depression 0/10; and hours of uninterrupted sleep 8.

#### Infusion 2:

Patient reports cognitive functions have improved he is able to concentrate better, and his mood is more stable. Patient denies having any cravings. Feels really good. Patient was observed on recliner watching TV and conversing with peers during infusion. Patient tolerated treatment well with no complaints of discomfort. The patient completed treatment and was left in stable condition.

#### Patient reports:

Cravings 0/10; anxiety 0/10; depression 0/10; and hours of uninterrupted sleep 8.

#### Infusion 3:

Patient reports feeling improved energy levels and getting good sleep. The patient was observed relaxing on couch and listening to music during infusion. Patients tolerated treatment with no complaints of discomfort. Patient completed treatment and left in stable condition

#### Patient reports:

Cravings 0/10; anxiety 0/10; depression 0/10; and hours of uninterrupted sleep 8.

#### Infusion 4:

Patient reports that he is feeling better and better each day. Reports that he is really focusing on his selfish ways and trying to really look inside of himself. He has been working on his anger and things that easily trigger him and he feels really good about making positive changes in his person. Patient reports that he has noticed that he is able to cope with things better since starting the infusions. Patient tolerated treatment well and had no complaints of discomfort. The patient completed treatment and left stable.

#### Patient reports:

Cravings 0/10; anxiety 0/10; depression 0/10; and hours of uninterrupted sleep 8.

#### Infusion 5:

Patient reports that he has been sleeping better and feeling more energetic and positive overall. Patient reports that he has been practicing mindfulness and overall positive thinking and feels like it has been working on his old selfish behaviors. Patient relaxed in recliner and chatted with others during infusion. Patient tolerated treatment well and had no complaints of discomfort. The patient completed treatment and left stable.

#### Patient reports:

Cravings 0/10; anxiety 0/10; depression 0/10; and hours of uninterrupted sleep 8.

#### Infusion 6:

Patient reports “going great, feeling good” patient states he is very focused on meditation and self-healing. Patient reports that he feels grateful for infusions because it has enhanced his meditation and mind power. Patient reports he feels clear minded and seems like he retains information better. IV site dry and intact, patient tolerated infusion well.

#### Patient reports:

Cravings 0/10; anxiety 1/10; depression 0/10; and hours of uninterrupted sleep 8.

#### Infusion 7:

Patient reports “I seem to be doing better” patient states he is very focused on meditation and self-healing. Patient seems to really value infusion therapy as an essential tool to his overall healing and well-being. Patient reports having researched a lot of vitamin infusions and value the benefits he is receiving. IV site dry and intact, patient tolerated infusion well.

#### Patient reports:

**Figure 50:**
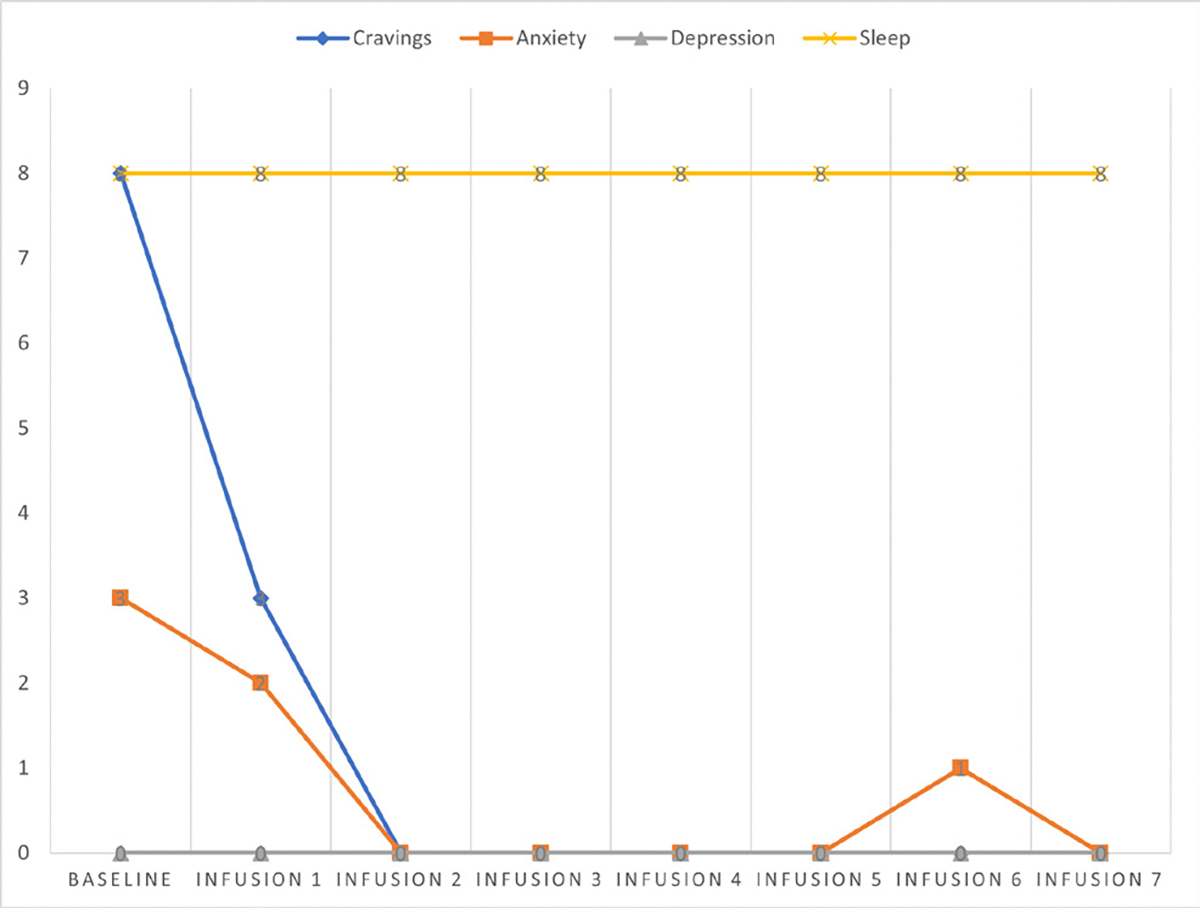
Patient 49 (Male/28/meth/ETOH).

Cravings 0/10; anxiety 0/10; depression 0/10; and hours of uninterrupted sleep 8 (Table 51 and figure 50).

### Patient 50 (Male/33/heroin)

#### Consultation:

Patient presents to clinic for NAD consultation. DOC is heroin. via IV. Last use was 4/21, Longest sobriety is 1.5 years in 2015. Has been to 8 treatment facilities. Denies any seizures in the past. Currently “ I have pretty much everything fatigue, and the anxiety is the worse and it keeps me awake at night. Today is the first day I have been able to eat a little.” Denies any abdominal pain, no chest pain. Denies any cravings.

#### Baseline:

Patient reports today for infusion therapy and seems to be in good spirits. C/O mild back pain and cravings still very high 9/10. Patient reports wanting to stay sober really badly and not having cravings anymore. Wants his life back. Patient tolerated infusion well. Reports she feels a lot more hydrated. The patient completed the infusion and left stable.

#### Patient reports:

Cravings 9/10; anxiety 2/10; depression 0/10; and hours of uninterrupted sleep 7.

#### Infusion 1:

Patient reports high levels of activity, patient states since the last infusions “more energy and endurance and improved focus” patient reports his workout after infusions was “amazing, I felt amped” reports, “I also for the first time haven’t had a single craving. IV site dry and intact, patient tolerated infusion well.

#### Patient reports:

Cravings 0/10; anxiety 2/10; depression 0/10; and hours of uninterrupted sleep 7.

#### Infusion 2:

Patient reports increased energy levels especially during work outs. Patient reports feeling calm and relaxed more often. Patient was observed relaxing on recliner and talking with peers during infusion. Patient tolerated treatment well with no complaint of discomfort. The patient completed treatment and was left in stable condition.

#### Patient reports:

Cravings 0/10; anxiety 0/10; depression 0/10; and hours of uninterrupted sleep 7.

#### Infusion 3:

Patient reports “high energy levels, improved focus and lowered my anxiety” patient states he tries his best to stay active and workouts out every day, reports infusions have helped him with his performance level. Patients drink about 1 – 2 caffeinated beverages daily and about 120 oz of water. IV site dry and intact, patient tolerated infusion well.

#### Patient reports:

Cravings 0/10; anxiety 3/10; depression 0/10; and hours of uninterrupted sleep 8.

#### Infusion 4:

Patient reports having higher levels of energy throughout the day. Patient reports feeling great with a little anxiety related to him moving from his apartment. The patient was observed relaxing on recliner during infusion. Patient tolerated treatment well with no complaint of discomfort. The patient completed treatment and was left in stable condition.

#### Patient reports:

Cravings 0/10; anxiety 2/10; depression 0/10; and hours of uninterrupted sleep 8.

#### Infusion 5:

Patient reports feeling good. Patient states he likes the infusions because they have been helping him improve his work outs, patient states he stays active and tries to work out every day. Patient denies any cravings. Patient drinks about a gallon of water daily and tries his best to stay hydrated. Sleep has been okay, sleeps about 6 – 7 h an evening and sometimes wakes up to use the restroom, advised patient to avoid liquids close to bedtime, patient expressed understanding. IV site dry and intact, patient tolerated infusion well.

#### Patient reports:

Cravings 0/10; anxiety 2/10; depression 0/10; and hours of uninterrupted sleep 8.

#### Infusion 6:

Patient reports feeling tired because he had a busy weekend moving from apartments. Patient reports an overall wellbeing. The patient was observed relaxing on recliner and working on his laptop during his infusion. Patient tolerated treatment well with no complaint of discomfort. The patient completed treatment and was left in stable condition.

#### Patient reports:

Cravings 0/10; anxiety 2/10; depression 0/10; and hours of uninterrupted sleep 7.

#### Infusion 7:

Patient reports that he has been feeling better than he ever has. Denies any desire to use or any cravings. Feels very motivated and it still working out. Feels like a brand new him or how he was before drugs. Patient reports anxiety 2/10, mostly situational and being roommates. Patients tolerated infusions well and had no complaints of discomfort.

#### Patient reports:

Cravings 0/10; anxiety 2/10; depression 0/10; and hours of uninterrupted sleep 7 (Table 52).

## Discussion

The present study relates to a method to treat and detoxify patients with SUD utilizing a series of NAD+ and enkephalinase (NADASEASE) infusions in subjects attending chemical dependency programs [5–49]. The primary objective of the current investigation is to provide some additional clinical evidence to show that NAD+ other amino acids including d-phenylalanine, glycine and alanyl glutamine dipeptide and Myers cocktail (B complex) infusions significantly attenuates substance craving behavior and concomitant psychiatric burden sequalae in poly-drug abusers attending both in-patient and out-patient level of care in a number of chemical dependency programs in orange country. In a detailed article submitted elsewhere, using wilcoxon signed rank tests and sign tests, we found the following significance comparing the baseline scores to post outcome scores after NADASE infusions; craving scores (P = 1.063E-9); anxiety (P = 5.487E-7); and depression (P = 1.763E-4). There was an improvement trend in the number of sleep hours post infusions, it was non-significant (Pre 6.28, post 7.34). Importantly, urine analysis of a standard panel of illicit drugs of abuse during the course of NAD+ infusions resulted in a subset of 40 patients tested at midway during infusions 100% of these patients tested negative.

## Conclusion

These robust annotated pilot results reveal the significant positive effects of especially NAD+ amino acid and enkephalinase inhibition infusions in treatment resistant probands showing reward deficiency and provides the rationale to extend these seemingly clinically relevant findings in extended futurist investigations utilizing NAD/NADH alone coupled with the GARS test as a DNA guided precision matched pro-dopamine regulator to help induce required “dopamine homeostasis.”

## Figures and Tables

**Table 1:** Detail of infusions per session.

	Infusion 1	Infusion 2	Infusion 3	Infusion 4	Infusion 5	Infusion 6	Infusion 7
Hydration multi-vitamin immune support with amino acids	X	X	X	X	-	-	-
Hydration multi-vitamin immune support	-	-	-	-	X	X	X
Alpha lipoic acid	X	X	-	-	-	-	-
Glutathione	-	-	X	X	X	X	X
NAD+	X	X	X	X	-	-	-
NAD+ with amino acids	-	-	-	-	X	X	X

**Table 2:** Demographics of the fifty patients in this cohort.

Gender	29 male (58%), 21 female (42%)
Age (years)	34.48 ± 7.46 (range 21 to 61 years)
Drug of choice (DOC)	3 air duster (6%), 8 benzodiazepines (16%), 1 cocaine (2%), 1 crack (2%), 16 ethanol (32%), 16 heroin (32%), 4 marijuana (8%), 38 methamphetamine (76%), 5 opiates (10%), 1 suboxone (2%), one substance: 22 (44%), 2 substances: 17 (34%), 3 substances: 8 (16%), and 4 substances: 3 (6%)
Days since l Last Use	21.76 ± 15.28 (range 1 to 80 days)
Level of care	11 detox (22%), 4 IOP (8%), 12 PHP (24%), and 23 residential (46%)
No. treatment facilities	1 facility: 32 (64%), 2 facilities: 6 (12%), 3 facilities: 5 (10%), 4 facilities: 2 (4%), 5 facilities: 1 (2%), 7 facilities: 1 (2%), 8 facilities: 2 (4%), and 10 facilities: 1 (2%)
Longest sobriety (years)	1.18 ± 1.41 (range 4 days to 7 years)
Years of use	16.80 ± 6.83 (range 5 to 33 years)
Random UA screening	40 negative UASO (80%)
Ethnicity	2 African American (4%), 1 Arabic (2%), 42 Caucasian (84%), 1 Hispanic (2%), and 4 Natives American (8%)

**Table 3:** Patient 1 (Female/42/meth/benzos).

Report	Baseline	Infusion 1	Infusion 2	Infusion 3	Infusion 4	Infusion 5	Infusion 6	Infusion 7
**Cravings**	6	4	0	0	0	0	0	0
**Anxiety**	2	2	0	0	0	0	0	0
**Depression**	2	0	0	0	0	0	0	0
**Hours of sleep**	7	7	7	6	8	8	8	7

**Table 4:** Patient 2 (Female/35/meth/ETOH/duster).

Report	Baseline	Infusion 1	Infusion 2	Infusion 3	Infusion 4	Infusion 5	Infusion 6	Infusion 7
Cravings	7	6	3	3	2	0	0	0
Anxiety	8	8	6	6	7	4	6	4
Depression	5	7	7	2	3	0	3	3
Hours of sleep	5	6	5	4	4	5	5	5

**Table 5:** Patient 3 (Female/27/meth).

Report	Baseline	Infusion 1	Infusion 2	Infusion 3	Infusion 4	Infusion 5	Infusion 6	Infusion 7
Cravings	5	2	0	0	0	0	0	0
Anxiety	6	2	0	0	0	0	1	1
Depression	0	0	0	0	0	1	1	1
Hours of sleep	8	9	8	8	9	8	8	8

**Table 6:** Patient 4 (Female/41/ETOH).

Report	Baseline	Infusion 1	Infusion 2	Infusion 3	Infusion 4	Infusion 5	Infusion 6	Infusion 7
Cravings	5	0	0	1	0	0	0	0
Anxiety	6	0	3	2	3	2	0	0
Depression	6	2	0	0	3	0	2	2
Hours of sleep	8	7	5	7	7	8	6	8

**Table 7:** Patient 5 (Female/45/meth).

Report	Baseline	Infusion 1	Infusion 2	Infusion 3	Infusion 4	Infusion 5	Infusion 6	Infusion 7
Cravings	8	2	0	0	0	0	0	1
Anxiety	0	2	0	2	0	0	0	4
Depression	0	2	0	0	0	0	0	0
Hours of sleep	7	5	5	5	5	5	5	6

**Table 8:** Patient 6 (Female/45/meth/marijuana/duster/ETOH).

Report	Baseline	Infusion 1	Infusion 2	Infusion 3	Infusion 4	Infusion 5	Infusion 6	Infusion 7
Cravings	10	2	2	0	0	0	0	0
Anxiety	6	3	2	0	0	0	0	0
Depression	4	0	0	0	0	0	0	0
Hours of sleep	3	8	8	8	8	8	8	8

**Table 9:** Patient 7 (Female/29/meth/opiates/benzos).

Report	Baseline	Infusion 1	Infusion 2	Infusion 3	Infusion 4	Infusion 5	Infusion 6	Infusion 7
Cravings	5	3	0	0	2	0	0	0
Anxiety	5	4	3	0	0	0	3	3
Depression	1	0	0	0	0	0	0	0
Hours of sleep	9	6	5	6	6	8	8	8

**Table 10:** Patient 8 (Female/28/suboxone).

Report	Baseline	Infusion 1	Infusion 2	Infusion 3	Infusion 4	Infusion 5	Infusion 6	Infusion 7
Cravings	8	5	0	0	0	0	0	0
Anxiety	2	2	0	0	0	6	2	0
Depression	0	0	0	0	0	6	3	3
Hours of sleep	3	8	8	8	7	8	8	7

**Table 11:** Patient 9 (Female/38/meth).

Report	Baseline	Infusion 1	Infusion 2	Infusion 3	Infusion 4	Infusion 5	Infusion 6	Infusion 7
**Cravings**	6	0	0	0	0	0	0	0
**Anxiety**	7	2	2	3	0	0	0	0
**Depression**	0	1	2	0	0	0	0	0
**Hours of sleep**	10	8	7	8	8	8	8	8

**Table 12:** Patient 10 (Female/32/meth/opiates/benzos).

Report	Baseline	Infusion 1	Infusion 2	Infusion 3	Infusion 4	Infusion 5	Infusion 6	Infusion 7
Cravings	4	1	1	0	0	4	0	0
Anxiety	6	2	1	0	3	3	0	0
Depression	1	1	1	0	0	3	0	0
Hours of sleep	8	8	4	6	6	5	6	8

**Table 13:** Patient 11 (Female/34/meth/heroin/ETOH).

Report	Baseline	Infusion 1	Infusion 2	Infusion 3	Infusion 4	Infusion 5	Infusion 6	Infusion 7
Cravings	5	2	4	0	3	0	4	5
Anxiety	4	3	7	3	5	3	4	4
Depression	0	0	2	3	2	0	1	3
Hours of sleep	4	8	8	8	9	3	10	8

**Table 14:** Patient 12 (Female/32/meth, ETOH, and heroin).

Report	Baseline	Infusion 1	Infusion 2	Infusion 3	Infusion 4	Infusion 5	Infusion 6	Infusion 7
Cravings	6	0	0	0	0	0	0	0
Anxiety	8	4	3	4	6	8	0	0
Depression	0	0	0	0	0	3	0	0
Hours of sleep	6	9	10	2	8	8	10	6

**Table 15:** Patient 13 (Female/21/meth/benzos).

Report	Baseline	Infusion 1	Infusion 2	Infusion 3	Infusion 4	Infusion 5	Infusion 6	Infusion 7
**Cravings**	6	4	4	4	3	3	3	0
Anxiety	5	2	7	7	7	7	7	10
Depression	5	1	7	7	7	5	5	9
Hours of sleep	6	8	5	5	6	6	6	7

**Table 16:** Patient 14 (Female/61/opiates/meth).

Report	Baseline	Infusion 1	Infusion 2	Infusion 3	Infusion 4	Infusion 5	Infusion 6	Infusion 7
Cravings	4	4	4	0	0	0	0	0
Anxiety	6	4	7	7	3	3	0	2
Depression	2	0	3	6	0	0	0	0
Hours of sleep	4	5	5	6	5	8	8	8

**Table 17:** Patient 15 (Female/29/meth).

Report	Baseline	Infusion1	Infusion 2	Infusion 3	Infusion 4	Infusion 5	Infusion 6	Infusion 7
Cravings	1	0	0	0	7	0	2	2
Anxiety	3	1	1	1	2	0	2	2
Depression	3	2	2	2	2	0	1	1
Hours of sleep	7	8	8	8	8	10	9	9

**Table 18:** Patient 16 (Female/28/meth/ETOH).

Report	Baseline	Infusion 1	Infusion 2	Infusion 3	Infusion 4	Infusion 5	Infusion 6	Infusion 7
Cravings	9	5	5	2	0	2	2	0
Anxiety	10	8	8	6	4	4	0	1
Depression	10	8	8	6	3	3	0	1
Hours of sleep	4	4	4	4	4	4	7	7

**Table 19:** Patient 17 (Female/36/meth/cocaine).

Report	Baseline	Infusion 1	Infusion 2	Infusion 3	Infusion 4	Infusion 5	Infusion 6	Infusion 7
Cravings	3	0	0	0	0	0	0	0
Anxiety	5	1	2	2	2	3	3	3
Depression	4	1	0	2	0	1	4	4
Hours of sleep	6	8	7	7	8	8	8	8

**Table 20:** Patient 18: (Female/30/heroin).

Report	Baseline	Infusion 1	Infusion 2	Infusion 3	Infusion 4	Infusion 5	Infusion 6	Infusion 7
Cravings	8	7	2	0	0	0	0	0
Anxiety	6	6	0	0	2	0	0	0
Depression	0	2	0	0	0	0	0	0
Hours of sleep	8	6	6	6	7	8	9	9

**Table 21:** Patient 19: (Female/29/meth benzos/ETOH/marijuana).

Report	Baseline	Infusion 1	Infusion 2	Infusion 3	Infusion 4	Infusion 5	Infusion 6	Infusion 7
Cravings	4	4	0	0	1	0	0	0
Anxiety	3	0	2	5	1	2	0	1
Depression	0	0	0	5	0	0	0	1
Hours of sleep	9	8	8	6	8	6	8	8

**Table 22:** Patient 20 (Female/41/heroin).

Report	Baseline	Infusion 1	Infusion 2	Infusion 3	Infusion 4	Infusion 5	Infusion 6	Infusion 7
Cravings	5	3	0	0	0	0	0	0
Anxiety	4	3	2	0	0	2	2	0
Depression	4	0	0	0	0	2	0	0
Hours of sleep	7	5	5	5	6	6	7	7

**Table 23:** Patient 21 (Female/26/heroin/meth).

Report	Baseline	Infusion 1	Infusion 2	Infusion 3	Infusion 4	Infusion 5	Infusion 6	Infusion 7
Cravings	8	2	2	0	0	0	0	0
Anxiety	6	0	3	2	0	0	0	0
Depression	3	3	2	0	0	0	0	0
Hours of sleep	5	8	7	4	8	8	7	8

**Table 24:** Patient 22 (Male/42/ETOH/benzos).

Report	Baseline	Infusion 1	Infusion 2	Infusion 3	Infusion 4	Infusion 5	Infusion 6	Infusion 7
Cravings	6	5	0	10	0	1	0	0
Anxiety	3	7	5	10	5	6	5	4
Depression	6	7	0	10	0	2	0	0
Hours of sleep	7	8	8	5	7	7	11	8

**Table 25:** Patient 23 (Male/33/meth/ETOH).

Report	Baseline	Infusion 1	Infusion 2	Infusion 3	Infusion 4	Infusion 5	Infusion 6	Infusion 7
Cravings	8	3	1	1	1	1	1	0
Anxiety	10	0	1	0	2	1	0	0
Depression	1	1	0	0	1	1	0	0
Hours of sleep	8	8	8	8	8	8	8	8

**Table 26:** Patient 24 (Male/42/ETOH).

Report	Baseline	Infusion 1	Infusion 2	Infusion 3	Infusion 4	Infusion 5	Infusion 6	Infusion 7
Cravings	5	3	1	0	0	0	0	0
Anxiety	3	2	2	0	0	0	2	2
Depression	0	0	0	0	0	0	0	0
Hours of sleep	7	7	5	7	6	8	7	6

**Table 27:** Patient 25 (Male/33/meth).

Report	Baseline	Infusion 1	Infusion 2	Infusion 3	Infusion 4	Infusion 5	Infusion 6	Infusion 7
Cravings	5	0	0	0	0	0	0	0
Anxiety	5	3	3	3	0	2	0	4
Depression	3	2	2	3	0	3	2	3
Hours of sleep	8	8	8	8	8	8	9	8

**Table 28:** Patient 26 (Male/35/heroin/opiates).

Report	Baseline	Infusion 1	Infusion 2	Infusion 3	Infusion 4	Infusion 5	Infusion 6	Infusion 7
Cravings	6	0	2	2	3	0	0	0
Anxiety	1	2	0	1	3	2	0	1
Depression	0	0	3	1	1	0	0	0
Hours of sleep	7	7	7	8	7	7	7	7

**Table 29:** Patient 27 (Male/44/heroin/meth).

Report	Baseline	Infusion 1	Infusion 2	Infusion 3	Infusion 4	Infusion 5	Infusion 6	Infusion 7
Cravings	4	0	0	0	8	0	0	0
Anxiety	7	0	1	0	0	0	3	0
Depression	0	0	0	0	0	0	0	0
Hours of sleep	3	5	7	8	8	5	8	8

**Table 30:** Patient 28 (Male/40/meth).

Report	Baseline	Infusion 1	Infusion 2	Infusion 3	Infusion 4	Infusion 5	Infusion 6	Infusion 7
Cravings	4	7	7	0	0	0	0	0
Anxiety	7	7	10	0	6	5	4	0
Depression	0	7	0	0	0	0	0	0
Hours of sleep	6	6	7	7	7	7	7	8

**Table 31:** Patient 29 (Male/36/meth/ETOH).

Report	Baseline	Infusion 1	Infusion 2	Infusion 3	Infusion 4	Infusion 5	Infusion 6	Infusion 7
Cravings	5	0	0	0	0	0	0	0
Anxiety	4	0	0	0	0	2	0	0
Depression	0	0	0	0	0	2	0	0
Hours of sleep	8	8	8	8	8	8	8	8

**Table 32:** Patient 30 (Male/meth/ETOH).

Report	Baseline	Infusion 1	Infusion 2	Infusion 3	Infusion 4	Infusion 5	Infusion 6	Infusion 7
Cravings	1	1	0	0	0	0	0	0
Anxiety	1	1	6	5	0	0	0	0
Depression	3	1	0	0	0	0	0	0
Hours of sleep	6	7	4	5	8	6	6	8

**Table 33:** Patient 31 (Male/28/meth/heroin).

Report	Baseline	Infusion 1	Infusion 2	Infusion 3	Infusion 4	Infusion 5	Infusion 6	Infusion 7
Cravings	10	6	0	0	0	0	0	0
Anxiety	4	2	8	0	0	0	0	0
Depression	0	0	0	0	0	0	0	0
Hours of sleep	6	8	8	8	6	5	6	8

**Table 34:** Patient 32 (Male/23/heroin/ETOH/crack).

Report	Baseline	Infusion 1	Infusion 2	Infusion 3	Infusion 4	Infusion 5	Infusion 6	Infusion 7
Cravings	5	4	4	4	8	2	0	0
Anxiety	5	3	4	3	6	6	0	2
Depression	5	6	4	4	5	5	2	1
Hours of sleep	5	5	6	5	4	7	7	7

**Table 35:** Patient 33 (Male/31/meth).

Report	Baseline	Infusion 1	Infusion 2	Infusion 3	Infusion 4	Infusion 5	Infusion 6	Infusion 7
Cravings	7	3	1	0	0	0	2	1
Anxiety	3	6	6	6	6	5	2	1
Depression	0	0	4	3	8	4	0	1
Hours of sleep	8	7	8	5	4	6	4	8

**Table 36:** Patient 34 (Male/30/ETOH/marijuana).

Report	Baseline	Infusion 1	Infusion 2	Infusion 3	Infusion 4	Infusion 5	Infusion 6	Infusion 7
Cravings	5	3	0	0	0	0	0	0
Anxiety	3	3	0	3	3	3	3	3
Depression	5	3	3	3	3	3	3	2
Hours of sleep	8	8	8	8	8	8	8	8

**Table 37:** Patient 35 (Male/37/meth).

Report	Baseline	Infusion 1	Infusion 2	Infusion 3	Infusion 4	Infusion 5	Infusion 6	Infusion 7
Cravings	5	0	0	0	0	0	0	0
Anxiety	6	8	7	2	4	0	0	0
Depression	2	0	4	0	6	0	0	0
Hours of sleep	4	8	8	8	6	8	8	8

**Table 38:** Patient 36 (Male/30/heroin/meth/duster/ETOH).

Report	Baseline	Infusion 1	Infusion 2	Infusion 3	Infusion 4	Infusion 5	Infusion 6	Infusion 7
Cravings	4	0	4	0	0	0	0	0
Anxiety	4	0	0	0	0	4	0	0
Depression	4	0	0	0	0	0	0	0
Hours of sleep	4	4	8	8	8	8	8	5

**Table 39:** Patient 37 (Male/31/heroin/meth/benzos).

Report	Baseline	Infusion 1	Infusion 2	Infusion 3	Infusion 4	Infusion 5	Infusion 6	Infusion 7
Cravings	5	0	2	0	0	0	0	1
Anxiety	3	0	2	5	3	0	3	1
Depression	0	0	0	0	0	0	0	1
Hours of sleep	2	3	4	7	6	8	7	6

**Table 40:** Patient 38 (Male/32/meth).

Report	Baseline	Infusion 1	Infusion 2	Infusion 3	Infusion 4	Infusion 5	Infusion 6	Infusion 7
Cravings	6	3	1	1	0	0	0	0
Anxiety	3	2	1	2	2	1	1	1
Depression	1	2	1	2	1	1	0	0
Hours of sleep	8	6	4	4	6	7	8	5

**Table 41:** Patient 39 (Male/41/meth).

Report	Baseline	Infusion 1	Infusion 2	Infusion 3	Infusion 4	Infusion 5	Infusion 6	Infusion 7
Cravings	5	0	0	0	0	4	0	0
Anxiety	3	0	0	0	3	4	4	4
Depression	0	0	0	0	0	0	0	0
Hours of sleep	8	8	8	6	8	7	6	6

**Table 42:** Patient 40 (Male/25/heroin/meth).

Report	Baseline	Infusion 1	Infusion 2	Infusion 3	Infusion 4	Infusion 5	Infusion 6	Infusion 7
Cravings	7	0	0	0	0	0	0	0
Anxiety	5	8	6	2	1	6	5	5
Depression	6	6	4	2	1	0	0	0
Hours of sleep	4	4	8	6	5	5	8	8

**Table 43:** Patient 41 (Male/42/meth).

Report	Baseline	Infusion 1	Infusion 2	Infusion 3	Infusion 4	Infusion 5	Infusion 6	Infusion 7
Cravings	5	2	2	2	2	0	0	0
Anxiety	6	6	5	4	2	2	2	0
Depression	6	6	5	4	2	2	2	0
Hours of sleep	10	10	10	10	10	2	2	6

**Table 44:** Patient 42 (Male/29/meth).

Report	Baseline	Infusion 1	Infusion 2	Infusion 3	Infusion 4	Infusion 5	Infusion 6	Infusion 7
Cravings	6	2	2	1	1	0	0	0
Anxiety	0	0	0	0	0	0	0	0
Depression	4	6	0	0	0	0	0	0
Hours of sleep	4	8	8	8	8	8	7	7

**Table 45:** Patient 43 (Male/40/meth).

Report	Baseline	Infusion 1	Infusion 2	Infusion 3	Infusion 4	Infusion 5	Infusion 6	Infusion 7
Cravings	8	4	4	0	0	0	0	0
Anxiety	10	7	7	4	2	3	1	4
Depression	9	7	7	2	2	2	0	0
Hours of sleep	3	5	6	8	6	7	3	6

**Table 46:** Patient 44 (Male/24/heroin/meth).

Report	Baseline	Infusion 1	Infusion 2	Infusion 3	Infusion 4	Infusion 5	Infusion 6	Infusion 7
Cravings	8	0	2	2	1	0	0	0
Anxiety	4	4	3	3	3	3	2	0
Depression	5	3	3	3	3	3	2	0
Hours of sleep	6	6	6	6	6	6	6	8

**Table 47:** Patient 45 (Male/30/heroin/opiates/benzos).

Report	Baseline	Infusion 1	Infusion 2	Infusion 3	Infusion 4	Infusion 5	Infusion 6	Infusion 7
Cravings	7	6	3	1	0	0	0	0
Anxiety	7	6	0	3	1	2	2	2
Depression	2	0	3	2	0	0	0	0
Hours of sleep	6	6	9	7	8	8	8	8

**Table 48:** Patient 46 (Male/32/heroin).

Report	Baseline	Infusion 1	Infusion 2	Infusion 3	Infusion 4	Infusion 5	Infusion 6	Infusion 7
Cravings	8	6	0	0	0	0	0	1
Anxiety	2	2	0	0	3	3	0	2
Depression	2	0	0	0	0	0	0	1
Hours of sleep	8	4	8	7	8	7	8	7

**Table 49:** Patient 47 (Male/46/meth).

Report	Baseline	Infusion 1	Infusion 2	Infusion 3	Infusion 4	Infusion 5	Infusion 6	Infusion 7
Cravings	5	3	0	0	0	0	0	0
Anxiety	8	3	5	2	4	3	3	1
Depression	5	5	7	5	7	5	5	2
Hours of sleep	4	4	7	7	7	6	7	7

**Table 50:** Patient 48 (Male/45/ETOH).

Report	Baseline	Infusion 1	Infusion 2	Infusion 3	Infusion 4	Infusion 5	Infusion 6	Infusion 7
Cravings	5	4	3	0	0	0	0	0
Anxiety	3	5	2	4	4	3	4	4
Depression	5	5	3	0	4	3	4	4
Hours of sleep	5	7	7	6	7	5	7	7

**Table 51:** Patient 49 (Male/28/meth/ETOH).

Report	Baseline	Infusion 1	Infusion 2	Infusion 3	Infusion 4	Infusion 5	Infusion 6	Infusion 7
Cravings	8	3	0	0	0	0	0	0
Anxiety	3	2	0	0	0	0	1	0
Depression	0	0	0	0	0	0	0	0
Hours of sleep	8	8	8	8	8	8	8	8

**Table 52:** Patient 50 (Male/33/heroin).

Report	Baseline	Infusion 1	Infusion 2	Infusion 3	Infusion 4	Infusion 5	Infusion 6	Infusion 7
Cravings	9	0	0	0	0	0	0	0
Anxiety	2	2	0	3	2	2	2	2
Depression	0	0	0	0	0	0	0	0
Hours of sleep	7	7	8	8	8	8	7	7
